# Supplementary material for: Antimicrobial-resistance of Escherichia coli in dogs and cats: A scoping review
Source: PLoS One. 2025 May 30;20(5):e0323246. doi: 10.1371/journal.pone.0323246 (PMC12124559; doi:10.1371/journal.pone.0323246)
Supplement: S1 Appendix — (PDF) [file pone.0323246.s003.pdf]

S1 Appendix A. Post-protocol deviation

The information regarding the specific Clinical and Laboratory Standards Institute guidelines implemented in the study and the editions used was not gathered. This deviation was due to practical constraints in extracting detailed information about specific guidelines and editions.
