# Supplementary material for: Antimicrobial-resistance of Escherichia coli in dogs and cats: A scoping review
Source: PLoS One. 2025 May 30;20(5):e0323246. doi: 10.1371/journal.pone.0323246 (PMC12124559; doi:10.1371/journal.pone.0323246)
Supplement: S2 Appendix — (PDF) [file pone.0323246.s004.pdf]

S2 Appendix B. Data items

The publication details gathered from all included studies encompassed the commencement and conclusion years of the data utilized in the study, the country or countries involved, the level of the study (e.g., individual facility, regional, national, or international), and the affiliation of the first author.

To address the primary research questions, reviewers provided answers to specific inquiries, which included reporting the study design (e.g., experimental, observational, simulation-modeling, etc.), defining or documenting the source population of animals, disclosing the clinical history of the animals (e.g., clinically healthy, sick, or both), noting recent antimicrobial history, specifying the origin of samples (e.g., households, veterinary clinic, shelter or kennel, etc.), identifying the bacteria isolated in the study (e.g., *E. coli*, extended-spectrum β-lactamase-producing *E. coli*, or both), specifying the specimen from which *E. coli* was isolated (e.g., urinary tract, non-urinary tract), determining the level at which AMR was based (e.g., animal, isolate, specimen, both, or unclear), enumerating the number of animals (dogs, cats, or both) subjected to AST, counting the number of *E. coli* isolates from dogs or cats that had AST, specifying the method of AST used in the study (e.g., disk diffusion, broth microdilution, Epsilometer test, etc.), indicating if the minimum inhibition concentration (MIC) was reported, noting the number of antimicrobials tested against *E. coli* isolates, discerning if *E. coli* was tested for carbapenem (e.g., imipenem, meropenem, ertapenem, etc.), reporting on multi-drug resistance (MDR) for *E. coli* that had AST, confirming if the reported MDR adhered to the standard definition (i.e., resistance to one or more drugs in three or more classes), specifying the guidelines used in classifying AST (e.g., Clinical and Laboratory Standard Institute, European Committee on Antimicrobial Susceptibility Testing, etc.), outlining how the intermediate category was handled (e.g., reported separately, combined

with resistance data, combined with susceptible data, etc.), and determining if *E. coli* isolates were tested against the same panel of antimicrobials.

All questions requiring enumeration were entered into the data charting form in numerical format, while the remaining questions featured multiple-choice answers, including selecting all that apply.
