# Supplementary material for: Antimicrobial-resistance of Escherichia coli in dogs and cats: A scoping review
Source: PLoS One. 2025 May 30;20(5):e0323246. doi: 10.1371/journal.pone.0323246 (PMC12124559; doi:10.1371/journal.pone.0323246)
Supplement: S1 Table — (PDF) [file pone.0323246.s005.pdf]

S1 Table. **Summary and data charting of articles: excluded articles with reasons, eligible articles, and charted data from included studies**

Level	Screening	# Articles excluded	Reasons for exclusions
1	Title and abstract	1 to 22	These studies were not published on or after January 01, 1990
		23 to 35	These studies are not available in English
		36 to 273	These studies did not describe dogs and/or cats
		274 to 391	These studies did not describe E. coli
		392 to 416	These studies did not describe antimicrobial resistance
		417 to 449	These studies are narrative reviews
		450 to 451	These studies are scoping reviews
		452 to 453	These studies are meta-analysis
		454 to 462	These studies are systematic reviews and meta-analyses
		463	This study is a scoping review and meta-analysis
		464 to 480	These studies are classified as another study (e.g., conference proceedings, commentary, editorial, letter to the editor, news reports, expert opinion)
		481 to 564	These are descriptive studies (e.g., case reports or case series)
		565 to 990	These studies are to understand the molecular basis of resistance
		991 to 1052	These studies are other types of studies (e.g., lab studies for validation of antimicrobial susceptibility)
2	Full text	1053 to 1061	The full text of these studies is not available
		1062 to 1064	These studies did not describe dogs and/or cats
		1065	These studies did not describe E. coli
		1066 to 1071	These studies did not describe antimicrobial resistance
		1072	This study is a narrative review
		1073	The study is a scoping review
		1074 to 1075	These are descriptive studies (e.g., case reports or case series)
		1076 to 1086	These studies are to understand the molecular basis of resistance
		1087 to 1092	The studies are other types of studies (e.g., validation of AST methodologies)

Level 1, Form level_1_screening, Is the full text published on or after 01-Jan-1990? -> No

1. Gaines, S A, Rollins, L D, Silver, R P, Washington, M (1978). Effect of low concentrations of dihydrostreptomycin on drug resistance in enteric bacteria. *Antimicrobial agents and chemotherapy*, 14(2), 252

2. Beaucage, C M, Fox, J G (1979). Transmissible antibiotic resistance in Salmonella isolated from random-source cats purchased for use in research. *American Journal of Veterinary Research*, 40(6), 849

3. CH Clark (1977). Bacterial sensitivity to the aminoglycosides. *Modern veterinary practice*, 58(8), #Pages#

4. RE Wooley, JL Blue (1976). Bacterial isolations from canine and feline urine. *Modern veterinary practice*, 57(7), #Pages#

5. RE Wooley, JL Blue (1976). Quantitative and bacteriological studies of urine specimens from canine and feline urinary tract infections. *Journal of clinical microbiology*, 4(4), #Pages#

6. Rollins, L D, Gaines, S A, Pocurull, D W, Mercer, H D (1975). Animal model for determining the no-effect level of an antimicrobial drug on drug resistance in the lactose-fermenting enteric flora. *Antimicrobial agents and chemotherapy*, 7(5), 661

7. Lewis, L D (1988). Comparison of virulence factors and antibiotic resistance profiles of Escherichia coli strains from humans and dogs with urinary tract infections. *Journal of veterinary internal medicine*, 2(1), 54

8. Sparks, S E, Jones, R L, Kilgore, W R (1988). In vitro susceptibility of bacteria to a ticarcillin-clavulanic acid combination. *American journal of veterinary research*, 49(12), 2038

9. RJ Bywater, GH Palmer, JF Buswell, A Stanton (1985). Clavulanate-potentiated amoxycillin: activity in vitro and bioavailability in the dog. *The Veterinary record*, 116(2), #Pages#

10. RM Hogle (1970). Antibacterial-agent sensitivity of bacteria isolated from dogs and cats. *Journal of the American Veterinary Medical Association*, 156(6), #Pages#

11. RS Roy (1972). [R factors in strains of pathogenic enterobacteria isolated from domestic animals and particularly from dogs]. *Canadian journal of comparative medicine : Revue canadienne de medecine comparee*, 36(1), #Pages#

12. Hariharan, H, Barnum, D A, Mitchell, W R (1974). Drug resistance among pathogenic bacteria from animals in Ontario. *Canadian journal of comparative medicine : Revue*

canadienne de medecine comparee, 38(3), 213

13. Hirsh, D C (1973). Multiple antimicrobial resistance in Escherichia coli isolated from the urine of dogs and cats with cystitis. *Journal of the American Veterinary Medical Association*, 162(10), 885

14. Gaines, S A, Rollins, L D, Williams, R D, Selwyn, M (1980). Effect of penicillin and virginiamycin on drug resistance in lactose-fermenting enteric flora. *Antimicrobial agents and chemotherapy*, 17(3), 428

15. Ling, G V, Rohrich, P J, Ruby, A L, Johnson, D L, Jang, S S (1984). Canine urinary tract infections: a comparison of in vitro antimicrobial susceptibility test results and response to oral therapy with ampicillin or with trimethoprim-sulfa. *Journal of the American Veterinary Medical Association*, 185(3), 277

16. Moss, S, Frost, A J (1984). The resistance to chemotherapeutic agents of Escherichia coli from domestic dogs and cats. *Australian veterinary journal*, 61(3), 82

17. A Jones (1964). The Activity Of Ampicillin And Phenethicillin For Bacteria Isolated From Veterinary Sources. *Journal of comparative pathology*, 74(), #Pages#

18. Fox, I W, Hoag, W G, Strout, J (1965). Breed susceptibility, pathogenicity and epidemiology of endemic coliform enteritis in the dog. *Laboratory animal care*, 15(#issue#), 194

19. Hariharan, H., Barnum, D. A. (1974). Antimicrobial drug susceptibility of certain bacterial pathogens from dogs and cats. *Canadian Veterinary Journal*, 15(No.4), 108

20. Roudebush, P., Fales, W. H. (1982). Antibacterial susceptibility of gentamicin resistant organisms recovered from small companion animals. *Journal of the American Animal Hospital Association*, 18(4), 649

21. Wooley, R E, Jones, M S (1983). Action of EDTA-Tris and antimicrobial agent combinations on selected pathogenic bacteria. *Veterinary microbiology*, 8(3), 271

22. Harsh, D C (1973). Multiple antimicrobial resistance in Escherichia coli isolated from the urine of dogs and cats with cystitis *Journal of the American Veterinary Medical Association*, 162(10), 885

Level 1, Form level_1_screening, Is the full text available in English? -> No

23. HC Rutgers, RL Stepien, CM Elwood, KW Simpson, RM Batt (1994). Enrofloxacin treatment of gram-negative infections. *The Veterinary record*, 135(15), #Pages#

24. Sternberg, S (1999). Antimicrobial resistance in bacteria from pets and horses. *Acta veterinaria Scandinavica. Supplementum*, 92(#issue#), 37

25. A Wissing, J Nicolet, P Boerlin (2001). The current antimicrobial resistance situation in Swiss veterinary medicine. *Schweizer Archiv fur Tierheilkunde*, 143(10), #Pages#

26. Scott Weese, J (2008). Antimicrobial resistance in companion animals. *Animal health research reviews*, 9(2), 169

27. Snell, C. B., Winston, J. A., Quimby, J. M., Diaz-Campos, D., Gibson, J. F., Byron, J. M., Rudinsky, A. J., Justice, S. S., Harrison, A. (2022). Escherichia coli probiotic exhibits in vitro growth limiting effects on clinical feline uropathogenic E coli isolates. *American Journal of Veterinary Research*, 83(7), #Pages#

28. Carvalho, V. M., Spinola, T., Tavolari, F., Irino, K., Oliveira, R. M., Ramos, M. C. C. (2014). Urinary tract infection (UTI) in dogs and cats: etiology and antimicrobial resistance. *Pesquisa Veterinária Brasileira*, 34(1), 62

29. Ishii, J. B., Freitas, J. C., Arias, M. V. B. (2011). Resistance of bacteria isolated from dogs and cats at Veterinary Hospital of Universidade Estadual de Londrina (2008-2009). *Pesquisa Veterinária Brasileira*, 31(6), 533

30. Habrun, B., Kompes, G., Špičić, S., Račić, I., Cvetnić, Ž. (2011). Bacterial agents, antimicrobial sensitivity and therapy for urinary infections of dogs and cats. *Veterinarska Stanica*, 42(6), 497

31. Zhao XiangSheng, Sun Yang, Ji Xue, Liu Jun, Zhu LingWei, Tong PanPan, Feng ShuZhang (2014). Antibiotic resistance of Escherichia coli isolated from dogs. *Chinese Journal of Zoonoses*, 30(3), 268

32. Basso, P. C., Raiser, A. G., Brun, M. V., Santos, L. R., Muller, D. C. de M., Trindade, A. B. (2009). Bronchoalveolar lavage fluid bacterial isolation and sensibility in health and sick dogs. *Ciência Animal Brasileira*, 10(3), 947

33. Duque, M, Uribe, N, Buitrago, J (2021). Patrones de resistencia en agentes bacterianos involucrados en otitis caninas en Medellín, Colombia, durante 2019: análisis retrospectivo *Revista de la Facultad de Medicina Veterinaria y de Zootecnia*, 68(3), #Pages#

34. Weiss, H-E, Bertl, F (1991). Therapy of canine and feline enteritis caused by E. coli resistant to antimicrobial agents by oral administration of autologous vaccines. *Praktische Tierarzt*, 72(1), 12

35. Arias, MVB, Carrilho, CMDD (2012). Antimicrobial resistance in animals and in human being. There is reason for concern? *Semina-Ciencias Agrarias*, 33(2), 775

Level 1, Form level_1_screening, Does the title/abstract describe dogs and/or cats? -> No

36. Dorn, C R, Tsutakawa, R K, Fein, D, Burton, G C, Blenden, D C (1975). Antibiotic resistance patterns of Escherichia coli isolated from farm families consuming home-raised meat. *American journal of epidemiology*, 102(4), 319

37. Alexander, W J, Cobbs, C G, Curtiss, R (1980). Modification of bacterial serum susceptibility by rifampin. *Infection and immunity*, 28(3), 923

38. Cloeckaert, A, Baucheron, S, Flaujac, G, Schwarz, S, Kehrenberg, C, Martel, J L, Chaslus-Dancla, E (2000). Plasmid-mediated florfenicol resistance encoded by the floR gene in Escherichia coli isolated from cattle. *Antimicrobial agents and chemotherapy*, 44(10), 2858

39. Auwerx, Joeri, North, Thomas W, Preston, Bradley D, Klarmann, George J, De Clercq, Erik, Balzarini, Jan (2002). Chimeric human immunodeficiency virus type 1 and feline immunodeficiency virus reverse transcriptases: role of the subunits in resistance/sensitivity to non-nucleoside reverse transcriptase inhibitors. *Molecular pharmacology*, 61(2), 400

40. Kaszanyitzky, Eva J, Tarpai, A, Janosi, Sz, Papp, Melitta, Skare, J, Semjen, G (2002). Development of an antibiotic resistance monitoring system in Hungary. *Acta veterinaria Hungarica*, 50(2), 189

41. Barton, Mary D, Pratt, Rachael, Hart, Wendy S (2003). Antibiotic resistance in animals. *Communicable diseases intelligence quarterly report*, 27 Suppl(#issue#), S121

42. Jost, B Helen, Field, Adam C, Trinh, Hien T, Songer, J Glenn, Billington, Stephen J (2003). Tylosin resistance in Arcanobacterium pyogenes is encoded by an erm X determinant. *Antimicrobial agents and chemotherapy*, 47(11), 3519

43. JL Smith, PM Fratamico, NW Gunther (2007). Extraintestinal pathogenic Escherichia coli. *Foodborne pathogens and disease*, 4(2), #Pages#

44. Schwarz, Stefan, Werckenthin, Christiane, Alesik, Eva, Wieler, Lothar H, Wallmann, Jurgen (2007). Susceptibility of bacterial pathogens against lincomycin/spectinomycin (1/2), penicillin G/neomycin (1/1), and penicillin G/dihydrostreptomycin (1/1) as determined in the BfT-GermVet monitoring program 2004-2006. *Berliner und Munchener tierarztliche Wochenschrift*, 120(9-10), 363

45. P Silley (2007). ESBL-producing bacteria isolated from companion animals. *The Veterinary record*, 161(23), #Pages#

46. W Sianglum, W Wonglumsom, P Srimanote, K Kittiniyom (2007). Analysis of gyrA mutations related to quinolone resistance in Escherichia coli isolates originating from pet, human, vegetable and ice in Bangkok and vicinity. *The Southeast Asian journal of tropical medicine and public health*, 38(6), #Pages#

47. Guenther, Sebastian, Grobbel, Mirjam, Lubke-Becker, Antina, Goedecke, Andreas, Friedrich, Nicole D, Wieler, Lothar H, Ewers, Christa (2010). Antimicrobial resistance profiles of Escherichia coli from common European wild bird species. *Veterinary microbiology*, 144(1-2), 219

48. BA Rogers, HE Sidjabat, DL Paterson (2011). Escherichia coli O25b-ST131: a pandemic, multiresistant, community-associated strain. *The Journal of antimicrobial chemotherapy*, 66(1), #Pages#

49. CP Murphy, RJ Reid-Smith, P Boerlin, JS Weese, JF Prescott, N Janecko, L Hassard, SA McEwen (2010). Escherichia coli and selected veterinary and zoonotic pathogens isolated from environmental sites in companion animal veterinary hospitals in southern Ontario. *The Canadian veterinary journal = La revue veterinaire canadienne*, 51(9), #Pages#

50. Chen, Yen-Ping, Lee, Shu-Hwae, Chou, Chung-Hsi, Tsai, Hsiang-Jung (2012). Detection of florfenicol resistance genes in Riemerella anatipestifer isolated from ducks and geese. *Veterinary microbiology*, 154(3-4), 325

51. Hordijk, Joost, Veldman, Kees, Dierikx, Cindy, van Essen-Zandbergen, Alieda, Wagenaar, Jaap A, Mevius, Dik (2012). Prevalence and characteristics of quinolone resistance in Escherichia coli in veal calves. *Veterinary microbiology*, 156(1-2), 136

52. E Meyer, P Gastmeier, A Kola, F Schwab (2012). Pet animals and foreign travel are risk factors for colonisation with extended-spectrum β-lactamase-producing Escherichia coli. *Infection*, 40(6), #Pages#

53. Ho, P L, Chan, Jane, Lo, W U, Law, Pierra Y, Chow, K H (2013). Plasmid-mediated fosfomycin resistance in Escherichia coli isolated from pig. *Veterinary microbiology*, 162(2-4), 964

54. S Abraham, HS Wong, J Turnidge, JR Johnson, DJ Trott (2014). Carbapenemase-producing bacteria in companion animals: a public health concern on the horizon. *The Journal of antimicrobial chemotherapy*, 69(5), #Pages#

55. LP Carmo, LR Nielsen, PM da Costa, L Alban (2014). Exposure assessment of extended-spectrum beta-lactamases/AmpC beta-lactamases-producing Escherichia coli in meat in Denmark. *Infection ecology & epidemiology*, 4(), #Pages#

56. B Guerra, J Fischer, R Helmuth (2014). An emerging public health problem: acquired carbapenemase-producing microorganisms are present in food-producing animals, their environment, companion animals and wild birds. *Veterinary microbiology*, 171(3-4), #Pages#

57. X Chen, L He, Y Li, Z Zeng, Y Deng, Y Liu, JH Liu (2014). Complete sequence of a F2:A-:B- plasmid pHN3A11 carrying rmtB and qepA, and its dissemination in China. *Veterinary*

microbiology, 174(1-2), #Pages#

58. Maddox, T W, Clegg, P D, Williams, N J, Pinchbeck, G L (2015). Antimicrobial resistance in bacteria from horses: Epidemiology of antimicrobial resistance. *Equine veterinary journal*, 47(6), 756

59. RM Gioia-Di Chiacchio, MP Cunha, RM Sturn, LZ Moreno, AM Moreno, CB Pereira, FH Martins, MR Franzolin, RM Piazza, T Knöbl (2016). Shiga toxin-producing Escherichia coli (STEC): Zoonotic risks associated with psittacine pet birds in home environments. *Veterinary microbiology*, 184(), #Pages#

60. D Parker, MK Sniatynski, D Mandrusiak, JE Rubin (2016). Extended-spectrum β-lactamase producing Escherichia coli isolated from wild birds in Saskatoon, Canada. *Letters in applied microbiology*, 63(1), #Pages#

61. CA Borges, LG Beraldo, RP Maluta, MV Cardozo, KB Barboza, EA Guastalli, S Kariyawasam, C DebRoy, FA Ávila (2017). Multidrug-resistant pathogenic Escherichia coli isolated from wild birds in a veterinary hospital. *Avian pathology : journal of the W.V.P.A*, 46(1), #Pages#

62. Huang, Li, Yuan, Hui, Liu, Ma-Feng, Zhao, Xin-Xin, Wang, Ming-Shu, Jia, Ren-Yong, Chen, Shun, Sun, Kun-Feng, Yang, Qiao, Wu, Ying, Chen, Xiao-Yue, Cheng, An-Chun, Zhu, De-Kang (2017). Type B Chloramphenicol Acetyltransferases Are Responsible for Chloramphenicol Resistance in Riemerella anatipestifer, China. *Frontiers in microbiology*, 8(#issue#), 297

63. Y Doi, A Iovleva, RA Bonomo (2017). The ecology of extended-spectrum β-lactamases (ESBLs) in the developed world. *Journal of travel medicine*, 24(suppl_1), #Pages#

64. N Gay, O Belmonte, JM Collard, M Halifa, MI Issack, S Mindjae, P Palmyre, AA Ibrahim, H Rasamoelina, L Flachet, L Filleul, E Cardinale (2017). Review of Antibiotic Resistance in the Indian Ocean Commission: A Human and Animal Health Issue. *Frontiers in public health*, 5(), #Pages#

65. Kylie, Jennifer, McEwen, Scott A, Boerlin, Patrick, Reid-Smith, Richard J, Weese, J Scott, Turner, Patricia V (2017). Prevalence of antimicrobial resistance in fecal Escherichia coli and Salmonella enterica in Canadian commercial meat, companion, laboratory, and shelter rabbits (Oryctolagus cuniculus) and its association with routine antimicrobial use in commercial meat rabbits. *Preventive veterinary medicine*, 147(#issue#), 53

66. Badi, Souhir, Cremonesi, Paola, Abbassi, Mohamed Salah, Ibrahim, Chourouk, Snoussi, Majdi, Bignoli, Giulia, Luini, Mario, Castiglioni, Bianca, Hassen, Abdennaceur (2018). Antibiotic resistance phenotypes and virulence-associated genes in Escherichia coli isolated from animals and animal food products in Tunisia. *FEMS microbiology letters*, 365(10),

#Pages#

67. A Vikram, JW Schmidt (2018). Functional bla Sequences Are Present in U.S. Beef Cattle Feces Regardless of Antibiotic Use. *Foodborne pathogens and disease*, 15(7), #Pages#

68. PS Pontes, SDA Coutinho, RO Iovine, MPV Cunha, T Knöbl, VM Carvalho (2018). Survey on pathogenic Escherichia coli and Salmonella spp. in captive cockatiels (Nymphicus hollandicus). *Brazilian journal of Microbiology: [publication of the Brazilian Society for Microbiology]*, 49 Suppl 1(Suppl 1), #Pages#

69. CA Kennedy, C Walsh, M Karczmarczyk, S O'Brien, N Akasheh, M Quirke, S Farrell-Ward, T Buckley, U Fogherty, K Kavanagh, CT Parker, T Sweeney, S Fanning (2018). Multi-drug resistant Escherichia coli in diarrhoeagenic foals: Pulsotyping, phylotyping, serotyping, antibiotic resistance and virulence profiling. *Veterinary microbiology*, 223(), #Pages#

70. Royden, Alexandra, Ormandy, Emma, Pinchbeck, Gina, Pascoe, Ben, Hitchings, Matthew D, Sheppard, Samuel K, Williams, Nicola J (2019). Prevalence of faecal carriage of extended-spectrum beta-lactamase (ESBL)-producing Escherichia coli in veterinary hospital staff and students. *Veterinary record open*, 6(1), e000307

71. Jung, Hi Eun, Oh, Ji Eun, Lee, Heung Kyu (2019). Cell-Penetrating Mx1 Enhances Anti-Viral Resistance against Mucosal Influenza Viral Infection. *Viruses*, 11(2), #Pages#

72. van Duijkeren, Engeline, Schwarz, Christine, Bouchard, Damien, Catry, Boudewijn, Pomba, Constanca, Baptiste, Keith Edward, Moreno, Miguel A, Rantala, Merja, Ruzauskas, Modestas, Sanders, Pascal, Teale, Christopher, Wester, Astrid L, Ignate, Kristine, Kunsagi, Zoltan, Jukes, Helen (2019). The use of aminoglycosides in animals within the EU: development of resistance in animals and possible impact on human and animal health: a review. *The Journal of antimicrobial chemotherapy*, 74(9), 2480

73. W Wetzker, Y Pfeifer, S Wolke, A Haselbeck, R Leistner, A Kola, P Gastmeier, F Salm (2019). Extended-Spectrum Beta-Lactamase (ESBL)-Producing <i>Escherichia coli</i> Isolated from Flies in the Urban Center of Berlin, Germany. *International journal of environmental research and public health*, 16(9), #Pages#

74. FM Lima, F de Paulo Daurelio, ER Mucci, CM Ahagon, AM Dos Santos Carmo, A Eterovic, L Fernando Dos Santos, MR Tiba-Casas, CH Camargo, MC Cergole-Novella (2019). Epidemiology and genetic screening of diarrheagenic Escherichia coli among symptomatic and asymptomatic children. *Journal of medical microbiology*, 68(7), #Pages#

75. O Goławska, M Zając, A Maluta, P Pristas, Ľ Hamarová, D Wasyl (2019). Complex bacterial flora of imported pet tortoises deceased during quarantine: Another zoonotic threat? *Comparative immunology, microbiology and infectious diseases*, 65(), #Pages#

76. Mughini-Gras, Lapo, Dorado-Garcia, Alejandro, van Duijkeren, Engeline, van den Bunt, Gerrita, Dierikx, Cindy M, Bonten, Marc J M, Bootsma, Martin C J, Schmitt, Heike, Hald,

Tine, Evers, Eric G, de Koeijer, Aline, van Pelt, Wilfrid, Franz, Eelco, Mevius, Dik J, Heederik, Dick J J (2019). Attributable sources of community-acquired carriage of Escherichia coli containing beta-lactam antibiotic resistance genes: a population-based modelling study. *The Lancet. Planetary health*, 3(8), e357

77. P Zhang, J Wang, X Wang, X Bai, J Ma, R Dang, Y Xiong, S Fanning, L Bai, Z Yang (2019). Characterization of Five <i>Escherichia coli</i> Isolates Co-expressing ESBL and MCR-1 Resistance Mechanisms From Different Origins in China. *Frontiers in microbiology*, 10(), #Pages#

78. L Varriale, TP Russo, A Pace, S Mediatore, L Borrelli, A Santaniello, LF Menna, A Fioretti, L Dipineto (2019). Microbiological survey of sugar gliders (Petaurus breviceps) kept as pets in Italy. *Letters in applied microbiology*, 69(6), #Pages#

79. H LE Huy, N Koizumi, TTH Ung, TT LE, HLK Nguyen, PVM Hoang, CN Nguyen, TM Khong, F Hasebe, T Haga, MTQ LE, K Hirayama, K Miura (2020). Antibiotic-resistant Escherichia coli isolated from urban rodents in Hanoi, Vietnam. *The Journal of veterinary medical science*, 82(5), #Pages#

80. K Kawamura, N Nagano, M Suzuki, JI Wachino, K Kimura, Y Arakawa (2017). ESBL-producing <i>Escherichia coli</i> and Its Rapid Rise among Healthy People. *Food safety (Tokyo, Japan)*, 5(4), #Pages#

81. Reynolds, Liam J, Sala-Comorera, Laura, Martin, Niamh A, Nolan, Tristan M, Stephens, Jayne H, Gitto, Aurora, O'Hare, Gregory M P, O'Sullivan, John J, Meijer, Wim G (2020). Correlation between antimicrobial resistance and faecal contamination in small urban streams and bathing waters. *The Science of the total environment*, 739(#issue#), 140242

82. L Steger, M Rinder, R Korbel (2020). [Phenotypical antibiotic resistances of bacteriological isolates originating from pet, zoo and falconry birds]. *Tierarztliche Praxis. Ausgabe K, Kleintiere/Heimtiere*, 48(4), #Pages#

83. Camargo, Livia Roberta Piedade, de Carvalho, Vania Maria, Diaz, Ingrit Elida Collantes, Paciencia, Mateus Luis Barradas, Frana, Sergio Alexandre, Younes, Riad Naim, Varella, Antonio Drauzio, Reis, Luiz Fernando Lima, Suffredini, Ivana Barbosa (2020). Susceptibility of virulent and resistant Escherichia coli strains to non-polar and polar compounds identified in Microplumeria anomala. *Veterinary world*, 13(7), 1376

84. M Nichols, L Stevenson, L Koski, C Basler, M Wise, L Whitlock, L Francois Watkins, CR Friedman, J Chen, K Tagg, L Joseph, H Caidi, K Patel, B Tolar, K Hise, A Classon, O Ceric, R Reimschuessel, IT Williams (2020). Detecting national human enteric disease outbreaks linked to animal contact in the United States of America. *Revue scientifique et technique (International Office of Epizootics)*, 39(2), #Pages#

85. L Varriale, L Dipineto, TP Russo, L Borrelli, V Romano, S D'Orazio, A Pace, LF Menna, A Fioretti, A Santaniello (2020). Antimicrobial Resistance of <i>Escherichia coli</i> and <i>Pseudomonas aeruginosa</i> from Companion Birds. *Antibiotics (Basel, Switzerland)*, 9(11), #Pages#

86. Suwono, Beneditta, Eckmanns, Tim, Kaspar, Heike, Merle, Roswitha, Zacher, Benedikt, Kollas, Chris, Weiser, Armin A, Noll, Ines, Feig, Marcel, Tenhagen, Bernd-Alois (2021). Cluster analysis of resistance combinations in Escherichia coli from different human and animal populations in Germany 2014-2017. *PloS one*, 16(1), e0244413

87. S Zhang, M Abbas, MU Rehman, M Wang, R Jia, S Chen, M Liu, D Zhu, X Zhao, Q Gao, B Tian, A Cheng (2021). Updates on the global dissemination of colistin-resistant Escherichia coli: An emerging threat to public health. *The Science of the total environment*, 799(), #Pages#

88. M Dec, D Stepien-Pysniak, K Szczepaniak, B Turchi, R Urban-Chmiel (2022). Virulence Profiles and Antibiotic Susceptibility of <i>Escherichia coli</i> Strains from Pet Reptiles. *Pathogens (Basel, Switzerland)*, 11(2), #Pages#

89. SD Cole, I Healy, JM Dietrich, LE Redding (2022). Evaluation of canine raw food products for the presence of extended-spectrum beta-lactamase- and carbapenemase-producing bacteria of the order Enterobacterales. *American journal of veterinary research*, 83(9), #Pages#

90. Mellor, Kate C, Blackwell, Grace A, Cawthraw, Shaun A, Mensah, Nana E, Reid, Stuart W J, Thomson, Nicholas R, Petrovska, Liljana, Mather, Alison E (2022). Contrasting long-term dynamics of antimicrobial resistance and virulence plasmids in Salmonella Typhimurium from animals. *Microbial genomics*, 8(8), #Pages#

91. ER Wyrsch, RN Bushell, MS Marenda, GF Browning, SP Djordjevic (2022). Global Phylogeny and F Virulence Plasmid Carriage in Pandemic Escherichia coli ST1193. *Microbiology spectrum*, 10(6), #Pages#

92. LJ Toombs-Ruane, JC Marshall, J Benschop, D Drinković, AC Midwinter, PJ Biggs, Z Grange, MG Baker, J Douwes, MG Roberts, NP French, SA Burgess (2023). Extended-spectrum β-lactamase- and AmpC β-lactamase-producing Enterobacterales associated with urinary tract infections in the New Zealand community: a case-control study. *International journal of infectious diseases : IJID : official publication of the International Society for Infectious Diseases*, 128(), #Pages#

93. ABS Saidenberg, LS Franco, JN Reple, YMG Hounmanou, MRT Casas, B Cardoso, F Esposito, N Lincopan, A Dalsgaard, M Stegger, T Knöbl (2023). Salmonella Heidelberg and Salmonella Minnesota in Brazilian broilers: Genomic characterization of third-generation cephalosporin and fluoroquinolone-resistant strains. *Environmental microbiology reports*, 15(2), #Pages#

94. Shimizu, Takae, Kido, Nobuhide, Miyashita, Naoki, Tanaka, Sohei, Omiya, Tomoko, Morikaku, Kouki, Kawahara, Minori, Harada, Kazuki (2022). Antimicrobial resistance in Escherichia coli isolates from Japanese raccoon dogs (Nyctereutes viverrinus) in Kanagawa Prefecture, Japan: Emergence of extended-spectrum cephalosporin-resistant human-related clones. *Journal of medical microbiology*, 71(12), #Pages#

95. D Fuentes-Castillo, D Castro-Tardón, F Esposito, I Neves, L Rodrigues, H Fontana, B Fuga, JL Catão-Dias, N Lincopan (2023). Genomic evidences of gulls as reservoirs of critical priority CTX-M-producing Escherichia coli in Corcovado Gulf, Patagonia. *The Science of the total environment*, 874(), #Pages#

96. B Davido, AC Crémieux, I Vaugier, P De Truchis, K Hamami, F Laurent, A Saleh-Mghir (2023). Efficacy of ceftazidime/avibactam in various combinations for the treatment of experimental osteomyelitis in rabbits caused by OXA-48-/ESBL-producing Escherichia coli. *The Journal of antimicrobial chemotherapy*, 78(5), #Pages#

97. M Li, H Zhang, W Zhang, Y Cao, B Sun, Q Jiang, Y Zhang, H Liu, W Guo, C Chang, N Zhou, C Lv, C Guo, X Guo, J Shang, S Huang, Y Zhu (2023). One global disseminated 193 kb high-risk hybrid plasmid harboring tet(X4), mcr or bla threatening public health. *The Science of the total environment*, 876(), #Pages#

98. Paredes, Rocio, Damme, Monica, Mantilla, Jazmin, Castellanos, Luis Ricardo, Clavijo, Viviana, Celis, Yamile, Mehta, Kedar, Kumar, Ajay, Patino, Ana, Jeyashree, Kathiresan (2023). Prevalence and antimicrobial resistance of Escherichia coli and Salmonella spp. in animal feed in Colombia. *Revista panamericana de salud publica = Pan American journal of public health*, 47(#issue#), e57

99. García, A., & Fox, J. G. (2021). A one health perspective for defining and deciphering Escherichia coli pathogenic potential in multiple hosts. *Comparative Medicine*, *71*(1), 3-45

100. Cabassi, C. S., Bertocchi, M., Spadini, C., Denti, L., Flisi, S., Schiano, E., Cavirani, S., Parmigiani, E., Taddei, S. (2021). Antimicrobial activity of a standardized medical honey on bacterial isolates from infected skin lesions of non-traditional companion animals. *Veterinaria Italiana*, 57(2), 119

101. Murphy, C. P., Reid-Smith, R. J., Boerlin, P., Weese, J. S., Prescott, J. F., Janecko, N., Hassard, L., McEwen, S. A. (2010). Escherichia coli and selected veterinary and zoonotic pathogens isolated from environmental sites in companion animal veterinary hospitals in southern Ontario. *Canadian Veterinary Journal*, 51(9), 963

102. Boag, A. K., Howie, K. J. (2012). Minimizing nosocomial infection. *Advanced monitoring and procedures for small animal emergency and critical care*, #volume#(#issue#), 695

103. Varriale, L., Dipineto, L., Russo, T. P., Borrelli, L., Romano, V., D'Orazio, S., Pace, A., Menna, L. F., Fioretti, A., Santaniello, A. (2020). Antimicrobial resistance of Escherichia

coli and Pseudomonas aeruginosa from companion birds. *Antibiotics*, 9(11), #Pages#

104. Okunlade, A. O., Esan, O. O., Ogunleye, A. O. (2021). Multidrug resistant E. coli recovered from household reared female budgerigar pet bird (Melopsittacus undulatus) in Ibadan, Oyo state Nigeria: a case report. *Bulletin of the National Research Centre*, 45(155), (17

105. Harrison, L., Tyson, G. H., Strain, E., Lindsey, R. L., Strockbine, N., Ceric, O., Fortenberry, G. Z., Harris, B., Shaw, S., Tillman, G., Zhao ShaoHua, Dessai, U. (2022). Use of large-scale genomics to identify the role of animals and foods as potential sources of extraintestinal pathogenic Escherichia coli that cause human illness. *Foods*, 11(13), #Pages#

106. Hoang Le Huy, Koizumi, N., Trang Thi Hong Ung, Thanh Thi Le, Hang Le Khanh Nguyen, Phuong Vu Mai Hoang, Cam Nhat Nguyen, Tuan Minh Khong, Hasebe, F., Haga, T., Mai Thi Quynh Le, Hirayama, K., Miura, K. (2020). Antibiotic-resistant Escherichia coli isolated from urban rodents in Hanoi, Vietnam. *Journal of Veterinary Medical Science*, 82(5), 653

107. Abraham, S., O'Dea, M., Page, S. W., Trott, D. J. (2017). Current and future antimicrobial resistance issues for the Australian pig industry. *Animal Production Science*, 57(12), 2398

108. Cole, S. D., Healy, I., Dietrich, J. M., Redding, L. E. (2022). Evaluation of canine raw food products for the presence of extended-spectrum beta-lactamase- and carbapenemase-producing bacteria of the order enterobacterales. *American Journal of Veterinary Research*, 83(9), #Pages#

109. Davido, B., Crémieux, A. C., Vaugier, I., Truchis, P. de, Hamami, K., Laurent, F., Saleh-Mghir, A. (2023). Efficacy of ceftazidime/avibactam in various combinations for the treatment of experimental osteomyelitis in rabbits caused by OXA-48-/ESBL-producing Escherichia coli. *Journal of Antimicrobial Chemotherapy*, 78(5), 1211

110. Veldman, K. T., Mevius, D. J., Wit, B., Pelt, W. van, Heederik, D. (2018). MARAN 2018. Monitoring of Antimicrobial Resistance and Antibiotic Usage in Animals in the Netherlands in 2017. *#journal#*, #volume#(#issue#), 78

111. Mughini-Gras, L., Dorado-García, A., Duijkeren, E. van, Bunt, G. van den, Dierikx, C. M., Bonten, M. J. M., Bootsma, M. C. J., Schmitt, H., Hald, T., Evers, E. G., Koeijer, A. de, Pelt, W. van, Franz, E., Mevius, D. J., Heederik, D. J. J. (2019). Attributable sources of community-acquired carriage of Escherichia coli containing β-lactam antibiotic resistance genes: a population-based modelling study. *Lancet Planetary Health*, 3(8), e357

112. Fernández, J., Guerra, B., Rodicio, M. R. (2018). Resistance to carbapenems in non-typhoidal Salmonella enterica serovars from humans, animals and food. *Veterinary Sciences*, 5(2), 40

113. Parker, D., Sniatynski, M. K., Mandrusiak, D., Rubin, J. E. (2016). Extended-spectrum β-lactamase producing Escherichia coli isolated from wild birds in Saskatoon, Canada. *Letters in Applied Microbiology*, 63(1), 11

114. Ramos, C. P., Santana, J. A., Coura, F. M., Xavier, R. G. C., Leal, C. A. G., Junior, C. A. O., Heinemann, M. B., Lage, A. P., Lobato, F. C. F., Silva, R. O. S. (2019). Identification and characterization of Escherichia coli, Salmonella spp., Clostridium perfringens, and C. difficile isolates from reptiles in Brazil. *BioMed Research International*, 2019(9530732), #Pages#

115. Zhang Pei, Wang Juan, Wang XingLong, Bai Xue, Ma JianGang, Dang RuYi, Xiong YiFei, Fanning, S., Bai Li, Yang ZengQi (2019). Characterization of five Escherichia coli isolates co-expressing ESBL and MCR-1 resistance mechanisms from different origins in China. *Frontiers in Microbiology*, 10(August), 1994

116. Veldman, K. T., Mevius, D. J., Wit, I. B., Pelt, W. van, Heederik, D. (2017). MARAN 2017. Monitoring of antimicrobial resistance and antibiotic usage in animals in The Netherlands in 2016. *MARAN 2017. Monitoring of Antimicrobial Resistance and Antibiotic Usage in Animals in the Netherlands in 2016*, #volume#(#issue#), 80

117. Nesse, L. L., Osland, A. M., Vestby, L. K. (2023). The role of biofilms in the pathogenesis of animal bacterial infections. *Microorganisms*, 11(3), #Pages#

118. Rogers, B. A., Sidjabat, H. E., Paterson, D. L. (2011). Escherichia coli O25b-ST131: a pandemic, multiresistant, community-associated strain. *Journal of Antimicrobial Chemotherapy*, 66(1), 1

119. Varriale, L., Russo, T. P., Pace, A., Mediatore, S., Borrelli, L., Santaniello, A., Menna, L. F., Fioretti, A., Dipineto, L. (2019). Microbiological survey of sugar gliders (Petaurus breviceps) kept as pets in Italy. *Letters in Applied Microbiology*, 69(6), 399

120. Lloyd, D. H. (2011). Recognising and controlling risk factors for antimicrobial resistance. *European Journal of Companion Animal Practice*, 21(1), 98

121. Ooi, N., Lee, V. E., Chalam-Judge, N., Newman, R., Wilkinson, A. J., Cooper, I. R., Orr, D., Lee, S., Savage, V. J. (2020). Restoring carbapenem efficacy: a novel carbapenem companion targeting metallo-β-lactamases in carbapenem-resistant enterobacterales. *Journal of Antimicrobial Chemotherapy*, 76(2), 460

122. Marchetti, M. L., Mestorino, N. (2013). Therapeutic alternatives against multidrug resistance by efflux pump. *Analecta Veterinaria*, 33(1), 22

123. Amit Vikram, Schmidt, J. W. (2018). Functional blaKPC-2 sequences are present in U.S. beef cattle feces regardless of antibiotic use. *Foodborne Pathogens and Disease*, 15(7), 444

124. Zare, P., Ghorbani-Choboghlo, H. (2015). Isolation and characterization of multidrug-resistant Gram-negative bacteria found in free-ranging long-eared hedgehogs (Erinaceus concolor) from Tabriz, Iran. *Journal of Exotic Pet Medicine*, 24(2), 235

125. Schink, A. K., Kadlec, K., Schwarz, S. (2012). Detection of qnr genes among Escherichia coli isolates of animal origin and complete sequence of the conjugative qnrB19-carrying plasmid pQNR2078. *Journal of Antimicrobial Chemotherapy*, 67(5), 1099

126. Rayamahji, N., Yoo HanSang, Kimura, J., Yanai, T. (2014). Characterization of cephalosporin resistance in Enterobacteriae isolated from farm animals. *Proceedings of First International Conference and Annual Meeting of Myanmar Veterinary Association, 2-3 February 2014, Yangon, Myanmar. Livestock-Human-Environment Interface Challenge to Animals' Health and Livestock Production*, #volume#(#issue#), 102

127. Li XianZhi, Mehrotra, M., Ghimire, S., Adewoye, L. (2007). β-Lactam resistance and β-lactamases in bacteria of animal origin. *Veterinary Microbiology*, 121(3/4), 197

128. Dolejska, M., Villa, L., Hasman, H., Hansen, L., Carattoli, A. (2013). Characterization of IncN plasmids carrying blaCTX-M-1 and qnr genes in Escherichia coli and Salmonella from animals, the environment and humans. *Journal of Antimicrobial Chemotherapy*, 68(2), 333

129. Gama, Gil Sander Próspero, Pimenta, Alexandre Santos, Feijó, Francisco Marlon Carneiro, Santos, Caio Sérgio, Fernandes, Bruno Caio Chaves, de Oliveira, Moacir Franco, de Souza, Elias Costa, Monteiro, Thays V. C., Fasciotti, Maíra, de Azevedo, Tatiane Kelly Barbosa, de Melo, Rafael Rodolfo, Júnior, Ananias Francisco Dias (2023). Antimicrobial activity and chemical profile of wood vinegar from eucalyptus (Eucalyptus urophylla x Eucalyptus grandis - clone I144) and bamboo (Bambusa vulgaris) *World Journal of Microbiology and Biotechnology*, 39(7), 186

130. Lemlem, Mulu, Erkihun Aklilu, Maizan Mohammed, Fadhilah Kamaruzzaman, Zunita Zakaria, Harun, Azian, Devan, Susmita Seenu (2023). Molecular detection and antimicrobial resistance profiles of Extended-Spectrum Beta-Lactamase (ESBL) producing Escherichia coli in broiler chicken farms in Malaysia *PLoS One*, 18(5), #Pages#

131. Igbinosa, Etinosa O, Beshiru, Abeni, Igbinosa, Isoken H, Gyu-Sung Cho, Charles M. A. P. Franz (2023). Multidrug-resistant extended spectrum β-lactamase (ESBL)-producing Escherichia coli from farm produce and agricultural environments in Edo State, Nigeria *PLoS One*, 18(3), #Pages#

132. Campos-Madueno, Edgar I, Moradi, Melika, Eddoubaji, Yasmine, Shahi, Fatemeh, Moradi, Sina, Bernasconi, Odette J, Moser, Aline I, Endimiani, Andrea (2023). Intestinal colonization with multidrug-resistant Enterobacterales: screening, epidemiology, clinical impact, and strategies to decolonize carriers. *European journal of clinical microbiology & infectious diseases : official publication of the European Society of Clinical Microbiology*, 42(3), 229

133. Bisi-Johnson, Mary A., Adedeji, Atilade A., Sulaiman, Adebayo A., Adefisoye, Martins A., Okoh, Anthony I. (2023). Isolation and genotypic characterization of extended-spectrum beta-lactamase-producing Escherichia coli O157:H7 and Aeromonas hydrophila from selected freshwater sources in Southwest Nigeria *Scientific Reports (Nature Publisher Group)*, 13(1), 10746

134. Endres, Creciana M, Moreira, Eliana, de Freitas, Andressa B, Dal Castel, Andréia P, Graciano, Fábio, Mann, Michele B, Frazzon, Ana Paula G, Mayer, Fabiana Q, Frazzon, Jeverson (2023). Evaluation of Enterotoxins and Antimicrobial Resistance in Microorganisms Isolated from Raw Sheep Milk and Cheese: Ensuring the Microbiological Safety of These Products in Southern Brazil *Microorganisms*, 11(6), 1618

135. Musa, Laura, Stefanetti, Valentina, Patrizia Casagrande Proietti, Grilli, Guido, Gobbi, Marco, Toppi, Valeria, Brustenga, Leonardo, Magistrali, Chiara Francesca, Franciosini, Maria Pia (2023). Antimicrobial Susceptibility of Commensal E. coli Isolated from Wild Birds in Umbria (Central Italy) *Animals*, 13(11), 1776

136. Soares, Rúben, Miranda, Carla, Cunha, Sandra, Ferreira, Luís, Martins, Ângela, Igrejas, Gilberto, Poeta, Patrícia (2023). Antibiotic Resistance of Enterococcus Species in Ornamental Animal Feed *Animals*, 13(11), 1761

137. Fernández, Mercedes, Biel Garcias, Duran, Inma, Molina-López, Rafael A, Darwich, Laila (2023). Current Situation of Bacterial Infections and Antimicrobial Resistance Profiles in Pet Rabbits in Spain *Veterinary Sciences*, 10(5), 352

138. Jiang, Xueqi, Miao, Beibei, Zhao, Xiaofei, Bai, Xuemei, Yuan, Min, Chen, Xia, Gong, Xinyi, Liu, Zeliang, Li, Jie, Meng, Shuang, Han, Xiao, Li, Juan (2023). Unveiling the Emergence and Genetic Diversity of OXA-48-like Carbapenemase Variants in Shewanella xiamenensis *Microorganisms*, 11(5), 1325

139. Gharbi, Manel, Béjaoui, Awatef, Safa Hamrouni, Arfaoui, Amel, Maaroufi, Abderrazak (2023). Persistence of Campylobacter spp. in Poultry Flocks after Disinfection, Virulence, and Antimicrobial Resistance Traits of Recovered Isolates *Antibiotics*, 12(5), 890

140. Nada, Hanady G, Amera Saeed El-Tahan, El-Didamony, Gamal, Askora, Ahmed (2023). Detection of multidrug-resistant Shiga toxin-producing Escherichia coli in some food products and cattle faeces in Al-Sharkia, Egypt: one health menace *BMC Microbiology*, 23(#issue#), 1

141. Benlabidi, Saloua, Raddaoui, Anis, Lengliz, Sana, Cheriet, Sarah, Hynds, Paul, Achour, Wafa, Ghrairi, Taoufik, Mohamed Salah Abbassi (2023). Occurrence of High-Risk Clonal Lineages ST58, ST69, ST224, and ST410 among Extended-Spectrum β-Lactamase-Producing Escherichia coli Isolated from Healthy Free-Range Chickens (Gallus gallus domesticus) in a Rural Region in Tunisia *Genes*, 14(4), 875

142. Duman, Muhammed, Elena García Valdés, Ay, Hilal, Altun, Soner, Saticioglu, Izzet Burcin (2023). Description of a Novel Fish Pathogen, Plesiomonas shigelloides subsp. oncorhynchi, Isolated from Rainbow Trout (Oncorhynchus mykiss): First Genome Analysis and Comparative Genomics *Fishes*, 8(4), 179

143. Barros, Maria Margarida, Castro, Joana, Araújo, Daniela, Campos, Ana Maria, Oliveira, Ricardo, Silva, Sónia, Outor-Monteiro, Divanildo, Almeida, Carina (2023). Swine Colibacillosis: Global Epidemiologic and Antimicrobial Scenario *Antibiotics*, 12(4), 682

144. Guarneri, Flavia, Bertasio, Cristina, Romeo, Claudia, Formenti, Nicoletta, Scali, Federico, Parisio, Giovanni, Canziani, Sabrina, Boifava, Chiara, Guadagno, Federica, Boniotti, Maria Beatrice, Giovanni Loris Alborali (2023). First Detection of mcr-9 in a Multidrug-Resistant Escherichia coli of Animal Origin in Italy Is Not Related to Colistin Usage on a Pig Farm *Antibiotics*, 12(4), 689

145. Ribeiro, Jessica, Silva, Vanessa, Monteiro, Andreia, Vieira-Pinto, Madalena, Igrejas, Gilberto, Reis, Filipa S, Barros, Lillian, Poeta, Patrícia (2023). Antibiotic Resistance among Gastrointestinal Bacteria in Broilers: A Review Focused on Enterococcus spp. and Escherichia coli *Animals*, 13(8), 1362

146. Wanja, Daniel W, Mbuthia, Paul G, Bebora, Lilly C, Aboge, Gabriel O, Ogoti, Brian (2023). Antimicrobial Usage, Susceptibility Profiles, and Resistance Genes in Campylobacter Isolated from Cattle, Chicken, and Water Samples in Kajiado County, Kenya *International Journal of Microbiology*, 2023(#issue#), #Pages#

147. Iulia, Adelina Bunduruș, Igori Balta, Ștef, Lavinia, Ahmadi, Mirela, Peț, Ioan, McCleery, David, Corcionivoschi, Nicolae (2023). Overview of Virulence and Antibiotic Resistance in Campylobacter spp. Livestock Isolates *Antibiotics*, 12(2), 402

148. Pottier, Marine, Castagnet, Sophie, Gravey, François, Leduc, Guillaume, Sévin, Corinne, Petry, Sandrine, Giard, Jean-Christophe, Simon Le Hello, Albertine Léon (2023). Antimicrobial Resistance and Genetic Diversity of Pseudomonas aeruginosa Strains Isolated from Equine and Other Veterinary Samples *Pathogens*, 12(1), 64

149. Hou, Bo, Wang, Chen-Yan, Li, Shao-Wen, Zhou, Lun-Jiang, Che, Yong-Liang, Chen, Qiu-Yong (2022). Effects of Toxin-Antitoxin System HicAB on Biofilm Formation by Extraintestinal Pathogenic E. coli. *Current microbiology*, 80(1), 50

150. Perestrelo, S., Carreira, G. C., Valentin, L., Fischer, J., Pfeifer, Y., Werner, G., Schmiedel, J., Falgenhauer, L., Imirzalioglu, C., Chakraborty, T., & Käsbohrer, A. (2022). Comparison of approaches for source attribution of ESBL-producing Escherichia coli in Germany. *PLOS ONE, 17*(7), e#Pages#

151. Da Silva, Wanderson Marques, Larzabal, Mariano, Aburjaile, Flavia Figueira, Riviere, Nahuel, Martorelli, Luisina, Bono, James, Amadio, Ariel, Cataldi, Angel (2022). Whole-genome sequencing analysis of Shiga toxin-producing Escherichia coli O22:H8 isolated from

cattle prediction pathogenesis and colonization factors and position in STEC universe phylogeny. *Journal of microbiology (Seoul, Korea)*, 60(7), 689

152. Maniam, Lalitha, Vellasamy, Kumutha Malar, Jindal, Hassan Mahmood, Narayanan, Vallikannu, Danaee, Mahmoud, Vadivelu, Jamuna, Pallath, Vinod (2022). Demonstrating the utility of Escherichia coli asymptomatic bacteriuria isolates' virulence profile towards diagnosis and management—A preliminary analysis *PLoS One*, 17(5), #Pages#

153. Peng, Z., Maciel-Guerra, A., Baker, M., Zhang, X., Hu, Y., Wang, W., ... & Dottorini, T. (2022). Whole-genome sequencing and gene sharing network analysis powered by machine learning identifies antibiotic resistance sharing between animals, humans and environment in livestock farming. *PLoS computational biology, 18*(3), e1010018

154. Kocsis, Béla, Gulyás, Dániel, Szabó, Dóra (2022). Emergence and Dissemination of Extraintestinal Pathogenic High-Risk International Clones of Escherichia coli *Life*, 12(12), 2077

155. Belmahdi, Mohamed, Nadia Safia Chenouf, Abdelkrim Ait Belkacem, Martinez-Alvarez, Sandra, Pino-Hurtado, Mario Sergio, Benkhechiba, Zahra, Lahrech, Samiha, Hakem, Ahcène, Torres, Carmen (2022). Extended Spectrum β-Lactamase-Producing Escherichia coli from Poultry and Wild Birds (Sparrow) in Djelfa (Algeria), with Frequent Detection of CTX-M-14 in Sparrow *Antibiotics*, 11(12), 1814

156. Zamudio, Roxana, Boerlin, Patrick, Beyrouthy, Racha, Madec, Jean-Yves, Schwarz, Stefan, Mulvey, Michael R., Zhanel, George G., Cormier, Ashley, Chalmers, Gabhan, Bonnet, Richard, Haenni, Marisa, Eichhorn, Inga, Kaspar, Heike, Garcia-Fierro, Raquel, Wood, James L. N., Mather, Alison E. (2022). Dynamics of extended-spectrum cephalosporin resistance genes in Escherichia coli from Europe and North America *Nature Communications*, 13(1), #Pages#

157. Sri, Anna, Bailey, Kirsten E, Gilkerson, James R, Browning, Glenn F, Hardefeldt, Laura Y (2022). Attitudes towards Use of High-Importance Antimicrobials—A Cross-Sectional Study of Australian Veterinarians *Antibiotics*, 11(11), 1589

158. Leresche Even Doneilly Oyaba Yinda, Onanga, Richard, Pierre Philippe Mbehang Nguema, Etienne François Akomo-Okoue, Akoue, Gontran Nsi, Neil Michel Longo Pendy, Ekore, Desire Otsaghe, Lendamba, Roméo Wenceslas, Mabika-Mabika, Arsène, Jean Constant Obague Mbeang, Poungou, Natacha, Ibrahim, Mavoungou, Jacques François, Godreuil, Sylvain (2022). Phylogenetic Groups, Pathotypes and Antimicrobial Resistance of Escherichia coli Isolated from Western Lowland Gorilla Faeces (Gorilla gorilla gorilla) of Moukalaba-Doudou National Park (MDNP) *Pathogens*, 11(10), 1082

159. Metreveli, Maia, Bulia, Salome, Tevzadze, Liana, Tsanava, Shota, Zarske, Michael, Juan Cruz Goenaga, Preuß, Sandra, Giorgi Lomidze, Koulouris, Stylianos, Imnadze, Paata, Stingl, Kerstin (2022). Comparison of Antimicrobial Susceptibility Profiles of Thermotolerant Campylobacter spp. Isolated from Human and Poultry Samples in Georgia (Caucasus)

Antibiotics, 11(10), 1419

160. Casalino, Gaia, Francesco D'Amico, Dinardo, Francesca Rita, Bozzo, Giancarlo, Napoletano, Valeria, Camarda, Antonio, Bove, Antonella, Lombardi, Roberto, Francesco Paolo D'Onghia, Circella, Elena (2022). Prevalence and Antimicrobial Resistance of Campylobacter jejuni and Campylobacter coli in Wild Birds from a Wildlife Rescue Centre *Animals*, 12(20), 2889

161. Dewi, Rita Rosmala, Latiffah Hassan, Hassan Mohammad Daud, Matori, Mohd Fuad, Zunita Zakaria, Ahmad, Nur Indah, Aziz, Saleha A, Saleh Mohammed Jajere (2022). On-Farm Practices Associated with Multi-Drug-Resistant Escherichia coli and Vibrio parahaemolyticus Derived from Cultured Fish *Microorganisms*, 10(8), 1520

162. Madubuike Umunna Anyanwu, Jaja, Ishmael Festus, Obichukwu Chisom Nwobi, Mgbeahuruike, Anthony Christian, Chinaza, Nnenna Ikpendu, Okafor, Nnenna Audrey, Oguttu, James Wabwire (2022). Epidemiology and Traits of Mobile Colistin Resistance (mcr) Gene-Bearing Organisms from Horses *Microorganisms*, 10(8), 1499

163. McDougall, Fiona, Boardman, Wayne, Power, Michelle (2022). High Prevalence of Beta-Lactam-Resistant Escherichia coli in South Australian Grey-Headed Flying Fox Pups (Pteropus poliocephalus) *Microorganisms*, 10(8), 1589

164. Chen, Qiyan, Zou, Zhiyu, Cai, Chang, Li, Hui, Wang, Yang, Lei, Lei, Shao, Bing (2022). Characterization of blaNDM-5-and blaCTX-M-199-Producing ST167 Escherichia coli Isolated from Shared Bikes *Antibiotics*, 11(8), 1030

165. Moawad, Amira A, Hotzel, Helmut, Hafez, Hafez M, Ramadan, Hazem, Herbert, Tomaso, Braun, Sascha D, Ehricht, Ralf, Diezel, Celia, Dominik, Gary, Engelmann, Ines, Zakaria, Islam M, Reda, Reem M, Eid, Samah, Shahien, Momtaz A, Neubauer, Heinrich, Monecke, Stefan (2022). Occurrence, Phenotypic and Molecular Characteristics of Extended-Spectrum Beta-Lactamase-Producing Escherichia coli in Healthy Turkeys in Northern Egypt *Antibiotics*, 11(8), 1075

166. Yamê Miniero Davies, Vieira Cunha, Marcos Paulo, Dropa, Milena, Lincopan, Nilton, Vasco Túlio Moura Gomes, Luisa Zanoli Moreno, Zanoli Sato, Maria Inês, Moreno, Andrea Micke, Knöbl, Terezinha (2022). Pandemic Clones of CTX-M-15 Producing Klebsiella pneumoniae ST15, ST147, and ST307 in Companion Parrots *Microorganisms*, 10(7), 1412

167. Galgano, Michela, Capozza, Paolo, Pellegrini, Francesco, Cordisco, Marco, Sposato, Alessio, Sblano, Sabina, Camero, Michele, Lanave, Gianvito, Fracchiolla, Giuseppe, Corrente, Marialaura, Cirone, Francesco, Trotta, Adriana, Tempesta, Maria, Buonavoglia, Domenico, Pratelli, Annamaria (2022). Antimicrobial Activity of Essential Oils Evaluated In Vitro against Escherichia coli and Staphylococcus aureus *Antibiotics*, 11(7), 979

168. Méndez-Moreno, Evelyn, Caporal-Hernandez, Liliana, Mendez-Pfeiffer, Pablo A, Enciso-Martinez, Yessica, Rafael De la Rosa López, Valencia, Dora, Arenas-Hernández, Margarita M P, Ballesteros-Monrreal, Manuel G, Barrios-Villa, Edwin (2022). Characterization of Diarreaghenic Escherichia coli Strains Isolated from Healthy Donors, including a Triple Hybrid Strain *Antibiotics*, 11(7), 833

169. Bastidas-Caldes, Carlos, de Waard, Jacobus H, Salgado, María Soledad, Villacís, María José, Coral-Almeida, Marco, Yamamoto, Yoshimasa, Calvopiña, Manuel (2022). Worldwide Prevalence of mcr-mediated Colistin-Resistance Escherichia coli in Isolates of Clinical Samples, Healthy Humans, and Livestock—A Systematic Review and Meta-Analysis *Pathogens*, 11(6), 659

170. Loucif, Lotfi, Chelaghma, Widad, Bendjama, Esma, Cherak, Zineb, Khellaf, Meriem, Khemri, Asma, Rolain, Jean-Marc (2022). Detection of blaOXA-48 and mcr-1 Genes in Escherichia coli Isolates from Pigeon (Columba livia) in Algeria *Microorganisms*, 10(5), 975

171. Su, Xiaoyan, Xia, Yan, Li, Yunli, Zhang, Dongsheng, Li, Lin, Geng, Yi, Su, Fei, Chanjuan Yue, Hou, Rong, Liu, Songrui (2022). Identification of extended-spectrum beta-lactamase (CTX-M)-producing Klebsiella pneumoniae belonging to ST37, ST290, and ST2640 in captive giant pandas *BMC Veterinary Research*, 18(#issue#), 1

172. Florea Dumitrascu, Ana-Maria Udrea, Caira, Mino R, Nuta, Diana Camelia, Limban, Carmen, Chifiriuc, Mariana Carmen, Popa, Marcela, Bleotu, Coralia, Hanganu, Anamaria, Dumitrescu, Denisa, Speranta Avram (2022). In Silico and Experimental Investigation of the Biological Potential of Some Recently Developed Carprofen Derivatives *Molecules*, 27(9), 2722

173. Luciana Sampaio Lima, Aldo Aparecido Proietti-Junior, Yan Corrêa Rodrigues, Marcelo Cleyton da Silva Vieira, Luana Nepomuceno Gondim Costa Lima, Cintya de Oliveira Souza, Verônica Dias Gonçalves, de Oliveira Lima, Marcelo, Dália dos Prazeres Rodrigues, Karla Valéria Batista Lima (2022). High Genetic Diversity and Antimicrobial Resistance in Escherichia coli Highlight Arapaima gigas (Pisces: Arapaimidae) as a Reservoir of Quinolone-Resistant Strains in Brazilian Amazon Rivers *Microorganisms*, 10(4), 808

174. Martínez-Álvarez, Sandra, Sanz, Susana, Olarte, Carmen, Hidalgo-Sanz, Raquel, Carvalho, Isabel, Fernández-Fernández, Rosa, Campaña-Burguet, Allelen, Latorre-Fernández, Javier, Zarazaga, Myriam, Torres, Carmen (2022). Antimicrobial Resistance in Escherichia coli from the Broiler Farm Environment, with Detection of SHV-12-Producing Isolates *Antibiotics*, 11(4), 444

175. Ghenea, Alice Elena, Ovidiu Mircea Zlatian, Cristea, Oana Mariana, Ungureanu, Anca, Mititelu, Radu Razvan, Balasoiu, Andrei Theodor, Vasile, Corina Maria, Alex-Ioan Salan, Iliuta, Daniel, Popescu, Mihaela, Anca-Loredana Udriștoiu, Balasoiu, Maria (2022). TEM,CTX-M,SHV Genes in ESBL-Producing Escherichia coli and Klebsiella pneumoniae Isolated from Clinical Samples in a County Clinical Emergency Hospital Romania-

Predominance of CTX-M-15 *Antibiotics*, 11(4), 503

176. Alzahrani, Othman M, Mahmoud Fayez, Alswat, Amal S, Alkafafy, Mohamed, Mahmoud, Samy F, Al-Marri, Theeb, Almuslem, Ahmed, Ashfaq, Hassan, Yusuf, Shaymaa (2022). Antimicrobial Resistance, Biofilm Formation, and Virulence Genes in Enterococcus Species from Small Backyard Chicken Flocks *Antibiotics*, 11(3), 380

177. Oluwawemimo Adebowale, Makanjuola, Motunrayo, Bankole, Noah, Olasoju, Mary, Alamu, Aderonke, Eniola Kperegbeyi, Oladejo, Oladotun, Fasanmi, Olubunmi, Adeyemo, Olanike, Fasina, Folorunso O (2022). Multi-Drug Resistant Escherichia coli, Biosecurity and Anti-Microbial Use in Live Bird Markets, Abeokuta, Nigeria *Antibiotics*, 11(2), 253

178. Hang Thi Nguyen, Venter, Henrietta, Woolford, Lucy, Young, Kelly, McCluskey, Adam, Garg, Sanjay, Page, Stephen W, Trott, Darren J, Ogunniyi, Abiodun David (2022). Impact of a Novel Anticoccidial Analogue on Systemic Staphylococcus aureus Infection in a Bioluminescent Mouse Model *Antibiotics*, 11(1), 65

179. Mitchell, Joseph, Purohit, Manju, Jewell, Chris P, Read, Jonathan M, Marrone, Gaetano, Diwan, Vishal, Stålsby Lundborg, Cecilia (2021). Trends, relationships and case attribution of antibiotic resistance between children and environmental sources in rural India. *Scientific reports*, 11(1), 22599

180. Ojha Purusottam, Kar Narayani Prasad, Nayak Shreenath, Patra, Ashok Kumar, Sahoo, Khirod Kumar (2021). Isolation of a broad spectrum antimicrobial producing thermophilic Bacillus and characterization of its antimicrobial protein *Archives of Microbiology*, 203(5), 2059

181. Takako, Taniguchi, Elpita, Tarigan, Hiroyuki, Sato, Chiho, Kaneko, Naoaki, Misawa (2021). Prevalence of Campylobacter spp. in Raccoon Dogs and Badgers in Miyazaki Prefecture, Japan. *EcoHealth*, 18(2), 241

182. Azimi Taher, Azimi Leila, Fallah Fatemeh, Pourmand, Mohammad Reza, Ostadtaghizadeh Abbas, Abai, Mohammad Reza, Rahimi, Foroushani Abbas (2021). Detection and characterization of Enterobacteriaceae family members carried by commensal Rattus norvegicus from Tehran, Iran *Archives of Microbiology*, 203(4), 1321

183. Sitthichai Kanokudom, Assawakongkarat, Thachaporn, Akeda, Yukihiro, Ratthawongjirakul, Panan, Chuanchuen, Rungtip, Chaichanawongsaroj, Nuntaree (2021). Rapid detection of extended spectrum β-lactamase producing Escherichia coli isolated from fresh pork meat and pig cecum samples using multiplex recombinase polymerase amplification and lateral flow strip analysis *PLoS One*, 16(3), #Pages#

184. Behruznia, Mahboobeh (2021). Genetic Structure of Escherichia Coli Strains Isolated from Septic Tanks in the Canberra Region, Australia *#journal#*, #volume#(#issue#), 171

185. Nigatu Aklilu Atlaw, Keelara, Shivaramu, Correa, Maria, Foster, Derek, Gebreyes, Wondwossen, Aidara-Kane, Awa, Harden, Lyndy, Thakur, Siddhartha, Fedorka Cray, Paula J (2021). Identification of CTX-M Type ESBL E. coli from Sheep and Their Abattoir Environment Using Whole-Genome Sequencing *Pathogens*, 10(11), 1480

186. Ballesteros-Monrreal, Manuel G, Arenas-Hernández, Margarita M P, Barrios-Villa, Edwin, Juarez, Josue, Álvarez-Ainza, Maritza Lizeth, Taboada, Pablo, De la Rosa-López, Rafael, Bolado-Martínez, Enrique, Valencia, Dora (2021). Bacterial Morphotypes as Important Trait for Uropathogenic E. coli Diagnostic; a Virulence-Phenotype-Phylogeny Study *Microorganisms*, 9(11), 2381

187. Rochegüe, Tony, Haenni, Marisa, Mondot, Stanislas, Astruc, Chloé, Cazeau, Géraldine, Ferry, Tristan, Madec, Jean-Yves, Lupo, Agnese (2021). Impact of Antibiotic Therapies on Resistance Genes Dynamic and Composition of the Animal Gut Microbiota *Animals*, 11(11), 3280

188. Clemente, Lurdes, Leão, Célia, Moura, Laura, Albuquerque, Teresa, Amaro, Ana (2021). Prevalence and Characterization of ESBL/AmpC Producing Escherichia coli from Fresh Meat in Portugal *Antibiotics*, 10(11), 1333

189. Kocúreková, Tímea, Karahutová, Lívia, Bujňáková, Dobroslava (2021). Antimicrobial Susceptibility and Detection of Virulence-Associated Genes in Escherichia coli Strains Isolated from Commercial Broilers *Antibiotics*, 10(11), 1303

190. Mišić, Dušan, Kiskaroly, Ferenc, Szostak, Michael P, Cabal, Adriana, Ruppitsch, Werner, Bernreiter-Hofer, Tanja, Milovanovic, Viktoria, Feßler, Andrea T, Allerberger, Franz, Spergser, Joachim, Müller, Elke, Schwarz, Stefan, Braun, Sascha D, Monecke, Stefan, Ehricht, Ralf, Korus, Maciej, Benković, Damir, Korzeniowska, Malgorzata, Loncaric, Igor (2021). The First Report of mcr-1-Carrying Escherichia coli Originating from Animals in Serbia *Antibiotics*, 10(9), 1063

191. Kim, Hyunsoo, Young Ah Kim, Seo, Young Hee, Lee, Hyukmin, Lee, Kyungwon (2021). Prevalence and Molecular Epidemiology of Extended-Spectrum-β-Lactamase (ESBL)-Producing Escherichia coli from Multiple Sectors of Poultry Industry in Korea *Antibiotics*, 10(9), 1050

192. Leonardos, Mageiros, Méric Guillaume, Bayliss, Sion C, Pensar Johan, Pascoe, Ben, Mourkas Evangelos, Calland, Jessica K, Yahara Koji, Murray, Susan, Wilkinson, Thomas S, Williams, Lisa K, Hitchings, Matthew D, Porter, Jonathan, Kemmett Kirsty, Feil, Edward J, Jolley, Keith A, Williams, Nicola J, Corander Jukka, Sheppard, Samuel K (2021). Genome evolution and the emergence of pathogenicity in avian Escherichia coli *Nature Communications*, 12(1), #Pages#

193. Alhamami, Tamara, Piklu Roy Chowdhury, Gomes, Nancy, Carr, Mandi, Veltman, Tania, Khazandi, Manouchehr, Mollinger, Joanne, Deutscher, Ania T, Turni, Conny, Mahdi,

Layla, Venter, Henrietta, Abraham, Sam, Djordjevic, Steven P, Trott, Darren J (2021). First Emergence of Resistance to Macrolides and Tetracycline Identified in Mannheimia haemolytica and Pasteurella multocida Isolates from Beef Feedlots in Australia *Microorganisms*, 9(6), 1322

194. Hang Thi Nguyen, Morshed, Mahmud T, Vuong, Daniel, Crombie, Andrew, Lacey, Ernest, Garg, Sanjay, Pi, Hongfei, Woolford, Lucy, Venter, Henrietta, Page, Stephen W, Piggott, Andrew M, Trott, Darren J, Ogunniyi, Abiodun D (2021). Evaluation of Benzguinols as Next-Generation Antibiotics for the Treatment of Multidrug-Resistant Bacterial Infections *Antibiotics*, 10(6), 727

195. Darwich, Laila, Seminati, Chiara, López-Olvera, Jorge R, Vidal, Anna, Aguirre, Laia, Cerdá, Marina, Biel Garcias, Valldeperes, Marta, Castillo-Contreras, Raquel, Migura-Garcia, Lourdes, Conejero, Carles, Mentaberre, Gregorio (2021). Detection of Beta-Lactam-Resistant Escherichia coli and Toxigenic Clostridioides difficile Strains in Wild Boars Foraging in an Anthropization Gradient *Animals*, 11(6), 1585

196. Homeier-Bachmann, Timo, Heiden, Stefan E, Lübcke, Phillip K, Bachmann, Lisa, Bohnert, Jürgen A, Zimmermann, Dirk, Schaufler, Katharina (2021). Antibiotic-Resistant Enterobacteriaceae in Wastewater of Abattoirs *Antibiotics*, 10(5), 568

197. Baez, Michel, Espinosa, Ivette, Collaud, Alexandra, Miranda, Iliana, Damarys de las Nieves Montano, Feria, Angel L, Hernández-Fillor, Rosa Elena, Obregón, Dasiel, Pastor, Alfonso, Perreten, Vincent (2021). Genetic Features of Extended-Spectrum β-Lactamase-Producing Escherichia coli from Poultry in Mayabeque Province, Cuba *Antibiotics*, 10(2), 107

198. Haroun, Michelyne, Tratrat, Christophe, Kolokotroni, Aggeliki, Petrou, Anthi, Geronikaki, Athina, Ivanov, Marija, Kostic, Marina, Sokovic, Marina, Carazo, Alejandro, Mladěnka, Přemysl, Nagaraja Sreeharsha, Venugopala, Katharigatta N, Nair, Anroop B, Elsewedy, Heba S (2021). 5-Benzyliden-2-(5-methylthiazol-2-ylimino)thiazolidin-4-ones as Antimicrobial Agents. Design, Synthesis, Biological Evaluation and Molecular Docking Studies *Antibiotics*, 10(3), 309

199. Sekiya, Hiroshi, Okada, Maho, Tamai, Eiji, Shimamoto, Toshi, Shimamoto, Tadashi, Nariya, Hirofumi (2021). A Putative Amidase Endolysin Encoded by Clostridium perfringens St13 Exhibits Specific Lytic Activity and Synergizes with the Muramidase Endolysin Psm *Antibiotics*, 10(3), 245

200. Mundbjerg, Karin, Pedersen, Peder Elbek, Hammer, Anne Sofie (2021). The effect of antimicrobial treatment on mortality associated with urinary tract disease in mink kits (Neovison vison) *Acta Veterinaria Scandinavica*, 63(#issue#), 1

201. Stachelek, Magdalena, Zalewska, Magdalena, Kawecka-Grochocka, Ewelina, Sakowski, Tomasz, Bagnicka, Emilia (2021). Overcoming bacterial resistance to antibiotics: the urgent

need – a review *Annals of Animal Science*, 21(1), 63

202. Ben Romdhane, Racem, Merle, Roswitha (2021). The Data Behind Risk Analysis of Campylobacter Jejuni and Campylobacter Coli Infections. *Current topics in microbiology and immunology*, 431(#issue#), 25

203. Gambino, D., Vicari, D., Vitale, M., Schirò, G., Mira, F., Giglia, M. L., ... & Gargano, V. (2021). Study on bacteria isolates and antimicrobial resistance in wildlife in Sicily, southern Italy. *Microorganisms*, *9*(1), 203

204. Denamur, Erick, Clermont, Olivier, Bonacorsi, Stéphane, Gordon, David (2021). The population genetics of pathogenic Escherichia coli. *Nature reviews. Microbiology*, 19(1), 37

205. Vercelli, Cristina, Lebkowska-Wieruszewska Beata Łebkowska-Wieruszewska, Barbero, Raffaella, Lisowski, Andrzej, Re, Giovanni, Giorgi, Mario (2020). Pharmacokinetics of levofloxacin in non-lactating goats and evaluation of drug effects on resistance in coliform rectal flora *Research in Veterinary Science*, 133(#issue#), 283

206. Akua Obeng Forson, David Nana Adjei, Olu-Taiwo, Michael, Quarchie, Marjorie Ntiwaa, Asmah, Harry Richard (2020). Characterization of Campylobacter associated gastric enteritis among patients with Human Immunodeficiency Virus (HIV) in a hospital in Accra, Ghana *PLoS One*, 15(10), #Pages#

207. Algammal, Abdelazeem M, Mabrok, Mahmoud, Sivaramasamy, Elayaraja, Youssef, Fatma M, Atwa, Mona H, El-Kholy, Ali W, Hetta, Helal F, Hozzein, Wael N (2020). Emerging MDR-Pseudomonas aeruginosa in fish commonly harbor oprL and toxA virulence genes and blaTEM, blaCTX-M, and tetA antibiotic-resistance genes. *Scientific reports*, 10(1), 15961

208. Silva, Nuno, Phythian, Clare J, Currie, Carol, Tassi, Riccardo, Ballingall, Keith T, Magro, Giada, McNeilly, Tom N, Zadoks, Ruth N (2020). Antimicrobial resistance in ovine bacteria: A sheep in wolf's clothing? *PLoS One*, 15(9), #Pages#

209. Carvalho Daiane, Kunert-Filho, Hiran Castagnino, Simoni Cintia, de Moraes Lucas Brunelli, Furian Thales Quedi, Borges, Karen Apellanis, Breunig, Jônatas Grellmann, Medeiros, Leonardo Pinto, Kobayashi Renata Katsuko Takayama, de Brito Kelly Cristina Tagliari, de Brito Benito Guimarães (2020). Antimicrobial susceptibility and detection of virulence-associated genes of Escherichia coli and Salmonella spp. isolated from domestic pigeons (Columba livia) in Brazil *Folia Microbiologica*, 65(4), 735

210. cio Mandomando, Delfino Vubil, Boisen, Nadia, Ruiz, Joaquim, Nhampossa, Tacilta, Garrine, Marcelino, Massora, Sergio, Aide, Pedro, Nhacolo, Ariel, Pons, Maria J, Bassat, Quique, Vila, Jordi, Macete, bio, Scheutz, Flemming, Levine, Myron M, Ruiz-Perez, Fernando, Nataro, James P, Alonso, Pedro L (2020). Escherichia coli ST131 clones harbouring AggR and AAF/V fimbriae causing bacteremia in Mozambican children:

Emergence of new variant of fimH27 subclone *PLoS Neglected Tropical Diseases*, 14(5), #Pages#

211. Palma, Ernesto, Tilocca, Bruno, Roncada, Paola (2020). Antimicrobial Resistance in Veterinary Medicine: An Overview *International Journal of Molecular Sciences*, 21(6), 1914

212. Loo, Clarissa van Der (2020). Analysis of Borehole Water with Specific Emphasis on the Detection of Free-Living Amoebae and Amoeba-Resistant Bacteria *#journal#*, #volume#(#issue#), 242

213. Taggar, Gurleen, Rheman, Muhammad Attiq, Boerlin, Patrick, Diarra, Moussa Sory (2020). Molecular Epidemiology of Carbapenemases in Enterobacteriales from Humans, Animals, Food and the Environment *Antibiotics*, 9(10), 693

214. Ebani, V. V., & Mancianti, F. (2020). Use of essential oils in veterinary medicine to combat bacterial and fungal infections. *Veterinary sciences*, *7*(4), 193.

215. Carvalho, Isabel, Tejedor-Junco, María Teresa, González-Martín, Margarita, Corbera, Juan Alberto, Silva, Vanessa, Igrejas, Gilberto, Torres, Carmen, Poeta, Patrícia (2020). Escherichia coli Producing Extended-Spectrum β-lactamases (ESBL) from Domestic Camels in the Canary Islands: A One Health Approach *Animals*, 10(8), 1295

216. Onanga, Richard, Pierre Philippe Mbehang Nguema, Guy Roger Ndong Atome, Arsène Mabika Mabika, Ngoubangoye, Berthelemy, Wed Leslie Komba Tonda, Jean Constant Obague Mbeang, Lebibi, Jacques (2020). Prevalence of Extended-Spectrum β-Lactamases in E. coli of Rats in the Region North East of Gabon *Veterinary Medicine International*, 2020(#issue#), #Pages#

217. Meroni, Gabriele, Cardin, Elena, Rendina, Charlotte, Herrera Millar, Valentina Rafaela, Soares Filipe, Joel Fernando, Martino, Piera Anna (2020). In Vitro Efficacy of Essential Oils from Melaleuca Alternifolia and Rosmarinus Officinalis, Manuka Honey-based Gel, and Propolis as Antibacterial Agents Against Canine Staphylococcus Pseudintermedius Strains *Antibiotics*, 9(6), 344

218. Kim, Shukho, Woo, Jung Hwa, So Hyun Jun, Dong Chan Moon, Suk-Kyung Lim, Lee, Je Chul (2020). Synergy between Florfenicol and Aminoglycosides against Multidrug-Resistant Escherichia coli Isolates from Livestock *Antibiotics*, 9(4), 185

219. Pérez-Etayo, Lara, González, David, Vitas, Ana Isabel (2020). The Aquatic Ecosystem, a Good Environment for the Horizontal Transfer of Antimicrobial Resistance and Virulence-Associated Factors Among Extended Spectrum β-lactamases Producing E. coli *Microorganisms*, 8(4), 568

220. Adator, Emelia H, Walker, Matthew, Narvaez-Bravo, Claudia, Zaheer, Rahat, Goji, Noriko, Cook, Shaun R, Tymensen, Lisa, Hannon, Sherry J, Church, Deirdre, Booker, Calvin W, Amoako, Kingsley, Nadon, Celine A, Read, Ron, McAllister, Tim A (2020). Whole

Genome Sequencing Differentiates Presumptive Extended Spectrum Beta-Lactamase Producing Escherichia coli along Segments of the One Health Continuum *Microorganisms*, 8(3), 448

221. Ishibashi, Sawako, Sumiyama, Daisuke, Kanazawa, Tomoko, Murata, Koichi (2019). Prevalence of antimicrobial-resistant Escherichia coli in endangered Okinawa rail (Gallirallus okinawae) inhabiting areas around a livestock farm *Veterinary Medicine and Science*, 5(4), 563

222. Barour, Djanette, Berghiche, Amine, Nadji Boulebda (2019). Antimicrobial resistance of Escherichia coli isolates from cattle in Eastern Algeria *Veterinary World*, 12(8), 1195

223. Long, Haiyan, Yu, Feng, Ma, Ke, Liu, Lu, McNally, Alan, Zong Zhiyong (2019). The co-transfer of plasmid-borne colistin-resistant genes mcr-1 and mcr-3.5, the carbapenemase gene blaNDM-5 and the 16S methylase gene rmtB from Escherichia coli *Scientific Reports (Nature Publisher Group)*, 9(1), #Pages#

224. Qiu, Jianhua, Jiang, Zhiyu, Ju, Zijing, Zhao, Xiaonan, Yang, Jie, Guo, Huijun, Sun, Shuhong (2019). Molecular and Phenotypic Characteristics of Escherichia coli Isolates from Farmed Minks in Zhucheng, China *BioMed Research International*, 2019(#issue#), 12

225. Manges, Amee R, Thuras, Paul, Porter, Stephen, Johnson, James R (2019). Self-reported risk factors for having Escherichia coli sequence type 131 or its H30 subclone among US Veterans with a clinical E. coli isolate *Epidemiology and Infection*, 147(#issue#), #Pages#

226. Navarro-Gonzalez, Nora, Castillo-Contreras, Raquel, Casas-Díaz, Encarna, Morellet, Nicolas, Porrero, M Concepción, Molina-Vacas, Guillem, Torres, Rita T, Fonseca, Carlos, Mentaberre, Gregorio, Domínguez, Lucas, Lavín, Santiago, Serrano, Emmanuel (2018). Carriage of antibiotic-resistant bacteria in urban versus rural wild boars *European Journal of Wildlife Research*, 64(5), 1

227. Tadesse, Daniel A, Li, Cong, Mukherjee, Sampa, Hsu, Chih-Hao, Bodeis Jones, Sonya, Gaines, Stuart A, Kabera, Claudine, Loneragan, Guy H, Torrence, Mary, Harhay, Dayna M, McDermott, Patrick F, Zhao, Shaohua (2018). Whole-Genome Sequence Analysis of CTX-M Containing Escherichia coli Isolates from Retail Meats and Cattle in the United States *Microbial Drug Resistance*, 24(7), 939

228. Bouhamed, Radia, Bouayad, Leila, Messad, Sara, Safia Zenia, Malek Naim, Taha-Mossadak Hamdi (2018). Sources of contamination, prevalence, and antimicrobial resistance of thermophilic Campylobacter isolated from turkeys *Veterinary World*, 11(8), 1074

229. Lugsomya, Kittitat, Chatsuwan, Thanitta, Niyomtham, Waree, Tummaruk, Padet, Hampson, David J, Prapasarakul, Nuvee (2018). Routine Prophylactic Antimicrobial Use Is Associated with Increased Phenotypic and Genotypic Resistance in Commensal Escherichia coli Isolates Recovered from Healthy Fattening Pigs on Farms in Thailand *Microbial Drug*

Resistance, 24(2), 213

230. Malekzadegan, Yalda, Khashei, Reza, Ebrahim-Saraie, Hadi Sedigh, Jahanabadi, Zahra (2018). Distribution of virulence genes and their association with antimicrobial resistance among uropathogenic Escherichia coli isolates from Iranian patients *BMC Infectious Diseases*, 18(#issue#), #Pages#

231. Kohansal, Maryam, Ali Ghanbari Asad (2018). Molecular analysis of Shiga toxin-producing Escherichia coli O157:H7 and non-O157 strains isolated from calves *The Onderstepoort Journal of Veterinary Research*, 85(1), #Pages#

232. Walther, Birgit, Klein, Katja-Sophia, Barton, Ann-Kristin, Semmler, Torsten, Huber, Charlotte, Silver, Anthony Wolf, Tedin, Karsten, Merle, Roswitha, Mitrach, Franziska, Guenther, Sebastian, Lübke-Becker, Antina, Gehlen, Heidrun (2018). Extended-spectrum beta-lactamase (ESBL)-producing Escherichia coli and Acinetobacter baumannii among horses entering a veterinary teaching hospital: The contemporary "Trojan Horse" *PLoS One*, 13(1), #Pages#

233. Sala, Andrea, Cabassi, Clotilde Silvia, Santospirito, Davide, Polverini, Eugenia, Flisi, Sara, Cavirani, Sandro, Taddei, Simone (2018). Novel Naja atra cardiotoxin 1 (CTX-1) derived antimicrobial peptides with broad spectrum activity *PLoS One*, 13(1), #Pages#

234. Ukah, U V, Glass, M, Avery, B, Daignault, D, Mulvey, M R, Reid-Smith, R J, Parmley, E J, Portt, A, Boerlin, P, Manges, A R (2018). Risk factors for acquisition of multidrug-resistant Escherichia coli and development of community-acquired urinary tract infections *Epidemiology and Infection*, 146(1), 46

235. Blondeau, Joseph M (2017). Antimicrobial resistance & ‘Man's best friend’: what they give to us we might be giving right back *Future Microbiology*, 12(7), 549

236. Wang, Jing, Ma, Zhen-Bao, Zeng, Zhen-Ling, Yang, Xue-Wen, Huang, Ying, Liu, Jian-Hua (2017). The role of wildlife (wild birds) in the global transmission of antimicrobial resistance genes *Zoological Research*, 38(2), 55

237. Szczepanska, Bernadeta, Andrzejewska, Magorzata, Spica, Dorota, Klawe, Jacek J (2017). Prevalence and antimicrobial resistance of Campylobacter jejuni and Campylobacter coli isolated from children and environmental sources in urban and suburban areas *BMC Microbiology*, 17(#issue#), #Pages#

238. Nowakiewicz, Aneta, Ziółkowska, Grażyna, Zięba, Przemysław, Gnat, Sebastian, Trościańczyk, Aleksandra, Adaszek, Łukasz (2017). Characterization of Multidrug Resistant E. faecalis Strains from Pigs of Local Origin by ADSRRS-Fingerprinting and MALDI -TOF MS; Evaluation of the Compatibility of Methods Employed for Multidrug Resistance Analysis *PLoS One*, 12(1), #Pages#

239. Mir, Raies A, Weppelmann, Thomas A, Johnson, Judith A, Archer, Douglas, Morris, J Glenn, Jr, KwangCheol Casey Jeong (2016). Identification and Characterization of Cefotaxime Resistant Bacteria in Beef Cattle *PLoS One*, 11(9), #Pages#

240. Saviolli, Juliana Yuri, Vieira Cunha, Marcos Paulo, Lopes Guerra, Maria Flávia, Irino, Kinue, Catão-Dias, José Luiz, de Carvalho, Vania Maria (2016). Free-Ranging Frigates (Fregata magnificens) of the Southeast Coast of Brazil Harbor Extraintestinal Pathogenic Escherichia coli Resistant to Antimicrobials *PLoS One*, 11(2), #Pages#

241. Rodriguez, C, Taminiau, B, Van Broeck, J, Delmée, M, Daube, G (2016). Clostridium difficile in Food and Animals: A Comprehensive Review. *Advances in experimental medicine and biology*, 932(#issue#), 65

242. Sunde, Marianne, Simonsen, Gunnar Skov, Jannice Schau Slettemeås, Böckerman, Inger, Norström, Madelaine (2015). Integron, Plasmid and Host Strain Characteristics of Escherichia coli from Humans and Food Included in the Norwegian Antimicrobial Resistance Monitoring Programs *PLoS One*, 10(6), #Pages#

243. Field, Des, Gaudin, Noémie, Lyons, Francy, O'Connor, Paula M, Cotter, Paul D, Hill, Colin, Ross, R Paul (2015). A Bioengineered Nisin Derivative to Control Biofilms of Staphylococcus pseudintermedius *PLoS One*, 10(3), #Pages#

244. Shobrak, Mohammed Y, Abo-Amer, Aly E (2014). Role of wild birds as carriers of multi-drug resistant Escherichia coli and Escherichia vulneris *Brazilian Journal of Microbiology*, 45(4), 1199

245. Cristóbal-Azkarate, Jurgi, Dunn, Jacob C, Day, Jennifer M W, Amábile-Cuevas, Carlos F (2014). Resistance to Antibiotics of Clinical Relevance in the Fecal Microbiota of Mexican Wildlife *PLoS One*, 9(9), #Pages#

246. Shin, Seung Won, Byun, Jae-won, Jung, Myounghwan, Shin, Min-kyoung, Yoo, Han Sang (2014). Antimicrobial resistance, virulence genes and PFGE-profiling of Escherichia coli isolates from South Korean cattle farms *The Journal of Microbiology*, 52(9), 785

247. Alghoribi, Majed F, Gibreel, Tarek M, Dodgson, Andrew R, Beatson, Scott A, Upton, Mathew (2014). Galleria mellonella Infection Model Demonstrates High Lethality of ST69 and ST127 Uropathogenic E. coli *PLoS One*, 9(7), #Pages#

248. Yu-Fang, Guo, Wen-Hui, Zhang, Si-Qi, Ren, Yang, Lin, Dian-Hong Lü, Zhen-Ling Zeng, Ya-Hong, Liu, Hong-Xia, Jiang (2014). IncA/C Plasmid-Mediated Spread of CMY-2 in Multidrug-Resistant Escherichia coli from Food Animals in China *PLoS One*, 9(5), #Pages#

249. Ma, Licai, Shen, Zhangqi, Gaowa Naren, Li, Hui, Xia, Xi, Wu, Congming, Shen, Jianzhong, Zhang, Qijing, Wang, Yang (2014). Identification of a Novel G2073A Mutation in 23S rRNA in Amphenicol-Selected Mutants of Campylobacter jejuni *PLoS One*, 9(4),

#Pages#

250. Guenther, Sebastian, Bethe, Astrid, Fruth, Angelika, Semmler, Torsten, Ulrich, Rainer G, Wieler, Lothar H, Ewers, Christa (2012). Frequent Combination of Antimicrobial Multiresistance and Extraintestinal Pathogenicity in Escherichia coli Isolates from Urban Rats (Rattus norvegicus) in Berlin, Germany *PLoS One*, 7(11), #Pages#

251. Ishihara, Kanako, Hosokawa, Yuko, Makita, Kohei, Noda, Jun, Ueno, Hiroshi, Muramatsu, Yasukazu, Mukai, Takeshi, Yamamoto, Hideaki, Ito, Masaki, Tamura, Yutaka (2012). Factors associated with antimicrobial-resistant Escherichia coli in zoo animals *Research in Veterinary Science*, 93(2), 574

252. Glenn, Lashanda M, Englen, Mark D, Lindsey, Rebecca L, Frank, Joseph F, Turpin, Jennifer E, Berrang, Mark E, Meinersmann, Richard J, Fedorka-Cray, Paula J, Frye, Jonathan G (2012). Analysis of Antimicrobial Resistance Genes Detected in Multiple-Drug-Resistant Escherichia coli Isolates from Broiler Chicken Carcasses *Microbial Drug Resistance*, 18(4), 453

253. Ahmed, Mohamed O, Williams, Nicola J, Clegg, Peter D, van Velkinburgh, Jennifer C, Baptiste, Keith E, Bennett, Malcolm (2012). Analysis of Risk Factors Associated with Antibiotic-Resistant Escherichia coli *Microbial Drug Resistance*, 18(2), 161

254. Kirchner, Miranda, Wearing, Heather, Hopkins, Katie L, Teale, Christopher (2011). Characterization of Plasmids Encoding Cefotaximases Group 1 Enzymes in Escherichia coli Recovered from Cattle in England and Wales *Microbial Drug Resistance*, 17(3), 463

255. Bélanger, Louise, Garenaux, Amélie, Harel, Josée, Boulianne, Martine, Nadeau, Eric, Dozois, Charles M (2011). Escherichia coli from animal reservoirs as a potential source of human extraintestinal pathogenic E. coli *Pathogens and Disease*, 62(1), 1

256. Falsafi Tahereh, Ebrahimi Monir, Asgarani Ezat, Mirtorabi Vahid (2009). The pattern, association with multidrug-resistance and transferability of plasmid-mediated tetracycline resistance inEscherichia coli isolates from the poultry in Iran *Annals of Microbiology*, 59(2), #Pages#

257. Donovan, David M, Foster-Frey, Juli (2008). LambdaSa2 prophage endolysin requires Cpl-7-binding domains and amidase-5 domain for antimicrobial lysis of streptococci *FEMS Microbiology Letters*, 287(1), 22

258. Dacosta, Mateus Matiuzzi, Drescher, Guilherme, Maboni, Franciele, Weber, Shana, Botton, Sônia Deavila, Vainstein, Marilene Henning, Schrank, Irene Silveira, Devargas, Agueda Castagna (2008). Virulence factors and antimicrobial resistance of escherichia coli isolated from urinary tract of swine in southern of Brazil *Brazilian Journal of Microbiology*, 39(4), 741

259. Dai, Lei, Li-Ming, Lu, Cong-Ming Wu, Li, Bei-Bei, Si-Yang, Huang, Shao-Chen, Wang, Yong-Hua, Qi, Jian-Zhong, Shen (2008). Characterization of antimicrobial resistance among Escherichia coli isolates from chickens in China between 2001 and 2006 *FEMS Microbiology Letters*, 286(2), 178

260. Garcia-Fernandez, Aurora, Chiaretto, Giuseppina, Bertini, Alessia, Villa, Laura, Fortini, Daniela, Ricci, Antonia, Carattoli, Alessandra (2008). Multilocus sequence typing of IncI1 plasmids carrying extended-spectrum [beta]-lactamases in Escherichia coli and Salmonella of human and animal origin *The Journal of Antimicrobial Chemotherapy*, 61(6), 1229

261. Kadlec, Kristina, Kehrenberg, Corinna, Schwarz, Stefan (2007). Efflux-mediated resistance to florfenicol and/or chloramphenicol in Bordetella bronchiseptica: identification of a novel chloramphenicol exporter *The Journal of Antimicrobial Chemotherapy*, 59(2), 191

262. Baldy-Chudzik, K, STOSIK, M (2007). Prevalence of Antibiotic Resistance Profile in Relation to Phylogenetic Background among Commensal Escherichia coli Derived from Various Mammals *Polish Journal of Microbiology*, 56(3), 175

263. DebRoy, Chitrita, Maddox, Carol W (2001). Identification of virulence attributes of gastrointestinal Escherichia coli isolates of veterinary significance *Animal Health Research Reviews*, 2(2), 129

264. de Lagarde, Maud, Larrieu, Caroline, Praud, Karine, Lallier, Nathalie, Trotereau, Angélina, Sallé, Guillaume, Fairbrother, John M, Schouler, Catherine, Doublet, Benoît (2020). Spread of multidrug-resistant IncHI1 plasmids carrying ESBL gene blaCTX-M-1 and metabolism operon of prebiotic oligosaccharides in commensal Escherichia coli from healthy horses, France *International journal of antimicrobial agents*, #volume#(#issue#), #Pages#

265. Wongtawan, T, Narinthorn, R, Sontigun, N, Sansamur, C, Petcharat, Y, Fungwithaya, P, Saengsawang, P, Blackall, PJ, Thomrongsuwannakij, T (2022). Characterizing the antimicrobial resistance profile of Escherichia coli found in sport animals (fighting cocks, fighting bulls, and sport horses) and soils from their environment *Veterinary World*, 15(11), 2673

266. Mitchell, PK, Wang, LY, Stanhope, BJ, Cronk, BD, Anderson, R, Mohan, S, Zhou, LJ, Sanchez, S, Bartlett, P, Maddox, C, DeShambo, V, Mani, R, Hengesbach, LM, Gresch, S, Wright, K, Mor, S, Zhang, SP, Shen, ZY, Yan, LF, Mackey, R, Franklin-Guild, R, Zhang, Y, Prarat, M, Shiplett, K, Ramachandran, A, Narayanan, S, Sanders, J, Hunkapiller, AA, Lahmers, K, Carbonello, AA, Aulik, N, Lim, A, Cooper, J, Jones, A, Guag, JK, Nemser, SM, Tyson, GH, Timme, R, Strain, E, Reimschuessel, R, Ceric, O, Goodman, LB (2022). Multi-laboratory evaluation of the Illumina iSeq platform for whole genome sequencing of Salmonella, Escherichia coli and Listeria *Microbial Genomics*, 8(2), #Pages#

267. Sigirci, BD, Celik, B, Halac, B, Adiguzel, MC, Kekec, I, Metiner, K, Ikiz, S, Bagcigil, AF, Ozgur, NY, Ak, S, Kahraman, BB (2020). Antimicrobial resistance profiles of Escherichia coli isolated from companion birds *Journal Of King Saud University Science*,

32(1), 1069

268. Bandyopadhyay, S, Samanta, I (2020). Antimicrobial Resistance in Agri-Food Chain and Companion Animals as a Re-emerging Menace in Post-COVID Epoch: Low-and Middle-Income Countries Perspective and Mitigation Strategies *Frontiers In Veterinary Science*, 7(#issue#), #Pages#

269. Ferriol-Gonzalez, C, Domingo-Calap, P (2021). Phage Therapy in Livestock and Companion Animals *Antibiotics-Basel*, 10(5), #Pages#

270. Steger, L, Rinder, M, Korbel, R (2020). Phenotypical antibiotic resistances of bacteriological isolates originating from pet, zoo and falconry birds *Tieraerztliche Praxis Ausgabe Kleintiere Heimtiere*, 48(04), 260

271. Toombs-Ruane, LJ, Benschop, J, Burgess, S, Priest, P, Murdoch, DR, French, NP (2017). Multidrug resistant Enterobacteriaceae in New Zealand: a current perspective *New Zealand Veterinary Journal*, 65(2), 62

272. Damborg, P, Morsing, MK, Petersen, T, Bortolaia, V, Guardabassi, L (2015). CTX-M-1 and CTX-M-15-producing Escherichia coli in dog faeces from public gardens *Acta Veterinaria Scandinavica*, 57(#issue#), #Pages#

273. Koeck, R, Cuny, C (2020). Multidrug-resistant bacteria in animals and humans *Medizinische Klinik-Intensivmedizin Und Notfallmedizin*, 115(3), 189

Level 1, Form level_1_screening, Does the title/abstract describe the pathogen E. coli? -> No

274. Skurnik, David, Ruimy, Raymond, Andremont, Antoine, Amorin, Christine, Rouquet, Pierre, Picard, Bertrand, Denamur, Erick (2006). Effect of human vicinity on antimicrobial resistance and integrons in animal faecal Escherichia coli. *The Journal of antimicrobial chemotherapy*, 57(6), 1215

275. JK Johnson, EN Perencevich, DP Lincalis, RA Venezia (2006). Dog bite transmission of antibiotic-resistant bacteria to a human. *Infection control and hospital epidemiology*, 27(7), #Pages#

276. Lloyd, David H (2007). Reservoirs of antimicrobial resistance in pet animals. *Clinical infectious diseases : an official publication of the Infectious Diseases Society of America*, 45 Suppl 2(#issue#), S148

277. S Le Hello, A Bekhit, SA Granier, H Barua, J Beutlich, M Zając, S Münch, V Sintchenko, B Bouchrif, K Fashae, JL Pinsard, L Sontag, L Fabre, M Garnier, V Guibert, P Howard, RS Hendriksen, JP Christensen, PK Biswas, A Cloeckaert, W Rabsch, D Wasyl, B Doublet, FX Weill (2013). The global establishment of a highly-fluoroquinolone resistant Salmonella enterica serotype Kentucky ST198 strain. *Frontiers in microbiology,* 4(), #Pages#

278. Yao, Hong, Wu, Dongfang, Lei, Lei, Shen, Zhangqi, Wang, Yang, Liao, Kang (2016). The detection of fosfomycin resistance genes in Enterobacteriaceae from pets and their owners. *Veterinary microbiology,* 193(#issue#), 67

279. Vangchhia, Belinda, Abraham, Sam, Bell, Jan M, Collignon, Peter, Gibson, Justine S, Ingram, Paul R, Johnson, James R, Kennedy, Karina, Trott, Darren J, Turnidge, John D, Gordon, David M (2016). Phylogenetic diversity, antimicrobial susceptibility and virulence characteristics of phylogroup F Escherichia coli in Australia. *Microbiology (Reading, England),* 162(11), 1904

280. Alcolea, Pedro J, Alonso, Ana, Larraga, Vicente (2019). The antibiotic resistance-free mammalian expression plasmid vector pPAL for development of third generation vaccines. *Plasmid,* 101(#issue#), 35

281. Bourely, Clemence, Cazeau, Geraldine, Jouy, Eric, Haenni, Marisa, Madec, Jean-Yves, Jarrige, Nathalie, Leblond, Agnes, Gay, Emilie (2019). Antimicrobial resistance of Pasteurella multocida isolated from diseased food-producing animals and pets. *Veterinary microbiology,* 235(#issue#), 280

282. Rumi, Maria Valeria, Nuske, Ezequiel, Mas, Javier, Arguello, Andrea, Gutkind, Gabriel, Di Conza, Jose (2021). Antimicrobial resistance in bacterial isolates from companion animals in Buenos Aires, Argentina: 2011-2017 retrospective study. *Zoonoses and public health,* 68(5), 516

283. V Dazio, A Nigg, JS Schmidt, M Brilhante, EI Campos-Madueno, N Mauri, SP Kuster, SG Brawand, B Willi, A Endimiani, V Perreten, S Schuller (2021). Duration of carriage of multidrug-resistant bacteria in dogs and cats in veterinary care and co-carriage with their owners. *One health (Amsterdam, Netherlands),* 13(), #Pages#

284. AM O'Neill, KA Worthing, N Kulkarni, F Li, T Nakatsuji, D McGrosso, RH Mills, G Kalla, JY Cheng, JM Norris, K Pogliano, J Pogliano, DJ Gonzalez, RL Gallo (2021). Antimicrobials from a feline commensal bacterium inhibit skin infection by drug-resistant <i>S. pseudintermedius</i>. *eLife,* 10(), #Pages#

285. Yin, Yi, Qiu, Lihao, Wang, Guizhen, Guo, Zhimin, Wang, Zhiqiang, Qiu, Jiazhang, Li, Ruichao (2022). Emergence and Transmission of Plasmid-Mediated Mobile Colistin Resistance Gene mcr-10 in Humans and Companion Animals. *Microbiology spectrum,* 10(5), e0209722

286. Ballash, Gregory A, Mollenkopf, Dixie F, Diaz-Campos, Dubraska, van Balen, Joany C, Cianciolo, Rachel E, Wittum, Thomas E (2022). Pathogenomics and clinical recurrence influence biofilm capacity of Escherichia coli isolated from canine urinary tract infections. *PloS one*, 17(8), e0270461

287. Ebani, V. V., Nardoni, S., Bertelloni, F., Pistelli, L., Mancianti, F. (2018). Antimicrobial activity of five essential oils against bacteria and fungi responsible for urinary tract infections. *Molecules*, 23(7), 1668

288. Vučković, S., Matanović, K., Martinec, B. Š. (2013). Prevalence and antimicrobial susceptibility of bacteria causing urinary tract infections in dogs and cats in Croatia. *5th International Congress "Veterinary Science and Profession", Zagreb, Croatia, 3-4 October 2013. Book of abstracts*, #volume#(#issue#), 80

289. Wagner, K. A., Hartmann, F. A., Trepanier, L. A. (2007). Bacterial culture results from liver, gallbladder, or bile in 248 dogs and cats evaluated for hepatobiliary disease: 1998-2003. *Journal of Veterinary Internal Medicine*, 21(3), 417

290. Farca, A. M., Cavana, P., Robino, P., Nebbia, P. (2007). In vitro antimicrobial activity of marbofloxacin and enrofloxacin against bacterial strains isolated from companion animals. *SAT, Schweizer Archiv für Tierheilkunde*, 149(6), 265

291. Park SeWon, Seo KyungWon, Hwang CheolYong, Youn HwaYoung, Han HongRyul (2004). Isolation of bacteria from clinical specimens in Veterinary Medical Teaching Hospital and trend of antimicrobial susceptibility. *Journal of Veterinary Clinics*, 21(1), 7

292. Lavin, Lindsey E, Maki, Lynn C (2023). Antimicrobial use in the surgical treatment of canine pyometra: A questionnaire survey of Arizona-licensed veterinarians *Veterinary Medicine and Science*, 9(3), 1124

293. de Sousa, Telma, Garcês, Andreia, Silva, Augusto, Lopes, Ricardo, Alegria, Nuno, Hébraud, Michel, Igrejas, Gilberto, Poeta, Patricia (2023). The Impact of the Virulence of Pseudomonas aeruginosa Isolated from Dogs *Veterinary Sciences*, 10(5), 343

294. Vercelli, Cristina, Amadori, Michela, Gambino, Graziana, Re, Giovanni (2023). Does Nitrofurantoin Improve the Portfolio of Vets against Resistant Bacteria in Companion Animals? *Antibiotics*, 12(5), 911

295. Szewczuk, Małgorzata Anna, Zych, Sławomir, Oster, Nicola, Karakulska, Jolanta (2023). Activity of Patchouli and Tea Tree Essential Oils against Staphylococci Isolated from Pyoderma in Dogs and Their Synergistic Potential with Gentamicin and Enrofloxacin *Animals*, 13(8), 1279

296. Ifarajimi Rapheal Olabode, Sachivkina, Nadezhda, Karamyan, Arfenia, Mannapova, Ramziya, Kuznetsova, Olga, Bobunova, Anna, Zhabo, Natallia, Avdonina, Marina, Gurina, Regina (2023). In Vitro Activity of Farnesol against Malassezia pachydermatis Isolates from

Otitis Externa Cases in Dogs *Animals*, 13(7), 1259

297. Yudhanto, Setyo, Varga, Csaba (2023). Knowledge and Attitudes of Small Animal Veterinarians on Antimicrobial Use Practices Impacting the Selection of Antimicrobial Resistance in Dogs and Cats in Illinois, United States: A Spatial Epidemiological Approach *Antibiotics*, 12(3), 542

298. Candellone, Alessia, Badino, Paola, Girolami, Flavia, Ala, Ugo, Mina, Floriana, Odore, Rosangela (2023). Dog Owners' Attitude toward Veterinary Antibiotic Use and Antibiotic Resistance with a Focus on Canine Diarrhea Management *Animals*, 13(6), 1061

299. Scarborough, Ri O, Sri, Anna E, Browning, Glenn F, Hardefeldt, Laura Y, Bailey, Kirsten E (2023). 'Brave Enough': A Qualitative Study of Veterinary Decisions to Withhold or Delay Antimicrobial Treatment in Pets *Antibiotics*, 12(3), 540

300. Hanna, Walter, Verspohl, Jutta, Meißner, Jessica, Oltmanns, Hilke, Geks, Anna Karoline, Busse, Claudia (2023). In Vitro Antimicrobial Activity of N-Acetylcysteine against Pathogens Most Commonly Associated with Infectious Keratitis in Dogs and Cats *Antibiotics*, 12(3), 559

301. Cunha, Eva, Ferreira, Ana Filipa, Valente, Sara, Matos, Alice, Luís Miguel Carreira, Videira, Marta, Chambel, Lélia, Tavares, Luís, Oliveira, Manuela (2023). In Vivo Effect of a Nisin–Biogel on the Antimicrobial and Virulence Signatures of Canine Oral Enterococci *Antibiotics*, 12(3), 468

302. Ebani, Valentina Virginia, Pieracci, Ylenia, Cagnoli, Giulia, Bertelloni, Fabrizio, Munafò, Chiara, Nardoni, Simona, Pistelli, Luisa, Mancianti, Francesca (2023). In Vitro Antimicrobial Activity of Thymus vulgaris, Origanum vulgare, Satureja montana and Their Mixture against Clinical Isolates Responsible for Canine Otitis Externa *Veterinary Sciences*, 10(1), 30

303. Tumpa, Andrea, Stritof, Zrinka Štritof, Pintaric Selma Pintarić (2022). Prevalence and antimicrobial susceptibility of Enterococcus spp. from urine of dogs and cats in northwestern Croatia *Research in Veterinary Science*, 151(#issue#), 42

304. Maggi, Ricardo Guillermo, Halls, Vicky, Krämer, Friederike, Lappin, Michael, Pennisi, Maria Grazia, Peregrine, Andrew S, Roura, Xavier, Schunack, Bettina, Scorza, Valeria, Tasker, Séverine, Baneth, Gad, Bourdeau, Patrick, Bowman, Dwight D, Breitschwerdt, Edward B, Capelli, Gioia, Cardoso, Luís, Dantas-Torres, Filipe, Dobler, Gerhard, Ferrer, Lluís, Gradoni, Luigi, Irwin, Peter, Jongejan, Frans, Kempf, Volkhard A J, Kohn, Barbara, Little, Susan, Madder, Maxime, Maia, Carla, Marcondes, Mary, Miró, Guadalupe, Naucke, Torsten, Oliva, Gaetano, Otranto, Domenico, Penzhorn, Barend L, Pfeffer, Martin, Sainz, Ángel, Shin, SungShik, Solano-Gallego, Laia, Straubinger, Reinhard K, Traub, Rebecca, Wright, Ian (2022). Vector-borne and other pathogens of potential relevance disseminated by relocated cats. *Parasites & vectors*, 15(1), 415

305. Flávia Mello Viegas, Contributed equally to this work with: Flávia Mello Viegas, Jordana Almeida Santana, Brendhal Almeida Silva, Rafael Gariglio Clark Xavier, Cláudia Teixeira Bonisson, Júlia Lara Sette Câmara, Rennó, Mário Cesar, Reis Cunha, João Luis, Pereira Figueiredo, Henrique César, Faria Lobato, Francisco Carlos, Silveira Silva, Rodrigo Otávio, Rodrigo Otávio Silveira Silva Brendhal Almeida Silva, Rodrigo Otávio Silveira Silva Rafael Gariglio Clark Xavier, Rodrigo Otávio Silveira Silva Cláudia Teixeira Bonisson, Rodrigo Otávio Silveira Silva Júlia Lara Sette Câmara, Rodrigo Otávio Silveira Silva Mário Cesar Rennó, Rodrigo Otávio Silveira Silva João Luis Reis Cunha, Rodrigo Otávio Silveira Silva Henrique César Pereira Figueiredo, Rodrigo Otávio Silveira Silva Rodrigo Otávio Silveira Silva Contributed equally to this work with: Flávia Mello Viegas (2022). Occurrence and characterization of methicillin-resistant Staphylococcus spp. in diseased dogs in Brazil *PLoS One*, 17(6), #Pages#

306. Allen, Joanne L, Doidge, Nicholas P, Bushell, Rhys N, Browning, Glenn F, Marenda, Marc S (2022). Healthcare-associated infections caused by chlorhexidine-tolerant Serratia marcescens carrying a promiscuous IncHI2 multi-drug resistance plasmid in a veterinary hospital *PLoS One*, 17(3), #Pages#

307. Bellato, Alessandro, Robino, Patrizia, Stella, Maria Cristina, Scarrone, Laura, Scalas, Daniela, Nebbia, Patrizia (2022). Resistance to Critical Important Antibacterials in Staphylococcus pseudintermedius Strains of Veterinary Origin *Antibiotics*, 11(12), 1758

308. Viñes, Joaquim, Fàbregas, Norma, Pérez, Daniel, Cuscó, Anna, Fonticoba, Rocío, Francino, Olga, Ferrer, Lluís, Migura-Garcia, Lourdes (2022). Concordance between Antimicrobial Resistance Phenotype and Genotype of Staphylococcus pseudintermedius from Healthy Dogs *Antibiotics*, 11(11), 1625

309. Kwon, Jun, Yang, Myoung-Hwan, Ko, Hyoung-Joon, Sang-Guen Kim, Park, Chul, Se-Chang, Park (2022). Antimicrobial Resistance and Virulence Factors of Proteus mirabilis Isolated from Dog with Chronic Otitis Externa *Pathogens*, 11(10), 1215

310. Grakh, Kushal, Mittal, Dinesh, Kumar, Tarun, Thakur, Swati, Panwar, Diksha, Singh, Lokender, Kumar, Manesh, Jindal, Naresh (2022). Attitude, Opinions, and Working Preferences Survey among Pet Practitioners Relating to Antimicrobials in India *Antibiotics*, 11(10), 1289

311. Lin, Haoyi, Liu, Zhihui, Zhou, Yingchun, Lu, Weiguo, Xu, Qian (2022). Characterization of Resistance and Virulence of Pasteurella multocida Isolated from Pet Cats in South China *Antibiotics*, 11(10), 1387

312. Stefańska, Ilona, Kwiecień, Ewelina, Kizerwetter-Świda, Magdalena, Chrobak-Chmiel, Dorota, Rzewuska, Magdalena (2022). Tetracycline, Macrolide and Lincosamide Resistance in Streptococcus canis Strains from Companion Animals and Its Genetic Determinants *Antibiotics*, 11(8), 1034

313. Muñoz-Ibarra, Eleonora, Molina-López, Rafael A, Durán, Inma, Biel Garcias, Martín, Marga, Darwich, Laila (2022). Antimicrobial Resistance in Bacteria Isolated from Exotic Pets: The Situation in the Iberian Peninsula *Animals*, 12(15), 1912

314. Murawska, Małgorzata, Sypecka, Monika, Bartosik, Justyna, Kwiecień, Ewelina, Rzewuska, Magdalena, Sałamaszyńska-Guz, Agnieszka (2022). Should We Consider Them as a Threat? Antimicrobial Resistance, Virulence Potential and Genetic Diversity of Campylobacter spp. Isolated from Varsovian Dogs *Antibiotics*, 11(7), 964

315. Marco-Fuertes, Ana, Marin, Clara, Lorenzo-Rebenaque, Laura, Vega, Santiago, Montoro-Dasi, Laura (2022). Antimicrobial Resistance in Companion Animals: A New Challenge for the One Health Approach in the European Union *Veterinary Sciences*, 9(5), 208

316. Marques, Cátia, Belas, Adriana, Menezes, Juliana, Joana Moreira da Silva, Cavaco-Silva, Patrícia, Trigueiro, Graça, Gama, Luís T, Pomba, Constança (2022). Human and Companion Animal Proteus mirabilis Sharing *Microbiology Research*, 13(1), 38

317. Hamame, Afaf, Davoust, Bernard, Rolain, Jean-Marc, Diene, Seydina M (2022). Screening of Colistin-Resistant Bacteria in Domestic Pets from France *Animals*, 12(5), 633

318. Schirò, Giorgia, Gambino, Delia, Mira, Francesco, Vitale, Maria, Guercio, Annalisa, Purpari, Giuseppa, Antoci, Francesco, Licitra, Francesca, Chiaramonte, Gabriele, Maria La Giglia, Randazzo, Vincenzo, Vicari, Domenico (2022). Antimicrobial Resistance (AMR) of Bacteria Isolated from Dogs with Canine Parvovirus (CPV) Infection: The Need for a Rational Use of Antibiotics in Companion Animal Health *Antibiotics*, 11(2), 142

319. Jimenez-Trigos, Estrella, Toquet, Marion, Barba, Marta, Gómez-Martín, Ángel, Quereda, Juan J, Bataller, Esther (2022). Search of antimicrobial lactic acid bacteria from Salmonella-negative dogs *BMC Veterinary Research*, 18(#issue#), 1

320. Usmael, Belisa, Abraha, Bruk, Mummed, Sisay Alemuhar, Hiko, Adem, Abdurehman, Abdallahi (2022). Isolation, antimicrobial susceptibility patterns, and risk factors assessment of non-typhoidal Salmonella from apparently healthy and diarrheic dogs *BMC Veterinary Research*, 18(#issue#), 1

321. O'Neill, Alan M, Worthing, Kate A, Kulkarni, Nikhil, Li, Fengwu, Nakatsuji, Teruaki, McGrosso, Dominic, Mills, Robert H, Kalla, Gayathri, Cheng, Joyce Y, Norris, Jacqueline M, Pogliano, Kit, Pogliano, Joe, Gonzalez, David J, Gallo, Richard L (2021). Antimicrobials from a feline commensal bacterium inhibit skin infection by drug-resistant S. pseudintermedius. *eLife*, 10(#issue#), #Pages#

322. Elnageh, Hiam R, Hiblu, Murad A, Abbassi, Mohamed S, Abouzeed, Yousef M, Ahmed, Mohamed O (2021). Prevalence and antimicrobial resistance of Salmonella serotypes isolated from cats and dogs in Tripoli, Libya. *Veterinaria Italiana*, 57(2), 111

323. Dégi, János, Imre, Kálmán, Herman, Viorel, Bucur, Iulia, Radulov, Isidora, Oana-Cătălina Petrec, Romeo, Teodor Cristina (2021). Antimicrobial Drug-Resistant Salmonella in Urban Cats: Is There an Actual Risk to Public Health? *Antibiotics*, 10(11), 1404

324. Prouillac, Caroline (2021). Use of Antimicrobials in a French Veterinary Teaching Hospital: A Retrospective Study *Antibiotics*, 10(11), 1369

325. Stępień-Pyśniak, Dagmara, Bertelloni, Fabrizio, Dec, Marta, Cagnoli, Giulia, Pietras-Ożga, Dorota, Urban-Chmiel, Renata, Ebani, Valentina Virginia (2021). Characterization and Comparison of Enterococcus spp. Isolates from Feces of Healthy Dogs and Urine of Dogs with UTIs *Animals*, 11(10), 2845

326. Pignataro, Giulia, Roberta Di Prinzio, Crisi, Paolo Emidio, Belà, Benedetta, Fusaro, Isa, Trevisan, Carlo, De Acetis, Luigi, Gramenzi, Alessandro (2021). Comparison of the Therapeutic Effect of Treatment with Antibiotics or Nutraceuticals on Clinical Activity and the Fecal Microbiome of Dogs with Acute Diarrhea *Animals*, 11(6), 1484

327. Sofia Santos Costa, Oliveira, Valéria, Serrano, Maria, Pomba, Constança, Couto, Isabel (2021). Phenotypic and Molecular Traits of Staphylococcus coagulans Associated with Canine Skin Infections in Portugal *Antibiotics*, 10(5), 518

328. Elmoslemany, Ahmed, Ibrahim Elsohaby, Alorabi, Mohammed, Alkafafy, Mohamed, Al-Marri, Theeb, Aldoweriej, Ali, Alaql, Fanan A, Almubarak, Abdullah, Mahmoud Fayez (2021). Diversity and Risk Factors Associated with Multidrug and Methicillin-Resistant Staphylococci Isolated from Cats Admitted to a Veterinary Clinic in Eastern Province, Saudi Arabia *Antibiotics*, 10(4), 367

329. Willis Gwenzi, Chaukura, Nhamo, Muisa-Zikali, Norah, Teta, Charles, Musvuugwa, Tendai, Rzymski, Piotr, Akebe Luther King Abia (2021). Insects, Rodents, and Pets as Reservoirs, Vectors, and Sentinels of Antimicrobial Resistance *Antibiotics*, 10(1), 68

330. Galarce, Nicolás, Arriagada, Gabriel, Sánchez, Fernando, Venegas, Vladimir, Cornejo, Javiera, Lapierre, Lisette (2021). Antimicrobial Use in Companion Animals: Assessing Veterinarians' Prescription Patterns through the First National Survey in Chile *Animals*, 11(2), 348

331. Wada, Y., Irekeola, A. A., EAR, E. N. S., Yusof, W., Lih Huey, L., Ladan Muhammad, S., ... & Zaidah, A. R. (2021). Prevalence of vancomycin-resistant Enterococcus (VRE) in companion animals: the first meta-analysis and systematic review. *Antibiotics*, *10*(2), 138.

332. Lynch, S. A., & Helbig, K. J. (2021). *The complex diseases of Staphylococcus pseudintermedius in Canines: where to next? Veterinary Sciences, 8, 11.*

333. Brosseau, Gabrielle, Pagé, Nadia, de Jaham, Caroline, Jérôme R E del Castillo (2020). Medical honey for canine nasal intertrigo: A randomized, blinded, placebo-controlled,

adaptive clinical trial to support antimicrobial stewardship in veterinary dermatology *PLoS One*, 15(8), #Pages#

334. Välkki, Kirsi Johanna, Katariina Hanne Thomson, Thomas Sven Christer Grönthal, Jouni Juho Tapio Junnila, Merja Hilma Johanna Rantala, Laitinen-Vapaavuori, Outi Maria, Mölsä, Sari Helena (2020). Antimicrobial prophylaxis is considered sufficient to preserve an acceptable surgical site infection rate in clean orthopaedic and neurosurgeries in dogs *Acta Veterinaria Scandinavica*, 62(#issue#), 1

335. Bataller, E, García-Romero, E, Llobat, L, Lizana, V, Jiménez-Trigos, E (2020). Dogs as a source of Salmonella spp. in apparently healthy dogs in the Valencia Region. Could it be related with intestinal lactic acid bacteria? *BMC Veterinary Research*, 16(#issue#), 1

336. Wu, Xin, Angkititrakul, Sunpetch, Richards, Allen L, Pulsrikarn, Chaiwat, Seri Khaengair, Keosengthong, Amphone, Siriwong, Supatcharee, Suksawat, Fanan (2020). Risk of Antimicrobial Resistant Non-Typhoidal Salmonella during Asymptomatic Infection Passage between Pet Dogs and Their Human Caregivers in Khon Kaen, Thailand *Antibiotics*, 9(8), 477

337. Woo, Kyung Jung, Shin, Sook, Park, Young Kyung, Suk-Kyung Lim, Dong-Chan, Moon, Kun Taek Park, Yong Ho Park (2020). Distribution and antimicrobial resistance profiles of bacterial species in stray cats, hospital-admitted cats, and veterinary staff in South Korea *BMC Veterinary Research*, 16(#issue#), 1

338. Park, Youjin, Oh, Jaeyoung, Park, Sowon, Sum, Samuth, Song, Wonkeun, Chae, Jongchan, Park, Heemyung (2020). Antimicrobial resistance and novel mutations detected in the gyrA and parC genes of Pseudomonas aeruginosa strains isolated from companion dogs *BMC Veterinary Research*, 16(#issue#), 1

339. Binagia, Erin M, Levy, Nyssa A (2020). Salmonella Mesenteric Lymphadenitis Causing Septic Peritonitis in Two Dogs *Veterinary Medicine : Research and Reports*, 11(#issue#), 25

340. Gahamanyi, Noel, Mboera, Leonard E G, Matee, Mecky I, Mutangana, Dieudonné, Komba, Erick V G (2020). Prevalence, Risk Factors, and Antimicrobial Resistance Profiles of Thermophilic Campylobacter Species in Humans and Animals in Sub-Saharan Africa: A Systematic Review *International Journal of Microbiology*, 2020(#issue#), 12

341. Norris, Jacqueline M, Zhuo, Annie, Govendir, Merran, Rowbotham, Samantha J, Labbate, Maurizio, Degeling, Chris, Gilbert, Gwendolyn L, Dominey-Howes, Dale, Ward, Michael P (2019). Factors influencing the behaviour and perceptions of Australian veterinarians towards antibiotic use and antimicrobial resistance *PLoS One*, 14(10), #Pages#

342. Iannino, Filomena, Salucci, Stefania, Guido Di Donato, Badagliacca, Pietro, Vincifori, Giacomo, Elisabetta Di Giannatale (2019). Campylobacter and antimicrobial resistance in dogs and humans: "One health" in practice *Veterinaria Italiana*, 55(3), 203

343. Schmitt, K, Lehner, C, Schuller, S, Schüpbach-Regula, G, Mevissen, M, Peter, R, Müntener, C R, Naegeli, H, Willi, B (2019). Antimicrobial use for selected diseases in cats in Switzerland *BMC Veterinary Research*, 15(#issue#), #Pages#

344. Saam Torkan, Vazirian, Behnam, Khamesipour, Faham, Dida, Gabriel O (2018). Prevalence of thermotolerant Campylobacter species in dogs and cats in Iran *Veterinary Medicine and Science*, 4(4), 296

345. Hardefeldt, Laura, Nielsen, Torben, Crabb, Helen, Gilkerson, James, Squires, Richard, Heller, Jane, Sharp, Claire, Cobbold, Rowland, Norris, Jacqueline, Browning, Glenn (2018). Veterinary Students' Knowledge and Perceptions About Antimicrobial Stewardship and Biosecurity—A National Survey *Antibiotics*, 7(2), #Pages#

346. Van Balen, J C, Landers, T, Nutt, E, Dent, A, Hoet, A E (2017). Molecular epidemiological analysis to assess the influence of pet-ownership in the biodiversity of Staphylococcus aureus and MRSA in dog- and non-dog-owning healthy households *Epidemiology and Infection*, 145(6), 1135

347. Saputra, Sugiyono, Jordan, David, Worthing, Kate A, Norris, Jacqueline M, Wong, Hui S, Abraham, Rebecca, Trott, Darren J, Abraham, Sam (2017). Antimicrobial resistance in coagulase-positive staphylococci isolated from companion animals in Australia: A one year study *PLoS One*, 12(4), #Pages#

348. Harada, Kazuki, Shimizu, Takae, Mukai, Yujiro, Kuwajima, Ken, Sato, Tomomi, Kajino, Akari, Usui, Masaru, Tamura, Yutaka, Kimura, Yui, Miyamoto, Tadashi, Tsuyuki, Yuzo, Ohki, Asami, Kataoka, Yasushi (2017). Phenotypic and molecular characterization of antimicrobial resistance in Enterobacter spp. isolates from companion animals in Japan *PLoS One*, 12(3), #Pages#

349. Zhu, Minwen, Miao, Bo, Zhu, Jianhua, Wang, Haiyan, Zhou, Zengtong (2017). Transplantation of periodontal ligament cell sheets expressing human β-defensin-3 promotes anti-inflammation in a canine model of periodontitis *Molecular Medicine Reports*, 16(5), 7459

350. Maria Angeles Argudín, Deplano, Ariane, Meghraoui, Alaeddine, Dodemont, Magali, Heinrichs, Amelie, Olivier, Denis, Nonhoff, Claire, Roisin, Sandrine (2017). Bacteria from Animals as a Pool of Antimicrobial Resistance Genes *Antibiotics*, 6(2), 12

351. Bitsu Kiflu, Haile Alemayehu, Abdurahaman, Mukarim, Negash, Yohannes, Tadesse Eguale (2017). Salmonella serotypes and their antimicrobial susceptibility in apparently healthy dogs in Addis Ababa, Ethiopia *BMC Veterinary Research*, 13(#issue#), #Pages#

352. Selwet, Marek, Galbas, Mariola, Słomski, Ryszard, Cłapa, Tomasz, Porzucek, Filip (2016). Monitoring of virulence genes, drug-resistance in Campylobacter coli isolated from golden retrievers *Polish Journal of Microbiology*, 65(2), 7

353. Olkkola, Satu, Kovanen, Sara, Roine, Johanna, Hänninen, Marja-Liisa, Hielm-Björkman, Anna, Kivistö, Rauni (2015). Population Genetics and Antimicrobial Susceptibility of Canine Campylobacter Isolates Collected before and after a Raw Feeding Experiment *PLoS One*, 10(7), #Pages#

354. Ruddat, I, Tietze, E, Ziehm, D, Kreienbrock, L (2014). Associations between host characteristics and antimicrobial resistance of Salmonella Typhimurium *Epidemiology and Infection*, 142(10), 2085

355. Schmidt, Vanessa M, Williams, Nicola J, Pinchbeck, Gina, Corless, Caroline E, Shaw, Stephen, McEwan, Neil, Dawson, Susan, Nuttall, Tim (2014). Antimicrobial resistance and characterisation of staphylococci isolated from healthy Labrador retrievers in the United Kingdom *BMC Veterinary Research*, 10(#issue#), 17

356. Andrzejewska, M, Szczepanska, B, Klawe, J J, Spica, D, Chudzinska, M (2013). Prevalence of Campylobacter jejuni and Campylobacter coli species in cats and dogs from Bydgoszcz (Poland) region *Polish Journal of Veterinary Sciences*, 16(1), 115

357. Lechowski, R, Cotard, J P, Boulouis, H J, Kietzman, M, Farca, A M, Fontaine, J, Caney, S, Dupree, G (2013). Proper use of Quinolones for canine colitis ambulatory treatment: literature review and REQUEST guidelines *Polish Journal of Veterinary Sciences*, 16(1), 193

358. Ghosh, Anuradha, Dowd, Scot E, Zurek, Ludek (2011). Dogs Leaving the ICU Carry a Very Large Multi-Drug Resistant Enterococcal Population with Capacity for Biofilm Formation and Horizontal Gene Transfer *PLoS One*, 6(7), #Pages#

359. Loutet, Slade A. (2010). Antimicrobial peptide resistance of Burkholderia cenocepacia *#journal#*, #volume#(#issue#), 284

360. Krutkiewicz, A, Salamaszynska-Guz, A, Rzewuska, M, Klimuszko, D, Binek, M (2009). Resistance to antimicrobial agents of Campylobacter spp. strains isolated from animals in Poland *Polish Journal of Veterinary Sciences*, 12(4), 465

361. H-J Tsai, H-C, Huang, C-M, Lin, Y-Y, Lien, C-H Chou (2007). Salmonellae and Campylobacters in Household and Stray Dogs in Northern Taiwan *Veterinary Research Communications*, 31(8), 931

362. Tankson, J D, Fedorka-Cray, P J, Jackson, C R, Headrick, M (2006). Genetic relatedness of a rarely isolated Salmonella: Salmonella enterica serotype Niakhar from NARMS animal isolates *The Journal of Antimicrobial Chemotherapy*, 57(2), 190

363. Ganière, Jean-Pierre, Médaille, Christine, Etoré, Florence (2004). In vitro antimicrobial activity of orbifloxacin against Staphylococcus intermedius isolates from canine skin and ear infections *Research in Veterinary Science*, 77(1), 67

364. Bartges, Joseph W (2002). Appropriate use of antimicrobials in cats *Veterinary Medicine, suppl. CE Advisor: Appropriate Use of Antimicrobials in Cats*, #volume#(#issue#), 1

365. White, Stephen D (1996). Systemic treatment of bacterial skin infections of dogs and cats. *Veterinary dermatology*, 7(3), 133

366. Acke, E (2018). Campylobacteriosis in dogs and cats: a review *New Zealand veterinary journal*, 66(5), 221

367. Leite-Martins, L, Mahu, MI, Costa, AL, Bessa, LJ, Vaz-Pires, P, Loureiro, L, Niza-Ribeiro, J, de Matos, AJF, da Costa, PM (2015). Prevalence of antimicrobial resistance in faecal enterococci from vet-visiting pets and assessment of risk factors *VETERINARY RECORD*, 176(26), 674

368. Hardefeldt, LY, Browning, GF, Thursky, K, Gilkerson, JR, Billman-Jacobe, H, Stevenson, MA, Bailey, KE (2017). Antimicrobials used for surgical prophylaxis by companion animal veterinarians in Australia *Veterinary Microbiology*, 203(#issue#), 301

369. Worthing, KA, Brown, J, Gerber, L, Trott, DJ, Abraham, S, Norris, JM (2018). Methicillin-resistant staphylococci amongst veterinary personnel, personnel-owned pets, patients and the hospital environment of two small animal veterinary hospitals *Veterinary Microbiology*, 223(#issue#), 79

370. Miranda, C, Silva, V, Igrejas, G, Poeta, P (2021). Impact of European pet antibiotic use on enterococci and staphylococci antimicrobial resistance and human health *Future Microbiology*, 16(3), #Pages#

371. Valiakos, G, Pavlidou, E, Zafeiridis, C, Tsokana, CN, Vilas, VJD (2020). Antimicrobial practices among small animal veterinarians in Greece: a survey *One Health Outlook*, 2(1), #Pages#

372. Vercelli, C, Gambino, G, Amadori, M, Re, G (2022). Implications of Veterinary Medicine in the comprehension and stewardship of antimicrobial resistance phenomenon. From the origin till nowadays *Veterinary And Animal Science*, 16(#issue#), #Pages#

373. Guardabassi, L, Larsen, J, Weese, JS, Butaye, P, Battisti, A, Kluytmans, J, Lloyd, DH, Skov, RL (2013). Public health impact and antimicrobial selection of meticillin-resistant staphylococci in animals *Journal Of Global Antimicrobial Resistance*, 1(2), 55

374. Kock, R, Daniels-Haardt, I, Becker, K, Mellmann, A, Friedrich, AW, Mevius, D, Schwarz, S, Jurke, A (2018). Carbapenem-resistant Enterobacteriaceae in wildlife, food-producing, and companion animals: a systematic review *Clinical Microbiology And Infection*, 24(12), 1241

375. Moerer, M, Merle, R, Baumer, W (2022). Antibiotic use and development of microbial resistance in dogs and cats under the influence of the TAHAV amendment 2018-a survey

analysis of veterinarians in Berlin *Berliner Und Munchener Tierarztliche Wochenschrift*, 135(#issue#), 1

376. Worthing, KA, Abraham, S, Pang, S, Coombs, GW, Saputra, S, Jordan, D, Wong, HS, Abraham, RJ, Trott, DJ, Norris, JM (2018). Molecular Characterization of Methicillin-Resistant Staphylococcus aureus Isolated from Australian Animals and Veterinarians *Microbial Drug Resistance*, 24(2), 203

377. Joosten, P, Van Cleven, A, Sarrazin, S, Paepe, D, De Sutter, A, Dewulf, J (2020). Dogs and Their Owners Have Frequent and Intensive Contact *International Journal Of Environmental Research And Public Health*, 17(12), #Pages#

378. Johnstone, T (2020). A clinical approach to multidrug-resistant urinary tract infection and subclinical bacteriuria in dogs and cats *NEW Zealand Veterinary Journal*, 68(2), 69

379. Fowler, H, Davis, MA, Perkins, A, Trufan, S, Joy, C, Buswell, M, McElwain, TF, Moore, D, Worhle, R, Rabinowitz, PM (2016). Survey of veterinary antimicrobial prescribing practices, Washington State 2015 *Veterinary Record*, 179(25), 651

380. Guardabassi, L, Fondati, A (2009). Prudent and rational use of antibiotics for treatment of canine and feline pyoderma *Veterinaria*, 23(3), 11

381. Grobbel, M, Lubke-Becker, A, Alesik, E, Schwarz, S, Wallmann, J, Werckenthin, C, Wieler, LH (2007). Antimicrobial susceptibility of Klebsiella spp. and Proteus spp. from various organ systems of horses, dogs and cats as determined in the BfT-GermVet monitoring program 2004-2006 *Berliner Und Munchener Tierarztliche Wochenschrift*, 120(9-10), 402

382. Souza, MM, Bordin, JT, Pavan, ACL, Rodrigues, RGA, Sfaciotte, RAP, Vignoto, VKC, Ferrante, M, Wosiacki, SR (2020). Antimicrobial resistance evaluation of bacteria isolated from infections in small animals in the Umuarama region, Parana *Pesquisa Veterinaria Brasileira*, 40(10), 804

383. Iannino, F, Di Donato, G, Salucci, S, Ruggieri, E, Vincifori, G, Danzetta, ML, Dalla Villa, P, Di Giannatale, E, Lotti, G, De Massis, F (2022). Campylobacter and risk factors associated with dog ownership: a retrospective study in household and shelter dogs *Veterinaria Italiana*, 58(1), 57

384. Marques, C, Menezes, J, Belas, A, Aboim, C, Cavaco-Silva, P, Trigueiro, G, Gama, LT, Pomba, C (2019). Klebsiella pneumoniae causing urinary tract infections in companion animals and humans: population structure, antimicrobial resistance and virulence genes *Journal Of Antimicrobial Chemotherapy*, 74(3), 594

385. Reimschuessel, R, Grabenstein, M, Guag, J, Nemser, SM, Song, K, Qiu, J, Clothier, KA, Byrne, BA, Marks, SL, Cadmus, K, Pabilonia, K, Sanchez, S, Rajeev, S, Ensley, S, Frana, TS, Jergens, AE, Chappell, KH, Thakur, S, Byrum, B, Cui, J, Zhang, Y, Erdman, MM,

Rankin, SC, Daly, R, Das, S, Ruesch, L, Lawhon, SD, Zhang, S, Baszler, T, Diaz-Campos, D, Hartmann, F, Okwumabua, O (2017). Multilaboratory Survey To Evaluate Salmonella Prevalence in Diarrheic and Nondiarrheic Dogs and Cats in the United States between 2012 and 2014 *Journal Of Clinical Microbiology*, 55(5), 1350

386. Hernando, E, Vila, A, D'Ippolito, P, Rico, AJ, Rodon, J, Roura, X (2021). Cats From Spain *Topics In Companion Animal Medicine*, 43(#issue#), #Pages#

387. Baker, SA, Van-Balen, J, Lu, B, Hillier, A, Hoet, AE (2012). Antimicrobial drug use in dogs prior to admission to a veterinary teaching hospital *Javma-Journal Of The American Veterinary Medical Association*, 241(2), 210

388. Marques, C, Belas, A, Aboim, C, Trigueiro, G, Cavaco-Silva, P, Gama, LT, Pomba, C (2019). Clonal relatedness of Proteus mirabilis strains causing urinary tract infections in companion animals and humans *Veterinary Microbiology*, 228(#issue#), 77

389. Frasao, BD, Marin, VA, Conte, CA (2017). Molecular Detection, Typing, and Quantification of Campylobacter spp. in Foods of Animal Origin *Comprehensive Reviews In Food Science And Food Safety*, 16(4), 721

390. Ribeiro, MG, de Morais, ABC, Alves, AC, Bolanos, CAD, de Paula, CL, Portilho, FVR, de Nardi, G, Lara, GHB, Martins, LDA, Moraes, LS, Risseti, RM, Guerra, ST, Bello, TS, Siqueira, AK, Bertolini, AB, Rodrigues, CA, Paschoal, NR, de Almeida, BO, Listoni, FJP, Sanchez, LFG, Paes, AC (2022). Klebsiella-induced infections in domestic species: a case-series study in 697 animals (1997-2019) *Brazilian Journal Of Microbiology*, 53(1), 455

391. Cazedey, ECL, Salgado, HRN (2013). Orbifloxacin: A Review of Properties, Its Antibacterial Activities, Pharmacokinetic/Pharmacodynamic Characteristics, Therapeutic Use, and Analytical Methods *Critical Reviews In Analytical Chemistry*, 43(2), 79

Level 1, Form level_1_screening, Does the title/abstract describe antimicrobial resistance? -> No

392. Canil, C, Rosenshine, I, Ruschkowski, S, Donnenberg, M S, Kaper, J B, Finlay, B B (1993). Enteropathogenic Escherichia coli decreases the transepithelial electrical resistance of polarized epithelial monolayers. *Infection and immunity*, 61(7), 2755

393. EA Casarez, SD Pillai, JB Mott, M Vargas, KE Dean, GD Di Giovanni (2007). Direct comparison of four bacterial source tracking methods and use of composite data sets. *Journal of applied microbiology*, 103(2), #Pages#

394. Shaheen, Bashar W, Boothe, Dawn M, Oyarzabal, Omar A, Wang, Chengming, Johnson, Calvin M (2011). Evaluation of the contribution of gyrA mutation and efflux pumps to fluoroquinolone and multidrug resistance in pathogenic Escherichia coli isolates from dogs

and cats. *American journal of veterinary research*, 72(1), 25

395. Sykes, Jane E, Blondeau, Joseph M (2014). Pradofloxacin: a novel veterinary fluoroquinolone for treatment of bacterial infections in cats. *Veterinary journal (London, England : 1997)*, 201(2), 207

396. ZT Kern, ME Jacob, JM Gilbertie, SL Vaden, SK Lyle (2018). Characteristics of Dogs with Biofilm-Forming Escherichia Coli Urinary Tract Infections. *Journal of veterinary internal medicine*, 32(5), #Pages#

397. J Li, Z Bi, S Ma, B Chen, C Cai, J He, S Schwarz, C Sun, Y Zhou, J Yin, A Hulth, Y Wang, Z Shen, S Wang, C Wu, LE Nilsson, TR Walsh, S Börjesson, J Shen, Q Sun, Y Wang (2019). Inter-host Transmission of Carbapenemase-Producing <i>Escherichia coli</i> among Humans and Backyard Animals. *Environmental health perspectives*, 127(10), #Pages#

398. RW Williams, S Cole, DE Holt (2020). Microorganisms associated with incisional infections after gastrointestinal surgery in dogs and cats. *Veterinary surgery : VS*, 49(7), #Pages#

399. AK Kidsley, M O'Dea, S Saputra, D Jordan, JR Johnson, DM Gordon, C Turni, SP Djordjevic, S Abraham, DJ Trott (2020). Genomic analysis of phylogenetic group B2 extraintestinal pathogenic E. coli causing infections in dogs in Australia. *Veterinary microbiology*, 248(), #Pages#

400. DC Moon, JH Choi, N Boby, SJ Kim, HJ Song, HS Park, MC Gil, SS Yoon, SK Lim (2022). Prevalence of Bacterial Species in Skin, Urine, Diarrheal Stool, and Respiratory Samples in Cats. *Pathogens (Basel, Switzerland)*, 11(3), #Pages#

401. S Perestrelo, G Correia Carreira, L Valentin, J Fischer, Y Pfeifer, G Werner, J Schmiedel, L Falgenhauer, C Imirzalioglu, T Chakraborty, A Käsbohrer (2022). Comparison of approaches for source attribution of ESBL-producing Escherichia coli in Germany. *PloS one*, 17(7), #Pages#

402. S Perestrelo, A Amaro, MSM Brouwer, L Clemente, AS Ribeiro Duarte, A Kaesbohrer, R Karpíšková, V Lopez-Chavarrias, D Morris, D Prendergast, A Pista, L Silveira, M Skarżyńska, R Slowey, KT Veldman, M Zając, C Burgess, J Alvarez (2023). Building an International One Health Strain Level Database to Characterise the Epidemiology of AMR Threats: ESBL-AmpC Producing <i>E. coli</i> as An Example-Challenges and Perspectives. *Antibiotics (Basel, Switzerland)*, 12(3), #Pages#

403. Williams, R. W., Cole, S., Holt, D. E. (2020). Microorganisms associated with incisional infections after gastrointestinal surgery in dogs and cats. *Veterinary Surgery*, 49(7), 1301

404. Rafael Gariglio Clark Xavier, Paloma Helena Sanches da Silva, Hanna Dornelas Trindade, Gabriela Muniz Carvalho, Rafael Romero Nicolino, Patrícia Maria Coletto Freitas,

Silveira Silva, Rodrigo Otávio (2022). Characterization of Escherichia coli in Dogs with Pyometra and the Influence of Diet on the Intestinal Colonization of Extraintestinal Pathogenic E. coli (ExPEC) *Veterinary Sciences*, 9(5), 245

405. Razali, Kahina, Kaidi, Rachid, Abdelli, Amine, Menoueri, Mohamed Nabil, Ait-Oudhia, Khatima (2020). Oral flora of stray dogs and cats in Algeria: Pasteurella and other zoonotic bacteria *Veterinary World*, 13(12), 2806

406. Carolina Lechinski de Paula, Silveira Silva, Rodrigo Otávio, Hernandes, Rodrigo Tavanelli, Geraldo de Nardi Júnior, Babboni, Selene Daniela, Guerra, Simony Trevizan, Fernando José Paganini Listoni, Giuffrida, Rogério, Takai, Shinji, Sasaki, Yukako, Márcio Garcia Ribeiro (2019). First Microbiological and Molecular Identification of Rhodococcus equi in Feces of Nondiarrheic Cats *BioMed Research International*, 2019(#issue#), 9

407. Dogan, Belgin, Hannah Facey Belcher-Timme, Dogan, Esra I, Zhi-Dong, Jiang, DuPont, Herbert L, Snyder, Ned, Yang, Stephen, Chandler, Brendan, Scherl, Ellen J, Simpson, Kenneth W (2018). Evaluation of Escherichia coli pathotypes associated with irritable bowel syndrome *FEMS Microbiology Letters*, 365(22), #Pages#

408. Moon, Christina D, Young, Wayne, Maclean, Paul H, Cookson, Adrian L, Bermingham, Emma N (2018). Metagenomic insights into the roles of Proteobacteria in the gastrointestinal microbiomes of healthy dogs and cats *Microbiology Open*, 7(5), #Pages#

409. Punia, Manisha, Kumar, Ashok, Charaya, Gaurav, Kumar, Tarun (2018). Pathogens isolated from clinical cases of urinary tract infection in dogs and their antibiogram *Veterinary World*, 11(8), 1037

410. Damborg, Peter, Gumpert, Heidi, Johansson, Laura, Jana, Bimal, Niels Frimodt-M□□ller, Guardabassi, Luca (2018). Dogs as reservoirs of Escherichia coli strains causing urinary tract infection in their owners *bioRxiv*, #volume#(#issue#), #Pages#

411. Chanchaithong, Pattrarat, Ritthikulprasert, Sukullaya (2018). Prevalence of bacteremia in dogs admitted to an intensive care unit with intravenous catheterization *The Thai Journal of Veterinary Medicine*, 48(1), 123

412. Kireewan, Supapatt, Suanpairintr, Nipattra (2017). Comparative in vitro killing activities of enrofloxacin when used alone and in combination with doxycycline against E. coli isolates from dogs and cats *The Thai Journal of Veterinary Medicine*, 47(2), 217

413. Damborg, P, Nielsen, S S, Guardabassi, L (2009). Escherichia coli shedding patterns in humans and dogs: insights into within-household transmission of phylotypes associated with urinary tract infections *Epidemiology and Infection*, 137(10), 1457

414. Féria, Constança, Machado, Jorge, José Duarte Correia, Gonçalves, José, Gaastra, Wim (2001). Distribution of papG alleles among uropathogenic Escherichia coli isolated from

different species *FEMS Microbiology Letters*, 202(2), 205

415. Thornton, LA, Burchell, RK, Burton, SE, Lopez-Villalobos, N, Pereira, D, MacEwan, I, Fang, C, Hatmodjo, AC, Nelson, MA, Grinberg, A, Velathanthiri, N, Gal, A (2018). The Effect of Urine Concentration and pH on the Growth of Escherichia Coli in Canine Urine In Vitro *Journal Of Veterinary Internal Medicine*, 32(2), 752

416. Shimizu, T, Harada, K (2017). Determination of minimum biofilm eradication concentrations of orbifloxacin for canine bacterial uropathogens over different treatment periods *Microbiology And Immunology*, 61(1), 17

Level 1, Form level_1_screening, Was the study a primary research article? -> No. It's a narrative review

417. Guardabassi, Luca, Schwarz, Stefan, Lloyd, David H (2004). Pet animals as reservoirs of antimicrobial-resistant bacteria. *The Journal of antimicrobial chemotherapy*, 54(2), 321

418. Heim, D, Kuster, SP, Willi, B (2020). Antibiotic-resistant bacteria in dogs and cats: recommendations for owners *Schweizer Archiv Fur Tierheilkunde*, 162(3), 141

419. AR Manges, JR Johnson (2015). Reservoirs of Extraintestinal Pathogenic Escherichia coli. *Microbiology spectrum*, 3(5), #Pages#

420. B Walther, K Tedin, A Lübke-Becker (2017). Multidrug-resistant opportunistic pathogens challenging veterinary infection control. *Veterinary microbiology*, 200(), #Pages#

421. Gronthal, Thomas, Osterblad, Monica, Eklund, Marjut, Jalava, Jari, Nykasenoja, Suvi, Pekkanen, Katariina, Rantala, Merja (2018). Sharing more than friendship - transmission of NDM-5 ST167 and CTX-M-9 ST69 Escherichia coli between dogs and humans in a family, Finland, 2015. *Euro surveillance : bulletin Europeen sur les maladies transmissibles = European communicable disease bulletin*, 23(27), #Pages#

422. FP Sellera, N Lincopan (2019). Zooanthroponotic transmission of high-risk multidrug-resistant pathogens: A neglected public health issue. *Journal of infection and public health*, 12(2), #Pages#

423. ME Reynolds, HTT Phan, S George, ATM Hubbard, N Stoesser, IE Maciuca, DW Crook, D Timofte (2019). Occurrence and characterization of Escherichia coli ST410 co-harbouring blaNDM-5, blaCMY-42 and blaTEM-190 in a dog from the UK. *The Journal of antimicrobial chemotherapy*, 74(5), #Pages#

424. J Harrer, R Dorsch (2020). [Bacterial urinary tract infection and subclinical bacteriuria in the dog: a current review]. *Tierarztliche Praxis. Ausgabe K, Kleintiere/Heimtiere*, 48(4),

#Pages#

425. N Puvača, R de Llanos Frutos (2021). Antimicrobial Resistance in <i>Escherichia coli</i> Strains Isolated from Humans and Pet Animals. *Antibiotics (Basel, Switzerland),* 10(1), #Pages#

426. Hong JunSung, Song WonKeun, Jeong SeokHoon (2020). Molecular characteristics of NDM-5-producing Escherichia coli from a cat and a dog in South Korea. *Microbial Drug Resistance*, 26(8), 1005

427. Nielsen, S. S., Bicout, D. J., Calistri, P., Canali, E., Drewe, J. A., Garin-Bastuji, B., Rojas, J. L. G., Schmidt, C. G., Herskin, M., Michel, V., Chueca, M. A. M., Padalino, B., Pasquali, P., Roberts, H. C., Sihvonen, L. H., Spoolder, H., Stahl, K., Velarde, A., Viltrop, A., Winckler, C., Guardabassi, L., Hilbert, F., Mader, R., Aznar, I., Baldinelli, F., Alvarez, J. (2021). Assessment of animal diseases caused by bacteria resistant to antimicrobials: dogs and cats. *EFSA Journal*, 19(6), #Pages#

428. Soares, M. T., Nakazato, G., Kobayashi, R. K. T., Zanutto, M. de S. (2022). Escherichia coli in dogs and cats and its zoonotic potential - a review. *Clínica Veterinária*, 27(156), 32

429. Haast, A. de (2017). Antibiotic resistance. *9th Veterinary, Paraveterinary & SASVEPM (South African Society for Veterinary Epidemiology and Preventive Medicine) Congress 2017, Boksburg, South Africa, 24-27 July 2017*, #volume#(#issue#), 151

430. Davies, R. H., Lawes, J. R., Wales, A. D. (2019). Raw diets for dogs and cats: a review, with particular reference to microbiological hazards. *Journal of Small Animal Practice*, 60(6), 329

431. Walther, B., Tedin, K., Lübke-Becker, A. (2017). Multidrug-resistant opportunistic pathogens challenging veterinary infection control. *Veterinary Microbiology*, 200(#issue#), 71

432. Harada, K. (2015). Epidemiology of antimicrobial-resistant bacteria isolated from companion animals in Japan. *Journal of Veterinary Epidemiology*, 19(2), 85

433. Guardabassi, L. (2013). Sixty years of antimicrobial use in animals: what is next? *Veterinary Record*, 173(24), 599

434. Perestrelo, S., Amaro, A., Brouwer, M. S. M., Clemente, L., Duarte, A. S. R., Kaesbohrer, A., Karpíšková, R., Lopez-Chavarrias, V., Morris, D., Prendergast, D., Pista, A., Silveira, L., Skarżyńska, M., Slowey, R., Veldman, K. T., Zając, M., Burgess, C., Alvarez, J. (2023). Building an international one health strain level database to characterise the epidemiology of AMR threats: ESBL - AmpC producing E. coli as an example - challenges and perspectives. *Antibiotics*, 12(3), #Pages#

435. Reynolds, M. E., Phan, H. T. T., George, S., Hubbard, A. T. M., Stoesser, N., Maciuca, I. E., Crook, D. W., Timofte, D. (2019). Occurrence and characterization of Escherichia coli ST410 co-harbouring blaNDM-5, blaCMY-42 and blaTEM-190 in a dog from the UK. *Journal of Antimicrobial Chemotherapy*, 74(5), 1207

436. Truszczyński, M., Pejsak, Z. (2013). Development of antibiotics resistance of zoonotic microorganism. *Życie Weterynaryjne*, 88(7), 535

437. Sastry, S., Doi YoHei (2016). Fosfomycin: resurgence of an old companion. *Journal of Infection and Chemotherapy*, 22(5), 273

438. Grudlewska-Buda, Katarzyna, Bauza-Kaszewska, Justyna, Wiktorczyk-Kapischke, Natalia, Budzyńska, Anna, Gospodarek-Komkowska, Eugenia, Skowron, Krzysztof (2023). Antibiotic Resistance in Selected Emerging Bacterial Foodborne Pathogens—An Issue of Concern? *Antibiotics*, 12(5), 880

439. Caneschi, Alice, Bardhi, Anisa, Barbarossa, Andrea, Zaghini, Anna (2023). The Use of Antibiotics and Antimicrobial Resistance in Veterinary Medicine, a Complex Phenomenon: A Narrative Review *Antibiotics*, 12(3), 487

440. Xu, Chunming, Kong, Lingqiang, Liao, Yonghong, Tian, Yuan, Wu, Qi, Liu, Haosi, Wang, Xiumin (2022). Mini-Review: Antibiotic-Resistant Escherichia coli from Farm Animal-Associated Sources *Antibiotics*, 11(11), 1535

441. Urban-Chmiel, Renata, Marek, Agnieszka, Stępień-Pyśniak, Dagmara, Wieczorek, Kinga, Dec, Marta, Nowaczek, Anna, Osek, Jacek (2022). Antibiotic Resistance in Bacteria—A Review *Antibiotics*, 11(8), 1079

442. Pattis, Isabelle, Weaver, Louise, Burgess, Sara, Ussher, James E, Dyet, Kristin (2022). Antimicrobial Resistance in New Zealand—A One Health Perspective *Antibiotics*, 11(6), 778

443. Monteiro, Kadja Luana Chagas, Silva, Osmar Nascimento, Dos Santos Nascimento, Igor José, Mendonça Júnior, Francisco Jaime Bezerra, Aquino, Pedro Gregório Vieira, da Silva-Júnior, Edeildo Ferreira, de Aquino, Thiago Mendonça (2022). Medicinal Chemistry of Inhibitors Targeting Resistant Bacteria. *Current topics in medicinal chemistry*, 22(24), 1983

444. Singleton, David A, Rayner, Angela, Brant, Bethaney, Smyth, Steven, Noble, Peter-John M, Radford, Alan D, Pinchbeck, Gina L (2021). A randomised controlled trial to reduce highest priority critically important antimicrobial prescription in companion animals. *Nature communications*, 12(1), 1593

445. Puvača, Nikola, Rosa de Llanos Frutos (2021). Antimicrobial Resistance in Escherichia coli Strains Isolated from Humans and Pet Animals *Antibiotics*, 10(1), 69

446. Ewers, C, Grobbel, M, Bethe, A, Wieler, LH, Guenther, S (2011). Extended-spectrum beta-lactamases-producing Gram-negative bacteria in companion animals: action is clearly

warranted! *Berliner Und Munchener Tierarztliche Wochenschrift*, 124(3-4), 94

447. Smith, JL, Fratamico, PM, Gunther, NW (2007). Extraintestinal pathogenic Escherichia coli *Foodborne Pathogens And Disease*, 4(2), 134

448. Thompson, MF, Litster, AL, Platell, JL, Trott, DJ (2011). Canine bacterial urinary tract infections: New developments in old pathogens *Veterinary Journal*, 190(1), 22

449. Belmar-Liberato, R, Gonzalez-Canga, A, Tamame-Martin, P, Escribano-Salazar, M (2011). Amoxicillin and amoxicillin-clavulanic acid resistance in veterinary medicine - the situation in Europe: a review *Veterinarni Medicina*, 56(10), 473

Level 1, Form level_1_screening, Was the study a primary research article? -> No. It's a scoping review

450. Bhat, Aashaq Hussain (2021). Bacterial zoonoses transmitted by household pets and as reservoirs of antimicrobial resistant bacteria. *Microbial pathogenesis*, 155(#issue#), 104891

451. Guardabassi, Luca, Schwarz, Stefan, Lloyd, David H (2004). Pet animals as reservoirs of antimicrobial-resistant bacteria: Review *The Journal of Antimicrobial Chemotherapy*, 54(2), 321

Level 1, Form level_1_screening, Was the study a primary research article? -> No. It's a meta-analysis

452. AB Yaovi, P Sessou, ABN Tonouhewa, GYM Hounmanou, D Thomson, R Pelle, S Farougou, A Mitra (2022). Prevalence of antibiotic-resistant bacteria amongst dogs in Africa: A meta-analysis review. *The Onderstepoort journal of veterinary research*, 89(1), #Pages#

453. Yaovi, Ayaovi B, Sessou, Philippe, Tonouhewa, Aretas B.N., Hounmanou, Gildas Y.M., Thomson, Deborah, Pelle, Roger, Farougou, Souaïbou, Mitra, Arindam (2022). Prevalence of antibiotic-resistant bacteria amongst dogs in Africa: A meta-analysis review *The Onderstepoort Journal of Veterinary Research*, 89(1), #Pages#

Level 1, Form level_1_screening, Was the study a primary research article? -> No. It's both a systematic review and meta-analysis

454. AK Castillo, K Espinoza, AF Chaves, F Guibert, J Ruiz, MJ Pons (2022). Antibiotic susceptibility among non-clinical <i>Escherichia coli</i> as a marker of antibiotic pressure in Peru (2009-2019): one health approach. *Heliyon*, 8(9), #Pages#

455. DC Sebola, JW Oguttu, MM Kock, DN Qekwana (2022). Hospital-acquired and zoonotic bacteria from a veterinary hospital and their associated antimicrobial-susceptibility profiles: A systematic review. *Frontiers in veterinary science*, 9(), #Pages#

456. M Lysitsas, I Chatzipanagiotidou, C Billinis, G Valiakos (2023). Fosfomycin Resistance in Bacteria Isolated from Companion Animals (Dogs and Cats). *Veterinary sciences*, 10(5), #Pages#

457. Sebola, D. C., Oguttu, J. W., Kock, M. M., Qekwana, D. N. (2023). Hospital-acquired and zoonotic bacteria from a veterinary hospital and their associated antimicrobial-susceptibility profiles: a systematic review. *Frontiers in Veterinary Science*, 9(January), #Pages#

458. Rodrigues, Inês C, Rodrigues, Sílvia C, Duarte, Filipe V, da Costa, Paula M, da Costa, Paulo M (2022). The Role of Outer Membrane Proteins in UPEC Antimicrobial Resistance: A Systematic Review *Membranes*, 12(10), 981

459. Hamame, Afaf, Davoust, Bernard, Cherak, Zineb, Rolain, Jean-Marc, Diene, Seydina M (2022). Mobile Colistin Resistance (mcr) Genes in Cats and Dogs and Their Zoonotic Transmission Risks *Pathogens*, 11(6), 698

460. Shahneaz Ali Khan, Mohammed Ashif Imtiaz. Abu Sayeed, Amir Hossan Shaikat, Hassan, Mohammad Mahmudul (2020). Antimicrobial resistance pattern in domestic animal - wildlife - environmental niche via the food chain to humans with a Bangladesh perspective; a systematic review *BMC Veterinary Research*, 16(#issue#), 1

461. Rincon-Real, AA, Suarez-Alfonso, MC (2022). Carbapenem resistance in critically important human pathogens isolated from companion animals: a systematic literature review *Osong Public Health And Research Perspectives*, 13(6), 407

462. Hackmann, C, Gastmeier, P, Schwarz, S, Lubke-Becker, A, Bischoff, P, Leistner, R (2021). Pet husbandry as a risk factor for colonization or infection with MDR organisms: a systematic meta-analysis *Journal Of Antimicrobial Chemotherapy*, 76(6), 1392

Level 1, Form level_1_screening, Was the study a primary research article? -> No. It's both a scoping review and meta-analysis

463. Salgado-Caxito, M., Benavides, J. A., Adell, A. D., Paes, A. C., Moreno-Switt, A. I. (2021). Global prevalence and molecular characterization of extended-spectrum β-lactamase

producing-Escherichia coli in dogs and cats - a scoping review and meta-analysis. *One Health*, 12(#issue#), #Pages#

Level 1, Form level_1_screening, Was the study a primary research article? -> No. It's another study (e.g., conference proceedings, commentary, editorial, letter-to-the-editor, news reports, expert opinions)

464. Goering, Richard V (2002). Questions study on Escherichia coli susceptibility. *Journal of the American Veterinary Medical Association*, 220(8), 1139

465. Maddox, Thomas W, Wedley, Amy L, Dawson, Susan, Clegg, Peter, Pinchbeck, Gina L, Nuttall, Tim, Williams, Nicola J (2008). Antimicrobial resistance in dogs and horses. *The Veterinary record*, 162(2), 63

466. Chan, Jane, Lo, Wai-U, Chow, Kin-Hung, Lai, Eileen L, Law, Pierra Y, Ho, Pak-Leung (2014). Clonal diversity of Escherichia coli isolates carrying plasmid-mediated fosfomycin resistance gene fosA3 from livestock and other animals. *Antimicrobial agents and chemotherapy*, 58(9), 5638

467. Papich, Mark G (2016). Antimicrobial susceptibility testing for feline urinary tract isolates. *Journal of feline medicine and surgery*, 18(2), 183

468. J Pires, OJ Bernasconi, S Kasraian, M Hilty, V Perreten, A Endimiani (2016). Intestinal colonisation with extended-spectrum cephalosporin-resistant Escherichia coli in Swiss pets: molecular features, risk factors and transmission with owners. *International journal of antimicrobial agents*, 48(6), #Pages#

469. H Ghosh, S Doijad, L Falgenhauer, M Fritzenwanker, C Imirzalioglu, T Chakraborty (2017). bla-Encoding Escherichia coli Sequence Type 131 Lineage C1-M27 Clone in Clinical Isolates, Germany. *Emerging infectious diseases*, 23(10), #Pages#

470. SS Nielsen, DJ Bicout, P Calistri, E Canali, JA Drewe, B Garin-Bastuji, JL Gonzales Rojas, C Gortazar Schmidt, M Herskin, V Michel, MA Miranda Chueca, B Padalino, P Pasquali, HC Roberts, LH Sihvonen, H Spoolder, K Stahl, A Velarde, A Viltrop, C Winckler, L Guardabassi, F Hilbert, R Mader, I Aznar, F Baldinelli, J Alvarez (2021). Assessment of animal diseases caused by bacteria resistant to antimicrobials: Dogs and cats. *EFSA journal. European Food Safety Authority*, 19(6), #Pages#

471. ACVIM Forum On Demand Research Report Program. (2020). *Journal of veterinary internal medicine*, *34*(6), 2817–3166. https://doi.org/10.1111/jvim.15903

472. Salvarani, S., Tramuta, C., Nebbia, P., Robino, P. (2011). Identification and characterization of β-lactamase producing strains of Escherichia coli isolated from dogs and

cats with cystitis in the province of Turin [Conference poster]. *XIII Congresso Nazionale S.I.Di.L.V., Trani, Italia, 12-14 ottobre 2011*, #volume#(#issue#), 366

473. Viana, T. D. C., Santos, N. M. O. dos, Lima, A. L. S. de, Monteiro, T. R. M., Espinheiro, R. de F., Dias, H. L. T. (2012). Vaginal bacterial microbiota study of domestic dogs and cats. *Archives of Veterinary Science*, 17(Suplemento), 233

474. Dawson, S. (2011). Antimicrobial resistance in pets: identifying key issues. *Veterinary Times*, 41(14), 18

475. Haraldsson, J. (2013). Njurvolym samt relation mellan bark och märg mätt med ultraljud hos katt.

476. Grimes, M., & Lidbury, J. (2020). Antibiotics in canine GI disease: When to use and when to ditch.

477. Worsley-Tonks, Katherine E.L. (2020). Ecology and Epidemiology of Antimicrobial Resistance in Urban and Peri-Urban Mesocarnivores *#journal#*, #volume#(#issue#), 164

478. Taylor, Daniel Davies (2020). Antimicrobial Drug Use and Antimicrobial Resistance in Companion Animal Medicine *#journal#*, #volume#(#issue#), 181

479. Murphy, Colleen P (2010). Antimicrobial resistance, antimicrobial use and infection control in community small animal veterinary hospitals in southern Ontario. *#journal#*, #volume#(#issue#), 1

480. Costa, Daniela, Poeta, Patricia, Briñas, Laura, Sáenz, Yolanda, Rodrigues, Jorge, Torres, Carmen (2004). Detection of CTX-M-1 and TEM-52 ²-lactamases in Escherichia coli strains from healthy pets in Portugal *The Journal of Antimicrobial Chemotherapy*, 54(5), 960

Level 1, Form level_1_screening, What was the study type? -> Descriptive studies (including case report or case series)

481. Jang, S S, Breher, J E, Dabaco, L A, Hirsh, D C (1997). Organisms isolated from dogs and cats with anaerobic infections and susceptibility to selected antimicrobial agents. *Journal of the American Veterinary Medical Association*, 210(11), 1610

482. Chan, O. S. K., Baranger-Ete, M., Lam, W. W. T., Wu Peng, Yeung, M., Lee, E., Bond, H., Swan, O., Tun HeinMin (2022). A retrospective study of antimicrobial resistant bacteria associated with feline and canine urinary tract infection in Hong Kong SAR, China - a case study on implication of first-line antibiotics use. *Antibiotics*, 11(9), #Pages#

483. Normand, E H, Gibson, N R, Reid, S W, Carmichael, S, Taylor, D J (2000). Antimicrobial-resistance trends in bacterial isolates from companion-animal community practice in the UK. *Preventive veterinary medicine*, 46(4), 267

484. Warren, A, Townsend, K, King, T, Moss, S, O'Boyle, D, Yates, R, Trott, D J (2001). Multi-drug resistant escherichia coli with extended-spectrum beta-lactamase activity and fluoroquinolone resistance isolated from clinical infections in dogs. *Australian veterinary journal*, 79(9), 621

485. RG Lobetti, KE Joubert, J Picard, J Carstens, E Pretorius (2002). Bacterial colonization of intravenous catheters in young dogs suspected to have parvoviral enteritis. *Journal of the American Veterinary Medical Association*, 220(9), #Pages#

486. Meunier, D, Acar, J-F, Martel, J-L, Kroemer, S, Valle, M (2004). A seven-year survey of susceptibility to marbofloxacin of pathogenic strains isolated from pets. *International journal of antimicrobial agents*, 24(6), 592

487. Hagman, R, Greko, C (2005). Antimicrobial resistance in Escherichia coli isolated from bitches with pyometra and from urine samples from other dogs. *The Veterinary record*, 157(7), 193

488. MR Stegemann, CA Passmore, J Sherington, CJ Lindeman, G Papp, DJ Weigel, TL Skogerboe (2006). Antimicrobial activity and spectrum of cefovecin, a new extended-spectrum cephalosporin, against pathogens collected from dogs and cats in Europe and North America. *Antimicrobial agents and chemotherapy*, 50(7), #Pages#

489. Radice, Mirko, Martino, Piera Anna, Reiter, Alexander M (2006). Evaluation of subgingival bacteria in the dog and susceptibility to commonly used antibiotics. *Journal of veterinary dentistry*, 23(4), 219

490. Lin, C-T, Petersen-Jones, S M (2007). Antibiotic susceptibility of bacterial isolates from corneal ulcers of dogs in Taiwan. *The Journal of small animal practice*, 48(5), 271

491. Gibson, J S, Morton, J M, Cobbold, R N, Sidjabat, H E, Filippich, L J, Trott, D J (2008). Multidrug-resistant E. coli and enterobacter extraintestinal infection in 37 dogs. *Journal of veterinary internal medicine*, 22(4), 844

492. Black, Dorothy M, Rankin, Shelley C, King, Lesley G (2009). Antimicrobial therapy and aerobic bacteriologic culture patterns in canine intensive care unit patients: 74 dogs (January-June 2006). *Journal of veterinary emergency and critical care (San Antonio, Texas. : 2001)*, 19(5), 489

493. Zamankhan Malayeri, Hamed, Jamshidi, Shahram, Zahraei Salehi, Taghi (2010). Identification and antimicrobial susceptibility patterns of bacteria causing otitis externa in dogs. *Veterinary research communications*, 34(5), 435

494. Gebru, Elias, Damte, Dereje, Choi, Myung-Jin, Lee, Seung-Jin, Kim, Young-Hoan, Park, Seung Chun (2012). Mutant prevention concentration and phenotypic and molecular basis of fluoroquinolone resistance in clinical isolates and in vitro-selected mutants of Escherichia

coli from dogs. *Veterinary microbiology*, 154(3-4), 384

495. EG Awji, D Damte, SJ Lee, JS Lee, YH Kim, SC Park (2012). The in vitro activity of 15 antimicrobial agents against bacterial isolates from dogs. *The Journal of veterinary medical science*, 74(8), #Pages#

496. Schink, Anne-Kathrin, Kadlec, Kristina, Hauschild, Tomasz, Brenner Michael, Geovana, Dorner, Julia C, Ludwig, Carolin, Werckenthin, Christiane, Hehnen, Hans-Robert, Stephan, Bernd, Schwarz, Stefan (2013). Susceptibility of canine and feline bacterial pathogens to pradofloxacin and comparison with other fluoroquinolones approved for companion animals. *Veterinary microbiology*, 162(1), 119

497. Bugden, D L (2013). Identification and antibiotic susceptibility of bacterial isolates from dogs with otitis externa in Australia. *Australian veterinary journal*, 91(1-2), 43

498. AB Bennett, PA Martin, SA Gottlieb, M Govendir (2013). In vitro susceptibilities of feline and canine Escherichia coli and Pseudomonas spp. isolates to ticarcillin and ticarcillin-clavulanic acid. *Australian veterinary journal*, 91(5), #Pages#

499. E Hamilton, JM Kruger, W Schall, M Beal, SD Manning, JB Kaneene (2013). Acquisition and persistence of antimicrobial-resistant bacteria isolated from dogs and cats admitted to a veterinary teaching hospital. *Journal of the American Veterinary Medical Association*, 243(7), #Pages#

500. Osugui, L, de Castro, A F Pestana, Iovine, R, Irino, K, Carvalho, V M (2014). Virulence genotypes, antibiotic resistance and the phylogenetic background of extraintestinal pathogenic Escherichia coli isolated from urinary tract infections of dogs and cats in Brazil. *Veterinary microbiology*, 171(1-2), 242

501. Lund, Heidi Sjetne, Skogtun, Gaute, Sorum, Henning, Eggertsdottir, Anna Vigdis (2015). Antimicrobial susceptibility in bacterial isolates from Norwegian cats with lower urinary tract disease. *Journal of feline medicine and surgery*, 17(6), 507

502. Siqueira, E G M, Rahal, S C, Ribeiro, M G, Paes, A C, Listoni, F P, Vassalo, F G (2014). Exogenous bacterial osteomyelitis in 52 dogs: a retrospective study of etiology and in vitro antimicrobial susceptibility profile (2000-2013). *The veterinary quarterly*, 34(4), 201

503. Rheinwald, M, Hartmann, K, Hahner, M, Wolf, G, Straubinger, R K, Schulz, B (2015). Antibiotic susceptibility of bacterial isolates from 502 dogs with respiratory signs. *The Veterinary record*, 176(14), 357

504. B Walther, A Lübke-Becker, I Stamm, H Gehlen, AK Barton, T Janssen, LH Wieler, S Guenther (2014). Suspected nosocomial infections with multi-drug resistant E. coli, including extended-spectrum beta-lactamase (ESBL)-producing strains, in an equine clinic. *Berliner und Munchener tierarztliche Wochenschrift*, 127(11-12), #Pages#

505. Tysnes, Kristoffer Relling, Luyckx, Katrien, Cantas, Leon, Robertson, Lucy J (2016). Treatment of feline giardiasis during an outbreak of diarrhoea in a cattery: potential effects on faecal Escherichia coli resistance patterns. *Journal of feline medicine and surgery*, 18(8), 679

506. Thungrat, Kamoltip, Price, Stuart B, Carpenter, D Mark, Boothe, Dawn Merton (2015). Antimicrobial susceptibility patterns of clinical Escherichia coli isolates from dogs and cats in the United States: January 2008 through January 2013. *Veterinary microbiology*, 179(3-4), 287

507. Dorsch, Roswitha, von Vopelius-Feldt, Clara, Wolf, Georg, Mueller, Ralf S, Straubinger, Reinhard K, Hartmann, Katrin (2016). Urinary tract infections in cats. Prevalence of comorbidities and bacterial species, and determination of antimicrobial susceptibility to commonly used antimicrobial agents. *Tierarztliche Praxis. Ausgabe K, Kleintiere/Heimtiere*, 44(4), 227

508. Brloznik, Maja, Sterk, Karmen, Zdovc, Irena (2016). Prevalence and resistance patterns of canine uropathogens in regard to concurrent diseases. *Berliner und Munchener tierarztliche Wochenschrift*, 129(7-8), 340

509. Marques, Catia, Gama, Luis Telo, Belas, Adriana, Bergstrom, Karin, Beurlet, Stephanie, Briend-Marchal, Alexandra, Broens, Els M, Costa, Marta, Criel, Delphine, Damborg, Peter, van Dijk, Marloes A M, van Dongen, Astrid M, Dorsch, Roswitha, Espada, Carmen Martin, Gerber, Bernhard, Kritsepi-Konstantinou, Maria, Loncaric, Igor, Mion, Domenico, Misic, Dusan, Movilla, Rebeca, Overesch, Gudrun, Perreten, Vincent, Roura, Xavier, Steenbergen, Joachim, Timofte, Dorina, Wolf, Georg, Zanoni, Renato Giulio, Schmitt, Sarah, Guardabassi, Luca, Pomba, Constanca (2016). European multicenter study on antimicrobial resistance in bacteria isolated from companion animal urinary tract infections. *BMC veterinary research*, 12(1), 213

510. Marques, Catia, Belas, Adriana, Franco, Andreia, Aboim, Catarina, Gama, Luis Telo, Pomba, Constanca (2018). Increase in antimicrobial resistance and emergence of major international high-risk clonal lineages in dogs and cats with urinary tract infection: 16 year retrospective study. *The Journal of antimicrobial chemotherapy*, 73(2), 377

511. Y Tsuyuki, G Kurita, Y Murata, T Takahashi, (2018). Bacteria isolated from companion animals in Japan (2014-2016) by blood culture. *Journal of infection and chemotherapy : official journal of the Japan Society of Chemotherapy*, 24(7), #Pages#

512. SHP Hartantyo, ML Chau, L Fillon, AZBM Ariff, JSL Kang, KT Aung, RA Gutiérrez (2018). Sick pets as potential reservoirs of antibiotic-resistant bacteria in Singapore. *Antimicrobial resistance and infection control,* 7(), #Pages#

513. Kurita, Goro, Tsuyuki, Yuzo, Murata, Yoshiteru, Takahashi, Takashi (2019). Reduced rates of antimicrobial resistance in Staphylococcus intermedius group and Escherichia coli

isolated from diseased companion animals in an animal hospital after restriction of antimicrobial use. *Journal of infection and chemotherapy : official journal of the Japan Society of Chemotherapy*, 25(7), 531

514. Roberts, Madeleine, White, Joanna, Lam, Amy (2019). Prevalence of bacteria and changes in trends in antimicrobial resistance of Escherichia coli isolated from positive canine urinary samples from an Australian referral hospital over a 5-year period (2013-2017). *Veterinary record open*, 6(1), e000345

515. Yu, Zhuoling, Wang, Yao, Chen, Yanyun, Huang, Min, Wang, Yang, Shen, Zhangqi, Xia, Zhaofei, Li, Gebin (2020). Antimicrobial resistance of bacterial pathogens isolated from canine urinary tract infections. *Veterinary microbiology*, 241(#issue#), 108540

516. Gilbertie, Jessica M, Levent, Gizem, Norman, Keri N, Vinasco, Javier, Scott, H Morgan, Jacob, Megan E (2020). Comprehensive phenotypic and genotypic characterization and comparison of virulence, biofilm, and antimicrobial resistance in urinary Escherichia coli isolated from canines. *Veterinary microbiology*, 249(#issue#), 108822

517. de Menezes, Mareliza Possa, Facin, Andreia Coutinho, Cardozo, Marita Vedovelli, Costa, Mirela Tinucci, Moraes, Paola Castro (2021). Evaluation of the Resistance Profile of Bacteria Obtained From Infected Sites of Dogs in a Veterinary Teaching Hospital in Brazil: A Retrospective Study. *Topics in companion animal medicine*, 42(#issue#), 100489

518. Guzman Ramos, P J, Shiel, R E, Fernandez Perez, C, Rios Boeta, A M, Perlado Chamizo, M R, Ballester Aguado, J I, Ruiz Duro, N, Ortiz-Diez, G (2021). Antimicrobial resistance increased over an 8-year period in Enterobacteriaceae cultured from canine urine samples. *The Journal of small animal practice*, 62(4), 279

519. Phongphaew, Wallaya, Kongtia, Mintraporn, Kim, Kiyeon, Sirinarumitr, Kaitkanoke, Sirinarumitr, Theerapol (2021). Association of bacterial isolates and antimicrobial susceptibility between prostatic fluid and urine samples in canine prostatitis with concurrent cystitis. *Theriogenology*, 173(#issue#), 202

520. Merino-Gutierrez, Virginia, Puig, Jordi, Feo-Bernabe, Luis (2022). Successful Treatment of 3 Dogs With Fluoroquinolone-Resistant Escherichia coli Associated Granulomatous Colitis. *Topics in companion animal medicine*, 47(#issue#), 100621

521. Fesler, Andrea T, Scholtzek, Anissa D, Schug, Angela R, Kohn, Barbara, Weingart, Christiane, Hanke, Dennis, Schink, Anne-Kathrin, Bethe, Astrid, Lubke-Becker, Antina, Schwarz, Stefan (2022). Antimicrobial and Biocide Resistance among Canine and Feline Enterococcus faecalis, Enterococcus faecium, Escherichia coli, Pseudomonas aeruginosa, and Acinetobacter baumannii Isolates from Diagnostic Submissions. *Antibiotics (Basel, Switzerland)*, 11(2), #Pages#

522. Rocha, Marcos F G, Paiva, Debora D Q, Amando, Bruno R, Melgarejo, Carliane M A, Freitas, Alyne S, Gomes, Francisco I F, Ocadaque, Crister J, Costa, Cecilia L, Guedes,

Glaucia M M, Lima-Neto, Reginaldo G, Cordeiro, Rossana A, Sidrim, Jose J C, Castelo-Branco, Debora S C M (2022). Antimicrobial susceptibility and production of virulence factors by bacteria recovered from bitches with pyometra. *Reproduction in domestic animals = Zuchthygiene*, 57(9), 1063

523. Lansubsakul, Niyada, Sirinarumitr, Kaitkanoke, Sirinarumitr, Theerapol, Imsilp, Kanjana, Wattananit, Podjana, Supanrung, Sasi, Limmanont, Chunsumon (2022). First report on clinical aspects, blood profiles, bacterial isolation, antimicrobial susceptibility, and histopathology in canine pyometra in Thailand. *Veterinary world*, 15(7), 1804

524. Haulisah, Nurul Asyiqin, Hassan, Latiffah, Jajere, Saleh Mohammed, Ahmad, Nur Indah, Bejo, Siti Khairani (2022). High prevalence of antimicrobial resistance and multidrug resistance among bacterial isolates from diseased pets: Retrospective laboratory data (2015-2017). *PloS one*, 17(12), e0277664

525. Tso, Suzanne Suk Kwan, Leister, Ellie, Sharp, Claire Rebecca, Heller, Jane, Gibson, Justine S (2022). Positive Airway Cultures in Dogs and Cats Receiving Mechanical Ventilation for Tick Paralysis. *Animals : an open access journal from MDPI*, 12(23), #Pages#

526. MG Ribeiro, TT Pereira, PJ de Lima Paz, BO de Almeida, CSA Cerviño, CA Rodrigues, GTS Santos, LM de Souza Freire, FVR Portilho, MFÁ Filho, NR Paschoal, TS Bello, J Megid, H Langoni, CM Appolinário, AS Borges, RM Amorim, R Giuffrida, JP de Oliveira Filho, AK Siqueira, FJP Listoni, AC Paes (2023). Bacterial identification in cerebrospinal fluid of domestic species with neurologic signs: a retrospective case-series study in 136 animals (2005-2021). *Brazilian journal of microbiology : [publication of the Brazilian Society for Microbiology]*, 54(1), #Pages#

527. Koontz, Caitlan W, Epstein, Steven E, Westropp, Jodi L (2023). Antimicrobial susceptibility patterns from urinary isolates obtained from cats (2013-2020). *Journal of veterinary internal medicine*, 37(3), 1077

528. Soonthornsit, J., Apiratwarrasakul, S., Phumthanakorn, N. (2022). Clinical characteristics, antimicrobial resistance and treatment outcomes of multidrug-resistant Escherichia coli infection in dogs and cats at a veterinary teaching hospital in Thailand. *Thai Journal of Veterinary Medicine*, 52(1), 207

529. Cleven, A. van, Boyen, F., Paepe, D., Chantziaras, I., Sarrazin, S., Haesebrouck, F., Dewulf, J. (2018). Presence of resistance against 'highest priority critically important antimicrobials' in Escherichia coli isolates from dogs and cats. *Vlaams Diergeneeskundig Tijdschrift*, 87(1), 22

530. Lien ChingJung, Wang ShangLin (2020). Antimicrobial resistance of common uropathogens from cats with urinary tract infections in a veterinary teaching hospital in

Taiwan. *Taiwan Veterinary Journal*, 46(1), 21

531. Murphy, C., Reid-Smith, R. J., Prescott, J. F., Bonnett, B. N., Poppe, C., Boerlin, P., Weese, J. S., Janecko, N., McEwen, S. A. (2009). Occurrence of antimicrobial resistant bacteria in healthy dogs and cats presented to private veterinary hospitals in southern Ontario: a preliminary study. *Canadian Veterinary Journal*, 50(10), 1047

532. Cole, S. D., Perez-Bonilla, D., Hallowell, A., Redding, L. E. (2022). Carbapenem prescribing at a veterinary teaching hospital before an outbreak of carbapenem-resistant Escherichia coli. *Journal of Small Animal Practice*, 63(6), 442

533. Njoroge, C. W., Mande, J. D., Mitema, E. S., Kitaa, J. M. A. (2016). Multidrug resistance of common bacterial pathogens from wounds and otitis externa in small animals during a 10 year period in Kenya. *International Journal of Veterinary Science*, 5(4), 262

534. Shahaza, O., Mohd. Azizul, O., Zakirah, S., Muhammad Azim, F. A. G., Syamsyul, A., Maswati, M. A. (2017). Antimicrobial resistance in veterinary clinical isolates of Escherichia coli from northern region of Peninsular Malaysia. *Malaysian Journal of Veterinary Research*, 8(2), 1

535. Shimada, E., Miyamoto, T., Hatoya, S. (2011). Prevalence and antimicrobial drug susceptibility of Gram-negative bacteria from canine and feline clinical specimens. *Journal of the Japan Veterinary Medical Association*, 64(11), 879

536. Shimada, E., Miyamoto, T., Hatoya, S. (2011). Causative bacteria and antibiogram in various infectious diseases in canines and felines. *Journal of the Japan Veterinary Medical Association*, 64(10), 810

537. Scartezzini, M., Cordova, D. de M., Lima, D. A. de, Silva, J. C. J. da, Oliveira, S. J. de (2011). Bacteriologic diagnosis of different pathologies of dogs and cats, antimicrobial susceptibility tests. *Veterinaria em foco*, 8(2), 152

538. Kimura, Y., Shimada, E., Miyamoto, T., Hatoya, S. (2014). Treatment outcomes of third-generation cephalosporin-susceptible and resistant Enterobacteriaceae infection in dogs and cats. *Journal of the Japan Veterinary Medical Association*, 67(6), 419

539. Shi ZhiHai, Wang WenJia, Lan YaLi, Chen ChaoXi (2013). Analysis of antibiotic resistance of Escherichia coli isolated from pets. *Animal Husbandry and Feed Science*, 5(1), 22

540. Shimada, E., Miyamoto, T., Kimura, Y., Kakumoto, M., Hatoya, S. (2012). Antimicrobial sensitivity of clinical isolates from dogs and cats in Yamaguchi City in 2011. *Journal of Animal Clinical Medicine*, 21(4), 170

541. Azevedo, J. S., Petrucci, C. G., Rodrigues, P. R. C., Oliveira, S. J. de (2003). Antimicrobial susceptibility of bacteria isolated from various disease conditions in dogs and

cats. *Veterinaria em foco*, 1(1), 77

542. Larsson Júnior, C. E., Henriques, D. A., Sales, T. C., Moraes, C. T. P., Megale, L. A. (2009). Antimicrobial activity of cefovecin against bacterial pathogens isolated from clinical samples of dogs and cats in São Paulo, Brazil. *34th World Small Animal Veterinary Association Congress, São Paulo, Brazil, 21-24 July 2009*, #volume#(#issue#), unpaginated

543. Hariharan, H., Coles, M., Poole, D., Lund, L., Page, R. (2006). Update on antimicrobial susceptibilities of bacterial isolates from canine and feline otitis externa. *Canadian Veterinary Journal*, 47(3), 253

544. Ortiz-Díez, G., Luque Mengíbar, R., Turrientes, M. C., Baquero Artigao, M. R., López Gallifa, R., Maroto Tello, A., Fernández Pérez, C., Santiago, T. A. (2023). Prevalence, incidence and risk factors for acquisition and colonization of extended-spectrum beta-lactamase- and carbapenemase-producing Enterobacteriaceae from dogs attended at a veterinary hospital in Spain. *Comparative Immunology, Microbiology & Infectious Diseases*, 92(#issue#), #Pages#

545. Awji, E. G., Damte, D., Lee SeungJin, Lee JoongSu, Kim YoungHoan, Park SeungChun (2012). The in vitro activity of 15 antimicrobial agents against bacterial isolates from dogs. *Journal of Veterinary Medical Science*, 74(8), 1091

546. Köse, S. I., Maden, M., Sayın, Z. (2021). Clinical and bacteriological analysis of respiratory tract infections in sheltered dogs and determination of antibacterial treatment options. *Journal of the Hellenic Veterinary Medical Society*, 72(4), 3491

547. Ogunleye, A. O., Omobowale, T. O., Okunlade, A., Ajuwape, A. T. P., Adetosoye, A. I. (2012). Multi-drug resistant bacteria isolated from dogs presented with otitis externa in a Veterinary Teaching Hospital in Nigeria. *Tropical Veterinarian*, 30(2), 74

548. Lobetti, R. G., Joubert, K. E., Picard, J., Carstens, J., Pretorius, E. (2002). Bacterial colonization of intravenous catheters in young dogs suspected to have parvoviral enteritis. *Journal of the American Veterinary Medical Association*, 220(9), 1321

549. Woerde, Dennis J, Reagan, Krystle L, Byrne, Barbara A, Weimer, Bart C, Epstein, Steven E, Schlesener, Cory, Huang, Bihua C, Sykes, Jane E (2023). Characteristics of Extended-Spectrum β-Lactamase Producing Enterobacterales Isolated from Dogs and Cats, 2011-2021. *Veterinary sciences*, 10(3), #Pages#

550. Ekakoro, John E, Guptill, Lynn, Hendrix, Kenitra, Anderson, Melinda, Ruple, Audrey (2023). Approaches Used to Construct Antibiograms for Dogs in a Veterinary Teaching Hospital in the United States *Antibiotics*, 12(6), 1034

551. Jordana Almeida Santana, Amanda Oliveira Paraguassu, Ranielle Stephanie Toledo Santana, Rafael Gariglio Clark Xavier, Colleto Freitas, Patricia Maria, Flavia Figueira Aburjaile, Vasco Ariston de Carvalho Azevedo, Brenig, Bertram, Anders Miki Bojesen,

Silveira Silva, Rodrigo Otávio (2023). Risk Factors, Genetic Diversity, and Antimicrobial Resistance of Staphylococcus spp. Isolates in Dogs Admitted to an Intensive Care Unit of a Veterinary Hospital *Antibiotics*, 12(3), 621

552. Meepoo, Wannisa, Jaroensong, Tassanee, Pruksakorn, Chantima, Rattanasrisomporn, Jatuporn (2022). Investigation of Bacterial Isolations and Antimicrobial Susceptibility of Chronic Rhinitis in Cats *Animals*, 12(12), 1572

553. Melo, Roberta T, Oliveira, Raquel P, Silva, Beatryz F, Monteiro, Guilherme P, João Paulo E Saut, Costa, Letícia R M, Sthéfany Da C Dias, Rossi, Daise A (2022). Phylogeny and Virulence Factors of Escherichia coli Isolated from Dogs with Pyometra *Veterinary Sciences*, 9(4), 158

554. Juin Jia Sim, Seng Fong Lau, Omar, Sharina, Watanabe, Malaika, Muhammad Waseem Aslam (2021). A Retrospective Study on Bacteriology, Clinicopathologic and Radiographic Features in 28 Cats Diagnosed with Pyothorax *Animals*, 11(8), 2286

555. Curran, Katie, Leeper, Haley, Kathy O′Reilly, Jacob, Joelle, Bermudez, Luiz E (2021). An Analysis of the Infections and Determination of Empiric Antibiotic Therapy in Cats and Dogs with Cancer-Associated Infections *Antibiotics*, 10(6), 700

556. Nocera, Francesca Paola, Ambrosio, Monica, Fiorito, Filomena (2021). On Gram-Positive- and Gram-Negative-Bacteria-Associated Canine and Feline Skin Infections: A 4-Year Retrospective Study of the University Veterinary Microbiology Diagnostic Laboratory of Naples, Italy *Animals*, 11(6), 1603

557. Shnaiderman-Torban, Anat, Navon-Venezia, Shiri, Kelmer, Efrat, Cohen, Adar, Paitan, Yossi, Arielly, Haya, Steinman, Amir (2020). Extended-Spectrum β-Lactamase-Producing Enterobacterales Shedding by Dogs and Cats Hospitalized in an Emergency and Critical Care Department of a Veterinary Teaching Hospital *Antibiotics*, 9(9), 545

558. Thepmanee, Jirapa, Rodroo, Jutamart, Awaiwanont, Nattakarn, Intanon, Montira, Na Lampang, Kannika, Thitaram, Niyada, Thongkorn, Kriangkrai (2019). Investigation of Extended-Spectrum Beta-Lactamase (ESBL)- producing Escherichia coli and antimicrobial resistance in dogs with periodontal disease *The Thai Journal of Veterinary Medicine*, 49(3), 227

559. Kalhoro, Dildar Hussain, Ansari, Mansoor Ibrahim, Abro, Shahid Hussain, Kalhoro, Muhammad Saleem, Tuino, Ahmed Nawaz, Kumbhar, Shahnawaz, Kaka, Asmatullah, Lochi, Ghulam Murtaza, Mazari, Muhammad Qasim, Chandio, Muhammad Ali (2019). Prevalence and antimicrobial sensitivity of bacterial isolates from canine wound infection in Tandojam, Sindh *Pure and Applied Biology*, 8(1), 372

560. Flisi, Sara, Manuel Dall'Aglio, Spadini, Costanza, Cabassi, Clotilde Silvia, Quintavalla, Fausto (2018). Microbial Isolates from Vegetable Foreign Bodies Inhaled by Dogs *Veterinary Medicine International*, 2018(#issue#), 9

561. Hartantyo, SHP, Chau, ML, Fillon, L, Ariff, AZB, Kang, JSL, Aung, KT, Gutierrez, RA (2018). Sick pets as potential reservoirs of antibiotic-resistant bacteria in Singapore *Antimicrobial Resistance And Infection Control*, 7(#issue#), #Pages#

562. Habib, I, Anjum, AA, Rabbani, M, Ahmad, MUD, Ali, MA, Nawaz, M, Kamran, M, Khan, HM (2016). Occurrence Of Antimicrobial Resistant Bacteria In Dogs Suffering From Enteritis *Journal Of Animal And Plant Sciences*, 26(1), 13

563. Seol, B, Matanovic, K, Mekic, S, Staresina, V (2011). In vitro activity of cefovecin, extended-spectrum cephalosporin, against 284 clinical isolates collected from cats and dogs in Croatia *Veterinarski Arhiv*, 81(1), 91

564. Gorz, M, Rohde, J, Amtsberg, G (1999). Prevalence and antibiotic resistance of bacterial pathogens isolated from dogs *Kleintierpraxis*, 44(2), 77

Level 1, Form level_1_screening, What was the study type? -> To understand the molecular basis of resistance

565. Nolan, L K, Wooley, R E, Brown, J, Blue, J L, Camp, M (1987). Comparison of virulence factors and antibiotic resistance profiles of Escherichia coli strains from humans and dogs with urinary tract infections. *Journal of veterinary internal medicine*, 1(4), 152

566. Aly, Sherine A, Debavalya, Nipattra, Suh, Sang-Jin, Oryazabal, Omar A, Boothe, Dawn M (2012). Molecular mechanisms of antimicrobial resistance in fecal Escherichia coli of healthy dogs after enrofloxacin or amoxicillin administration. *Canadian journal of microbiology*, 58(11), 1288

567. Kameda, M, Harada, K, Suzuki, M, Mitshuhasi, S (1969). Formation of transferable drug resistance factor by recombination between resistance determinants and transfer factors. *Japanese journal of microbiology*, 13(3), 255

568. Monaghan, C, Tierney, U, Colleran, E (1981). Antibiotic resistance and R-factors in the fecal coliform flora of urban and rural dogs. *Antimicrobial agents and chemotherapy*, 19(2), 266

569. Y Péan (2000). Surveillance of bacterial antibiotic resistance. *Presse medicale (Paris, France : 1983)*, 29(37), #Pages#

570. Feria, Constanca, Ferreira, Eugenia, Correia, Jose Duarte, Goncalves, Jose, Canica, Manuela (2002). Patterns and mechanisms of resistance to beta-lactams and beta-lactamase inhibitors in uropathogenic Escherichia coli isolated from dogs in Portugal. *The Journal of antimicrobial chemotherapy*, 49(1), 77

571. Lanz, Roland, Kuhnert, Peter, Boerlin, Patrick (2003). Antimicrobial resistance and resistance gene determinants in clinical Escherichia coli from different animal species in Switzerland. *Veterinary microbiology*, 91(1), 73

572. Son, R, Rusu, G, Karim, M I (1997). Conjugal transfer of plasmids and antibiotic resistance among Escherichia coli isolated from animals in a rural area in Sarawak (Malaysia). *Journal of applied microbiology*, 82(2), 240

573. Prapasarakul, Nuvee, Ochi, Kozo, Adachi, Yoshikazu (2003). In vitro susceptibility and a new point mutation associated with tylosin-resistance in Japanese canine intestinal spirochetes. *The Journal of veterinary medical science*, 65(12), 1275

574. Bryan, Andrew, Shapir, Nir, Sadowsky, Michael J (2004). Frequency and distribution of tetracycline resistance genes in genetically diverse, nonselected, and nonclinical Escherichia coli strains isolated from diverse human and animal sources. *Applied and environmental microbiology*, 70(4), 2503

575. A Carattoli, S Lovari, A Franco, G Cordaro, P Di Matteo, A Battisti (2005). Extended-spectrum beta-lactamases in Escherichia coli isolated from dogs and cats in Rome, Italy, from 2001 to 2003. *Antimicrobial agents and chemotherapy*, 49(2), #Pages#

576. Sidjabat, Hanna E, Townsend, Kirsty M, Hanson, Nancy D, Bell, Jan M, Stokes, H W, Gobius, Kari S, Moss, Susan M, Trott, Darren J (2006). Identification of bla(CMY-7) and associated plasmid-mediated resistance genes in multidrug-resistant Escherichia coli isolated from dogs at a veterinary teaching hospital in Australia. *The Journal of antimicrobial chemotherapy*, 57(5), 840

577. C Pomba, N Mendonça, M Costa, D Louro, B Baptista, M Ferreira, JD Correia, M Caniça (2006). Improved multiplex PCR method for the rapid detection of beta-lactamase genes in Escherichia coli of animal origin. *Diagnostic microbiology and infectious disease*, 56(1), #Pages#

578. I Rosas, E Salinas, L Martínez, E Calva, A Cravioto, C Eslava, CF Amábile-Cuevas (2006). Urban dust fecal pollution in Mexico City: antibiotic resistance and virulence factors of Escherichia coli. *International journal of hygiene and environmental health*, 209(5), #Pages#

579. Li, Xian-Zhi, Mehrotra, Manisha, Ghimire, Shiva, Adewoye, Lateef (2007). beta-Lactam resistance and beta-lactamases in bacteria of animal origin. *Veterinary microbiology*, 121(3-

4), 197

580. Costa, Daniela, Poeta, Patricia, Saenz, Yolanda, Coelho, Ana Claudia, Matos, Manuela, Vinue, Laura, Rodrigues, Jorge, Torres, Carmen (2008). Prevalence of antimicrobial resistance and resistance genes in faecal Escherichia coli isolates recovered from healthy pets. *Veterinary microbiology*, 127(1-2), 97

581. A Moreno, H Bello, D Guggiana, M Domínguez, G González (2008). Extended-spectrum beta-lactamases belonging to CTX-M group produced by Escherichia coli strains isolated from companion animals treated with enrofloxacin. *Veterinary microbiology*, 129(1-2), #Pages#

582. K Kadlec, S Schwarz (2008). Analysis and distribution of class 1 and class 2 integrons and associated gene cassettes among Escherichia coli isolates from swine, horses, cats and dogs collected in the BfT-GermVet monitoring study. *The Journal of antimicrobial chemotherapy*, 62(3), #Pages#

583. Ma, Junying, Zeng, Zhenling, Chen, Zhangliu, Xu, Xiaogang, Wang, Xiaoying, Deng, Yuting, Lu, Dianhong, Huang, Liangzong, Zhang, Yunyuan, Liu, Jianhua, Wang, Minggui (2009). High prevalence of plasmid-mediated quinolone resistance determinants qnr, aac(6')-Ib-cr, and qepA among ceftiofur-resistant Enterobacteriaceae isolates from companion and food-producing animals. *Antimicrobial agents and chemotherapy*, 53(2), 519

584. Johnson, James R, Kuskowski, Michael A, Owens, Krista, Clabots, Connie, Singer, Randall S (2009). Virulence genotypes and phylogenetic background of fluoroquinolone-resistant and susceptible Escherichia coli urine isolates from dogs with urinary tract infection. *Veterinary microbiology*, 136(1-2), 108

585. SK Lim, HS Lee, HM Nam, SC Jung, YC Bae (2009). CTX-M-type beta-lactamase in Escherichia coli isolated from sick animals in Korea. *Microbial drug resistance (Larchmont, N.Y.)*, 15(2), #Pages#

586. Shaheen, Bashar W, Wang, Chengming, Johnson, Calvin M, Kaltenboeck, Bernhard, Boothe, Dawn M (2009). Detection of fluoroquinolone resistance level in clinical canine and feline Escherichia coli pathogens using rapid real-time PCR assay. *Veterinary microbiology*, 139(3-4), 379

587. JS Gibson, RN Cobbold, DJ Trott (2010). Characterization of multidrug-resistant Escherichia coli isolated from extraintestinal clinical infections in animals. *Journal of medical microbiology*, 59(Pt 5), #Pages#

588. Shaheen, Bashar W, Oyarzabal, Omar A, Boothe, Dawn M (2010). The role of class 1 and 2 integrons in mediating antimicrobial resistance among canine and feline clinical E. coli isolates from the US. *Veterinary microbiology*, 144(3-4), 363

589. Gibson, Justine S, Cobbold, Rowland N, Kyaw-Tanner, Myat T, Heisig, Peter, Trott, Darren J (2010). Fluoroquinolone resistance mechanisms in multidrug-resistant Escherichia coli isolated from extraintestinal infections in dogs. *Veterinary microbiology*, 146(1-2), 161

590. JL Platell, RN Cobbold, JR Johnson, DJ Trott (2010). Clonal group distribution of fluoroquinolone-resistant Escherichia coli among humans and companion animals in Australia. *The Journal of antimicrobial chemotherapy*, 65(9), #Pages#

591. Ho, P L, Chow, K H, Lai, Eileen L, Lo, W U, Yeung, M K, Chan, Jane, Chan, P Y, Yuen, K Y (2011). Extensive dissemination of CTX-M-producing Escherichia coli with multidrug resistance to 'critically important' antibiotics among food animals in Hong Kong, 2008-10. *The Journal of antimicrobial chemotherapy*, 66(4), 765

592. Gebru, Elias, Choi, Myung-Jin, Lee, Seung-Jin, Damte, Dereje, Park, Seung Chun (2011). Mutant-prevention concentration and mechanism of resistance in clinical isolates and enrofloxacin/marbofloxacin-selected mutants of Escherichia coli of canine origin. *Journal of medical microbiology*, 60(Pt 10), 1512

593. JL Platell, RN Cobbold, JR Johnson, A Heisig, P Heisig, C Clabots, MA Kuskowski, DJ Trott (2011). Commonality among fluoroquinolone-resistant sequence type ST131 extraintestinal Escherichia coli isolates from humans and companion animals in Australia. *Antimicrobial agents and chemotherapy*, 55(8), #Pages#

594. JL Platell, JR Johnson, RN Cobbold, DJ Trott (2011). Multidrug-resistant extraintestinal pathogenic Escherichia coli of sequence type ST131 in animals and foods. *Veterinary microbiology*, 153(1-2), #Pages#

595. Y Deng, L He, S Chen, H Zheng, Z Zeng, Y Liu, Y Sun, J Ma, Z Chen, JH Liu (2011). F33:A-:B- and F2:A-:B- plasmids mediate dissemination of rmtB-blaCTX-M-9 group genes and rmtB-qepA in Enterobacteriaceae isolates from pets in China. *Antimicrobial agents and chemotherapy*, 55(10), #Pages#

596. Shaheen, Bashar W, Nayak, Rajesh, Foley, Steven L, Kweon, Ohgew, Deck, Joanna, Park, Miseon, Rafii, Fatemeh, Boothe, Dawn M (2011). Molecular characterization of resistance to extended-spectrum cephalosporins in clinical Escherichia coli isolates from companion animals in the United States. *Antimicrobial agents and chemotherapy*, 55(12), 5666

597. Hou, Jianxia, Huang, Xianhui, Deng, Yuting, He, Liangying, Yang, Tong, Zeng, Zhenling, Chen, Zhangliu, Liu, Jian-Hua (2012). Dissemination of the fosfomycin resistance gene fosA3 with CTX-M beta-lactamase genes and rmtB carried on IncFII plasmids among Escherichia coli isolates from pets in China. *Antimicrobial agents and chemotherapy*, 56(4), 2135

598. MD Tamang, HM Nam, GC Jang, SR Kim, MH Chae, SC Jung, JW Byun, YH Park, SK Lim (2012). Molecular characterization of extended-spectrum-β-lactamase-producing and

plasmid-mediated AmpC β-lactamase-producing Escherichia coli isolated from stray dogs in South Korea. *Antimicrobial agents and chemotherapy*, 56(5), #Pages#

599. Harada, Kazuki, Niina, Ayaka, Nakai, Yuka, Kataoka, Yasushi, Takahashi, Toshio (2012). Prevalence of antimicrobial resistance in relation to virulence genes and phylogenetic origins among urogenital Escherichia coli isolates from dogs and cats in Japan. *American journal of veterinary research*, 73(3), 409

600. CM Dierikx, E van Duijkeren, AH Schoormans, A van Essen-Zandbergen, K Veldman, A Kant, XW Huijsdens, K van der Zwaluw, JA Wagenaar, DJ Mevius (2012). Occurrence and characteristics of extended-spectrum-β-lactamase- and AmpC-producing clinical isolates derived from companion animals and horses. *The Journal of antimicrobial chemotherapy*, 67(6), #Pages#

601. Harada, Kazuki, Nakai, Yuka, Kataoka, Yasushi (2012). Mechanisms of resistance to cephalosporin and emergence of O25b-ST131 clone harboring CTX-M-27 beta-lactamase in extraintestinal pathogenic Escherichia coli from dogs and cats in Japan. *Microbiology and immunology*, 56(7), 480

602. K Albrechtova, M Dolejska, A Cizek, D Tausova, J Klimes, L Bebora, I Literak (2012). Dogs of nomadic pastoralists in northern Kenya are reservoirs of plasmid-mediated cephalosporin- and quinolone-resistant Escherichia coli, including pandemic clone B2-O25-ST131. *Antimicrobial agents and chemotherapy*, 56(7), #Pages#

603. C Ewers, A Bethe, T Semmler, S Guenther, LH Wieler (2012). Extended-spectrum β-lactamase-producing and AmpC-producing Escherichia coli from livestock and companion animals, and their putative impact on public health: a global perspective. *Clinical microbiology and infection : the official publication of the European Society of Clinical Microbiology and Infectious Diseases*, 18(7), #Pages#

604. JL Platell, DJ Trott, JR Johnson, P Heisig, A Heisig, CR Clabots, B Johnston, RN Cobbold (2012). Prominence of an O75 clonal group (clonal complex 14) among non-ST131 fluoroquinolone-resistant Escherichia coli causing extraintestinal infections in humans and dogs in Australia. *Antimicrobial agents and chemotherapy*, 56(7), #Pages#

605. Liu, Xiaoqiang, Boothe, Dawn M, Thungrat, Kamoltip, Aly, Sherine (2012). Mechanisms accounting for fluoroquinolone multidrug resistance Escherichia coli isolated from companion animals. *Veterinary microbiology*, 161(1-2), 159

606. Trott, Darren (2013). beta-lactam resistance in gram-negative pathogens isolated from animals. *Current pharmaceutical design*, 19(2), 239

607. Ho, P L, Chan, J, Lo, W U, Law, P Y, Li, Z, Lai, E L, Chow, K H (2013). Dissemination of plasmid-mediated fosfomycin resistance fosA3 among multidrug-resistant Escherichia coli from livestock and other animals. *Journal of applied microbiology*, 114(3), 695

608. Sallem, Rym Ben, Gharsa, Haythem, Slama, Karim Ben, Rojo-Bezares, Beatriz, Estepa, Vanesa, Porres-Osante, Nerea, Jouini, Ahlem, Klibi, Naouel, Saenz, Yolanda, Boudabous, Abdellatif, Torres, Carmen (2013). First detection of CTX-M-1, CMY-2, and QnrB19 resistance mechanisms in fecal Escherichia coli isolates from healthy pets in Tunisia. *Vector borne and zoonotic diseases (Larchmont, N.Y.)*, 13(2), 98

609. Shaheen, Bashar W, Nayak, Rajesh, Foley, Steven L, Boothe, Dawn M (2013). Chromosomal and plasmid-mediated fluoroquinolone resistance mechanisms among broad-spectrum-cephalosporin-resistant Escherichia coli isolates recovered from companion animals in the USA. *The Journal of antimicrobial chemotherapy*, 68(5), 1019

610. Sato, Toyotaka, Yokota, Shin-Ichi, Okubo, Torahiko, Usui, Masaru, Fujii, Nobuhiro, Tamura, Yutaka (2014). Phylogenetic association of fluoroquinolone and cephalosporin resistance of D-O1-ST648 Escherichia coli carrying blaCMY-2 from faecal samples of dogs in Japan. *Journal of medical microbiology*, 63(Pt 2), 263

611. BW Shaheen, R Nayak, DM Boothe (2013). Emergence of a New Delhi metallo-β-lactamase (NDM-1)-encoding gene in clinical Escherichia coli isolates recovered from companion animals in the United States. *Antimicrobial agents and chemotherapy*, 57(6), #Pages#

612. S Dahmen, M Haenni, P Châtre, JY Madec (2013). Characterization of blaCTX-M IncFII plasmids and clones of Escherichia coli from pets in France. *The Journal of antimicrobial chemotherapy*, 68(12), #Pages#

613. C Pomba, L López-Cerero, M Bellido, L Serrano, A Belas, N Couto, P Cavaco-Silva, J Rodríguez-Baño, A Pascual (2014). Within-lineage variability of ST131 Escherichia coli isolates from humans and companion animals in the south of Europe. *The Journal of antimicrobial chemotherapy*, 69(1), #Pages#

614. de Cleber Jacob Silva Paula, Marin, Jose Moacir (2008). Isolation of extraintestinal pathogenic Escherichia coli from diarrheic dogs and their antimicrobial resistance profile. *Brazilian journal of microbiology : [publication of the Brazilian Society for Microbiology]*, 39(3), 498

615. RP Maluta, AE Stella, K Riccardi, EC Rigobelo, JM Marin, MB Carvalho, FA de Ávila (2012). Phenotypical characterization and adhesin identification in Escherichia coli strains isolated from dogs with urinary tract infections. *Brazilian journal of microbiology : [publication of the Brazilian Society for Microbiology]*, 43(1), #Pages#

616. C Ewers, A Bethe, I Stamm, M Grobbel, PA Kopp, B Guerra, M Stubbe, Y Doi, Z Zong, A Kola, K Schaufler, T Semmler, A Fruth, LH Wieler, S Guenther (2014). CTX-M-15-D-ST648 Escherichia coli from companion animals and horses: another pandemic clone combining multiresistance and extraintestinal virulence? *The Journal of antimicrobial chemotherapy*, 69(5), #Pages#

617. V Bortolaia, KH Hansen, CA Nielsen, TR Fritsche, L Guardabassi (2014). High diversity of plasmids harbouring blaCMY-2 among clinical Escherichia coli isolates from humans and companion animals in the upper Midwestern USA. *The Journal of antimicrobial chemotherapy*, 69(6), #Pages#

618. JR Johnson, O Clermont, B Johnston, C Clabots, V Tchesnokova, E Sokurenko, AF Junka, B Maczynska, E Denamur (2014). Rapid and specific detection, molecular epidemiology, and experimental virulence of the O16 subgroup within Escherichia coli sequence type 131. *Journal of clinical microbiology*, 52(5), #Pages#

619. Yang, Tong, Zeng, Zhenling, Rao, Lili, Chen, Xiaojie, He, Dandan, Lv, Luchao, Wang, Jing, Zeng, Li, Feng, Minsha, Liu, Jian-Hua (2014). The association between occurrence of plasmid-mediated quinolone resistance and ciprofloxacin resistance in Escherichia coli isolates of different origins. *Veterinary microbiology*, 170(1-2), 89

620. D Timofte, IE Maciuca, K Kemmett, A Wattret, NJ Williams (2014). Detection of the human-pandemic Escherichia coli B2-O25b-ST131 in UK dogs. *The Veterinary record*, 174(14), #Pages#

621. JE Rubin, JD Pitout (2014). Extended-spectrum β-lactamase, carbapenemase and AmpC producing Enterobacteriaceae in companion animals. *Veterinary microbiology*, 170(1-2), #Pages#

622. Sato, Toyotaka, Yokota, Shin-ichi, Ichihashi, Risa, Miyauchi, Tomoka, Okubo, Torahiko, Usui, Masaru, Fujii, Nobuhiro, Tamura, Yutaka (2014). Isolation of Escherichia coli strains with AcrAB-TolC efflux pump-associated intermediate interpretation or resistance to fluoroquinolone, chloramphenicol and aminopenicillin from dogs admitted to a university veterinary hospital. *The Journal of veterinary medical science*, 76(7), 937

623. T Okubo, T Sato, S Yokota, M Usui, Y Tamura (2014). Comparison of broad-spectrum cephalosporin-resistant Escherichia coli isolated from dogs and humans in Hokkaido, Japan. *Journal of infection and chemotherapy : official journal of the Japan Society of Chemotherapy*, 20(4), #Pages#

624. Tramuta, Clara, Robino, Patrizia, Nucera, Daniele, Salvarani, Sara, Banche, Giuliana, Malabaila, Aurelio, Nebbia, Patrizia (2014). Molecular characterization and antimicrobial resistance of faecal and urinary Escherichia coli isolated from dogs and humans in Italy. *Veterinaria italiana*, 50(1), 23

625. Vingopoulou, E I, Siarkou, V I, Batzias, G, Kaltsogianni, F, Sianou, E, Tzavaras, I, Koutinas, A, Saridomichelakis, M N, Sofianou, D, Tzelepi, E, Miriagou, V (2014). Emergence and maintenance of multidrug-resistant Escherichia coli of canine origin harbouring a blaCMY-2-IncI1/ST65 plasmid and topoisomerase mutations. *The Journal of antimicrobial chemotherapy*, 69(8), 2076

626. JM Agostinho, A de Souza, RP Schocken-Iturrino, LG Beraldo, CA Borges, FA Avila, JM Marin (2014). Escherichia coli Strains Isolated from the Uteri Horn, Mouth, and Rectum of Bitches Suffering from Pyometra: Virulence Factors, Antimicrobial Susceptibilities, and Clonal Relationships among Strains. *International journal of microbiology*, 2014(), #Pages#

627. R Ben Sallem, K Ben Slama, B Rojo-Bezares, N Porres-Osante, A Jouini, N Klibi, A Boudabous, Y Sáenz, C Torres (2014). IncI1 plasmids carrying bla(CTX-M-1) or bla(CMY-2) genes in Escherichia coli from healthy humans and animals in Tunisia. *Microbial drug resistance (Larchmont, N.Y.)*, 20(5), #Pages#

628. A Belas, AS Salazar, LT Gama, N Couto, C Pomba (2014). Risk factors for faecal colonisation with Escherichia coli producing extended-spectrum and plasmid-mediated AmpC β-lactamases in dogs. *The Veterinary record*, 175(8), #Pages#

629. J Schmiedel, L Falgenhauer, E Domann, R Bauerfeind, E Prenger-Berninghoff, C Imirzalioglu, T Chakraborty (2014). Multiresistant extended-spectrum β-lactamase-producing Enterobacteriaceae from humans, companion animals and horses in central Hesse, Germany. *BMC microbiology*, 14(), #Pages#

630. L Valentin, H Sharp, K Hille, U Seibt, J Fischer, Y Pfeifer, GB Michael, S Nickel, J Schmiedel, L Falgenhauer, A Friese, R Bauerfeind, U Roesler, C Imirzalioglu, T Chakraborty, R Helmuth, G Valenza, G Werner, S Schwarz, B Guerra, B Appel, L Kreienbrock, A Käsbohrer (2014). Subgrouping of ESBL-producing Escherichia coli from animal and human sources: an approach to quantify the distribution of ESBL types between different reservoirs. *International journal of medical microbiology : IJMM*, 304(7), #Pages#

631. Windahl, Ulrika, Holst, Bodil Strom, Nyman, Ann, Gronlund, Ulrika, Bengtsson, Bjorn (2014). Characterisation of bacterial growth and antimicrobial susceptibility patterns in canine urinary tract infections. *BMC veterinary research*, 10(#issue#), 217

632. Liu, Xiaoqiang, Lazzaroni, Caterina, Aly, Sherine A, Thungrat, Kamoltip, Boothe, Dawn M (2014). In vitro selection of resistance to pradofloxacin and ciprofloxacin in canine uropathogenic Escherichia coli isolates. *Veterinary microbiology*, 174(3-4), 514

633. K Albrechtova, I Papousek, H De Nys, M Pauly, E Anoh, A Mossoun, M Dolejska, M Masarikova, S Metzger, E Couacy-Hymann, C Akoua-Koffi, RM Wittig, J Klimes, A Cizek, FH Leendertz, I Literak (2014). Low rates of antimicrobial-resistant Enterobacteriaceae in wildlife in Taï National Park, Côte d'Ivoire, surrounded by villages with high prevalence of multiresistant ESBL-producing Escherichia coli in people and domestic animals. *PloS one*, 9(12), #Pages#

634. MG Maaland, SS Mo, S Schwarz, L Guardabassi (2015). In vitro assessment of chloramphenicol and florfenicol as second-line antimicrobial agents in dogs. *Journal of veterinary pharmacology and therapeutics*, 38(5), #Pages#

635. Jackson, C R, Davis, J A, Frye, J G, Barrett, J B, Hiott, L M (2015). Diversity of Plasmids and Antimicrobial Resistance Genes in Multidrug-Resistant Escherichia coli Isolated from Healthy Companion Animals. *Zoonoses and public health*, 62(6), 479

636. K Schaufler, A Bethe, A Lübke-Becker, C Ewers, B Kohn, LH Wieler, S Guenther (2015). Putative connection between zoonotic multiresistant extended-spectrum beta-lactamase (ESBL)-producing Escherichia coli in dog feces from a veterinary campus and clinical isolates from dogs. *Infection ecology & epidemiology*, 5(), #Pages#

637. Chang, Shao-Kuang, Lo, Dan-Yuan, Wei, Hen-Wei, Kuo, Hung-Chih (2015). Antimicrobial resistance of Escherichia coli isolates from canine urinary tract infections. *The Journal of veterinary medical science*, 77(1), 59

638. Schmidt, Vanessa M, Pinchbeck, Gina L, Nuttall, Tim, McEwan, Neil, Dawson, Susan, Williams, Nicola J (2015). Antimicrobial resistance risk factors and characterisation of faecal E. coli isolated from healthy Labrador retrievers in the United Kingdom. *Preventive veterinary medicine*, 119(1-2), 31

639. Falgenhauer, Linda, Schmiedel, Judith, Ghosh, Hiren, Fritzenwanker, Moritz, Yao, Yancheng, Bauerfeind, Rolf, Imirzalioglu, Can, Chakraborty, Trinad (2014). Resistance plasmids in ESBL-encoding Escherichia coli isolates from humans, dogs and cats. *Berliner und Munchener tierarztliche Wochenschrift*, 127(11-12), 458

640. Aly, Sherine A, Boothe, Dawn M, Suh, Sang-Jin (2015). A novel alanine to serine substitution mutation in SoxS induces overexpression of efflux pumps and contributes to multidrug resistance in clinical Escherichia coli isolates. *The Journal of antimicrobial chemotherapy*, 70(8), 2228

641. Meireles, D, Leite-Martins, L, Bessa, L J, Cunha, S, Fernandes, R, de Matos, A, Manaia, C M, Martins da Costa, P (2015). Molecular characterization of quinolone resistance mechanisms and extended-spectrum beta-lactamase production in Escherichia coli isolated from dogs. *Comparative immunology, microbiology and infectious diseases*, 41(#issue#), 43

642. LX Fang, J Sun, L Li, H Deng, T Huang, QE Yang, X Li, MY Chen, XP Liao, YH Liu (2015). Dissemination of the chromosomally encoded CMY-2 cephalosporinase gene in Escherichia coli isolated from animals. *International journal of antimicrobial agents*, 46(2), #Pages#

643. L Clemente, V Manageiro, D Jones-Dias, I Correia, P Themudo, T Albuquerque, M Geraldes, F Matos, C Almendra, E Ferreira, M Caniça (2015). Antimicrobial susceptibility and oxymino-β-lactam resistance mechanisms in Salmonella enterica and Escherichia coli isolates from different animal sources. *Research in microbiology*, 166(7), #Pages#

644. Piras, Cristian, Soggiu, Alessio, Greco, Viviana, Martino, Piera Anna, Del Chierico, Federica, Putignani, Lorenza, Urbani, Andrea, Nally, Jarlath E, Bonizzi, Luigi, Roncada, Paola (2015). Mechanisms of antibiotic resistance to enrofloxacin in uropathogenic

Escherichia coli in dog. *Journal of proteomics*, 127(Pt B), 365

645. P Bogaerts, TD Huang, W Bouchahrouf, C Bauraing, C Berhin, F El Garch, Y Glupczynski, (2015). Characterization of ESBL- and AmpC-Producing Enterobacteriaceae from Diseased Companion Animals in Europe. *Microbial drug resistance (Larchmont, N.Y.)*, 21(6), #Pages#

646. Espinosa-Gongora, Carmen, Shah, Syed Qaswar Ali, Jessen, Lisbeth Rem, Bortolaia, Valeria, Langebaek, Rikke, Bjornvad, Charlotte Reinhard, Guardabassi, Luca (2015). Quantitative assessment of faecal shedding of beta-lactam-resistant Escherichia coli and enterococci in dogs. *Veterinary microbiology*, 181(3-4), 298

647. M Rzewuska, I Stefańska, M Kizerwetter-Swida, D Chrobak-Cmiel, P Szczygielska, M Leśniak, M Binek (2015). Characterization of Extended-Spectrum-β-Lactamases Produced by Escherichia coli Strains Isolated from Dogs in Poland. *Polish journal of microbiology*, 64(3), #Pages#

648. K Schaufler, T Semmler, LH Wieler, M Wöhrmann, R Baddam, N Ahmed, K Müller, A Kola, A Fruth, C Ewers, S Guenther (2016). Clonal spread and interspecies transmission of clinically relevant ESBL-producing Escherichia coli of ST410--another successful pandemic clone? *FEMS microbiology ecology*, 92(1), #Pages#

649. Guillard, T, de Jong, A, Limelette, A, Lebreil, A L, Madoux, J, de Champs, C (2016). Characterization of quinolone resistance mechanisms in Enterobacteriaceae recovered from diseased companion animals in Europe. *Veterinary microbiology*, 194(#issue#), 23

650. M Yousfi, A Touati, A Mairi, L Brasme, A Gharout-Sait, T Guillard, C De Champs (2016). Emergence of Carbapenemase-Producing Escherichia coli Isolated from Companion Animals in Algeria. *Microbial drug resistance (Larchmont, N.Y.)*, 22(4), #Pages#

651. Carvalho, A C, Barbosa, A V, Arais, L R, Ribeiro, P F, Carneiro, V C, Cerqueira, A M F (2016). Resistance patterns, ESBL genes, and genetic relatedness of Escherichia coli from dogs and owners. *Brazilian Journal of Microbiology: [publication of the Brazilian Society for Microbiology]*, 47(1), 150

652. Yousfi, Massilia, Mairi, Assia, Touati, Abdelaziz, Hassissene, Lila, Brasme, Lucien, Guillard, Thomas, De Champs, Christophe (2016). Extended spectrum beta-lactamase and plasmid mediated quinolone resistance in Escherichia coli fecal isolates from healthy companion animals in Algeria. *Journal of infection and chemotherapy : official journal of the Japan Society of Chemotherapy*, 22(7), 431

653. L Falgenhauer, C Imirzalioglu, H Ghosh, K Gwozdzinski, J Schmiedel, K Gentil, R Bauerfeind, P Kämpfer, H Seifert, GB Michael, S Schwarz, Y Pfeifer, G Werner, M Pietsch, U Roesler, B Guerra, J Fischer, H Sharp, A Käsbohrer, A Goesmann, K Hille, L Kreienbrock, T Chakraborty (2016). Circulation of clonal populations of fluoroquinolone-resistant CTX-M-15-producing Escherichia coli ST410 in humans and animals in Germany.

International journal of antimicrobial agents, 47(6), #Pages#

654. AK Siqueira, GB Michael, DF Domingos, MM Ferraz, MG Ribeiro, S Schwarz, DS Leite (2016). Diversity of class 1 and 2 integrons detected in Escherichia coli isolates from diseased and apparently healthy dogs. *Veterinary microbiology*, 194(), #Pages#

655. D Timofte, IE Maciuca, NJ Williams, A Wattret, V Schmidt (2016). Veterinary Hospital Dissemination of CTX-M-15 Extended-Spectrum Beta-Lactamase-Producing Escherichia coli ST410 in the United Kingdom. *Microbial drug resistance (Larchmont, N.Y.)*, 22(7), #Pages#

656. I Tuerena, NJ Williams, T Nuttall, G Pinchbeck (2016). Antimicrobial-resistant Escherichia coli in hospitalised companion animals and their hospital environment. *The Journal of small animal practice*, 57(7), #Pages#

657. Courtice, Rachel, Sniatynski, Michelle, Rubin, Joseph E (2016). Antimicrobial resistance and beta-lactamase production of Escherichia coli causing canine urinary tract infections: Passive surveillance of laboratory isolates in Saskatoon, Canada, 2014. *The Canadian veterinary journal = La revue veterinaire canadienne*, 57(11), 1166

658. A Karkaba, A Grinberg, J Benschop, E Pleydell (2017). Characterisation of extended-spectrum β-lactamase and AmpC β-lactamase-producing Enterobacteriaceae isolated from companion animals in New Zealand. *New Zealand veterinary journal*, 65(2), #Pages#

659. Guenther, Sebastian, Falgenhauer, Linda, Semmler, Torsten, Imirzalioglu, Can, Chakraborty, Trinad, Roesler, Uwe, Roschanski, Nicole (2017). Environmental emission of multiresistant Escherichia coli carrying the colistin resistance gene mcr-1 from German swine farms. *The Journal of antimicrobial chemotherapy*, 72(5), 1289

660. Akhtardanesh, Baharak, Ghanbarpour, Reza, Ganjalikhani, Sadaf, Gazanfari, Parisa (2016). Determination of antibiotic resistance genes in relation to phylogenetic background in Escherichia coli isolates from fecal samples of healthy pet cats in Kerman city. *Veterinary research forum : an international quarterly journal*, 7(4), 301

661. YS Chung, YK Park, YH Park, KT Park (2017). Probable secondary transmission of antimicrobial-resistant Escherichia coli between people living with and without pets. *The Journal of veterinary medical science*, 79(3), #Pages#

662. L Lei, Y Wang, S Schwarz, TR Walsh, Y Ou, Y Wu, M Li, Z Shen (2017). mcr-1 in Enterobacteriaceae from Companion Animals, Beijing, China, 2012-2016. *Emerging infectious diseases*, 23(4), #Pages#

663. S Li, J Liu, Y Zhou, Z Miao (2017). Characterization of ESBL-producing Escherichia coli recovered from companion dogs in Tai'an, China. *Journal of infection in developing countries*, 11(3), #Pages#

664. K Kawamura, T Sugawara, N Matsuo, K Hayashi, C Norizuki, K Tamai, T Kondo, Y Arakawa (2017). Spread of CTX-Type Extended-Spectrum β-Lactamase-Producing Escherichia coli Isolates of Epidemic Clone B2-O25-ST131 Among Dogs and Cats in Japan. *Microbial drug resistance (Larchmont, N.Y.)*, 23(8), #Pages#

665. Chung, Yeon Soo, Hu, Yoon Sung, Shin, Sook, Lim, Suk Kyung, Yang, Soo Jin, Park, Yong Ho, Park, Kun Taek (2017). Mechanisms of quinolone resistance in Escherichia coli isolated from companion animals, pet-owners, and non-pet-owners. *Journal of veterinary science*, 18(4), 449

666. Ö Aslantaş, EŞ Yilmaz (2017). Prevalence and molecular characterization of extended-spectrum β-lactamase (ESBL) and plasmidic AmpC β-lactamase (pAmpC) producing Escherichia coli in dogs. *The Journal of veterinary medical science*, 79(6), #Pages#

667. Liu, Xiaoqiang, Liu, Haixia, Li, Yinqian, Hao, Caiju (2017). Association between virulence profile and fluoroquinolone resistance in Escherichia coli isolated from dogs and cats in China. *Journal of infection in developing countries*, 11(4), 306

668. MT Brock, GC Fedderly, GI Borlee, MM Russell, LK Filipowska, DR Hyatt, RA Ferris, BR Borlee (2017). Pseudomonas aeruginosa variants obtained from veterinary clinical samples reveal a role for cyclic di-GMP in biofilm formation and colony morphology. *Microbiology (Reading, England)*, 163(11), #Pages#

669. M Yousfi, A Touati, A Muggeo, B Mira, B Asma, L Brasme, T Guillard, C de Champs (2018). Clonal dissemination of OXA-48-producing Enterobacter cloacae isolates from companion animals in Algeria. *Journal of global antimicrobial resistance*, 12(), #Pages#

670. Wagner, Sam, Lupolova, Nadejda, Gally, David L, Argyle, Sally A (2017). Convergence of plasmid architectures drives emergence of multi-drug resistance in a clonally diverse Escherichia coli population from a veterinary clinical care setting. *Veterinary microbiology*, 211(#issue#), 6

671. L Cui, L Lei, Y Lv, R Zhang, X Liu, M Li, F Zhang, Y Wang (2018). bla-producing multidrug-resistant Escherichia coli isolated from a companion dog in China. *Journal of global antimicrobial resistance*, 13(), #Pages#

672. Sato, Toyotaka, Harada, Kazuki, Usui, Masaru, Tsuyuki, Yuzo, Shiraishi, Tsukasa, Tamura, Yutaka, Yokota, Shin-Ichi (2018). Tigecycline Susceptibility of Klebsiella pneumoniae Complex and Escherichia coli Isolates from Companion Animals: The Prevalence of Tigecycline-Nonsusceptible K. pneumoniae Complex, Including Internationally Expanding Human Pathogenic Lineages. *Microbial drug resistance (Larchmont, N.Y.)*, 24(6), 860

673. Vingopoulou, Elpida I, Delis, Georgios A, Batzias, Georgios C, Kaltsogianni, Flora, Koutinas, Alexandros, Kristo, Ioulia, Pournaras, Spyros, Saridomichelakis, Manolis N, Siarkou, Victoria I (2018). Prevalence and mechanisms of resistance to fluoroquinolones in

Pseudomonas aeruginosa and Escherichia coli isolates recovered from dogs suffering from otitis in Greece. *Veterinary microbiology*, 213(#issue#), 102

674. Zhang, Pauline L C, Shen, Xiao, Chalmers, Gabhan, Reid-Smith, Richard J, Slavic, Durda, Dick, Hani, Boerlin, Patrick (2018). Prevalence and mechanisms of extended-spectrum cephalosporin resistance in clinical and fecal Enterobacteriaceae isolates from dogs in Ontario, Canada. *Veterinary microbiology*, 213(#issue#), 82

675. Derakhshandeh, Abdollah, Eraghi, Vida, Boroojeni, Azar Motamedi, Niaki, Malihe Akbarzadeh, Zare, Sahar, Naziri, Zahra (2018). Virulence factors, antibiotic resistance genes and genetic relatedness of commensal Escherichia coli isolates from dogs and their owners. *Microbial pathogenesis*, 116(#issue#), 241

676. B Liu, H Wu, Y Zhai, Z He, H Sun, T Cai, D He, J Liu, S Wang, Y Pan, L Yuan, G Hu (2018). Prevalence and molecular characterization of <i>oqxAB</i> in clinical <i>Escherichia coli</i> isolates from companion animals and humans in Henan Province, China. *Antimicrobial resistance and infection control*, 7(), #Pages#

677. de Jong, Anno, Muggeo, Anaelle, El Garch, Farid, Moyaert, Hilde, de Champs, Christophe, Guillard, Thomas (2018). Characterization of quinolone resistance mechanisms in Enterobacteriaceae isolated from companion animals in Europe (ComPath II study). *Veterinary microbiology*, 216(#issue#), 159

678. Y Maeyama, Y Taniguchi, W Hayashi, Y Ohsaki, S Osaka, S Koide, K Tamai, Y Nagano, Y Arakawa, N Nagano (2018). Prevalence of ESBL/AmpC genes and specific clones among the third-generation cephalosporin-resistant Enterobacteriaceae from canine and feline clinical specimens in Japan. *Veterinary microbiology*, 216(), #Pages#

679. Zogg, Anna Lena, Zurfluh, Katrin, Schmitt, Sarah, Nuesch-Inderbinen, Magdalena, Stephan, Roger (2018). Antimicrobial resistance, multilocus sequence types and virulence profiles of ESBL producing and non-ESBL producing uropathogenic Escherichia coli isolated from cats and dogs in Switzerland. *Veterinary microbiology*, 216(#issue#), 79

680. J Fernández, B Guerra, MR Rodicio (2018). Resistance to Carbapenems in Non-Typhoidal Salmonella enterica Serovars from Humans, Animals and Food. *Veterinary sciences*, 5(2), #Pages#

681. AL Zogg, S Simmen, K Zurfluh, R Stephan, SN Schmitt, M Nüesch-Inderbinen (2018). High Prevalence of Extended-Spectrum β-Lactamase Producing Enterobacteriaceae Among Clinical Isolates From Cats and Dogs Admitted to a Veterinary Hospital in Switzerland. *Frontiers in veterinary science*, 5(), #Pages#

682. Okpara, Evelyn O, Ojo, Olufemi E, Awoyomi, Olajoju J, Dipeolu, Morenike A, Oyekunle, Mufutau A, Schwarz, Stefan (2018). Antimicrobial usage and presence of extended-spectrum beta-lactamase-producing Enterobacteriaceae in animal-rearing households of selected rural and peri-urban communities. *Veterinary microbiology*,

218(#issue#), 31

683. A Liakopoulos, J Betts, R La Ragione, A van Essen-Zandbergen, D Ceccarelli, E Petinaki, CK Koutinas, DJ Mevius (2018). Occurrence and characterization of extended-spectrum cephalosporin-resistant Enterobacteriaceae in healthy household dogs in Greece. *Journal of medical microbiology*, 67(7), #Pages#

684. FP Sellera, MR Fernandes, R Ruiz, ACM Falleiros, FP Rodrigues, L Cerdeira, N Lincopan (2018). Identification of KPC-2-producing Escherichia coli in a companion animal: a new challenge for veterinary clinicians. *The Journal of antimicrobial chemotherapy*, 73(8), #Pages#

685. Behringer, Megan G, Boothe, Dawn M, Thungrat, Kamoltip (2018). Evaluation of a fluorescence resonance energy transfer quantitative polymerase chain reaction assay for identification of gyrA mutations conferring enrofloxacin resistance in canine urinary Escherichia coli isolates and canine urine specimens. *American journal of veterinary research*, 79(7), 755

686. LC Melo, C Oresco, L Leigue, HM Netto, PA Melville, NR Benites, E Saras, M Haenni, N Lincopan, JY Madec (2018). Prevalence and molecular features of ESBL/pAmpC-producing Enterobacteriaceae in healthy and diseased companion animals in Brazil. *Veterinary microbiology*, 221(), #Pages#

687. LeCuyer, Tessa E, Byrne, Barbara A, Daniels, Joshua B, Diaz-Campos, Dubraska V, Hammac, G Kenitra, Miller, Claire B, Besser, Thomas E, Davis, Margaret A (2018). Population Structure and Antimicrobial Resistance of Canine Uropathogenic Escherichia coli. *Journal of clinical microbiology*, 56(9), #Pages#

688. I Jamborova, BD Johnston, I Papousek, K Kachlikova, L Micenkova, C Clabots, A Skalova, K Chudejova, M Dolejska, I Literak, JR Johnson (2018). Extensive Genetic Commonality among Wildlife, Wastewater, Community, and Nosocomial Isolates of Escherichia coli Sequence Type 131 (<i>H</i>30R1 and <i>H</i>30Rx Subclones) That Carry <i>bla</i> or <i>bla</i>. *Antimicrobial agents and chemotherapy*, 62(10), #Pages#

689. JS Hong, W Song, HM Park, JY Oh, JC Chae, JI Han, SH Jeong (2019). First Detection of New Delhi Metallo-β-Lactamase-5-Producing Escherichia coli from Companion Animals in Korea. *Microbial drug resistance (Larchmont, N.Y.)*, 25(3), #Pages#

690. MM Silva, FP Sellera, MR Fernandes, Q Moura, F Garino, SS Azevedo, N Lincopan (2018). Genomic features of a highly virulent, ceftiofur-resistant, CTX-M-8-producing Escherichia coli ST224 causing fatal infection in a domestic cat. *Journal of global antimicrobial resistance*, 15(), #Pages#

691. U Kaspar, A von Lützau, A Schlattmann, U Roesler, R Köck, K Becker (2018). Zoonotic multidrug-resistant microorganisms among small companion animals in Germany. *PloS one*,

13(12), #Pages#

692. K Umeda, A Hase, M Matsuo, T Horimoto, J Ogasawara (2019). Prevalence and genetic characterization of cephalosporin-resistant Enterobacteriaceae among dogs and cats in an animal shelter. *Journal of medical microbiology*, 68(3), #Pages#

693. MV Rumi, J Mas, A Elena, L Cerdeira, ME Muñoz, N Lincopan, ÉR Gentilini, J Di Conza, G Gutkind (2019). Co-occurrence of clinically relevant β-lactamases and MCR-1 encoding genes in Escherichia coli from companion animals in Argentina. *Veterinary microbiology*, 230(), #Pages#

694. G Abbas, I Khan, M Mohsin, Sajjad-Ur-Rahman, T Younas, S Ali (2019). High rates of CTX-M group-1 extended-spectrum β-lactamases producing <i>Escherichia coli</i> from pets and their owners in Faisalabad, Pakistan. *Infection and drug resistance*, 12(), #Pages#

695. A Cormier, PLC Zhang, G Chalmers, JS Weese, A Deckert, M Mulvey, T McAllister, P Boerlin (2019). Diversity of CTX-M-positive Escherichia coli recovered from animals in Canada. *Veterinary microbiology*, 231(), #Pages#

696. A Mairi, A Pantel, F Ousalem, A Sotto, A Touati, JP Lavigne (2019). OXA-48-producing Enterobacterales in different ecological niches in Algeria: clonal expansion, plasmid characteristics and virulence traits. *The Journal of antimicrobial chemotherapy*, 74(7), #Pages#

697. Ceric, Olgica, Tyson, Gregory H, Goodman, Laura B, Mitchell, Patrick K, Zhang, Yan, Prarat, Melanie, Cui, Jing, Peak, Laura, Scaria, Joy, Antony, Linto, Thomas, Milton, Nemser, Sarah M, Anderson, Renee, Thachil, Anil J, Franklin-Guild, Rebecca J, Slavic, Durda, Bommineni, Yugendar R, Mohan, Shipra, Sanchez, Susan, Wilkes, Rebecca, Sahin, Orhan, Hendrix, G Kenitra, Lubbers, Brian, Reed, Deborah, Jenkins, Tracie, Roy, Alma, Paulsen, Daniel, Mani, Rinosh, Olsen, Karen, Pace, Lanny, Pulido, Martha, Jacob, Megan, Webb, Brett T, Dasgupta, Sarmila, Patil, Amar, Ramachandran, Akhilesh, Tewari, Deepanker, Thirumalapura, Nagaraja, Kelly, Donna J, Rankin, Shelley C, Lawhon, Sara D, Wu, Jing, Burbick, Claire R, Reimschuessel, Renate (2019). Enhancing the one health initiative by using whole genome sequencing to monitor antimicrobial resistance of animal pathogens: Vet-LIRN collaborative project with veterinary diagnostic laboratories in United States and Canada. *BMC veterinary research*, 15(1), 130

698. M Usui, A Kajino, M Kon, A Fukuda, T Sato, T Shirakawa, M Kawanishi, K Harada, C Nakajima, Y Suzuki, Y Tamura (2019). Prevalence of 16S rRNA methylases in Gram-negative bacteria derived from companion animals and livestock in Japan. *The Journal of veterinary medical science*, 81(6), #Pages#

699. A Karkaba, K Hill, J Benschop, E Pleydell, A Grinberg (2019). Carriage and population genetics of extended spectrum β-lactamase-producing Escherichia coli in cats and dogs in New Zealand. *Veterinary microbiology*, 233(), #Pages#

700. A Bortolami, F Zendri, EI Maciuca, A Wattret, C Ellis, V Schmidt, G Pinchbeck, D Timofte (2019). Diversity, Virulence, and Clinical Significance of Extended-Spectrum β-Lactamase- and pAmpC-Producing <i>Escherichia coli</i> From Companion Animals. *Frontiers in microbiology*, 10(), #Pages#

701. LC Melo, M Haenni, E Saras, M Duprilot, MH Nicolas-Chanoine, JY Madec (2019). Emergence of the C1-M27 cluster in ST131 Escherichia coli from companion animals in France. *The Journal of antimicrobial chemotherapy*, 74(10), #Pages#

702. LC Melo, M Haenni, E Saras, L Cerdeira, Q Moura, HJ Boulouis, JY Madec, N Lincopan (2019). Genomic characterisation of a multidrug-resistant TEM-52b extended-spectrum β-lactamase-positive Escherichia coli ST219 isolated from a cat in France. *Journal of global antimicrobial resistance*, 18(), #Pages#

703. Piccolo, Francesco Lo, Belas, Adriana, Foti, Maria, Fisichella, Vittorio, Marques, Catia, Pomba, Constanca (2020). Detection of multidrug resistance and extended-spectrum/plasmid-mediated AmpC beta-lactamase genes in Enterobacteriaceae isolates from diseased cats in Italy. *Journal of feline medicine and surgery*, 22(7), 613

704. Dupouy, Veronique, Abdelli, Mouni, Moyano, Gabriel, Arpaillange, Nathalie, Bibbal, Delphine, Cadiergues, Marie-Christine, Lopez-Pulin, Diego, Sayah-Jeanne, Sakina, de Gunzburg, Jean, Saint-Lu, Nathalie, Gonzalez-Zorn, Bruno, Andremont, Antoine, Bousquet-Melou, Alain (2019). Prevalence of Beta-Lactam and Quinolone/Fluoroquinolone

705. Resistance in Enterobacteriaceae From Dogs in France and Spain-Characterization of ESBL/pAmpC Isolates, Genes, and Conjugative Plasmids. *Frontiers in veterinary science*, 6(#issue#), 279

706. M Nüesch-Inderbinen, A Treier, K Zurfluh, R Stephan (2019). Raw meat-based diets for companion animals: a potential source of transmission of pathogenic and antimicrobial-resistant Enterobacteriaceae. *Royal Society open science*, 6(10), #Pages#

707. Loayza-Villa, Fernanda, Salinas, Liseth, Tijet, Nathalie, Villavicencio, Fernando, Tamayo, Rafael, Salas, Stephanie, Rivera, Ruth, Villacis, Jose, Satan, Carolina, Ushina, Liliana, Munoz, Olga, Zurita, Jeannette, Melano, Roberto, Reyes, Jorge, Trueba, Gabriel A (2020). Diverse Escherichia coli lineages from domestic animals carrying colistin resistance gene mcr-1 in an Ecuadorian household. *Journal of global antimicrobial resistance*, 22(#issue#), 63

708. M Mohamed, C Clabots, SB Porter, T Bender, P Thuras, JR Johnson (2020). Large Fecal Reservoir of Escherichia coli Sequence Type 131-H30 Subclone Strains That Are Shared Within Households and Resemble Clinical ST131-H30 Isolates. *The Journal of infectious diseases*, 221(10), #Pages#

709. Chen, Yanyun, Liu, Zhihai, Zhang, Yaru, Zhang, Zhenbiao, Lei, Lei, Xia, Zhaofei (2019). Increasing Prevalence of ESBL-Producing Multidrug Resistance Escherichia coli

From Diseased Pets in Beijing, China From 2012 to 2017. *Frontiers in microbiology*, 10(#issue#), 2852

710. SD Cole, L Peak, GH Tyson, R Reimschuessel, O Ceric, SC Rankin (2020). New Delhi Metallo-β-Lactamase-5-Producing Escherichia coli in Companion Animals, United States. *Emerging infectious diseases*, 26(2), #Pages#

711. JS Hong, W Song, SH Jeong (2020). Molecular Characteristics of NDM-5-Producing <i>Escherichia coli</i> from a Cat and a Dog in South Korea. *Microbial drug resistance (Larchmont, N.Y.)*, 26(8), #Pages#

712. Vega-Manriquez, X D, Ubiarco-Lopez, A, Verdugo-Rodriguez, A, Hernandez-Chinas, U, Navarro-Ocana, A, Ahumada-Cota, R E, Ramirez-Badillo, D, Hernandez-Diaz de Leon, N, Eslava, C A (2020). Pet dogs potential transmitters of pathogenic Escherichia coli with resistance to antimicrobials. *Archives of microbiology*, 202(5), 1173

713. Pepin-Puget, Line, El Garch, Farid, Bertrand, Xavier, Valot, Benoit, Hocquet, Didier (2020). Genome analysis of enterobacteriaceae with non-wild type susceptibility to third-generation cephalosporins recovered from diseased dogs and cats in Europe. *Veterinary microbiology*, 242(#issue#), 108601

714. M de Lagarde, C Larrieu, K Praud, N Lallier, A Trotereau, G Sallé, JM Fairbrother, C Schouler, B Doublet (2020). Spread of multidrug-resistant IncHI1 plasmids carrying ESBL gene bla and metabolism operon of prebiotic oligosaccharides in commensal Escherichia coli from healthy horses, France. *International journal of antimicrobial agents*, 55(6), #Pages#

715. Iseppi, Ramona, Di Cerbo, Alessandro, Messi, Patrizia, Sabia, Carla (2020). Antibiotic Resistance and Virulence Traits in Vancomycin-Resistant Enterococci (VRE) and Extended-Spectrum beta-Lactamase/AmpC-producing (ESBL/AmpC) Enterobacteriaceae from Humans and Pets. *Antibiotics (Basel, Switzerland)*, 9(4), #Pages#

716. V Mattioni Marchetti, I Bitar, A Mercato, E Nucleo, F Marchesini, M Mancinelli, P Prati, GS Scarsi, J Hrabak, L Pagani, M Fabbi, R Migliavacca (2020). Deadly Puppy Infection Caused by an MDR <i>Escherichia coli</i> O39 <i>bla</i> , <i>bla</i> , <i>bla</i> , and <i>aac(6)-Ib-cr</i> - Positive in a Breeding Kennel in Central Italy. *Frontiers in microbiology*, 11(), #Pages#

717. JS Hong, W Song, HM Park, JY Oh, JC Chae, S Jeong, SH Jeong (2020). Molecular Characterization of Fecal Extended-Spectrum β-Lactamase- and AmpC β-Lactamase-Producing <i>Escherichia coli</i> From Healthy Companion Animals and Cohabiting Humans in South Korea. *Frontiers in microbiology*, 11(), #Pages#

718. Sevilla, Eloisa, Mainar-Jaime, Raul C, Moreno, Bernardino, Martin-Burriel, Inmaculada, Morales, Mariano, Andres-Lasheras, Sara, Chirino-Trejo, Manuel, Badiola, Juan J, Bolea, Rosa (2020). Antimicrobial resistance among canine enteric Escherichia coli isolates and prevalence of attaching-effacing and extraintestinal pathogenic virulence factors in Spain.

Acta veterinaria Hungarica, 68(1), 1

719. Kidsley, Amanda K, O'Dea, Mark, Ebrahimie, Esmaeil, Mohammadi-Dehcheshmeh, Manijeh, Saputra, Sugiyono, Jordan, David, Johnson, James R, Gordon, David, Turni, Conny, Djordjevic, Steven P, Abraham, Sam, Trott, Darren
720. J (2020). Genomic analysis of fluoroquinolone-susceptible phylogenetic group B2 extraintestinal pathogenic Escherichia coli causing infections in cats. *Veterinary microbiology*, 245(#issue#), 108685

721. JS Schmidt, SP Kuster, A Nigg, V Dazio, M Brilhante, H Rohrbach, OJ Bernasconi, T Büdel, EI Campos-Madueno, S Gobeli Brawand, S Schuller, A Endimiani, V Perreten, B Willi (2020). Poor infection prevention and control standards are associated with environmental contamination with carbapenemase-producing Enterobacterales and other multidrug-resistant bacteria in Swiss companion animal clinics. *Antimicrobial resistance and infection control*, 9(1), #Pages#

722. Wang, Yining, Zhou, Jianhua, Li, Xuerui, Ma, Lina, Cao, Xiaoan, Hu, Wen, Zhao, Lu, Jing, Wenxian, Lan, Xi, Li, Youquan, Gong, Xiaowei, Chen, Qiwei, Stipkvits, Laszlo, Szathmary, Susan, Tarasiuk, Kazimierz, Pejsak, Zygmunt, Liu, Yongsheng (2020). Genetic diversity, antimicrobial resistance and extended-spectrum beta-lactamase type of Escherichia coli isolates from chicken, dog, pig and yak in Gansu and Qinghai Provinces, China. *Journal of global antimicrobial resistance*, 22(#issue#), 726

723. Ngbede, Emmanuel O, Poudel, Anil, Kalalah, Anwar, Yang, Yi, Adekanmbi, Folasade, Adikwu, Alex A, Adamu, Andrew M, Mamfe, Levi M, Daniel, Salem T, Useh, Nicodemus M, Kwaga, Jacob K P, Adah, Mohammed I, Kelly, Patrick, Butaye, Patrick, Wang, Chengming (2020). Identification of mobile colistin resistance genes (mcr-1.1, mcr-5 and mcr-8.1) in Enterobacteriaceae and Alcaligenes faecalis of human and animal origin, Nigeria. *International journal of antimicrobial agents*, 56(3), 106108

724. AK Kidsley, RT White, SA Beatson, S Saputra, MA Schembri, D Gordon, JR Johnson, M O'Dea, JL Mollinger, S Abraham, DJ Trott (2020). Companion Animals Are Spillover Hosts of the Multidrug-Resistant Human Extraintestinal <i>Escherichia coli</i> Pandemic Clones ST131 and ST1193. *Frontiers in microbiology*, 11(), #Pages#

725. V Zechner, D Sofka, P Paulsen, F Hilbert (2020). Antimicrobial Resistance in <i>Escherichia coli</i> and Resistance Genes in Coliphages from a Small Animal Clinic and in a Patient Dog with Chronic Urinary Tract Infection. *Antibiotics (Basel, Switzerland)*, 9(10), #Pages#

726. LJ Toombs-Ruane, J Benschop, NP French, PJ Biggs, AC Midwinter, JC Marshall, M Chan, D Drinković, A Fayaz, MG Baker, J Douwes, MG Roberts, SA Burgess (2020). Carriage of Extended-Spectrum-Beta-Lactamase- and AmpC Beta-Lactamase-Producing Escherichia coli Strains from Humans and Pets in the Same Households. *Applied and environmental microbiology*, 86(24), #Pages#

727. MJ Rodríguez-González, MA Jiménez-Pearson, F Duarte, T Poklepovich, J Campos, LN Araya-Sánchez, M Chirino-Trejo, E Barquero-Calvo (2020). Multidrug-Resistant CTX-M and CMY-2 Producing <i>Escherichia coli</i> Isolated from Healthy Household Dogs from the Great Metropolitan Area, Costa Rica. *Microbial drug resistance (Larchmont, N.Y.)*, 26(11), #Pages#

728. E Massella, CJ Reid, ML Cummins, K Anantanawat, T Zingali, A Serraino, S Piva, F Giacometti, SP Djordjevic (2020). Snapshot Study of Whole Genome Sequences of <i>Escherichia coli</i> from Healthy Companion Animals, Livestock, Wildlife, Humans and Food in Italy. *Antibiotics (Basel, Switzerland)*, 9(11), #Pages#

729. RAP Sfaciotte, L Parussolo, FD Melo, P Wildemann, G Bordignon, ND Israel, M Leitzke, SR Wosiacki, FZ Salbego, UM da Costa, SM Ferraz (2021). Identification and Characterization of Multidrug-Resistant Extended-Spectrum Beta-Lactamase-Producing Bacteria from Healthy and Diseased Dogs and Cats Admitted to a Veterinary Hospital in Brazil. *Microbial drug resistance (Larchmont, N.Y.)*, 27(6), #Pages#

730. J Wang, YB Xia, XY Huang, Y Wang, LC Lv, QQ Lin, MY Yi, PL Lu, JH Liu, ZL Zeng (2021). Emergence of <i>bla</i> in Enterobacteriaceae Isolates from Companion Animals in Guangzhou, China. *Microbial drug resistance (Larchmont, N.Y.)*, 27(6), #Pages#

731. Manchester, Alison C, Dogan, Belgin, Guo, Yongli, Simpson, Kenneth W (2021). Escherichia coli-associated granulomatous colitis in dogs treated according to antimicrobial susceptibility profiling. *Journal of veterinary internal medicine*, 35(1), 150

732. Lei, Lei, Wang, Yongqiang, He, Junjia, Cai, Chang, Liu, Qingzhi, Yang, Dawei, Zou, Zhiyu, Shi, Lingyu, Jia, Jianqin, Wang, Yang, Walsh, Timothy R, Shen, Jianzhong, Zhong, Yougang (2021). Prevalence and risk analysis of mobile colistin resistance and extended-spectrum beta-lactamase genes carriage in pet dogs and their owners: a population based cross-sectional study. *Emerging microbes & infections*, 10(1), 242

733. S Bandyopadhyay, J Banerjee, D Bhattacharyya, R Tudu, I Samanta, P Dandapat, PK Nanda, AK Das, B Mondal, S Batabyal, TK Dutta (2021). Companion Animals Emerged as an Important Reservoir of Carbapenem-Resistant Enterobacteriaceae: A Report from India. *Current microbiology*, 78(3), #Pages#

734. V Dazio, A Nigg, JS Schmidt, M Brilhante, N Mauri, SP Kuster, SG Brawand, G Schüpbach-Regula, B Willi, A Endimiani, V Perreten, S Schuller (2021). Acquisition and carriage of multidrug-resistant organisms in dogs and cats presented to small animal practices and clinics in Switzerland. *Journal of veterinary internal medicine*, 35(2), #Pages#

735. K Schmitt, SP Kuster, K Zurfluh, RS Jud, JE Sykes, R Stephan, B Willi (2021). Transmission Chains of Extended-Spectrum Beta-Lactamase-Producing Enterobacteriaceae at the Companion Animal Veterinary Clinic-Household Interface. *Antibiotics (Basel, Switzerland)*, 10(2), #Pages#

736. M Salgado-Caxito, JA Benavides, JM Munita, L Rivas, P García, FJP Listoni, AI Moreno-Switt, AC Paes (2021). Risk factors associated with faecal carriage of extended-spectrum cephalosporin-resistant Escherichia coli among dogs in Southeast Brazil. *Preventive veterinary medicine*, 190(), #Pages#

737. R Courtice, M Sniatynski, JE Rubin (2021). Characterization of antimicrobial-resistant Escherichia coli causing urinary tract infections in dogs: Passive surveillance in Saskatchewan, Canada 2014 to 2018. *Journal of veterinary internal medicine*, 35(3), #Pages#

738. Ilbeigi, Kayhan, Askari Badouei, Mahdi, Vaezi, Hossein, Zaheri, Hassan, Aghasharif, Sina, Kafshdouzan, Khatereh (2021). Molecular survey of mcr1 and mcr2 plasmid mediated colistin resistance genes in Escherichia coli isolates of animal origin in Iran. *BMC research notes*, 14(1), 107

739. SR Shin, SM Noh, WK Jung, S Shin, YK Park, DC Moon, SK Lim, YH Park, KT Park (2021). Characterization of Extended-Spectrum β-Lactamase-Producing and AmpC β-Lactamase-Producing <i>Enterobacterales</i> Isolated from Companion Animals in Korea. *Antibiotics (Basel, Switzerland)*, 10(3), #Pages#

740. Carvalho, Isabel, Safia Chenouf, Nadia, Cunha, Rita, Martins, Carla, Pimenta, Paulo, Pereira, Ana Raquel, Martinez-Alvarez, Sandra, Ramos, Sonia, Silva, Vanessa, Igrejas, Gilberto, Torres, Carmen, Poeta, Patricia (2021). Antimicrobial Resistance Genes and Diversity of Clones among ESBL- and Acquired AmpC-Producing Escherichia coli Isolated from Fecal Samples of Healthy and Sick Cats in Portugal. *Antibiotics (Basel, Switzerland)*, 10(3), #Pages#

741. P Alba, R Taddei, G Cordaro, MC Fontana, E Toschi, P Gaibani, I Marani, A Giacomi, EL Diaconu, M Iurescia, V Carfora, A Franco (2021). Carbapenemase IncF-borne bla gene in the E. coli ST167 high-risk clone from canine clinical infection, Italy. *Veterinary microbiology*, 256(), #Pages#

742. M Salgado-Caxito, JA Benavides, AD Adell, AC Paes, AI Moreno-Switt (2021). Global prevalence and molecular characterization of extended-spectrum β-lactamase producing-<i>Escherichia coli</i> in dogs and cats - A scoping review and meta-analysis. *One health (Amsterdam, Netherlands)*, 12(), #Pages#

743. L Karahutová, R Mandelík, D Bujňáková (2021). Antibiotic Resistant and Biofilm-Associated <i>Escherichia coli</i> Isolates from Diarrheic and Healthy Dogs. *Microorganisms*, 9(6), #Pages#

744. Carvalho, Isabel, Cunha, Rita, Martins, Carla, Martinez-Alvarez, Sandra, Safia Chenouf, Nadia, Pimenta, Paulo, Pereira, Ana Raquel, Ramos, Sonia, Sadi, Madjid, Martins, Angela, Facanha, Jorge, Rabbi, Fazle, Capita, Rosa, Alonso-Calleja, Carlos, de Lurdes Nunes Enes Dapkevicius, Maria, Igrejas, Gilberto, Torres, Carmen, Poeta, Patricia (2021). Antimicrobial Resistance Genes and Diversity of Clones among Faecal ESBL-Producing Escherichia coli Isolated from Healthy and Sick Dogs Living in Portugal. *Antibiotics (Basel, Switzerland)*,

10(8), #Pages#

745. N Formenti, A Grassi, G Parisio, C Romeo, F Guarneri, L Birbes, A Pitozzi, F Scali, AM Maisano, MB Boniotti, P Pasquali, GL Alborali (2021). Extended-Spectrum-β-Lactamase- and AmpC-Producing <i>Escherichia coli</i> in Domestic Dogs: Spread, Characterisation and Associated Risk Factors. *Antibiotics (Basel, Switzerland)*, 10(10), #Pages#

746. MG Wang, C Fang, KD Liu, LL Wang, RY Sun, RM Zhang, LX Fang, J Sun, YH Liu, XP Liao (2022). Transmission and molecular characteristics of blaNDM-producing Escherichia coli between companion animals and their healthcare providers in Guangzhou, China. *The Journal of antimicrobial chemotherapy*, 77(2), #Pages#

747. N Nittayasut, J Yindee, P Boonkham, T Yata, N Suanpairintr, P Chanchaithong (2021). Multiple and High-Risk Clones of Extended-Spectrum Cephalosporin-Resistant and <i>bla</i>-Harbouring Uropathogenic <i>Escherichia coli</i> from Cats and Dogs in Thailand. *Antibiotics (Basel, Switzerland)*, 10(11), #Pages#

748. D Li, ER Wyrsch, P Elankumaran, M Dolejska, MS Marenda, GF Browning, RN Bushell, J McKinnon, PR Chowdhury, N Hitchick, N Miller, E Donner, B Drigo, D Baker, IG Charles, T Kudinha, VM Jarocki, SP Djordjevic (2021). Genomic comparisons of <i>Escherichia coli</i> ST131 from Australia. *Microbial genomics*, 7(12), #Pages#

749. FL Liu, NL Kuan, KS Yeh (2021). Presence of the Extended-Spectrum-β-Lactamase and Plasmid-Mediated AmpC-Encoding Genes in <i>Escherichia coli</i> from Companion Animals-A Study from a University-Based Veterinary Hospital in Taipei, Taiwan. *Antibiotics (Basel, Switzerland)*, 10(12), #Pages#

750. A Jonker, J Gouws, ER Kapp, A Henning (2022). Isolation of a multidrug-resistant <i>Escherichia coli</i> pathotype Stx2:Cnf1:Cnf2:Eae as a potential cause of hemorrhagic diarrhea and secondary septicemia in a dog. *Journal of veterinary diagnostic investigation : official publication of the American Association of Veterinary Laboratory Diagnosticians, Inc*, 34(2), #Pages#

751. P Elankumaran, GF Browning, MS Marenda, CJ Reid, SP Djordjevic (2022). Close genetic linkage between human and companion animal extraintestinal pathogenic <i>Escherichia coli</i> ST127. *Current research in microbial sciences*, 3(), #Pages#

752. Algammal, Abdelazeem M, El-Tarabili, Reham M, Alfifi, Khyreyah J, Al-Otaibi, Amenah S, Hashem, Marwa E Abo, El-Maghraby, Mamdouh M, Mahmoud, Ahmed E (2022). Virulence determinant and antimicrobial resistance traits of Emerging MDR Shiga toxigenic E. coli in diarrheic dogs. *AMB Express*, 12(1), 34

753. JMD Silva, J Menezes, C Marques, CF Pomba (2022). Companion Animals-An Overlooked and Misdiagnosed Reservoir of Carbapenem Resistance. *Antibiotics (Basel, Switzerland)*, 11(4), #Pages#

754. Naziri, Zahra, Poormaleknia, Meisam, Ghaedi Oliyaei, Azar (2022). Risk of sharing resistant bacteria and/or resistance elements between dogs and their owners. *BMC veterinary research*, 18(1), 203

755. X Kuang, R Yang, X Ye, J Sun, X Liao, Y Liu, Y Yu (2022). NDM-5-Producing <i>Escherichia coli</i> Co-Harboring <i>mcr-1</i> Gene in Companion Animals in China. *Animals : an open access journal from MDPI*, 12(10), #Pages#

756. V Johansson, S Nykäsenoja, AL Myllyniemi, H Rossow, A Heikinheimo (2022). Genomic characterization of ESBL/AmpC-producing and high-risk clonal lineages of Escherichia coli and Klebsiella pneumoniae in imported dogs with shelter and stray background. *Journal of global antimicrobial resistance*, 30(), #Pages#

757. G Gruel, D Couvin, S Guyomard-Rabenirina, G Arlet, JC Bambou, M Pot, X Roy, A Talarmin, B Tressieres, S Ferdinand, S Breurec (2022). High Prevalence of <i>bla</i> /IncI1-Iγ/ST3 Plasmids in Extended-Spectrum β-Lactamase-Producing <i>Escherichia coli</i> Isolates Collected From Domestic Animals in Guadeloupe (French West Indies). *Frontiers in microbiology*, 13(), #Pages#

758. G Batisti Biffignandi, A Piazza, F Marchesini, P Prati, A Mercato, A Abu Alshaar, G Andreoli, D Sassera, R Migliavacca (2022). Genomic Characterization of an O101:H9-ST167 NDM-5-Producing Escherichia coli Strain from a Kitten in Italy. *Microbiology spectrum*, 10(3), #Pages#

759. P Elankumaran, ML Cummins, GF Browning, MS Marenda, CJ Reid, SP Djordjevic (2022). Genomic and Temporal Trends in Canine ExPEC Reflect Those of Human ExPEC. *Microbiology spectrum*, 10(3), #Pages#

760. Fernandes, Vera, Cunha, Eva, Nunes, Telmo, Silva, Elisabete, Tavares, Luis, Mateus, Luisa, Oliveira, Manuela (2022). Antimicrobial Resistance of Clinical and Commensal Escherichia coli Canine Isolates: Profile Characterization and Comparison of Antimicrobial Susceptibility Results According to Different Guidelines. *Veterinary sciences*, 9(6), #Pages#

761. L Cui, X Zhao, R Li, Y Han, G Hao, G Wang, S Sun (2022). Companion Animals as Potential Reservoirs of Antibiotic Resistant Diarrheagenic <i>Escherichia coli</i> in Shandong, China. *Antibiotics (Basel, Switzerland)*, 11(6), #Pages#

762. TJ Johnson, JR Armstrong, B Johnston, I Merino-Velasco, I Jamborova, RS Singer, JR Johnson, JB Bender (2022). Occurrence and potential transmission of extended-spectrum beta-lactamase-producing extraintestinal pathogenic and enteropathogenic Escherichia coli in domestic dog faeces from Minnesota. *Zoonoses and public health*, 69(7), #Pages#

763. Sato, Toyotaka, Harada, Kazuki, Usui, Masaru, Yokota, Shin-Ichi, Horiuchi, Motohiro (2022). Colistin Susceptibility in Companion Animal-Derived Escherichia coli, Klebsiella spp., and Enterobacter spp. in Japan: Frequent Isolation of Colistin-Resistant Enterobacter

cloacae Complex. *Frontiers in cellular and infection microbiology*, 12(#issue#), 946841

764. Nguyen, Phuong Thi Lan, Tran, Hung Thi Mai, Tran, Hai Anh, Pham, Thai Duy, Luong, Tan Minh, Nguyen, Thanh Ha, Nguyen, Lien Thi Phuong, Nguyen, Tho Thi Thi, Hoang, Ha Thi An, Nguyen, Chi, Tran, Duong Nhu, Dang, Anh Duc, Suzuki, Masato, Le, Thanh Viet, Banuls, Anne-Laure, Choisy, Marc, Van Doorn, Rogier H, Tran, Huy Hoang (2022). Carriage of Plasmid-Mediated Colistin Resistance-1-Positive Escherichia coli in Humans, Animals, and Environment on Farms in Vietnam. *The American journal of tropical medicine and hygiene*, 107(1), 65

765. A Banerjee, S Pal, P Goswami, K Batabyal, SN Joardar, S Dey, DP Isore, TK Dutta, S Bandyopadhyay, I Samanta (2022). Docking analysis of circulating CTX-M variants in multi-drug resistant, beta-lactamase and biofilm-producing E. coli isolated from pet animals and backyard livestock. *Microbial pathogenesis*, 170(), #Pages#

766. Naziri, Zahra, Derakhshandeh, Abdollah, Zare, Sahar, Akbarzadeh Niaki, Malihe, Motamedi Boroojeni, Azar, Eraghi, Vida, Shirmohamadi Sosfad, Abolfazl (2023). Identification of faecal Escherichia coli isolates with similar patterns of virulence and antimicrobial resistance genes in dogs and their owners. *Veterinary medicine and science*, 9(1), 126

767. Menezes, Juliana, Moreira da Silva, Joana, Frosini, Sian-Marie, Loeffler, Anette, Weese, Scott, Perreten, Vincent, Schwarz, Stefan, Telo da Gama, Luis, Amaral, Andreia Jesus, Pomba, Constanca (2022). mcr-1 colistin resistance gene sharing between Escherichia coli from cohabiting dogs and humans, Lisbon, Portugal, 2018 to 2020. *Euro surveillance : bulletin Europeen sur les maladies transmissibles = European communicable disease bulletin*, 27(44), #Pages#

768. Zhou, Yifan, Ji, Xue, Liang, Bing, Jiang, Bowen, Li, Yan, Yuan, Tingyv, Zhu, Lingwei, Liu, Jun, Guo, Xuejun, Sun, Yang (2022). Antimicrobial Resistance and Prevalence of Extended Spectrum beta-Lactamase-Producing Escherichia coli from Dogs and Cats in Northeastern China from 2012 to 2021. *Antibiotics (Basel, Switzerland)*, 11(11), #Pages#

769. Nguyen, Phuong Thi Lan, Ngo, Thi Hong Hanh, Tran, Thi Mai Hung, Vu, Thi Ngoc Bich, Le, Viet Thanh, Tran, Hai Anh, Pham, Duy Thai, Nguyen, Ha Thanh, Tran, Dieu Linh, Nguyen, Thi Phuong Lien, Nguyen, Thi Thi Tho, Tran, Nhu Duong, Dang, Duc Anh, Banuls, Anne-Laure, Choisy, Marc, van Doorn, H Rogier, Suzuki, Masato, Tran, Huy Hoang (2022). Genomic epidemiological analysis of mcr-1-harboring Escherichia coli collected from livestock settings in Vietnam. *Frontiers in veterinary science*, 9(#issue#), 1034610

770. Gargano, Valeria, Gambino, Delia, Orefice, Tiziana, Cirincione, Roberta, Castelli, Germano, Bruno, Federica, Interrante, Paolo, Pizzo, Mariangela, Spada, Eva, Proverbio, Daniela, Vicari, Domenico, Salgado-Caxito, Marilia, Benavides, Julio A, Cassata, Giovanni (2022). Can Stray Cats Be Reservoirs of Antimicrobial Resistance?. *Veterinary sciences*, 9(11), #Pages#

771. R Tudu, J Banerjee, M Habib, S Bandyopadhyay, S Biswas, SS Kesh, A Maity, S Batabyal, S Polley (2022). Prevalence and molecular characterization of extended-spectrum β-lactamase (ESBL) producing <i>Escherichia coli</i> isolated from dogs suffering from diarrhea in and around Kolkata. *Iranian journal of veterinary research*, 23(3), #Pages#

772. G Ortiz-Díez, RL Mengíbar, MC Turrientes, MB Artigao, RL Gallifa, AM Tello, CF Pérez, TA Santiago (2023). Prevalence, incidence and risk factors for acquisition and colonization of extended-spectrum beta-lactamase- and carbapenemase-producing Enterobacteriaceae from dogs attended at a veterinary hospital in Spain. *Comparative immunology, microbiology and infectious diseases*, 92(), #Pages#

773. M Piekar, VE Álvarez, C Knecht, C Leguina, N García Allende, L Carrera Páez, AS Gambino, A González Machuca, J Campos, B Fox, E Carpio, A Aguilar, FM Alonso, L Fernández Canigia, MP Quiroga, D Centrón (2023). Genomic data reveals the emergence of the co-occurrence of bla and bla in an Escherichia coli ST648 strain isolated from rectal swab within the framework of hospital surveillance. *Journal of global antimicrobial resistance*, 32(), #Pages#

774. Fayez, Mahmoud, Elmoslemany, Ahmed, Al Romaihi, Ahmad A, Azzawi, Abdulfattah Y, Almubarak, Abdullah, Elsohaby, Ibrahim (2023). Prevalence and Risk Factors Associated with Multidrug Resistance and Extended-Spectrum beta-lactamase Producing E. coli Isolated from Healthy and Diseased Cats. *Antibiotics (Basel, Switzerland)*, 12(2), #Pages#

775. KW Seo (2023). Development of a Method for the Fast Detection of Extended-Spectrum β-Lactamase- and Plasmid-Mediated AmpC β-Lactamase-Producing <i>Escherichia coli</i> and <i>Klebsiella pneumoniae</i> from Dogs and Cats in the USA. *Animals : an open access journal from MDPI*, 13(4), #Pages#

776. L Teng, M Feng, S Liao, Z Zheng, C Jia, X Zhou, RB Nambiar, Z Ma, M Yue (2023). A Cross-Sectional Study of Companion Animal-Derived Multidrug-Resistant Escherichia coli in Hangzhou, China. *Microbiology spectrum*, 11(2), #Pages#

777. Tong, Yin-Chao, Zhang, Yi-Ning, Li, Peng-Cheng, Cao, Ya-Li, Ding, Dong-Zhao, Yang, Yang, Lin, Qing-Yi, Gao, Yi-Nuo, Sun, Shao-Qiang, Fan, Yun-Peng, Liu, Ying-Qiu, Qing, Su-Zhu, Ma, Wu-Ren, Zhang, Wei-Min (2023). Detection of antibiotic-resistant canine origin Escherichia coli and the synergistic effect of magnolol in reducing the resistance of multidrug-resistant Escherichia coli. *Frontiers in veterinary science*, 10(#issue#), 1104812

778. JH Choi, MS Ali, BY Moon, HY Kang, SJ Kim, HJ Song, AF Mechesso, DC Moon, SK Lim (2023). Prevalence and Characterization of Extended-Spectrum β-Lactamase-Producing <i>Escherichia coli</i> Isolated from Dogs and Cats in South Korea. *Antibiotics (Basel, Switzerland)*, 12(4), #Pages#

779. Collineau, Lucie, Bourely, Clemence, Rousset, Leo, Berger-Carbonne, Anne, Ploy, Marie-Cecile, Pulcini, Celine, Colomb-Cotinat, Melanie (2023). Towards One Health surveillance of antibiotic resistance: characterisation and mapping of existing programmes in

humans, animals, food and the environment in France, 2021. *Euro surveillance : bulletin Europeen sur les maladies transmissibles = European communicable disease bulletin*, 28(22), #Pages#

780. Tong, Yin-Chao, Li, Peng-Cheng, Yang, Yang, Lin, Qing-Yi, Liu, Jin-Tong, Gao, Yi-Nuo, Zhang, Yi-Ning, Jin, Shuo, Qing, Su-Zhu, Xing, Fu-Shan, Fan, Yun-Peng, Liu, Ying-Qiu, Wang, Wei-Ling, Zhang, Wei-Min, Ma, Wu-Ren (2023). Detection of Antibiotic Resistance in Feline-Origin ESBL Escherichia coli from Different Areas of China and the Resistance Elimination of Garlic Oil to Cefquinome on ESBL E. coli. *International journal of molecular sciences*, 24(11), #Pages#

781. Ballash, Gregory A, Diaz-Campos, Dubraska, van Balen, Joany C, Mollenkopf, Dixie F, Wittum, Thomas E (2023). Previous Antibiotic Exposure Reshapes the Population Structure of Infecting Uropathogenic Escherichia coli Strains by Selecting for Antibiotic Resistance over Urovirulence. *Microbiology spectrum*, #volume#(#issue#), e0524222

782. PB Stege, J Hordijk, AKS Sandholt, AL Zomer, MC Viveen, MRC Rogers, M Salomons, JA Wagenaar, L Mughini-Gras, RJL Willems, FL Paganelli (2023). Gut Colonization by ESBL-Producing Escherichia coli in Dogs Is Associated with a Distinct Microbiome and Resistome Composition. *Microbiology spectrum*, (), #Pages#

783. J Menezes, SM Frosini, A Belas, C Marques, JM da Silva, AJ Amaral, A Loeffler, C Pomba (2023). Longitudinal study of ESBL/AmpC-producing Enterobacterales strains sharing between cohabiting healthy companion animals and humans in Portugal and in the United Kingdom. *European journal of clinical microbiology & infectious diseases : official publication of the European Society of Clinical Microbiology*, (), #Pages#

784. Nittayasut, N., Yindee, J., Boonkham, P., Yata, T., Suanpairintr, N., Chanchaithong, P. (2021). Multiple and high-risk clones of extended-spectrum cephalosporin-resistant and blaNDM-5-harbouring uropathogenic Escherichia coli from cats and dogs in Thailand. *Antibiotics*, 10(11), #Pages#

785. Ashraf, A. A. E. T., Ahmed, A. A. M., Nermin, E., Emad, E. E. M., Wedad, A. (2022). Characterization of some antimicrobial genes of Escherichia coli isolated from pet animals and human with urinary tract infections. *Benha Veterinary Medical Journal*, 43(1), 75

786. Sfaciotte, R. A. P., Parussolo, L., Melo, F. D., Wildemann, P., Bordignon, G., Israel, N. D., Leitzke, M., Wosiacki, S. R., Salbego, F. Z., Costa, U. M. da, Ferraz, S. M. (2021). Identification and characterization of multidrug-resistant extended-spectrum beta-lactamase-producing bacteria from healthy and diseased dogs and cats admitted to a veterinary hospital in Brazil. *Microbial Drug Resistance*, 27(6), 855

787. Asai, T., Usui, M., Sugiyama, M., Andoh, M. (2022). A survey of antimicrobial-resistant Escherichia coli prevalence in wild mammals in Japan using antimicrobial-containing media. *Journal of Veterinary Medical Science*, 84(12), 1645

788. Yi MengYing, Wang Jing, Lu PeiLan, Huang XinYi, Xia YingBi, Huang JiaWei, Yan JieCong, Zhuang ZiLin, Liu JianHua (2019). Antimicrobial resistance and resistance genes of Escherichia coli from pets. *Journal of South China Agricultural University*, 40(6), 15

789. Buyukcangaz, E., Khider, M., Sen, A., Cengiz, M., Sonal, S. (2019). Detection of Shiga-toxin producing E. coli (STEC), enteropathogenic E. coli (EPEC) and enterotoxigenic E. coli (ETEC) from animals by M-PCR. *Veteriner Fakültesi Dergisi, Uludag Üniversitesi*, 38(2), 88

790. Huang YiHsuan, Kuan NanLing, Yeh KuangSheng (2020). Characteristics of extended-spectrum β-lactamase-producing Escherichia coli from dogs and cats admitted to a veterinary teaching hospital in Taipei, Taiwan from 2014 to 2017. *Frontiers in Veterinary Science,* 6(July), #Pages#

791. Yang ShouShen, Wang JiaHui, Lin Min, Zeng XiaoFei, Qiu MinHua, Chen WenYan, He YuQin, Lin ZhaoSheng, Lin WeiMing (2019). Prevalence and drug-resistance mechanism of ESBLs in Escherichia coli isolates of pet origins. *Chinese Journal of Zoonoses*, 35(12), 1110

792. Muhammad Mustapha, Parveen Goel, Dinesh Mittal, Maan, S. (2020). Molecular investigations of tetracycline resistance genes in Escherichia coli strains from dogs affected with urinary tract infections. *Alexandria Journal of Veterinary Sciences*, 64(1), 17

793. Younis, K., Baddour, M., Ibrahim, M. S. (2015). Detection of diarrheagenic Escherichia coli in pet animals and its antibiotic resistance in Alexandria governorate. *Alexandria Journal of Veterinary Sciences*, 45(#issue#), 113

794. Abhiroop Banerjee, Surajit Pal, Purba Goswami, Kunal Batabyal, Joardar, S. N., Samir Dey, Isore, D. P., Dutta, T. K., Samiran Bandyopadhyay, Indranil Samanta (2022). Docking analysis of circulating CTX-M variants in multi-drug resistant, beta-lactamase and biofilm-producing E. coli isolated from pet animals and backyard livestock. *Microbial Pathogenesis*, 170(#issue#), #Pages#

795. Kuang Xu, Yang RunShi, Ye XinQing, Sun Jian, Liao XiaoPing, Liu YaHong, Yu Yang (2022). NDM-5-producing Escherichia coli co-harboring mcr-1 gene in companion animals in China. *Animals*, 12(10), #Pages#

796. Melo, L. C., Boisson, M. N. G., Saras, E., Médaille, C., Boulouis, H. J., Madec, J. Y., Haenni, M. (2017). OXA-48-producing ST372 Escherichia coli in a French dog. *Journal of Antimicrobial Chemotherapy*, 72(4), 1256

797. Torkan, S., Bahadoranian, M. A., Khamesipour, F., Anyanwu, M. U. (2016). Detection of virulence and antimicrobial resistance genes in Escherichia coli isolates from diarrhoiec dogs in Iran. *Archivos de Medicina Veterinaria*, 48(2), 181

798. Gruel, G., Couvin, D., Guyomard-Rabenirina, S., Arlet, G., Bambou, J. C., Pot, M., Roy, X., Talarmin, A., Tressieres, B., Ferdinand, S., Breurec, S. (2022). High prevalence of

blaCTXM-1/IncI1-Iγ/ST3 plasmids in extended-spectrum β-lactamase-producing Escherichia coli isolates collected from domestic animals in Guadeloupe (French West Indies). *Frontiers in Microbiology*, 13(May), #Pages#

799. Zogg, A. L., Simmen, S., Zurfluh, K., Stephan, R., Schmitt, S. N., Nüesch-Inderbinen, M. (2018). High prevalence of extended-spectrum β-lactamase producing Enterobacteriaceae among clinical isolates from cats and dogs admitted to a veterinary hospital in Switzerland. *Frontiers in Veterinary Science*, 5(March), 62

800. Liu FangLing, Kuan NanLing, Yeh KuangSheng (2021). Presence of the extended-spectrum-β-lactamase and plasmid-mediated AmpC-encoding genes in Escherichia coli from companion animals-a study from a university-based veterinary hospital in Taipei, Taiwan. *Antibiotics*, 10(12), #Pages#

801. Vinocur, F., Nievas, V. F., Garassino, B. J., Ramos Rodríguez, S., Giacoboni, G. I., Moredo, F. A. (2021). Retrospective study to determine the presence of colistin-resistant and extended-spectrum β-lactamase producing Escherichia coli, in animals for human consumption and pets in the province of Buenos Aires, Argentina. *Veterinaria Cuyana*, 15(#issue#), 15

802. Dahmen, S., Haenni, M., Châtre, P., Madec, J. Y. (2013). Characterization of blaCTX-M IncFII plasmids and clones of Escherichia coli from pets in France. *Journal of Antimicrobial Chemotherapy*, 68(12), 2797

803. Maaland, M. G., Mo, S. S., Schwarz, S., Guardabassi, L. (2015). In vitro assessment of chloramphenicol and florfenicol as second-line antimicrobial agents in dogs. *Journal of Veterinary Pharmacology and Therapeutics*, 38(5), 443

804. Hong JunSung, Song Wonkeun, Park HeeMyung, Oh JaeYoung, Chae, J. C., Jeong SeRi, Jeong, S. H. (2020). Molecular characterization of fecal extended-spectrum β-lactamase- and AmpC β-lactamase-producing Escherichia coli from healthy companion animals and cohabiting humans in South Korea. *Frontiers in Microbiology*, 11(April), #Pages#

805. ŞahÌntürk, P., Arslan, E., Büyükcangaz, E., Sonal, S., Şen, A., Ersoy, F., Webber, M. A., Piddock, L. J. V., Cengİz, M. (2016). High level fluoroquinolone resistance in Escherichia coli isolated from animals in Turkey is due to multiple mechanisms. *Turkish Journal of Veterinary & Animal Sciences*, 40(2), 214

806. Puño-Sarmiento, J., Medeiros, L., Chiconi, C., Martins, F., Pelayo, J., Rocha, S., Blanco, J., Blanco, M., Zanutto, M., Kobayashi, R., Nakazato, G. (2013). Detection of diarrheagenic Escherichia coli strains isolated from dogs and cats in Brazil. *Veterinary Microbiology*, 166(3/4), 676

807. Kobs, V. C., Medeiros, F. de, Fernandes, P. P., Deglmann, R. C., Fontana, H., Cardoso, B., Sano, E., Esposito, F., Sellera, F. P., Lincopan, N., França, P. H. C. de (2023). Healthcare-associated NDM-1-producing Enterobacter hormaechei subsp. xiangfangensis

clone ST136 emerging as pathogen of companion animals in Brazil. *Journal of Antimicrobial Chemotherapy*, 78(6), 1553

808. Silva, J. M. da, Menezes, J., Marques, C., Pomba, C. F. (2022). Companion animals-an overlooked and misdiagnosed reservoir of carbapenem resistance. *Antibiotics*, 11(4), #Pages#

809. Ewers, C., Bethe, A., Stamm, I., Grobbel, M., Kopp, P. A., Guerra, B., Stubbe, M., Doi, Y. H., Zong ZhiYong, Kola, A., Schaufler, K., Semmler, T., Fruth, A., Wieler, L. H., Guenther, S. (2014). CTX-M-15-D-ST648 Escherichia coli from companion animals and horses: another pandemic clone combining multiresistance and extraintestinal virulence? *Journal of Antimicrobial Chemotherapy*, 69(5), 1224

810. Pomba, C., López-Cerero, L., Bellido, M., Serrano, L., Belas, A., Couto, N., Cavaco-Silva, P., Rodríguez-Baño, J., Pascual, A. (2014). Within-lineage variability of ST131 Escherichia coli isolates from humans and companion animals in the south of Europe. *Journal of Antimicrobial Chemotherapy*, 69(1), 271

811. Smet, A., Vaes, R., Praud, K., Doublet, B., Daminet, S., Cloeckaert, A., Haesebrouck, F. (2014). New broad-spectrum β-lactamases emerging among Enterobacteriaceae from healthy cats and dogs: a public health concern? *International Journal of Antimicrobial Agents*, 44(1), 81

812. Bortolaia, V., Hansen, K. H., Nielsen, C. A., Fritsche, T. R., Guardabassi, L. (2014). High diversity of plasmids harbouring blaCMY-2 among clinical Escherichia coli isolates from humans and companion animals in the upper Midwestern USA. *Journal of Antimicrobial Chemotherapy*, 69(6), 1492

813. Moreno Anzola, M. A., Castillo Huertas, M. A., Ferrebuz, A. J., Osorio Zambrano, W. F., Torres Caycedo, M. I., López Velandia, D. P. (2018). Bacterial resistance in small animals, risk potential for human health. *REDVET*, 19(2), 021819

814. Bogaerts, P., Huang TeDin, Bouchahrouf, W., Bauraing, C., Berhin, C., El-Garch, F., Glupczynski, Y. (2015). Characterization of ESBL- and AmpC-producing Enterobacteriaceae from diseased companion animals in Europe. *Microbial Drug Resistance*, 21(6), 643

815. Dahmen, S., Haenni, M., Madec, J. Y. (2012). IncI1/ST3 plasmids contribute to the dissemination of the blaCTX-M-1 gene in Escherichia coli from several animal species in France. *Journal of Antimicrobial Chemotherapy*, 67(12), 3011

816. Zhu HengQian, Liao XiaoPing, Chen ChaoXi, Wang XiuMei, Sun Jian, Sun Ying, Li Liang, Zhang MeiJun, Liu YaHong (2010). Detection of plasmid-mediated quinolone resistance in clinical isolates of Escherichia coli from pet animals. *Scientia Agricultura Sinica*, 43(16), 3447

817. Cengİz, M., Buyukcangaz, E., Arslan, E., Mat, B., Sahİnturk, P., Sonal, S., Gocmen, H., Sen, A. (2012). Molecular characterisation of quinolone resistance in Escherichia coli from animals in Turkey. *Veterinary Record*, 171(6), 155

818. Pomba, C., Fonseca, J. D. da, Baptista, B. C., Correia, J. D., Martínez-Martínez, L. (2009). Detection of the pandemic O25-ST131 human virulent Escherichia coli CTX-M-15-producing clone harboring the qnrB2 and aac(6′)-Ib-cr genes in a dog. *Antimicrobial Agents and Chemotherapy*, 53(1), 327

819. Carattoli, A., Lovari, S., Franco, A., Cordaro, G., Matteo, P. di, Battisti, A. (2005). Extended-spectrum β-lactamases in Escherichia coli isolated from dogs and cats in Rome, Italy, from 2001 to 2003. *Antimicrobial Agents and Chemotherapy*, 49(2), 833

820. Shaheen, B. W., Nayak, R., Boothe, D. M. (2013). Emergence of a New Delhi metallo-β-lactamase (NDM-1)-encoding gene in clinical Escherichia coli isolates recovered from companion animals in the United States. *Antimicrobial Agents and Chemotherapy*, 57(6), 2902

821. Schink, A. K., Kadlec, K., Kaspar, H., Mankertz, J., Schwarz, S. (2013). Analysis of extended-spectrum-β-lactamase-producing Escherichia coli isolates collected in the GERM-Vet monitoring programme. *Journal of Antimicrobial Chemotherapy*, 68(8), 1741

822. Belas, A., Marques, C., Menezes, J., Gama, L. T. da, Cavaco-Silva, P., Pomba, C. (2022). ESBL/pAmpC-producing Escherichia coli causing urinary tract infections in non-related companion animals and humans. *Antibiotics*, 11(5), #Pages#

823. Tudu, R., Banerjee, J., Md Habib, Bandyopadhyay, S., Biswas, S., Kesh, S. S., Maity, A., Batabyal, S., Polley, S. (2022). Prevalence and molecular characterization of extended-spectrum β-lactamase (ESBL) producing Escherichia coli isolated from dogs suffering from diarrhea in and around Kolkata. *Iranian Journal of Veterinary Research*, 23(3), 237

824. Wang MinGe, Fang Chang, Liu KaiDi, Wang LinLin, Sun RuanYang, Zhang RongMin, Fang LiangXing, Sun Jian, Liu YaHong, Liao XiaoPing (2021). Transmission and molecular characteristics of blaNDM-producing Escherichia coli between companion animals and their healthcare providers in Guangzhou, China. *Journal of Antimicrobial Chemotherapy*, 77(2), 351

825. Johnson, T. J., Armstrong, J. R., Johnston, B., Merino-Velasco, I., Jamborova, I., Singer, R. S., Johnson, J. R., Bender, J. B. (2022). Occurrence and potential transmission of extended-spectrum beta-lactamase-producing extraintestinal pathogenic and enteropathogenic Escherichia coli in domestic dog faeces from Minnesota. *Zoonoses and Public Health*, 69(7), 888

826. Courtice, R., Sniatynski, M., Rubin, J. E. (2021). Characterization of antimicrobial-resistant Escherichia coli causing urinary tract infections in dogs: passive surveillance in

Saskatchewan, Canada 2014 to 2018. *Journal of Veterinary Internal Medicine*, 35(3), 1389

827. Massella, E., Reid, C. J., Cummins, M. L., Anantanawat, K., Zingali, T., Serraino, A., Piva, S., Giacometti, F., Djordjevic, S. P. (2020). Snapshot study of whole genome sequences of Escherichia coli from healthy companion animals, livestock, wildlife, humans and food in Italy. *Antibiotics*, 9(11), #Pages#

828. Jonker, A., Gouws, J., Kapp, E. R., Henning, A. (2022). Isolation of a multidrug-resistant Escherichia coli pathotype stx2:CNF1:CNF2:eae as a potential cause of hemorrhagic diarrhea and secondary septicemia in a dog. *Journal of Veterinary Diagnostic Investigation*, 34(2), 339

829. Tuerena, I., Williams, N. J., Nuttall, T., Pinchbeck, G. (2016). Antimicrobial-resistant Escherichia coli in hospitalised companion animals and their hospital environment. *Journal of Small Animal Practice*, 57(7), 339

830. Kobs, V. C., Valdez, R. E., Medeiros, F. de, Fernandes, P. P., Deglmann, R. C., Gern, R. M. M., França, P. H. C. (2020). mcr-1-carrying Enterobacteriaceae isolated from companion animals in Brazil. *Pesquisa Veterinária Brasileira*, 40(9), 690

831. Marchetti, V. M., Bitar, I., Mercato, A., Nucleo, E., Marchesini, F., Mancinelli, M., Prati, P., Scarsi, G. S., Hrabak, J., Pagani, L., Fabbi, M., Migliavacca, R. (2020). Deadly puppy infection caused by an MDR Escherichia coli O39 blactx-m-15, blacmy-2, bladha-1, and aac(6)-Ib-cr - positive in a breeding kennel in Central Italy. *Frontiers in Microbiology*, 11(April), #Pages#

832. Moon DongChan, Mechesso, A. F., Kang HeeYoung, Kim SuJeong, Choi JiHyun, Kim MiHyun, Song HyunJu, Yoon SoonSeek, Lim SukKyung (2020). First report of an Escherichia coli strain carrying the colistin resistance determinant mcr-1 from a dog in South Korea. *Antibiotics*, 9(11), #Pages#

833. Thongratsakul, S., Poolkhet, C., Amavisit, P., Sato, T., Fukuda, A., Usui, M., Tamura, Y. (2019). Antimicrobial resistance and STEC virulence genes of Escherichia coli isolated from non-diarrheic and diarrheic dogs at a veterinary teaching hospital in Thailand. *Southeast Asian Journal of Tropical Medicine and Public Health*, 50(4), 708

834. Moon DongChan, Choi JiHyun, Boby NaiLa, Kang HeeYoung, Kim SuJeong, Song HyunJu, Park HoSung, Gil MinChan, Yoon SoonSeek, Lim SukKyung (2022). Bacterial prevalence in skin, urine, diarrheal stool, and respiratory samples from dogs. *Microorganisms*, 10(8), #Pages#

835. Schmitt, K., Biggel, M., Stephan, R., Willi, B. (2022). Massive spread of OXA-48 carbapenemase-producing Enterobacteriaceae in the environment of a Swiss companion animal clinic. *Antibiotics*, 11(2), #Pages#

836. Platell, J. L., Cobbold, R. N., Johnson, J. R., Heisig, A., Heisig, P., Clabots, C., Kuskowski, M. A., Trott, D. J. (2011). Commonality among fluoroquinolone-resistant sequence type ST131 extraintestinal Escherichia coli isolates from humans and companion animals in Australia. *Antimicrobial Agents and Chemotherapy*, 55(8), 3782

837. Chung YeonSoo, Park YoungKyung, Park YongHo, Park KunTaek (2017). Probable secondary transmission of antimicrobial-resistant Escherichia coli between people living with and without pets. *Journal of Veterinary Medical Science*, 79(3), 486

838. Elankumaran, P., Cummins, M. L., Browning, G. F., Marenda, M. S., Reid, C. J., Djordjevic, S. P. (2022). Genomic and temporal trends in canine ExPEC reflect those of human ExPEC. *Microbiology Spectrum*, 10(3), #Pages#

839. Agnoletti, F., Brunetta, R., Drigo, I., Tonon, E., Deotto, S., D'Este, L., Barberio, A., Cocchi, M., Ustulin, M., Corrò, M., Dellamaria, D., Trevisiol, K., Mazzolini, E. (2018). Genotyping and antimicrobial resistance of Escherichia coli ESBL/AmpC-producers isolated from dogs in northeastern Italy [Conference poster]. *XVIII Congresso Nazionale S.I.Di.L.V., Perugia (PG), Italia, 7-9 Novembre 2018*, #volume#(#issue#), 229

840. Belas, A., Salazar, A. S., Gama, L. T. da, Couto, N., Pomba, C. (2014). Risk factors for faecal colonisation with Escherichia coli producing extended-spectrum and plasmid-mediated AmpC β-lactamases in dogs. *Veterinary Record*, 175(8), 202

841. Alba, P., Taddei, R., Cordaro, G., Fontana, M. C., Toschi, E., Gaibani, P., Marani, I., Giacomi, A., Diaconu, E. L., Iurescia, M., Carfora, V., Franco, A. (2021). Carbapenemase IncF-borne blaNDM-5 gene in the E. coli ST167 high-risk clone from canine clinical infection, Italy. *Veterinary Microbiology*, 256(#issue#), #Pages#

842. Prati, P., Spalla, M., Scarsi, G., Marchetti, V. M., Caltagirone, M., Mercato, A., Novazzi, F., Nucleo, E., Migliavacca, R., Pagani, L., Fabbi, M. (2018). Fatal infection in bulldog puppies caused by Escherichia coli ST58, O141, multi antibiotic resistant, CTX-M-15, CMY-2, DHA-1 and AAC (6′)-IB-cr positive, in a farm in central Italy [Conference poster]. *XVIII Congresso Nazionale S.I.Di.L.V., Perugia (PG), Italia, 7-9 Novembre 2018*, #volume#(#issue#), 191

843. Siqueira, A. K., Michael, G. B., Domingos, D. F., Ferraz, M. M. G., Ribeiro, M. G., Schwarz, S., Leite, D. S. (2016). Diversity of class 1 and 2 integrons detected in Escherichia coli isolates from diseased and apparently healthy dogs. *Veterinary Microbiology*, 194(#issue#), 79

844. Abraham, S., Wong HuiSan, Turnidge, J., Johnson, J. R., Trott, D. J. (2014). Carbapenemase-producing bacteria in companion animals: a public health concern on the horizon. *Journal of Antimicrobial Chemotherapy*, 69(5), 1155

845. Platell, J. L., Cobbold, R. N., Johnson, J. R., Trott, D. J. (2010). Clonal group distribution of fluoroquinolone-resistant Escherichia coli among humans and companion animals in

Australia. *Journal of Antimicrobial Chemotherapy*, 65(9), 1936

846. Schaufler, K. A. C. (2016). Molecular analysis of ESBL-producing Escherichia coli from different habitats discloses insights into phylogeny, clonal relationships and transmission scenarios. *#journal#*, #volume#(#issue#), v + 63 pp.

847. Ji Xue, Wang Rong, Sun Yang, Zhao XiangSheng, Guo XueJun, Liu Jun, Zhu LingWei, Feng ShuZhang (2014). Molecular characterization of pathogenetic Escherichia coli isolated from dogs. *Chinese Journal of Veterinary Science*, 34(4), 578

848. Jamborova, I., Johnston, B. D., Papousek, I., Kachlikova, K., Micenkova, L., Clabots, C., Skalova, A., Chudejova, K., Dolejska, M., Literak, I., Johnson, J. R. (2018). Extensive genetic commonality among wildlife, wastewater, community, and nosocomial isolates of Escherichia coli sequence type 131 (H30R1 and H30Rx subclones) that carry blaCTX-M-27 or blaCTX-M-15. *Antimicrobial Agents and Chemotherapy*, 62(10), e00519

849. Salehi, T. Z., Badouei, M. A., Gohari, I. M. (2011). Molecular detection and antibacterial susceptibility of enteropathogenic Escherichia coli (EPEC) and shigatoxigenic Escherichia coli (STEC) strains isolated from healthy and diarrhoeic dogs. *Comparative Clinical Pathology*, 20(6), 585

850. Fang LiangXing, Sun Jian, Li Liang, Deng Hui, Huang Ting, Yang, Q. E., Li Xue, Chen MuYa, Liao XiaoPing, Liu YaHong (2015). Dissemination of the chromosomally encoded CMY-2 cephalosporinase gene in Escherichia coli isolated from animals. *International Journal of Antimicrobial Agents*, 46(2), 209

851. Wieler, L. H., Ewers, C., Guenther, S., Walther, B., Lübke-Becker, A. (2011). Methicillin-resistant staphylococci (MRS) and extended-spectrum beta-lactamases (ESBL)-producing Enterobacteriaceae in companion animals: nosocomial infections as one reason for the rising prevalence of these potential zoonotic pathogens in clinical samples. *International Journal of Medical Microbiology*, 301(8), 635

852. Ewers, C., Grobbel, M., Stamm, I., Kopp, P. A., Diehl, I., Semmler, T., Fruth, A., Beutlich, J., Guerra, B., Wieler, L. H., Guenther, S. (2010). Emergence of human pandemic O25:H4-ST131 CTX-M-15 extended-spectrum-β-lactamase-producing Escherichia coli among companion animals. *Journal of Antimicrobial Chemotherapy*, 65(4), 651

853. Platell, J. L., Trott, D. J., Johnson, J. R., Heisig, P., Heisig, A., Clabots, C. R., Johnston, B., Cobbold, R. N. (2012). Prominence of an O75 clonal group (clonal complex 14) among non-ST131 fluoroquinolone-resistant Escherichia coli causing extraintestinal infections in humans and dogs in Australia. *Antimicrobial Agents and Chemotherapy*, 56(7), 3898

854. Maluta, R. P., Stella, A. E., Riccardi, K., Rigobelo, E. C., Marin, J. M., Carvalho, M. B., Ávila, F. A. de (2012). Phenotypical characterization and adhesin identification in Escherichia coli strains isolated from dogs with urinary tract infections. *Brazilian Journal of*

Microbiology, 43(1), 375

855. Gibson, J. S., Cobbold, R. N., Trott, D. J. (2010). Characterization of multidrug-resistant Escherichia coli isolated from extraintestinal clinical infections in animals. *Journal of Medical Microbiology*, 59(5), 592

856. Choi, Ji-Hyun, Ali, Md Sekendar, Moon, Bo-Youn, Kang, Hee-Young, Kim, Su-Jeong, Song, Hyun-Ju, Mechesso, Abraham Fikru, Moon, Dong-Chan, Lim, Suk-Kyung (2023). Prevalence and Characterization of Extended-Spectrum β-Lactamase-Producing Escherichia coli Isolated from Dogs and Cats in South Korea. *Antibiotics (Basel, Switzerland)*, 12(4), #Pages#

857. Teng, Lin, Feng, Mengyao, Liao, Sihao, Zheng, Zhijie, Jia, Chenghao, Zhou, Xin, Nambiar, Reshma B, Ma, Zhengxin, Yue, Min (2023). A Cross-Sectional Study of Companion Animal-Derived Multidrug-Resistant Escherichia coli in Hangzhou, China. *Microbiology spectrum*, #volume#(#issue#), 1

858. Seo, Kwang-Won (2023). Development of a Method for the Fast Detection of Extended-Spectrum β-Lactamase- and Plasmid-Mediated AmpC β-Lactamase-Producing Escherichia coli and Klebsiella pneumoniae from Dogs and Cats in the USA. *Animals : an open access journal from MDPI*, 13(4), #Pages#

859. Joddha, Harshrajsinh B, Mathakiya, Rafiyuddin A, Joshi, Kuldip V, Khant, Ravindra B, Golaviya, Akash V, Hinsu, Ankit T, Desai, Mansi R, Jakhesara, Subhash J, Koringa, Prakash G (2023). Profiling of Antimicrobial Resistance Genes and Integron from Escherichia coli Isolates Using Whole Genome Sequencing *Genes*, 14(6), 1212

860. Mandujano, Antonio, Cortés-Espinosa, Diana Verónica, Vásquez-Villanueva, José, Guel, Paulina, Rivera, Gildardo, Juárez-Rendón, Karina, Cruz-Pulido, Wendy Lizeth, Aguilera-Arreola, Guadalupe, Guerrero, Abraham, Bocanegra-García, Virgilio, Martínez-Vázquez, Ana Verónica (2023). Extended-Spectrum β-Lactamase-Producing Escherichia coli Isolated from Food-Producing Animals in Tamaulipas, Mexico *Antibiotics*, 12(6), 1010

861. Alonso-García, Isaac, Vázquez-Ucha, Juan Carlos, Martínez-Guitián, Marta, Lasarte-Monterrubio, Cristina, Rodríguez-Pallares, Salud, Camacho-Zamora, Pablo, Rumbo-Feal, Soraya, Aja-Macaya, Pablo, González-Pinto, Lucía, Outeda-García, Michelle, Maceiras, Romina, Guijarro-Sánchez, Paula, Muíño-Andrade, María José, Fernández-González, Ana, Oviaño, Marina, González-Bello, Concepción, Arca-Suárez, Jorge, Beceiro, Alejandro, Bou, Germán (2023). Interplay between OXA-10 β-Lactamase Production and Low Outer-Membrane Permeability in Carbapenem Resistance in Enterobacterales *Antibiotics*, 12(6), 999

862. Chien-Hao Tseng, Chia-Wei, Liu, Po-Yu, Liu (2023). Extended-Spectrum β-Lactamases (ESBL) Producing Bacteria in Animals *Antibiotics*, 12(4), 661

863. Bastos, Taís Silvino, Camilla Mariane Menezes Souza, Legendre, Héloïse, Nadège, Richard, Pilla, Rachel, Suchodolski, Jan S, de Oliveira, Simone Gisele, Achraf Adib Lesaux, Ananda Portella Félix (2023). Effect of Yeast Saccharomyces cerevisiae as a Probiotic on Diet Digestibility, Fermentative Metabolites, and Composition and Functional Potential of the Fecal Microbiota of Dogs Submitted to an Abrupt Dietary Change *Microorganisms*, 11(2), 506

864. Primeau, Courtney A, Bharat, Amrita, Nicol Janecko, Carson, Carolee A, Mulvey, Michael, Reid-Smith, Richard, McEwen, Scott, McWhirter, Jennifer E, Parmley, E Jane (2023). Integrated surveillance of extended-spectrum beta-lactamase (ESBL)-producing Salmonella and Escherichia coli from humans and animal species raised for human consumption in Canada from 2012 to 2017 *Epidemiology and Infection*, 151(#issue#), #Pages#

865. Núñez-Samudio, Virginia, Pimentel-Peralta, Gumercindo, De La Cruz, Alexis, Landires, Iván (2023). Genetic Diversity and New Sequence Types of Escherichia coli Coharboring β-Lactamases and PMQR Genes Isolated from Domestic Dogs in Central Panama *Genes*, 14(1), 73

866. Ibrahim, Delveen R, Dodd, Christine E R, Stekel, Dov J, Meshioye, Remilekun T, Diggle, Mathew, Lister, Michelle, Hobman, Jon L (2023). Multidrug-Resistant ESBL-Producing E. coli in Clinical Samples from the UK *Antibiotics*, 12(1), 169

867. Karakaya, Emre, Aydin, Fuat, Kayman, Tuba, Abay, Seçil (2023). Escherichia coli in different animal feces: phylotypes and virulence genes *World Journal of Microbiology and Biotechnology*, 39(1), 14

868. Asai, Tetsuo, Sugiyama, Michiyo, Omatsu, Tsutomu, Yoshikawa, Masato, Minamoto, Toshifumi (2022). Isolation of extended-spectrum β-lactamase-producing Escherichia coli from Japanese red fox (Vulpes vulpes japonica) *MicrobiologyOpen*, 11(5), #Pages#

869. Sun, L, Meng, N, Wang, Z, Hong, J, Dai, Y, Wang, J, Jiao, X (2022). Genomic Characterization of ESBL/AmpC-Producing Escherichia coli in Stray Dogs Sheltered in Yangzhou, China *Infection and Drug Resistance*, 15(#issue#), 7741

870. Tóth, Adrienn Gréta, Tóth, Imre, Rózsa, Bernadett, Dubecz, Attila, Patai, Árpád V, Németh, Tibor, Kaplan, Selçuk, Kovács, Eszter Gabriella, Makrai, László, Solymosi, Norbert (2022). Canine Saliva as a Possible Source of Antimicrobial Resistance Genes *Antibiotics*, 11(11), 1490

871. Debergh, Hanne, Maex, Margo, Garcia-Graells, Cristina, Boland, Cécile, Saulmont, Marc, Koenraad Van Hoorde, Saegerman, Claude (2022). First Belgian Report of Ertapenem Resistance in an ST11 Klebsiella Pneumoniae Strain Isolated from a Dog Carrying blaSCO-1 and blaDHA-1 Combined with Permeability Defects *Antibiotics*, 11(9), 1253

872. Andreea Paula Cozma, Rimbu, Cristina Mihaela, Zendri, Flavia, Maciuca, Iuliana Elena, Timofte, Dorina (2022). Clonal Dissemination of Extended-Spectrum Cephalosporin-Resistant Enterobacterales between Dogs and Humans in Households and Animal Shelters of Romania *Antibiotics*, 11(9), 1242

873. Shnaiderman-Torban, Anat, Navon-Venezia, Shiri, Baron, Hadar, Abu-Ahmad, Wiessam, Arielly, Haya, Gal Zizelski Valenci, Nissan, Israel, Paitan, Yossi, Steinman, Amir (2022). Prevalence and Molecular Characterization of Extended-Spectrum β-Lactamase Producing Enterobacterales in Healthy Community Dogs in Israel *Antibiotics*, 11(8), 1069

874. Cheng-Hung, Lai, Yu-Chan, Ma, Wei-Yau Shia, Yu-Ling, Hsieh, Chao-Min, Wang (2022). Risk Factors for Antimicrobial Resistance of Staphylococcus Species Isolated from Dogs with Superficial Pyoderma and Their Owners *Veterinary Sciences*, 9(7), 306

875. Chen, Wenxin, Liu, Zhihong, Lin, Hongguang, Yang, Jie, Liu, Ting, Zheng, Jiaomei, Long, Xueming, Sun, Zhiliang, Li, Jiyun, Chen, Xiaojun (2022). Occurrence of blaNDM-1-Positive Providencia spp. in a Pig Farm of China *Antibiotics*, 11(6), 713

876. Cui, Lulu, Zhao, Xiaonan, Li, Ruibo, Han, Yu, Hao, Guijuan, Wang, Guisheng, Sun, Shuhong (2022). Companion Animals as Potential Reservoirs of Antibiotic Resistant Diarrheagenic Escherichia coli in Shandong, China *Antibiotics*, 11(6), 828

877. Melgarejo, Tonatiuh, Sharp, Nathan, Krumbeck, Janina A, Wu, Guangxi, Kim, Young J, Linde, Annika (2022). The Urinary Resistome of Clinically Healthy Companion Dogs: Potential One Health Implications *Antibiotics*, 11(6), 780

878. Medina-Pizzali, Maria Luisa, Venkatesh, Apoorva, Riveros, Maribel, Cuicapuza, Diego, Salmon-Mulanovich, Gabriela, Mäusezahl, Daniel, Hartinger, Stella M (2022). Whole-Genome Characterisation of ESBL-Producing E. coli Isolated from Drinking Water and Dog Faeces from Rural Andean Households in Peru *Antibiotics*, 11(5), 692

879. Lin, Teng, Liao, Sihao, Zhou, Xin, Jia, Chenghao, Feng, Mengyao, Pan, Hang, Ma, Zhengxin, Yue, Min (2022). Prevalence and Genomic Investigation of Multidrug-Resistant Salmonella Isolates from Companion Animals in Hangzhou, China *Antibiotics*, 11(5), 625

880. Silva, Vanessa, Caniça, Manuela, Manageiro, Vera, Newton Verbisck, Tejedor-Junco, María Teresa, González-Martin, Margarita, Corbera, Juan Alberto, Poeta, Patrícia, Igrejas, Gilberto (2022). Staphylococcus aureus and Methicillin-Resistant Coagulase-Negative Staphylococci in Nostrils and Buccal Mucosa of Healthy Camels Used for Recreational Purposes *Animals*, 12(10), 1255

881. Abdelwahab, Ghada Elderdiri, Hassan Zackaria Ali Ishag, Zulaikha Mohamed Al Hammadi, Saeed Mohamed S Al Yammahi, Mohd Faoruk Bin Mohd Yusof, Muna Sayed Y Al Yassi, Shaikha Saeed A Al neyadi, Asma Mohammed A Al Mansoori, Fawzia Hassan A Al Hamadi, Ibtesam Abdullah S Al Hamadi, Mohamed Ali Abdalla Al Hosani, Salama Suhail Mohammed Al Muhairi (2022). Antibiotics Resistance in Escherichia coli Isolated

from Livestock in the Emirate of Abu Dhabi, UAE, 2014–2019 *International Journal of Microbiology*, 2022(#issue#), #Pages#

882. Ramos, Carolina Pantuzza, Carolina Yumi Iceri Kamei, Flávia Mello Viegas, Jonata de Melo Barbieri, Reis Cunha, João Luís, Yaovi Mahuton Gildas Hounmanou, Coura, Fernanda Morcatti, Jordana Almeida Santana, Faria Lobato, Francisco Carlos, Anders Miki Bojesen, Silveira Silva, Rodrigo Otávio (2022). Fecal Shedding of Multidrug Resistant Escherichia coli Isolates in Dogs Fed with Raw Meat-Based Diets in Brazil *Antibiotics*, 11(4), 534

883. Soe Yu Naing, Hordijk, Joost, Duim, Birgitta, Broens, Els M, Linda van der Graaf-van Bloois, Rossen, John W, Robben, Joris H, Leendertse, Masja, Wagenaar, Jaap A, Zomer, Aldert L (2022). Genomic Investigation of Two Acinetobacter baumannii Outbreaks in a Veterinary Intensive Care Unit in The Netherlands *Pathogens*, 11(2), 123

884. Feßler, Andrea T, Scholtzek, Anissa D, Schug, Angela R, Kohn, Barbara, Weingart, Christiane, Schink, Anne-Kathrin, Bethe, Astrid, Lübke-Becker, Antina, Schwarz, Stefan (2022). Antimicrobial and Biocide Resistance among Feline and Canine Staphylococcus aureus and Staphylococcus pseudintermedius Isolates from Diagnostic Submissions *Antibiotics*, 11(2), 127

885. Johnston, Brian D, Thuras, Paul, Porter, Stephen B, Anacker, Melissa, VonBank, Brittany, Vagnone, Paula Snippes, Witwer, Medora, Castanheira, Mariana, Johnson, James R (2021). Global molecular epidemiology of carbapenem-resistant Escherichia coli (2002-2017). *European journal of clinical microbiology & infectious diseases : official publication of the European Society of Clinical Microbiology*, #volume#(#issue#), #Pages#

886. Hattab, Jasmine, Mosca, Francesco, Di Francesco, Cristina Esmeralda, Aste, Giovanni, Marruchella, Giuseppe, Guardiani, Pierluigi, Tiscar, Pietro Giorgio (2021). Occurrence, antimicrobial susceptibility, and pathogenic factors of Pseudomonas aeruginosa in canine clinical samples *Veterinary World*, 14(4), 978

887. Bandyopadhyay Samiran, Banerjee Jaydeep, Bhattacharyya Debaraj, Tudu Rahul, Samanta Indranil, Dandapat Premanshu, Nanda, Pramod K, Das, Arun K, Mondal Bimalendu, Batabyal Subhasis, Dutta, Tapan K (2021). Companion Animals Emerged as an Important Reservoir of Carbapenem-Resistant Enterobacteriaceae: A Report from India *Current Microbiology*, 78(3), 1006

888. Salinas, Liseth, Loayza, Fernanda, Cárdenas, Paúl, Saraiva, Carlos, Johnson, Timothy J, Amato, Heather, Graham, Jay P, Trueba, Gabriel (2021). Environmental Spread of Extended Spectrum Beta-Lactamase (ESBL) Producing Escherichia coli and ESBL Genes among Children and Domestic Animals in Ecuador *Environmental Health Perspectives (Online)*, 129(2), #Pages#

889. Mayo Yasugi, Hatoya, Shingo, Motooka, Daisuke, Matsumoto, Yuki, Shimamura, Shunsuke, Tani, Hiroyuki, Furuya, Masaru, Mie, Keiichiro, Miyake, Masami, Nakamura, Shota, Shimada, Terumasa (2021). Whole-genome analyses of extended-spectrum or AmpC

β-lactamase-producing Escherichia coli isolates from companion dogs in Japan *PLoS One*, 16(2), #Pages#

890. Bourne, Judith Anne (2021). Comparative Genotypic and Phenotypic Characteristics of Human Associated Extraintestinal Escherichia Coli Isolated from Cats and Dogs *#journal#*, #volume#(#issue#), 226

891. Sabença, Carolina, Igrejas, Gilberto, Poeta, Patrícia, Robin, Frédéric, Bonnet, Richard, Beyrouthy, Racha (2021). Multidrug Resistance Dissemination in Escherichia coli Isolated from Wild Animals: Bacterial Clones and Plasmid Complicity *Microbiology Research*, 12(1), 123

892. Belas, Adriana, Correia, Joana, Marques, Cátia, Luís Telo da Gama, Pomba, Constança (2021). ESBL/AmpC-Producing Enterobacteriaceae Fecal Colonization in Dogs after Elective Surgery *Microbiology Research*, 12(4), 907

893. Sato, Toyotaka, Yokota, Shin-ichi, Tachibana, Tooru, Tamai, Satoshi, Maetani, Shigeki, Tamura, Yutaka, Horiuchi, Motohiro (2021). Isolation of Human Lineage, Fluoroquinolone-Resistant and Extended-β-Lactamase-Producing Escherichia coli Isolates from Companion Animals in Japan *Antibiotics*, 10(12), 1463

894. NandaKafle, Gitanjali, Taylor Huegen, Potgieter, Sarah C, Steenkamp, Emma, Venter, Stephanus N, Brözel, Volker S (2021). Niche Preference of Escherichia coli in a Peri-Urban Pond Ecosystem *Life*, 11(10), 1020

895. Biel Garcias, Aguirre, Laia, Seminati, Chiara, Reyes, Nerea, Allepuz, Alberto, Obón, Elena, Molina-Lopez, Rafael A, Darwich, Laila (2021). Extended-Spectrum β-Lactam Resistant Klebsiella pneumoniae and Escherichia coli in Wild European Hedgehogs (Erinaceus europeus) Living in Populated Areas *Animals*, 11(10), 2837

896. Carvalho, Isabel, Carvalho, José António, Martínez-Álvarez, Sandra, Sadi, Madjid, Capita, Rosa, Alonso-Calleja, Carlos, Rabbi, Fazle, Maria de Lurdes Nunes Enes Dapkevicius, Igrejas, Gilberto, Torres, Carmen, Poeta, Patrícia (2021). Characterization of ESBL-Producing Escherichia coli and Klebsiella pneumoniae Isolated from Clinical Samples in a Northern Portuguese Hospital: Predominance of CTX-M-15 and High Genetic Diversity *Microorganisms*, 9(9), 1914

897. Salgado-Caxito, Marília, Moreno-Switt, Andrea I, Paes, Antonio Carlos, Shiva, Carlos, Munita, Jose M, Rivas, Lina, Benavides, Julio A (2021). Higher Prevalence of Extended-Spectrum Cephalosporin-Resistant Enterobacterales in Dogs Attended for Enteric Viruses in Brazil Before and After Treatment with Cephalosporins *Antibiotics*, 10(2), 122

898. Schmitt, Kira, Kuster, Stefan P, Zurfluh, Katrin, Jud, Rahel S, Sykes, Jane E, Stephan, Roger, Willi, Barbara (2021). Transmission Chains of Extended-Spectrum Beta-Lactamase-Producing Enterobacteriaceae at the Companion Animal Veterinary Clinic–Household

Interface *Antibiotics*, 10(2), 171

899. Se Ra Shin, Noh, Seong Mi, Woo, Kyung Jung, Shin, Sook, Park, Young Kyung, Dong Chan Moon, Suk-Kyung Lim, Yong Ho Park, Kun Taek Park (2021). Characterization of Extended-Spectrum β-Lactamase-Producing and AmpC β-Lactamase-Producing Enterobacterales Isolated from Companion Animals in Korea *Antibiotics*, 10(3), 249

900. Algammal, Abdelazeem M, Hashem, Hany R, Alfifi, Khyreyah J, Hetta, Helal F, Sheraba, Norhan S, Ramadan Hazem, El-Tarabili, Reham M (2021). atpD gene sequencing, multidrug resistance traits, virulence-determinants, and antimicrobial resistance genes of emerging XDR and MDR-Proteus mirabilis *Scientific Reports (Nature Publisher Group)*, 11(1), #Pages#

901. Khalifa, Hazim O, Oreiby, Atef F, Okanda Takashi, Kato Yasuyuki, Matsumoto, Tetsuya (2021). High β-lactam resistance in Gram-negative bacteria associated with kennel cough and cat flu in Egypt *Scientific Reports (Nature Publisher Group)*, 11(1), #Pages#

902. Hasan Ejaz, Younas, Sonia, Abosalif, Khalid O A, Kashaf Junaid, Alzahrani, Badr, Alsrhani, Abdullah, Abualgasim Elgaili Abdalla, Muhammad Ikram Ullah, Qamar, Muhammad Usman, Hamam, Sanaa S M (2021). Molecular analysis of blaSHV, blaTEM, and blaCTX-M in extended-spectrum β-lactamase producing Enterobacteriaceae recovered from fecal specimens of animals *PLoS One*, 16(1), #Pages#

903. Toombs-Ruane, Leah J, Benschop, Jackie, French, Nigel P, Biggs, Patrick J, Midwinter, Anne C, Marshall, Jonathan C, Chan, Maggie, Drinković, Dragana, Fayaz, Ahmed, Baker, Michael G, Douwes, Jeroen, Roberts, Mick G, Burgess, Sara A (2020). Carriage of Extended-Spectrum-Beta-Lactamase- and AmpC Beta-Lactamase-Producing Escherichia coli Strains from Humans and Pets in the Same Households. *Applied and environmental microbiology*, 86(24), #Pages#

904. Phoo, May Thet Paing, Ngasaman, Ruttayaporn, Indoung, Saowakon, Naknaen, Ampapan, Chukamnerd, Arnon, Pomwised, Rattanaruji (2020). Occurrence Of Ndm-5 And Antibiotic Resistance Genes Among Escherichia Coli And Klebsiella Pneumoniae In Companion Animals In Thailand *Southeast Asian Journal Of Tropical Medicine And Public Health*, 51(3), 391

905. Poudel, Anil, Kang, Yuan, Mandal, Rabindra K, Kalalah, Anwar, Butaye, Patrick, Hathcock, Terri, Kelly, Patrick, Walz, Paul, Macklin, Kenneth, Cattley, Russell, Price, Stuart, Adekanmbi, Folasade, Zhang, Lixin, Kitchens, Steven, Kaltenboeck, Bernhard, Wang, Chengming (2020). Comparison of microbiota, antimicrobial resistance genes and mobile genetic elements in flies and the feces of sympatric animals *FEMS Microbiology Ecology*, 96(4), #Pages#

906. Brilhante, Michael, Menezes, Juliana, Belas, Adriana, Feudi, Claudia, Schwarz, Stefan, Pomba, Constança, Perreten, Vincent (2020). OXA-181-Producing Extraintestinal Pathogenic Escherichia coli Sequence Type 410 Isolated from a Dog in Portugal.

Antimicrobial agents and chemotherapy, 64(4), #Pages#

907. Cole, Stephen D, Peak, Laura, Tyson, Gregory H, Reimschuessel, Renate, Ceric, Olgica, Rankin, Shelley C (2020). New Delhi Metallo-β-Lactamase-5-Producing Escherichia coli in Companion Animals, United States. *Emerging infectious diseases*, 26(2), 381

908. Dogan, Belgin, Zhang, Shiying, Kalla, Sarah E, Dogan, Esra I, Guo, Cindy, Ang, Chelston R, Simpson, Kenneth W (2020). Molecular and Phenotypic Characterization of Escherichia coli Associated with Granulomatous Colitis of Boxer Dogs *Antibiotics*, 9(9), 540

909. Moser, Sarah, Seth-Smith, Helena, Egli, Adrian, Kittl, Sonja, Overesch, Gudrun (2020). Campylobacter jejuni from Canine and Bovine Cases of Campylobacteriosis Express High Antimicrobial Resistance Rates against (Fluoro)quinolones and Tetracyclines *Pathogens*, 9(9), 691

910. Abreu-Salinas, Fátima, Díaz-Jiménez, Dafne, García-Meniño, Isidro, Lumbreras, Pilar, López-Beceiro, Ana María, Fidalgo, Luis Eusebio, María Rosario Rodicio, Mora, Azucena, Fernández, Javier (2020). High Prevalence and Diversity of Cephalosporin-Resistant Enterobacteriaceae Including Extraintestinal Pathogenic E. coli CC648 Lineage in Rural and Urban Dogs in Northwest Spain *Antibiotics*, 9(8), 468

911. Igwaran, Aboi, Okoh, Anthony Ifeanyi (2020). Occurrence, Virulence and Antimicrobial Resistance-Associated Markers in Campylobacter Species Isolated from Retail Fresh Milk and Water Samples in Two District Municipalities in the Eastern Cape Province, South Africa *Antibiotics*, 9(7), 426

912. Loncaric, Igor, Misic, Dusan, Szostak, Michael P, Künzel, Frank, Schäfer-Somi, Sabine, Spergser, Joachim (2020). Broad-Spectrum Cephalosporin-Resistant and/or Fluoroquinolone-Resistant Enterobacterales Associated with Canine and Feline Urogenital Infections *Antibiotics*, 9(7), 387

913. Adator, Emelia H, Narvaez-Bravo, Claudia, Zaheer, Rahat, Cook, Shaun R, Tymensen, Lisa, Hannon, Sherry J, Booker, Calvin W, Church, Deirdre, Read, Ron R, McAllister, Tim A (2020). A One Health Comparative Assessment of Antimicrobial Resistance in Generic and Extended-Spectrum Cephalosporin-Resistant Escherichia coli from Beef Production, Sewage and Clinical Settings *Microorganisms*, 8(6), 885

914. Vidal, Anna, Aguirre, Laia, Seminati, Chiara, Tello, Montse, Redondo, Noelia, Martín, Marga, Darwich, Laila (2020). Antimicrobial Resistance Profiles and Characterization of Escherichia coli Strains from Cases of Neonatal Diarrhea in Spanish Pig Farms *Veterinary Sciences*, 7(2), 48

915. Ball, Takiyah, Monte, Daniel, Aidara-Kane, Awa, Matheu, Jorge, Ru, Hongyu, Thakur, Siddhartha, Ejobi, Francis, Fedorka-Cray, Paula (2020). International lineages of Salmonella enterica serovars isolated from chicken farms, Wakiso District, Uganda *PLoS One*, 15(1),

#Pages#

916. Jun Sung Hong, Song, Wonkeun, Park, Hee-Myung, Jae-Young, Oh, Jong-Chan, Chae, Jae-Ik Han, Jeong, Seok Hoon (2019). First Detection of New Delhi Metallo-β-Lactamase-5-Producing Escherichia coli from Companion Animals in Korea *Microbial Drug Resistance*, 25(3), 344

917. Harada, Kazuki, Shimizu, Takae, Ozaki, Hiroichi, Kimura, Yui, Miyamoto, Tadashi, Tsuyuki, Yuzo (2019). Characterization of Antimicrobial Resistance in Serratia spp. and Citrobacter spp. Isolates from Companion Animals in Japan: Nosocomial Dissemination of Extended-Spectrum Cephalosporin-Resistant Citrobacter freundii *Microorganisms*, 7(3), 64

918. Baloch, Zulqarnain, Lv, Luchao, Lingxian Yi, Miao Wan, Aslam, Bilal, Yang, Jun, Liu, Jian-Hua (2019). Emergence of Almost Identical F36:A-:B32 Plasmids Carrying blaNDM-5 and qepA in Escherichia coli from Both Pakistan and Canada *Infection and Drug Resistance*, 12(#issue#), 3981

919. Salinas Liseth, Cárdenas Paúl, Johnson, Timothy J, Vasco Karla, Graham, Jay, Trueba Gabriel (2019). Diverse Commensal Escherichia coli Clones and Plasmids Disseminate Antimicrobial Resistance Genes in Domestic Animals and Children in a Semirural Community in Ecuador *mSphere*, 4(3), #Pages#

920. Ghazanfar Abbas, Khan, Iahtasham, Mohsin, Mashkoor, Sajjad-ur Rahman, Younas, Tayyaba, Shahzad, Ali (2019). High rates of CTX-M group-1 extended-spectrum β-lactamases producing Escherichia coli from pets and their owners in Faisalabad, Pakistan *Infection and Drug Resistance*, 12(#issue#), 571

921. Amer, Mohamed M, Mekky, Hoda M, Amer, Aziza M, Fedawy, Hanaa S (2018). Antimicrobial resistance genes in pathogenic Escherichia coli isolated from diseased broiler chickens in Egypt and their relationship with the phenotypic resistance characteristics *Veterinary World*, 11(8), 1082

922. Wolny-Koładka, Katarzyna, Lenart-Boroń, Anna (2018). Antimicrobial resistance and the presence of extended-spectrum beta-lactamase genes in Escherichia coli isolated from the environment of horse riding centers *Environmental Science and Pollution Research*, 25(22), 21789

923. Mansour Amin, Mehrandokht Sirous, Javaherizadeh, Hazhir, Motamedifar, Mohammad, Saki, Morteza, Veisi, Hojat, Ebrahimi, Saeedeh, Seyed-Mohammadi, Sakineh, Hashemzadeh, Mohammad (2018). Antibiotic resistance pattern and molecular characterization of extended-spectrum β-lactamase producing enteroaggregative Escherichia coli isolates in children from southwest Iran *Infection and Drug Resistance*, 11(#issue#), 1097

924. Lecuyer, Tessa Emily (2018). Clonality and Antimicrobial Resistance in Canine Uropathogenic Escherichia coli *#journal#*, #volume#(#issue#), 125

925. Liu, Baoguang, Wu, Hua, Zhai, Yajun, He, Zhipei, Sun, Huarun, Cai, Tian, He, Dandan, Liu, Jianhua, Wang, Shanmei, Pan, Yushan, Yuan, Li, Hu, Gongzheng (2018). Prevalence and molecular characterization of oqxAB in clinical Escherichia coli isolates from companion animals and humans in Henan Province, China. *Antimicrobial resistance and infection control*, 7(#issue#), 18

926. Touzain, Fabrice, Laetitia Le Devendec, de Boisséson, Claire, Baron, Sandrine, Jouy, Eric, Perrin-Guyomard, Agnès, Blanchard, Yannick, Kempf, Isabelle (2018). Characterization of plasmids harboring blaCTX-M and blaCMY genes in E. coli from French broilers *PLoS One*, 13(1), #Pages#

927. Todorovic, Dalibor, Velhner, Maja, Grego, Edita, Vidanovic, Dejan, Milanov, Dubravka, Krnjaic, Dejan, Kehrenberg, Corinna (2018). Molecular Characterization of Multidrug-Resistant Escherichia coli Isolates from Bovine Clinical Mastitis and Pigs in the Vojvodina Province, Serbia *Microbial Drug Resistance*, 24(1), 95

928. Baede, Valérie O, Broens, Els M, Spaninks, Mirlin P, Timmerman, Arjen J, Graveland, Haitske, Wagenaar, Jaap A, Duim, Birgitta, Hordijk, Joost (2017). Raw pet food as a risk factor for shedding of extended-spectrum beta-lactamase-producing Enterobacteriaceae in household cats *PLoS One*, 12(11), #Pages#

929. N Mohammad Sharif, Sreedevi, B, Chaitanya, R K, Sreenivasulu, D (2017). Beta-lactamase antimicrobial resistance in Klebsiella and Enterobacter species isolated from healthy and diarrheic dogs in Andhra Pradesh, India *Veterinary World*, 10(8), 950

930. Vieira Cunha, Marcos Paulo, Andre Becker Saidenberg, Moreno, Andrea Micke, Antonio José Piantino Ferreira, Mônica Aparecida Midolli Vieira, Tânia Aparecida Tardelli Gomes, Knöbl, Terezinha (2017). Pandemic extra-intestinal pathogenic Escherichia coli (ExPEC) clonal group O6-B2-ST73 as a cause of avian colibacillosis in Brazil *PLoS One*, 12(6), #Pages#

931. Timofte, Dorina, Maciuca, Iuliana Elena, Williams, Nicola J, Wattret, Andrew, Schmidt, Vanessa (2016). Veterinary Hospital Dissemination of CTX-M-15 Extended-Spectrum Beta-Lactamase-Producing Escherichia coli ST410 in the United Kingdom *Microbial Drug Resistance*, 22(7), 609

932. Ljungquist, Oskar, MD, Ljungquist, Ditte, DVM, Myren◦s, Mattias, MSc, Rydén, Cecilia, MD, PhD, Finn, Maria, MLS, Bengtsson, Björn, DVM, PhD (2016). Evidence of household transfer of ESBL-/pAmpCproducing Enterobacteriaceae between humans and dogs - a pilot study *Infection Ecology & Epidemiology*, 6(1), 1

933. Liu, Xiaoqiang, Thungrat, Kamoltip, Boothe, Dawn M (2015). Multilocus Sequence Typing and Virulence Profiles in Uropathogenic Escherichia coli Isolated from Cats in the United States *PLoS One*, 10(11), #Pages#

934. Rzewuska, Magdalena, Stefańska, Ilona, Kizerwetter-Świda, Magdalena, Chrobak-Chmiel, Dorota, Szczygielska, Paulina, Leśniak, Monika, Binek, Marian (2015). Characterization of extended-spectrum-β-lactamases produced by Escherichia coli strains isolated from dogs in Poland *Polish Journal of Microbiology*, 64(3), 5

935. Schaufler, Katharina, Bethe, Astrid, Lübke-Becker, Antina, Ewers, Christa, Kohn, Barbara, Wieler, Lothar H, Günther, Sebastian (2015). Putative connection between zoonotic multiresistant extended-spectrum beta-lactamase (ESBL)-producing Escherichia coli in dog feces from a veterinary campus and clinical isolates from dogs *Infection Ecology & Epidemiology*, 5(#issue#), #Pages#

936. Donati, Valentina, Feltrin, Fabiola, Hendriksen, Rene S, Christina Aaby Svendsen, Cordaro, Gessica, García-Fernández, Aurora, Lorenzetti, Serena, Lorenzetti, Raniero, Battisti, Antonio, Franco, Alessia (2014). Extended-Spectrum-Beta-Lactamases, AmpC Beta-Lactamases and Plasmid Mediated Quinolone Resistance in Klebsiella spp. from Companion Animals in Italy *PLoS One*, 9(3), #Pages#

937. Agostinho, Juliana M A, de Souza, Andressa, Schocken-Iturrino, Ruben P, Beraldo, Livia G, Borges, Clarissa A, Ávila, Fernando A, Marin, Jose M (2014). Escherichia coli Strains Isolated from the Uteri Horn, Mouth, and Rectum of Bitches Suffering from Pyometra: Virulence Factors, Antimicrobial Susceptibilities, and Clonal Relationships among Strains *International Journal of Microbiology*, 2014(#issue#), #Pages#

938. Wu, Guanghui, Day, Michaela J, Mafura, Muriel T, Nunez-Garcia, Javier, Fenner, Jackie J, Sharma, Meenaxi, Alieda van Essen-Zandbergen, Rodríguez, Irene, Dierikx, Cindy, Kadlec, Kristina, Schink, Anne-Kathrin, Wain, John, Reiner Helmuth, Guerra, Beatriz, Schwarz, Stefan, Threlfall, John, Woodward, Martin J, Woodford, Neil, Coldham, Nick, Mevius, Dik (2013). Comparative Analysis of ESBL-Positive Escherichia coli Isolates from Animals and Humans from the UK, The Netherlands and Germany *PLoS One*, 8(9), #Pages#

939. Ghanbarpour, Reza (2013). Detection of [beta]-lactamase and urovirulence genes in Escherichia coli serogroups isolated from urinary tract infection in cats *Comparative Clinical Pathology*, 22(4), 591

940. Ghanbarpour, Reza, Akhtardanesh, Baharak (2012). Genotype and antibiotic resistance profile of Escherichia coli strains involved in canine pyometra *Comparative Clinical Pathology*, 21(5), 737

941. Umaer Naseer, Olsson-Liljequist, Barbro E, Woodford, Neil, Dhanji, Hiran, Cantón, Rafael, Sundsfjord, Arnfinn, Lindstedt, Bjørn-Arne (2012). Multi-Locus Variable Number of Tandem Repeat Analysis for Rapid and Accurate Typing of Virulent Multidrug Resistant Escherichia coli Clones *PLoS One*, 7(7), #Pages#

942. Leonard, Brian Callan (2012). Role of Antimicrobial Peptides in Companion Animal Health. *#journal#*, #volume#(#issue#), 1

943. Brolund, Alma, Sundqvist, Martin, Kahlmeter, Gunnar, Grape, Malin (2010). Molecular Characterisation of Trimethoprim Resistance in Escherichia coli and Klebsiella pneumoniae during a Two Year Intervention on Trimethoprim Use *PLoS One*, 5(2), #Pages#

944. Lim, Suk-Kyung, Lee, Hee-Soo, Nam, Hyang-Mi, Jung, Suk-Chan, Bae, You-chan (2009). CTX-M-Type [beta]-Lactamase in Escherichia coli Isolated from Sick Animals in Korea *Microbial Drug Resistance*, 15(2), 139

945. Depaula, Cleber Jacob Silva, Marin, Jose Moacir (2008). Isolation of extraintestinal pathogenic Escherichia coli from diarrheic dogs and their antimicrobial resistance profile *Brazilian Journal of Microbiology*, 39(3), 498

946. Costa, Daniela, Poeta, Patricia, Senz, Yolanda, Vinu, Laura, Coelho, Ana Cludia, Matos, Manuela, Rojo-Bezares, Beatriz, Rodrigues, Jorge, Torres, Carmen (2008). Mechanisms of Antibiotic Resistance in Escherichia coli Isolates Recovered from Wild Animals *Microbial Drug Resistance*, 14(1), 71

947. Jouini, Ahlem, Vinué, Laura, Slama, Karim Ben, Sáenz, Yolanda, Klibi, Naouel, Hammami, Salah, Boudabous, Abdellatif, Torres, Carmen (2007). Characterization of CTX-M and SHV extended-spectrum [beta]-lactamases and associated resistance genes in Escherichia coli strains of food samples in Tunisia *The Journal of Antimicrobial Chemotherapy*, 60(5), 1137

948. Cocchi, Stefano, Grasselli, Elena, Gutacker, Michaela, Benagli, Cinzia, Convert, Maruska, Piffaretti, Jean-Claude (2007). Distribution and characterization of integrons in Escherichia coli strains of animal and human origin *Pathogens and Disease*, 50(1), 126

949. Sidjabat, Hanna E, Townsend, Kirsty M, Hanson, Nancy D, Bell, Jan M, Stokes, H W, Gobius, Kari S, Moss, Susan M, Trott, Darren J (2006). Identification of blaCMY-7 and associated plasmid-mediated resistance genes in multidrug-resistant Escherichia coli isolated from dogs at a veterinary teaching hospital in Australia *The Journal of Antimicrobial Chemotherapy*, 57(5), 840

950. Kadlec, Kristina, Kehrenberg, Corinna, Schwarz, Stefan (2005). Molecular basis of resistance to trimethoprim, chloramphenicol and sulphonamides in Bordetella bronchiseptica *The Journal of Antimicrobial Chemotherapy*, 56(3), 485

951. Briñas, Laura, Moreno, Miguel Angel, Tirushet Teshager, Zarazaga, Myriam, Sáenz, Yolanda, Porrero, Concepción, Dominguez, Lucas, Torres, Carmen (2003). β-Lactamase Characterization in Escherichia coli Isolates with Diminished Susceptibility or Resistance to Extended-Spectrum Cephalosporins Recovered from Sick Animals in Spain *Microbial Drug Resistance*, 9(2), 201

952. Salgado-Caxito, Marília, Benavides, Julio A, Munita, Jose M, Rivas, Lina, García, Patricia, Listoni, Fernando J.P., Moreno-Switt, Andrea I, Paes, Antonio C (2021). Risk factors associated with faecal carriage of extended-spectrum cephalosporin-resistant

Escherichia coli among dogs in Southeast Brazil *Preventive veterinary medicine*, 190(#issue#), #Pages#

953. Kidsley, Amanda K, O'Dea, Mark, Saputra, Sugiyono, Jordan, David, Johnson, James R, Gordon, David M, Turni, Conny, Djordjevic, Steven P, Abraham, Sam, Trott, Darren J (2020). Genomic analysis of phylogenetic group B2 extraintestinal pathogenic E. coli causing infections in dogs in Australia *Veterinary microbiology*, 248(#issue#), #Pages#

954. Vikram, Amit, Schmidt, John W (2018). Functional blaKPC−2 Sequences Are Present in U.S. Beef Cattle Feces Regardless of Antibiotic Use *Foodborne pathogens & disease*, 15(7), 444

955. Fang, Liang-Xing, Deng, Hui, Sun, Jian, Li, Liang, Chen, Mu-Ya, Qiu-E. Yang, Huang, Ting, Xiao-Ping Liao, Li, Xue, Ya-Hong, Liu (2015). Dissemination of the chromosomally encoded CMY-2 cephalosporinase gene in Escherichia coli isolated from animals *International journal of antimicrobial agents*, 46(2), 209

956. Sun, Y, Zeng, Z, Chen, S, Ma, J, He, L, Liu, Y, Deng, Y, Lei, T, Zhao, J, Liu, JH (2010). High prevalence of bla(CTX-M) extended-spectrum beta-lactamase genes in Escherichia coli isolates from pets and emergence of CTX-M-64 in China *Clinical Microbiology And Infection*, 16(9), 1475

957. Srisanga, S, Angkititrakul, S, Sringam, P, Ho, PTL, Vo, ATT, Chuanchuen, R (2017). Phenotypic and genotypic antimicrobial resistance and virulence genes of Salmonella enterica isolated from pet dogs and cats *Journal Of Veterinary Science*, 18(3), 273

958. Yousfi, M, Touati, A, Muggeo, A, Mira, B, Asma, B, Brasme, L, Guillard, T, de Champs, C (2018). Clonal dissemination of OXA-48-producing Enterobacter cloacae isolates from companion animals in Algeria *Journal Of Global Antimicrobial Resistance*, 12(#issue#), 187

959. Melo, LC, Haenni, M, Saras, E, Cerdeira, L, Moura, Q, Boulouis, HJ, Madec, JY, Lincopan, N (2019). Genomic characterisation of a multidrug-resistant TEM-52b extended-spectrum beta-lactamase-positive Escherichia coli ST219 isolated from a cat in France *Journal Of Global Antimicrobial Resistance*, 18(#issue#), 223

960. Askari, A, Ghanbarpour, R, Akhtardanesh, B, Aflatoonian, MR, Sharifi, H, Jajarmi, M, Molaei, R (2020). Detection of zoonotic diarrheagenic pathotypes of Escherichia coli in healthy household dogs *Iranian Journal Of Microbiology*, 12(6), 522

961. Harada, K, Shimizu, T, Mukai, Y, Kuwajima, K, Sato, T, Usui, M, Tamura, Y, Kimura, Y, Miyamoto, T, Tsuyuki, Y, Ohki, A, Kataoka, Y (2016). Phenotypic and Molecular Characterization of Antimicrobial Resistance in Klebsiella spp. Isolates from Companion Animals in Japan: Clonal Dissemination of Multidrug-Resistant Extended-Spectrum beta-Lactamase-Producing Klebsiella pneumoniae *Frontiers In Microbiology*, 7(#issue#), #Pages#

962. Silva, MM, Sellera, FP, Fernandes, MR, Moura, Q, Garino, F, Azevedo, SS, Lincopan, N (2018). Genomic features of a highly virulent, ceftiofur-resistant, CTX-M-8-producing Escherichia coli ST224 causing fatal infection in a domestic cat *Journal Of Global Antimicrobial Resistance*, 15(#issue#), 252

963. Gandolfi-Decristophoris, P, Petrini, O, Ruggeri-Bernardi, N, Schelling, E (2013). Extended-spectrum beta-lactamase-producing Enterobacteriaceae in healthy companion animals living in nursing homes and in the community *American Journal Of Infection Control*, 41(9), 831

964. Zhang, SQ, Abbas, M, Rehman, MU, Wang, MS, Jia, RY, Chen, S, Liu, MF, Zhu, DK, Zhao, XX, Gao, Q, Tian, B, Cheng, AC (2021). Updates on the global dissemination of colistin-resistant Escherichia coli: An emerging threat to public health *Science Of The Total Environment*, 799(#issue#), #Pages#

965. Matusaiki, CD, Ferreira, RG, Otutumi, LK, dos Santos, IC, Ramos, FAP, Mezalira, TS, Jacomassi, E, Barbosa, LN, Goncalves, DD, Soares, AA (2021). Antibiotic resistance profile of gram-negative bacteria isolated from dog nasal swab samples, and antibacterial and antioxidant activities of aqueous extracts of Alpinia purpurta (Vieill.) K. Schum (Zingiberaceae) *Semina-Ciencias Agrarias*, 42(1), 179

966. Toombs-Ruane, LJ, Marshall, JC, Benschop, J, Drinkovi, D, Midwinter, AC, Biggs, PJ, Grange, Z, Baker, MG, Douwes, J, Roberts, MG, French, NP, Burgess, SA (2023). Extended-spectrum fi-lactamase- and AmpC fi-lactamase-producing Enterobacterales associated with urinary tract infections in the New Zealand community: a case-control study *International Journal Of Infectious Diseases*, 128(#issue#), 325

967. Chantharothaiphaichit, T, Phongaran, D, Angkittitrakul, S, Aunpromma, S, Chuanchuen, R (2022). Clinically healthy household dogs and cats as carriers of multidrug-resistant Salmonella enterica with variable R plasmids *Journal Of Medical Microbiology*, 71(2), #Pages#

968. Hidalgo, L, Gutierrez, B, Ovejero, CM, Carrilero, L, Matrat, S, Saba, CKS, Santos-Lopez, A, Thomas-Lopez, D, Hoefer, A, Suarez, M, Santurde, G, Martin-Espada, C, Gonzalez-Zorn, B (2013). Klebsiella pneumoniae Sequence Type 11 from Companion Animals Bearing ArmA Methyltransferase, DHA-1 beta-Lactamase, and QnrB4 *Antimicrobial Agents And Chemotherapy*, 57(9), 4532

969. Baede, VO, Wagenaar, JA, Broens, EM, Duim, B, Dohmen, W, Nijsse, R, Timmerman, AJ, Hordijk, J (2015). Longitudinal Study of Extended-Spectrum-beta-Lactamase- and AmpC-Producing Enterobacteriaceae in Household Dogs *Antimicrobial Agents And Chemotherapy*, 59(6), 3117

970. Johard, S, Borjesson, S, Trowald-Wigh, G, Fernstrom, LL, Nicol, C, Bergstrom, A (2015). Extended Spectrum Beta-Lactamase/AmpC-Producing E Coli in Dogs Treated with Antimicrobials in Surgical Wards *International Journal Of Applied Research In Veterinary*

Medicine, 13(1), 80

971. Kalayci-Yuksek, F, Gumus, D, Macunluoglu, AC, Eroglu, E, Camadan, D, Ang-Kucuker, M (2022). Mobile resistance determinants, plasmid replicon types and phylogeny among Escherichia coli strains isolated from cats and dogs *Journal Of The Hellenic Veterinary Medical Society*, 73(4), 5039

972. Franiek, N, Orth, D, Grif, K, Ewers, C, Wieler, LH, Thalhammer, JG, Wurzner, R (2012). ESBL-producing E. coli and EHEC in dogs and cats in the Tyrol as possible source of human infection *Berliner Und Munchener Tierarztliche Wochenschrift*, 125(11-12), 469

973. Flament-Simon, SC, de Toro, M, Garcia, V, Blanco, JE, Blanco, M, Alonso, MP, Goicoa, A, Diaz-Gonzalez, J, Nicolas-Chanoine, MH, Blanco, J (2020). Molecular Characteristics of Extraintestinal Pathogenic E. coli (ExPEC), Uropathogenic E. coli (UPEC), and Multidrug Resistant E. coli Isolated from Healthy Dogs in Spain. Whole Genome Sequencing of Canine ST372 Isolates and Comparison with Human Isolates Causing Extraintestinal Infections *Microorganisms*, 8(11), #Pages#

974. de Paula, CJS, Marin, JM (2008). Occurrence of non-O157 Shiga toxin-producing Escherichia coli in dogs with diarrhea *CIENCIA RURAL*, 38(6), 1682

975. Rodrigues, CG, Melo, RT, Fonseca, BB, Martins, PA, Ferreira, FA, Araujo, MBJ, Rossi, DA (2015). Occurrence and characterization of Campylobacter spp. isolates in dogs, cats and children *Pesquisa Veterinaria Brasileira*, 35(4), 365

976. Coura, FM, Diniz, AN, Oliveira, CA, Lage, AP, Lobato, FCF, Heinemann, MB, Silva, ROS (2018). Detection of virulence genes and the phylogenetic groups of Escherichia coli isolated from dogs in Brazil *Ciencia Rural*, 48(2), #Pages#

977. Aslantas, O, Yilmaz, ES (2017). Prevalence and molecular characterization of extended-spectrum beta-lactamase (ESBL) and plasmidic AmpC beta-lactamase (pAmpC) producing Escherichia coli in dogs *Journal Of Veterinary Medical Science*, 79(6), 1024

978. Liu, XQ, Liu, HX, Li, YQ, Hao, CJ (2016). High Prevalence of beta-lactamase and Plasmid-Mediated Quinolone Resistance Genes in Extended-Spectrum Cephalosporin-Resistant Escherichia coli from Dogs in Shaanxi, China *Frontiers In Microbiology*, 7(#issue#), #Pages#

979. Harada, K, Sasaki, A, Shimizu, T (2016). Effects of oral orbifloxacin on fecal coliforms in healthy cats: a pilot study *Journal Of Veterinary Medical Science*, 78(1), 83

980. Acke, E, McGill, K, Quinn, T, Jones, BR, Fanning, S, Whyte, P (2009). Antimicrobial Resistance Profiles and Mechanisms of Resistance in Campylobacter jejuni Isolates from Pets *Foodborne Pathogens And Disease*, 6(6), 705

981. Nebbia, P, Tramuta, C, Odore, R, Nucera, D, Zanatta, R, Robino, P (2014). Genetic and phenotypic characterisation of Escherichia coli producing cefotaximase-type extended-spectrum beta-lactamases: first evidence of the ST131 clone in cats with urinary infections in Italy *Journal Of Feline Medicine And Surgery*, 16(12), 966

982. van den Bunt, G, Fluit, AC, Spaninks, MP, Timmerman, AJ, Geurts, Y, Kant, A, Scharringa, J, Mevius, D, Wagenaar, JA, Bonten, MJM, van Pelt, W, Hordijk, J (2020). Faecal carriage, risk factors, acquisition and persistence of ESBL-producing Enterobacteriaceae in dogs and cats and co-carriage with humans belonging to the same household *Journal Of Antimicrobial Chemotherapy*, 75(2), 342

983. Gentilini, F, Turba, ME, Pasquali, F, Mion, D, Romagnoli, N, Zambon, E, Terni, D, Peirano, G, Pitout, JDD, Parisi, A, Sambri, V, Zanoni, RG (2018). Hospitalized Pets as a Source of Carbapenem-Resistance *Frontiers In Microbiology*, 9(#issue#), #Pages#

984. Marques, C, Betas, A, Aboim, C, Cavaco-Silva, P, Trigueiro, G, Gama, LT, Pomba, C (2019). Evidence of Sharing of Klebsiella pneumoniae Strains between Healthy Companion Animals and Cohabiting Humans *Journal Of Clinical Microbiology*, 57(6), #Pages#

985. DebRoy, C, Sidhu, MS, Sarker, U, Jayarao, BM, Stell, AL, Bell, NP, Johnson, TJ (2010). Complete sequence of pEC14_114, a highly conserved IncFIB/FIIA plasmid associated with uropathogenic Escherichia coli cystitis strains *Plasmid*, 63(1), 53

986. Nigg, A, Brilhante, M, Dazio, V, Clement, M, Collaud, A, Brawand, SG, Willi, B, Endimiani, A, Schuller, S, Perreten, V (2019). Shedding of OXA-181 carbapenemase-producing Escherichia coli from companion animals after hospitalisation in Switzerland: an outbreak in 2018 *Eurosurveillance*, 24(39), 13

987. Kidsley, AK, White, RT, Beatson, SA, Saputra, S, Schembri, MA, Gordon, D, Johnson, JR, O'Dea, M, Mollinger, JL, Abraham, S, Trott, DJ (2020). Companion Animals Are Spillover Hosts of the Multidrug-Resistant Human ExtraintestinalEscherichia coliPandemic Clones ST131 and ST1193 *Frontiers In Microbiology*, 11(#issue#), #Pages#

988. Mahmoud, AE, El-Maghraby, MM, Eltarabili, RM, Soliman, ES (2022). Epidemiological investigations on microbial infection and crystals causing feline lower urinary tract disease in tomcats in Ismailia, Egypt *Open Veterinary Journal*, 12(2), 290

989. Rubin, JE, Pitout, JDD (2014). Extended-spectrum beta-lactamase, carbapenemase and AmpC producing Enterobacteriaceae in companion animals *Veterinary Microbiology*, 170(1-2), 10

990. Pehlivanoglu, F, Sababoglu, E (2021). Characterisation Of Ampc / Esbl Genes In Some Pathogen Gram-Negatives Isolated From Clinical Cases Of Livestock And Companion Animals *Acta Veterinaria-Beograd*, 71(4), 435

Level 1, Form level_1_screening, What was the study type? -> Other (e.g., Lab studies for validation of AST)

991. Shah, P M, Modak, S, Fox, C L, Babu, S C, Sampath, L, Clauss, R H, Stahl, W M (1987). PTFE graft treated with silver norfloxacin (AgNF): drug retention and resistance to bacterial challenge. *The Journal of surgical research*, 42(3), 298

992. RE Wooley, MS Jones (1983). Action of EDTA-Tris and antimicrobial agent combinations on selected pathogenic bacteria. *Veterinary microbiology*, 8(3), #Pages#

993. Speakman, A J, Binns, S H, Osborn, A M, Corkill, J E, Kariuki, S, Saunders, J R, Dawson, S, Gaskell, R M, Hart, C A (1997). Characterization of antibiotic resistance plasmids from Bordetella bronchiseptica. *The Journal of antimicrobial chemotherapy*, 40(6), 811

994. C Goldstein, MD Lee, S Sanchez, C Hudson, B Phillips, B Register, M Grady, C Liebert, AO Summers, DG White, JJ Maurer (2001). Incidence of class 1 and 2 integrases in clinical and commensal bacteria from livestock, companion animals, and exotics. *Antimicrobial agents and chemotherapy*, 45(3), #Pages#

995. Harmoinen, Jaana, Mentula, Silja, Heikkila, Matti, van der Rest, Michel, Rajala-Schultz, Paivi J, Donskey, Curtis J, Frias, Rafael, Koski, Pertti, Wickstrand, Nina, Jousimies-Somer, Hannele, Westermarck, Elias, Lindevall, Kai (2004). Orally administered targeted recombinant Beta-lactamase prevents ampicillin-induced selective pressure on the gut microbiota: a novel approach to reducing antimicrobial resistance. *Antimicrobial agents and chemotherapy*, 48(1), 75

996. Moore, D F, Harwood, V J, Ferguson, D M, Lukasik, J, Hannah, P, Getrich, M, Brownell, M (2005). Evaluation of antibiotic resistance analysis and ribotyping for identification of faecal pollution sources in an urban watershed. *Journal of applied microbiology*, 99(3), 618

997. Wetzstein, H-G (2005). Comparative mutant prevention concentrations of pradofloxacin and other veterinary fluoroquinolones indicate differing potentials in preventing selection of resistance. *Antimicrobial agents and chemotherapy*, 49(10), 4166

998. Mentula, Silja, Virtanen, Terhi, Kanervo-Nordstrom, Arja, Harmoinen, Jaana, Westermarck, Elias, Rautio, Merja, Huovinen, Pentti, Kononen, Eija (2006). Relatedness of Escherichia coli strains with different susceptibility patterns isolated from beagle dogs during ampicillin treatment. *International journal of antimicrobial agents*, 27(1), 46

999. DM Boothe, A Boeckh, RB Simpson, K Dubose (2006). Comparison of pharmacodynamic and pharmacokinetic indices of efficacy for 5 fluoroquinolones toward pathogens of dogs and cats. *Journal of veterinary internal medicine*, 20(6), #Pages#

1000. Lefebvre, S L, Reid-Smith, R, Boerlin, P, Weese, J S (2008). Evaluation of the risks of shedding Salmonellae and other potential pathogens by therapy dogs fed raw diets in Ontario

and Alberta. *Zoonoses and public health*, 55(8-10), 470

1001. JR Johnson, S Miller, B Johnston, C Clabots, C Debroy (2009). Sharing of Escherichia coli sequence type ST131 and other multidrug-resistant and Urovirulent E. coli strains among dogs and cats within a household. *Journal of clinical microbiology*, 47(11), #Pages#

1002. Lindsey, Rebecca L, Frye, Jonathan G, Thitaram, Sutawee N, Meinersmann, Richard J, Fedorka-Cray, Paula J, Englen, Mark D (2011). Characterization of multidrug-resistant Escherichia coli by antimicrobial resistance profiles, plasmid replicon typing, and pulsed-field gel electrophoresis. *Microbial drug resistance (Larchmont, N.Y.)*, 17(2), 157

1003. X Liu, DM Boothe, Y Jin, K Thungrat (2013). In vitro potency and efficacy favor later generation fluoroquinolones for treatment of canine and feline Escherichia coli uropathogens in the United States. *World journal of microbiology & biotechnology*, 29(2), #Pages#

1004. Sato, Toyotaka, Yokota, Shin-ichi, Okubo, Torahiko, Ishihara, Kanako, Ueno, Hiroshi, Muramatsu, Yasukazu, Fujii, Nobuhiro, Tamura, Yutaka (2013). Contribution of the AcrAB-TolC efflux pump to high-level fluoroquinolone resistance in Escherichia coli isolated from dogs and humans. *The Journal of veterinary medical science*, 75(4), 407

1005. Martins, Liliana Raquel Leite, Pina, Susana Maria Rocha, Simoes, Romeo Luis Rocha, de Matos, Augusto Jose Ferreira, Rodrigues, Pedro, da Costa, Paulo Martins Rodrigues (2013). Common phenotypic and genotypic antimicrobial resistance patterns found in a case study of multiresistant E. coli from cohabitant pets, humans, and household surfaces. *Journal of environmental health*, 75(6), 74

1006. Oliveira, Manuela, Dias, Filipa Rocha, Pomba, Constanca (2014). Biofilm and fluoroquinolone resistance of canine Escherichia coli uropathogenic isolates. *BMC research notes*, 7(#issue#), 499

1007. Guardabassi, Luca, Hedberg, Sandra, Jessen, Lisbeth Rem, Damborg, Peter (2015). Optimization and evaluation of Flexicult R Vet for detection, identification and antimicrobial susceptibility testing of bacterial uropathogens in small animal veterinary practice. *Acta veterinaria Scandinavica*, 57(#issue#), 72

1008. SI Green, JT Kaelber, L Ma, BW Trautner, RF Ramig, AW Maresso (2017). Bacteriophages from ExPEC Reservoirs Kill Pandemic Multidrug-Resistant Strains of Clonal Group ST131 in Animal Models of Bacteremia. *Scientific reports*, 7(), #Pages#

1009. Badger, Skye, Abraham, Sam, Saputra, Sugiyono, Trott, Darren J, Turnidge, John, Mitchell, Tahlia, Caraguel, Charles G B, Jordan, David (2018). Relative performance of antimicrobial susceptibility assays on clinical Escherichia coli isolates from animals. *Veterinary microbiology*, 214(#issue#), 56

1010. VV Ebani, S Nardoni, F Bertelloni, L Pistelli, F Mancianti (2018). Antimicrobial Activity of Five Essential Oils against Bacteria and Fungi Responsible for Urinary Tract Infections. *Molecules (Basel, Switzerland)*, 23(7), #Pages#

1011. C Bacci, A Vismarra, S Dander, E Barilli, P Superchi (2019). Occurrence and Antimicrobial Profile of Bacterial Pathogens in Former Foodstuff Meat Products Used for Pet Diets. *Journal of food protection*, 82(2), #Pages#

1012. SY Lu, T Graça, JJ Avillan, Z Zhao, DR Call (2019). Microcin PDI Inhibits Antibiotic-Resistant Strains of Escherichia coli and <i>Shigella</i> through a Mechanism of Membrane Disruption and Protection by Homotrimer Self-Immunity. *Applied and environmental microbiology*, 85(11), #Pages#

1013. TE Fiamengo, EE Runcan, C Premanandan, B Blawut, MA Coutinho da Silva (2020). Evaluation of Biofilm Production by Escherichia coli Isolated From Clinical Cases of Canine Pyometra. *Topics in companion animal medicine*, 39(), #Pages#

1014. C Vercelli, M Della Ricca, M Re, G Gambino, G Re (2021). Antibiotic Stewardship for Canine and Feline Acute Urinary Tract Infection: An Observational Study in a Small Animal Hospital in Northwest Italy. *Antibiotics (Basel, Switzerland)*, 10(5), #Pages#

1015. Cugmas, Blaz, Avbersek, Miha, Rosa, Teja, Godec, Leonida, Struc, Eva, Golob, Majda, Zdovc, Irena (2021). How Accurate Are Veterinary Clinicians Employing Flexicult Vet for Identification and Antimicrobial Susceptibility Testing of Urinary Bacteria?. *Antibiotics (Basel, Switzerland)*, 10(10), #Pages#

1016. M Rutkowski, L Krzemińska-Fiedorowicz, G Khachatryan, J Kabacińska, M Tischner, A Suder, K Kulik, A Lenart-Boroń (2022). Antibacterial Properties of Biodegradable Silver Nanoparticle Foils Based on Various Strains of Pathogenic Bacteria Isolated from the Oral Cavity of Cats, Dogs and Horses. *Materials (Basel, Switzerland)*, 15(3), #Pages#

1017. V Bampidis, G Azimonti, ML Bastos, H Christensen, B Dusemund, M Fašmon Durjava, M Kouba, M López-Alonso, S López Puente, F Marcon, B Mayo, A Pechová, M Petkova, F Ramos, Y Sanz, RE Villa, R Woutersen, G Bories, B Glandorf, K Svensson, M Anguita, R Brozzi, J Galobart, L Gregoretti, ML Innocenti, E Pettenati, F Pizzo, J Tarrés-Call, MV Vettori, G López-Gálvez (2022). Safety and efficacy of a feed additive consisting of zearalenone hydrolase produced by <i>Escherichia coli</i> DSM 32731 for all terrestrial animal species (Biomin GmbH). *EFSA journal. European Food Safety Authority*, 20(2), #Pages#

1018. McDonnell, Shannon, Gutierrez, Montserrat, Leonard, Finola C, O'Brien, Tony, Kearney, Pat, Swan, Catherine, Madigan, Gillian, Bracken, Elaine, McLernon, Joanne, Griffin, Margaret, O'Sullivan, Ciaran M, Egan, John, Prendergast, Deirdre M (2022). A survey of food-borne and antimicrobial resistance-harbouring bacteria in meat by-products from knackeries and associated equipment and kennels. *Irish veterinary journal*, 75(1), 9

1019. SS Nielsen, DJ Bicout, P Calistri, E Canali, JA Drewe, B Garin-Bastuji, JL Gonzales Rojas, C Gortázar, M Herskin, V Michel, MÁ Miranda Chueca, B Padalino, P Pasquali, HC Roberts, H Spoolder, K Ståhl, A Velarde, A Viltrop, C Winckler, F Baldinelli, A Broglia, L

Kohnle, J Alvarez (2022). Assessment of listing and categorisation of animal diseases within the framework of the Animal Health Law (Regulation (EU) No 2016/429): antimicrobial-resistant <i>Escherichia coli</i> in dogs and cats, horses, swine, poultry, cattle, sheep and goats. *EFSA journal. European Food Safety Authority*, 20(5), #Pages#

1020. D Nobrega, G Peirano, JDD Pitout (2022). Escherichia coli sequence type 73 bloodstream infections in a centralized Canadian region and their association with companion animals: an ecological study. *Infection*, 50(6), #Pages#

1021. Menard, Julie, Goggs, Robert, Mitchell, Patrick, Yang, Yufan, Robbins, Sarah, Franklin-Guild, Rebecca J, Thachil, Anil J, Altier, Craig, Anderson, Renee, Putzel, Gregory G, McQueary, Holly, Goodman, Laura B (2022). Effect of antimicrobial administration on fecal microbiota of critically ill dogs: dynamics of antimicrobial resistance over time. *Animal microbiome*, 4(1), 36

1022. L Harrison, GH Tyson, E Strain, RL Lindsey, N Strockbine, O Ceric, GZ Fortenberry, B Harris, S Shaw, G Tillman, S Zhao, U Dessai (2022). Use of Large-Scale Genomics to Identify the Role of Animals and Foods as Potential Sources of Extraintestinal Pathogenic Escherichia coli That Cause Human Illness. *Foods (Basel, Switzerland)*, 11(13), #Pages#

1023. Azzariti, Stefano, Bond, Ross, Loeffler, Anette, Zendri, Flavia, Timofte, Dorina, Chang, Yu-Mei, Pelligand, Ludovic (2022). Investigation of In Vitro Susceptibility and Resistance Mechanisms in Skin Pathogens: Perspectives for Fluoroquinolone Therapy in Canine Pyoderma. *Antibiotics (Basel, Switzerland)*, 11(9), #Pages#

1024. CS Machado, MG Seeger, KS Moreira, TAL Burgo, BA Iglesias, FSF Vogel, JF Cargnelutti (2022). In vitro porphyrin-based photodynamic therapy against mono and polyculture of multidrug-resistant bacteria isolated from integumentary infections in animals. *Photodiagnosis and photodynamic therapy*, 40(), #Pages#

1025. Pinthanon, Artitaya, Nithitarnwat, Chayanan, Pintapin, Chadaporn, Siripanee, Chonradee, Yindee, Jitrapa, Am-In, Nutthee, Kesdangsakonwut, Sawang, Surachetpong, Sirilak, Prapasarakul, Nuvee (2023). Rapid identification of canine uropathogens by matrix-assisted laser desorption/ionization-time-of-flight mass spectrometry and the clinical factors that correlated bacterial species and antimicrobial resistance. *Veterinary research communications*, #volume#(#issue#), #Pages#

1026. Bampidis, V., Azimonti, G., Lourdes Bastos, M. de, Christensen, H., Dusemund, B., Durjava, M. F., Kouba, M., López-Alonso, M., Puente, S. L., Marcon, F., Mayo, B., Pechová, A., Petkova, M., Ramos, F., Sanz, Y., Villa, R. E., Woutersen, R., Bories, G., Glandorf, B., Svensson, K., Anguita, M., Brozzi, R., Galobart, J., Gregoretti, L., Innocenti, M. L., Pettenati, E., Pizzo, F., Tarrés-Call, J., Vettori, M. V., López-Gálvez, G. (2022). Safety and efficacy of a feed additive consisting of zearalenone hydrolase produced by Escherichia coli DSM 32731 for all terrestrial animal species (Biomin GmbH). *EFSA Journal*, 20(2), #Pages#

1027. Chen ChaoXi, Liao XiaoPing, Zhu HengQian, Liu YaHong (2010). Biofilm phenotype and drug resistance pattern analyses in 106 Escherichia coli strains from pets. *Chinese Veterinary Science / Zhongguo Shouyi Kexue*, 40(9), 886

1028. Lu ShaoYeh, Graça, T., Avillan, J. J., Zhao Zhe, Call, D. R. (2019). Microcin PDI inhibits antibiotic-resistant strains of Escherichia coli and Shigella through a mechanism of membrane disruption and protection by homotrimer self-immunity. *Applied and Environmental Microbiology*, 85(11), #Pages#

1029. Fiamengo, T. E., Runcan, E. E., Premanandan, C., Blawut, B., Silva, M. A. C. da (2020). Evaluation of biofilm production by Escherichia coli isolated from clinical cases of canine pyometra. *Topics in Companion Animal Medicine*, 39(#issue#), #Pages#

1030. Oyagbemi, T. O., Ogunleye, A. O., Lawal, T. O., Oyagbemi, A. A. (2013). Antimicrobial activity of Parquetina nigrescens on some multidrug resistant pathogens isolated from poultry and cases of otitis media in dogs from Nigeria. *African Journal of Biotechnology*, 12(13), 1559

1031. Goldstein, C., Lee, M. D., Sanchez, S., Hudson, C., Phillips, B., Register, B., Grady, M., Liebert, C., Summers, A. O., White, D. G., Maurer, J. J. (2001). Incidence of class 1 and 2 integrases in clinical and commensal bacteria from livestock, companion animals, and exotics. *Antimicrobial Agents and Chemotherapy*, 45(3), 723

1032. Butucel, Eugenia, Igori Balta, Iulia, Adelina Bundurus, Popescu, Cosmin Alin, Iancu, Tiberiu, Venig, Adelina, Pet, Ioan, Ducu Stef, McCleery, David, Stef, Lavinia, Corcionivoschi, Nicolae (2023). Natural Antimicrobials Promote the Anti-Oxidative Inhibition of COX-2 Mediated Inflammatory Response in Primary Oral Cells Infected with Staphylococcus aureus, Streptococcus pyogenes and Enterococcus faecalis *Antioxidants*, 12(5), 1017

1033. Deliephan, Aiswariya, Dhakal, Janak, Subramanyam, Bhadriraju, Aldrich, Charles G (2023). Use of Organic Acid Mixtures Containing 2-Hydroxy-4-(Methylthio) Butanoic Acid (HMTBa) to Mitigate Salmonella enterica, Shiga Toxin-Producing Escherichia coli (STEC) and Aspergillus flavus in Pet Food Kibbles *Animals*, 13(5), 877

1034. Ortega-Lozano, Ariadna Jazmín, Hernández-Cruz, Estefani Yaquelin, Gómez-Sierra, Tania, Pedraza-Chaverri, José (2023). Antimicrobial Activity of Spices Popularly Used in Mexico against Urinary Tract Infections *Antibiotics*, 12(2), 325

1035. Nobrega, Diego, Peirano, Gisele, Pitout, Johann D. D. (2022). Escherichia coli sequence type 73 bloodstream infections in a centralized Canadian region and their association with companion animals: an ecological study *Infection*, 50(6), 1579

1036. Foster-Nyarko, Ebenezer, Pallen, Mark J (2022). The microbial ecology of Escherichia coli in the vertebrate gut *FEMS Microbiology Reviews*, 46(3), #Pages#

1037. EFSA Panel on Animal Health and Welfare (AHAW), Nielsen, Søren Saxmose, Bicout, Dominique Joseph, Calistri, Paolo, Canali, Elisabetta, Drewe, Julian Ashley, Garin-Bastuji, Bruno, Gonzales Rojas, José Luis, Gortázar, Christian, Herskin, Mette, Michel, Virginie, Miranda Chueca, Miguel Ángel, Padalino, Barbara, Pasquali, Paolo, Roberts, Helen Clare, Spoolder, Hans, Ståhl, Karl, Velarde, Antonio, Viltrop, Arvo, Winckler, Christoph, Baldinelli, Francesca, Broglia, Alessandro, Kohnle, Lisa, Alvarez, Julio (2022). Assessment of listing and categorisation of animal diseases within the framework of the Animal Health Law (Regulation (EU) No 2016/429): antimicrobial-resistant Escherichia coli in dogs and cats, horses, swine, poultry, cattle, sheep and goats. *EFSA journal. European Food Safety Authority*, 20(5), 1

1038. de Miranda, Juliana Oliveira, Oliveira, Samily Aquino Sá, de Sales, Sinara Laís Ramalho, Rosa, Danillo Sales, Coelho, Jéssica Xavier, da Costa, Mateus Matiuzzi, Gois, Glayciane Costa, Rodrigues, Rafael Torres de Souza (2022). Prevalence, Biofilm Formation, and Antimicrobial Resistance of Escherichia coli, Staphylococcus aureus, and Salmonella Isolates from Goat Meat Marketed in Petrolina, Brazil *Food Protection Trends*, 42(2), 139

1039. Pezzanite, Lynn M, Chow, Lyndah, Strumpf, Alyssa, Johnson, Valerie, Dow, Steven W (2022). Immune Activated Cellular Therapy for Drug Resistant Infections: Rationale, Mechanisms, and Implications for Veterinary Medicine *Veterinary Sciences*, 9(11), 610

1040. Karen C. Carroll, Carey-Ann D. Burnham, Lars F. Westblade (2021). From canines to humans: Clinical importance of Staphylococcus pseudintermedius *PLoS Pathogens*, 17(12), #Pages#

1041. Dazio, Valentina, Nigg, Aurélien, Schmidt, Janne S, Brilhante, Michael, Campos-Madueno, Edgar I, Mauri, Nico, Kuster, Stefan P, Brawand, Stefanie Gobeli, Willi, Barbara, Endimiani, Andrea, Perreten, Vincent, Schuller, Simone (2021). Duration of carriage of multidrug-resistant bacteria in dogs and cats in veterinary care and co-carriage with their owners. *One health (Amsterdam, Netherlands)*, 13(#issue#), 100322

1042. Hayakawa Ito de Sousa, Alessandra Tammy, Dos Santos Costa, Marco Túlio, Makino, Herica, Cândido, Stéfhano Luis, de Godoy Menezes, Isabela, Lincopan, Nilton, Nakazato, Luciano, Dutra, Valéria (2021). Multidrug-resistant mcr-1 gene-positive Klebsiella pneumoniae ST307 causing urinary tract infection in a cat. *Brazilian journal of microbiology : [publication of the Brazilian Society for Microbiology]*, 52(2), 1043

1043. da Cunha, Eva Sofia Gonçalves (2021). Periodontal Disease in Dogs: An Experimental Approach Towards Prevention Using Antimicrobial Peptides *#journal#*, #volume#(#issue#), 168

1044. Rödel, Alice (2021). Biocide Tolerance and Antibiotic Cross-Resistance in Human Pathogenic Bacteria *#journal#*, #volume#(#issue#), 144

1045. Gómez-Beltrán, David A, Schaeffer, David J, Ferguson, Duncan C, Monsalve, Laura K, Villar, David (2021). Antimicrobial Prescribing Practices in Dogs and Cats by Colombian

Veterinarians in the City of Medellin *Veterinary Sciences*, 8(5), 73

1046. Hubbuch, Alina, Schmitt, Kira, Lehner, Claudia, Hartnack, Sonja, Schuller, Simone, Schüpbach-Regula, Gertraud, Mevissen, Meike, Ruth, Peter, Müntener, Cedric, Naegeli, Hanspeter, Willi, Barbara (2020). Antimicrobial prescriptions in cats in Switzerland before and after the introduction of an online antimicrobial stewardship tool *BMC Veterinary Research*, 16(#issue#), 1

1047. Delis, Georgios A, Siarkou, Victoria I, Vingopoulou, Elpida I, Koutsoviti-Papadopoulou, Maria, Batzias, Georgios C (2018). Pharmacodynamic interactions of amikacin with selected β -lactams and fluoroquinolones against canine Escherichia coli isolates *Research in Veterinary Science*, 117(#issue#), 187

1048. Palaniyandi, Sasikumar Arunachalam, Damodharan, Karthiyaini, Suh, Joo-won, Yang, Seung Hwan (2017). In Vitro Characterization of Lactobacillus plantarum Strains with Inhibitory Activity on Enteropathogens for Use as Potential Animal Probiotics *Indian Journal of Microbiology*, 57(2), 201

1049. Liu, Xiaoqiang, Boothe, Dawn M, Jin, Yaping, Thungrat, Kamoltip (2013). In vitro potency and efficacy favor later generation fluoroquinolones for treatment of canine and feline Escherichia coli uropathogens in the United States *World Journal of Microbiology and Biotechnology*, 29(2), 347

1050. Grønvold, Anne-Mette R, L'Abée-Lund, Trine M, Sørum, Henning, Skancke, Ellen, Yannarell, Anthony C, Mackie, Roderick I (2009). Changes in fecal microbiota of healthy dogs administered amoxicillin *FEMS Microbiology Ecology*, 71(2), 313

1051. Wernicki, A, Krzyzanowski, J, Puchalski, A, Kowalczyk, D (2000). Characteristics of some properties of E. coli isolates from canine pyometra *Medycyna Weterynaryjna*, 56(1), 49

1052. Shimizu, T, Harada, K, Manabe, S, Tsukamoto, T, Ito, N, Hikasa, Y (2017). Assessment of urinary pharmacokinetics and pharmacodynamics of orbifloxacin in healthy dogs with ex vivo modelling *Journal Of Medical Microbiology*, 66(5), 616

Level 2, Form level_2_screening, Is the full text available? -> No

1053. Vogel, P L, Komtebedde, J, Hirsh, D C, Kass, P H (1999). Wound contamination and antimicrobial susceptibility of bacteria cultured during total ear canal ablation and lateral bulla osteotomy in dogs. *Journal of the American Veterinary Medical Association*, 214(11), 1641

1054. J Wipler, Z Čermáková, T Hanzálek, H Horáková, H Žemličková (2017). [Sharing bacterial microbiota between owners and their pets (dogs, cats)]. *Klinicka mikrobiologie a infekcni lekarstvi*, 23(2), #Pages#

1055. Okpara, E. O., Olufemi, F. O., Ojo, E. O., Nwanta, J. A. (2018). Prevalence and antimicrobial resistance of Streptococcus species isolated from the vagina of bitches in Lagos metropolis, Nigeria. *Tropical Veterinarian*, 36(2), 134

1056. Ossiprandi, M. C., Cattabiani, F., Risio, L. de, Bottarelli, E. (2006). Antibiotic resistance in bacteria isolated from urinary tracts of cats and dogs with neurologic impairment. *Igiene Moderna*, 125(1), 27

1057. Poeta, P., Rodrigues, J. (2008). Detection of antibiotic resistance in clinical bacterial strains from pets. *Arquivo Brasileiro de Medicina Veterinária e Zootecnia*, 60(2), 506

1058. Tsuyuki, Y., Takahashi, T. (2017). Isolated bacteria and their antimicrobial susceptibility through urine culture obtained from companion animals in Japan. *Journal of the Japanese Association for Infectious Diseases*, 91(3), 392

1059. Salgado, Brianna, KuKanich, Katherine, Lubbers, Brian (2019). PSI-25 development of local Kansas E. coli UTI antibiograms to improve antimicrobial stewardship in companion animal medicine *Journal of Animal Science, suppl. Supplement 2*, 97(#issue#), 241

1060. Trolldenier, H (2001). Quantitative sensitivity of frequently isolated veterinary pathogens as ascertained by multicentric studies in comparison to chemotherapeutics used in veterinary medicine - Part I: E. coli-bovine and porcine strains *Praktische Tierarzt*, 82(1), 52

1061. [Anonymous] (2005). Risk factors affecting the occurrence of antimicrobial resistance or intermediate susceptibility in fecal E-coli isolated from healthy dogs and cats from private veterinary clinics in Southern Ontario. *Journal Of Veterinary Internal Medicine*, 19(3), 402

Level 2, Form level_2_screening, Does the full text describe dogs and/or cats? -> No

1062. Ebrahim, M, Gravel, D, Thabet, C, Abdesselam, K, Paramalingam, S, Hyson, C (2016). Antimicrobial use and antimicrobial resistance trends in Canada: 2014. *Canada communicable disease report = Releve des maladies transmissibles au Canada*, 42(11), 227

1063. Ge, Beilei, Domesle, Kelly J, Gaines, Stuart A, Lam, Claudia, Bodeis Jones, Sonya M, Yang, Qianru, Ayers, Sherry L, McDermott, Patrick F (2020). Prevalence and Antimicrobial Susceptibility of Indicator Organisms Escherichia coli and Enterococcus spp. Isolated from U.S. Animal Food, 2005–2011 *Microorganisms*, 8(7), 1048

1064. Zara, ES, Vital, PG (2022). Phylogroup typing and carbapenem resistance of Escherichia coli from agricultural samples in Metro Manila, Philippines *Journal Of Environmental Science And Health Part B-Pesticides Food Contaminants And Agricultural Wastes*, 57(8), 644

Level 2, Form level_2_screening, Does the full text describe the pathogen E. coli? -> No

1065. Prüller, Sandra, Rensch, Ulrike, Meemken, Diana, Kaspar, Heike, Kopp, Peter A, Klein, Günter, Kehrenberg, Corinna (2015). Antimicrobial Susceptibility of Bordetella bronchiseptica Isolates from Swine and Companion Animals and Detection of Resistance Genes *PLoS One*, 10(8), #Pages#

Level 2, Form level_2_screening, Does the full text describe antimicrobial resistance? -> No

1066. Lyskova, P, Vydrzalova, M, Mazurova, J (2007). Identification and antimicrobial susceptibility of bacteria and yeasts isolated from healthy dogs and dogs with otitis externa. *Journal of veterinary medicine. A, Physiology, pathology, clinical medicine*, 54(10), 559

1067. KEL Worsley-Tonks, SD Gehrt, EA Miller, RS Singer, JB Bender, JD Forester, SC McKenzie, DA Travis, TJ Johnson, ME Craft (2021). Comparison of Antimicrobial-Resistant Escherichia coli Isolates from Urban Raccoons and Domestic Dogs. *Applied and environmental microbiology*, 87(15), #Pages#

1068. DC Moon, JH Choi, N Boby, HY Kang, SJ Kim, HJ Song, HS Park, MC Gil, SS Yoon, SK Lim (2022). Bacterial Prevalence in Skin, Urine, Diarrheal Stool, and Respiratory Samples from Dogs. *Microorganisms*, 10(8), #Pages#

1069. Moon DongChan, Choi JiHyun, Boby NaiLa, Kim SuJeong, Song HyunJu, Park HoSung, Gil MinChan, Yoon SoonSeek, Lim SukKyung (2022). Prevalence of bacterial species in skin, urine, diarrheal stool, and respiratory samples in cats. *Pathogens*, 11(3), #Pages#

1070. Gibson, J S, Morton, J M, Cobbold, R N, Filippich, L J, Trott, D J (2011). Risk factors for dogs becoming rectal carriers of multidrug-resistant Escherichia coli during hospitalization *Epidemiology and Infection, suppl. Zoonoses*, 139(10), 1511

1071. Camargo, VE, Arias, MVB, Perugini, MRE (2020). Clinical and microbiological characteristics of dogs in sepsis in an academic veterinary hospital in the north of Parana *Pesquisa Veterinaria Brasileira*, 40(11), 903

Level 2, Form level_2_screening, Was the study a primary research article? -> No. It's a narrative review

1072. Schwarz, S., Alešík, E., Grobbel, M., Lübke-Becker, A., Wallmann, J., Werckenthin, C., Wieler, L. H. (2007). The BfT-GermVet monitoring program - aims and basics. *Berliner und Münchener Tierärztliche Wochenschrift*, 120(9/10), 357

Level 2, Form level_2_screening, Was the study a primary research article? -> No. It's a scoping review

1073. CM Marinho, T Santos, A Gonçalves, P Poeta, G Igrejas (2016). A Decade-Long Commitment to Antimicrobial Resistance Surveillance in Portugal. *Frontiers in microbiology*, 7(), #Pages#

Level 2, Form level_2_screening, What was the study type? -> Descriptive studies (including case report or case series)

1074. Pumphrey, Stephanie A, Wayne, Annie S (2022). Patterns of bacterial culture and antimicrobial susceptibility test results for dogs with retrobulbar abscesses: 133 cases (2002-2019). *Journal of the American Veterinary Medical Association*, 260(8), 1

1075. Ramos, C. G., Costa, M. T. (2006). Patterns of resistance to β-lactams and β-lactamase inhibitors in uropathogenic Escherichia coli strains isolated from animals in Portugal. *African Journal of Biotechnology*, 5(6), 523

Level 2, Form level_2_screening, What was the study type? -> To understand the molecular basis of resistance

1076. N Drazenovich, GV Ling, J Foley (2004). Molecular investigation of Escherichia coli strains associated with apparently persistent urinary tract infection in dogs. *Journal of veterinary internal medicine*, 18(3), #Pages#

1077. Takagi, Hirotaka, Yamane, Kunikazu, Matsui, Mari, Suzuki, Satowa, Ito, Kenichiro (2020). Pathotypes and Drug Susceptibility of Escherichia coli Isolated from Companion Dogs in Japan. *Japanese Journal of infectious diseases*, 73(3), 253

1078. Singleton, David A, Pongchaikul, Pisut, Smith, Shirley, Bengtsson, Rebecca J, Baker, Kate, Timofte, Dorina, Steen, Stephen, Jones, Matthew, Roberts, Larry, Sanchez-Vizcaino, Fernando, Dawson, Susan, Noble, P-J M, Radford, Alan D, Pinchbeck, Gina L, Williams, Nicola J (2021). Temporal, Spatial, and Genomic Analyses of Enterobacteriaceae Clinical Antimicrobial Resistance in Companion Animals Reveals Phenotypes and Genotypes of One Health Concern. *Frontiers in microbiology*, 12(#issue#), 700698

1079. Mekky, R., Moustafa, A. M., Abo-Sakaya, R., Abu-Zaid, K. (2021). Molecular characterization of salmonella species and E.coli isolated from dogs and cats. *Benha Veterinary Medical Journal*, 40(2), 12

1080. Tramuta, C., Nucera, D., Robino, P., Salvarani, S., Nebbia, P. (2012). Antibiotic resistance profiles in relation to virulence factors and phylogenetic groups of uropathogenic Escherichia coli isolated from dogs and cats. *Veterinary science. LXIV Annual Meeting of the Italian Society for Veterinary Sciences, Asti, 2010*, #volume#(#issue#), 51

1081. Hata Akihisa, Fujitani Noboru, Ono Fumiko, Yoshikawa Yasuhiro (2022). Surveillance of antimicrobial-resistant Escherichia coli in Sheltered dogs in the Kanto Region of Japan *Scientific Reports (Nature Publisher Group)*, 12(1), #Pages#

1082. Börjesson, Stefan, Gunnarsson, Lotta, Landén, Annica, Grönlund, Ulrika (2020). Low occurrence of extended-spectrum cephalosporinase producing Enterobacteriaceae and no detection of methicillin-resistant coagulase-positive staphylococci in healthy dogs in Sweden *Acta Veterinaria Scandinavica*, 62(#issue#), 1

1083. Boehmer, Tim, Vogler, Amy J, Thomas, Astrid, Sauer, Sabine, Hergenroether, Markus, Straubinger, Reinhard K, Birdsell, Dawn, Keim, Paul, × Jason W Sahl, Williamson, Charles H D, Julia M Riehm × (2018). Phenotypic characterization and whole genome analysis of extended-spectrum beta-lactamase-producing bacteria isolated from dogs in Germany *PLoS One*, 13(10), #Pages#

1084. Rusdi, Bertha, Laird, Tanya, Abraham, Rebecca, Ash, Amanda, Robertson, Ian D, Mukerji, Shewli, Coombs, Geoffrey W, Abraham, Sam, Mark A O'Dea (2018). Carriage of critically important antimicrobial resistant bacteria and zoonotic parasites amongst camp dogs in remote Western Australian indigenous communities *Scientific Reports (Nature Publisher Group)*, 8(#issue#), 1

1085. Silley, Peter, Stephan, Bernd, Greife, Heinrich A, Pridmore, Andrew (2007). Comparative activity of pradofloxacin against anaerobic bacteria isolated from dogs and cats *The Journal of Antimicrobial Chemotherapy*, 60(5), 999

1086. Wedley, AL, Dawson, S, Maddox, TW, Coyne, KP, Pinchbeck, GL, Clegg, P, Nuttall, T, Kirchner, M, Williams, NJ (2017). Carriage of antimicrobial resistant Escherichia coli in dogs: Prevalence, associated risk factors and molecular characteristics *Veterinary Microbiology*, 199(#issue#), 23

Level 2, Form level_2_screening, What was the study type? -> Other (e.g., validation of AST methodologies)

1087. Harada, Kazuki, Okada, Erika, Shimizu, Takae, Kataoka, Yasushi, Sawada, Takuo, Takahashi, Toshio (2012). Antimicrobial resistance, virulence profiles, and phylogenetic groups of fecal Escherichia coli isolates: a comparative analysis between dogs and their owners in Japan. *Comparative immunology, microbiology and infectious diseases*, 35(2), 139

1088. E Frey, M Jacob (2020). Development of a method for creating antibiograms for use in companion animal private practices. *Journal of the American Veterinary Medical Association*, 257(9), #Pages#

1089. Blondeau, Joseph M, Fitch, Shantelle D (2021). In Vitro Killing of Canine Urinary Tract Infection Pathogens by Ampicillin, Cephalexin, Marbofloxacin, Pradofloxacin, and Trimethoprim/Sulfamethoxazole. *Microorganisms*, 9(11), #Pages#

1090. Mader, Rodolphe, Bourely, Clemence, Amat, Jean-Philippe, Broens, Els M, Busani, Luca, Callens, Benedicte, Crespo-Robledo, Paloma, Damborg, Peter, Filippitzi, Maria-Eleni, Fitzgerald, William, Gronthal, Thomas, Haenni, Marisa, Heuvelink, Annet, van Hout, Jobke, Kaspar, Heike, Munoz Madero, Cristina, Norstrom, Madelaine, Pedersen, Karl, Pokludova, Lucie, Dal Pozzo, Fabiana, Slowey, Rosemarie, Urdahl, Anne Margrete, Vatopoulos, Alkiviadis, Zafeiridis, Christos, Madec, Jean-Yves (2022). Defining the scope of the European Antimicrobial Resistance Surveillance network in Veterinary medicine (EARS-Vet): a bottom-up and One Health approach. *The Journal of antimicrobial chemotherapy*, 77(3), 816

1091. Matos, Alice, Cunha, Eva, Baptista, Lara, Tavares, Luís, Oliveira, Manuela (2023). ESBL-Positive Enterobacteriaceae from Dogs of Santiago and Boa Vista Islands, Cape Verde: A Public Health Concern *Antibiotics*, 12(3), 447

1092. Mader, Rodolphe, Bourély, Clémence, Amat, Jean-Philippe, Broens, Els M, Busani, Luca, Callens, Bénédicte, Crespo, Paloma, Damborg, Peter, Maria-Eleni Filippitzi, Fitzgerald, William, Grönthal, Thomas, Haenni, Marisa, Heuvelink, Annet, Jobke Van Hout, Kaspar, Heike, Munoz, Cristina, Norström, Madelaine, Pedersen, Karl, Pokludova, Lucie, Fabiana Dal Pozzo, Slowey, Rosemarie, Urdahl, Anne Margrete, Vatopoulos, Alkiviadis, Zafeiridis, Christos, Madec, Jean-Yves (2021). Defining the scope of the European Antimicrobial Resistance Surveillance network in Veterinary medicine (EARS-Vet): a bottom-up and One Health approach *bioRxiv*, #volume#(#issue#), #Pages#

Summary of articles that were deemed eligible to be included and that data were charted

Level	Data charting	# Articles included	# Total
3	Full text	1093 to 1200	108

1093. Normand, E H, Gibson, N R, Taylor, D J, Carmichael, S, Reid, S W (2000). Trends of antimicrobial resistance in bacterial isolates from a small animal referral hospital. The Veterinary record, 146(6), 151

1094. Y Sáenz, M Zarazaga, L Briñas, M Lantero, F Ruiz-Larrea, C Torres (2001). Antibiotic resistance in Escherichia coli isolates obtained from animals, foods and humans in Spain. International journal of antimicrobial agents, 18(4), #Pages#

1095. Cooke, Cara L, Singer, Randall S, Jang, Spencer S, Hirsh, Dwight C (2002). Enrofloxacin resistance in Escherichia coli isolated from dogs with urinary tract infections. Journal of the American Veterinary Medical Association, 220(2), 190

1096. Cohn, Leah A, Gary, Anthony T, Fales, William H, Madsen, Richard W (2003). Trends in fluoroquinolone resistance of bacteria isolated from canine urinary tracts. Journal of veterinary diagnostic investigation : official publication of the American Association of Veterinary Laboratory Diagnosticians, Inc, 15(4), 338

1097. De Graef, E M, Decostere, A, Devriese, L A, Haesebrouck, F (2004). Antibiotic resistance among fecal indicator bacteria from healthy individually owned and kennel dogs. Microbial drug resistance (Larchmont, N.Y.), 10(1), 65

1098. Sannes, Mark R, Kuskowski, Michael A, Johnson, James R (2004). Antimicrobial resistance of Escherichia coli strains isolated from urine of women with cystitis or pyelonephritis and feces of dogs and healthy humans. Journal of the American Veterinary Medical Association, 225(3), 368

1099. Rantala, M, Lahti, E, Kuhalampil, J, Pesonen, S, Jarvinen, A K, Saijonmaa-Koulumies, Honkanen-Buzalski, T (2004). Antimicrobial resistance in Staphylococcus spp., Escherichia coli and Enterococcus spp. in dogs given antibiotics for chronic dermatological disorders, compared with non-treated control dogs. Acta veterinaria Scandinavica, 45(1-2), 37

1100. Sayah, Raida S, Kaneene, John B, Johnson, Yvette, Miller, RoseAnn (2005). Patterns of antimicrobial resistance observed in Escherichia coli isolates obtained from domestic- and wild-animal fecal samples, human septage, and surface water. Applied and environmental microbiology, 71(3), 1394

1101. Moyaert, H, De Graef, E M, Haesebrouck, F, Decostere, A (2006). Acquired antimicrobial resistance in the intestinal microbiota of diverse cat populations. Research in veterinary science, 81(1), 1

1102. Authier, Simon, Paquette, Dominique, Labrecque, Olivia, Messier, Serge (2006). Comparison of susceptibility to antimicrobials of bacterial isolates from companion animals in a veterinary diagnostic laboratory in Canada between 2 time points 10 years apart. The Canadian veterinary journal = La revue veterinaire canadienne, 47(8), 774

1103. Ogeer-Gyles, Jennifer, Mathews, Karol A, Sears, William, Prescott, John F, Weese, J S, Boerlin, Patrick (2006). Development of antimicrobial drug resistance in rectal Escherichia coli isolates from dogs hospitalized in an intensive care unit. Journal of the American Veterinary Medical Association, 229(5), 694

1104. Pedersen, Karl, Pedersen, Kristina, Jensen, Helene, Finster, Kai, Jensen, Vibeke F, Heuer, Ole E (2007). Occurrence of antimicrobial resistance in bacteria from diagnostic samples from dogs. The Journal of antimicrobial chemotherapy, 60(4), 775

1105. Bubenik, Loretta J, Hosgood, Giselle L, Waldron, Don R, Snow, Lynne A (2007). Frequency of urinary tract infection in catheterized dogs and comparison of bacterial culture and susceptibility testing results for catheterized and noncatheterized dogs with urinary tract infections. Journal of the American Veterinary Medical Association, 231(6), 893

1106. Grobbel, Mirjam, Lubke-Becker, Antina, Alesik, Eva, Schwarz, Stefan, Wallmann, Jurgen, Werckenthin, Christiane, Wieler, Lothar H (2007). Antimicrobial susceptibility of Escherichia coli from swine, horses, dogs and cats as determined in the BfT-GermVet monitoring program 2004-2006. Berliner und Munchener tierarztliche Wochenschrift, 120(9-10), 391

1107. Gottlieb, S, Wigney, D I, Martin, P A, Norris, J M, Malik, R, Govendir, M (2008). Susceptibility of canine and feline Escherichia coli and canine Staphylococcus intermedius isolates to fluoroquinolones. Australian veterinary journal, 86(4), 147

1108. Damborg, Peter, Sorensen, Anne H, Guardabassi, Luca (2008). Monitoring of antimicrobial resistance in healthy dogs: first report of canine ampicillin-resistant Enterococcus faecium clonal complex 17. Veterinary microbiology, 132(1-2), 190

1109. Ball, Katherine R, Rubin, Joseph E, Chirino-Trejo, M, Dowling, Patricia M (2008). Antimicrobial resistance and prevalence of canine uropathogens at the Western College of Veterinary Medicine Veterinary Teaching Hospital, 2002-2007. The Canadian veterinary journal = La revue veterinaire canadienne, 49(10), 985

1110. Stenske, Katherine A, Bemis, David A, Gillespie, Barbara E, D'Souza, Doris H, Oliver, Stephen P, Draughon, Francis A, Matteson, Karla J, Bartges, Joseph W (2009). Comparison

of clonal relatedness and antimicrobial susceptibility of fecal Escherichia coli from healthy dogs and their owners. American journal of veterinary research, 70(9), 1108

1111. C Murphy, RJ Reid-Smith, JF Prescott, BN Bonnett, C Poppe, P Boerlin, JS Weese, N Janecko, SA McEwen (2009). Occurrence of antimicrobial resistant bacteria in healthy dogs and cats presented to private veterinary hospitals in southern Ontario: A preliminary study. The Canadian veterinary journal = La revue veterinaire canadienne, 50(10), #Pages#

1112. Shaheen, B W, Boothe, D M, Oyarzabal, O A, Smaha, T (2010). Antimicrobial resistance profiles and clonal relatedness of canine and feline Escherichia coli pathogens expressing multidrug resistance in the United States. Journal of veterinary internal medicine, 24(2), 323

1113. Nam, Hyang-Mi, Lee, Hee-Soo, Byun, Jae-Won, Yoon, Soon-Seek, Jung, Suk-Chan, Joo, Yi-Seok, Lim, Suk-Kyung (2010). Prevalence of antimicrobial resistance in fecal Escherichia coli isolates from stray pet dogs and hospitalized pet dogs in Korea. Microbial drug resistance (Larchmont, N.Y.), 16(1), 75

1114. Craven, M, Dogan, B, Schukken, A, Volkman, M, Chandler, A, McDonough, P L, Simpson, K W (2010). Antimicrobial resistance impacts clinical outcome of granulomatous colitis in boxer dogs. Journal of veterinary internal medicine, 24(4), 819

1115. Lei, Tao, Tian, Wei, He, Liu, Huang, Xian-Hui, Sun, Yong-Xue, Deng, Yu-Ting, Sun, Yan, Lv, Dian-Hong, Wu, Cong-Ming, Huang, Liang-Zong, Shen, Jian-Zhong, Liu, Jian-Hua (2010). Antimicrobial resistance in Escherichia coli isolates from food animals, animal food products and companion animals in China. Veterinary microbiology, 146(1-2), 85

1116. Hariharan, Harry, Matthew, Vanessa, Fountain, Jacqueline, Snell, Alicia, Doherty, Devin, King, Brittany, Shemer, Eran, Oliveira, Simone, Sharma, Ravindra N (2011). Aerobic bacteria from mucous membranes, ear canals, and skin wounds of feral cats in Grenada, and the antimicrobial drug susceptibility of major isolates. Comparative immunology, microbiology and infectious diseases, 34(2), 129

1117. Hubka, Patrick, Boothe, Dawn M (2011). In vitro susceptibility of canine and feline Escherichia coli to fosfomycin. Veterinary microbiology, 149(1-2), 277

1118. JA Davis, CR Jackson, PJ Fedorka-Cray, JB Barrett, JH Brousse, J Gustafson, M Kucher (2011). Anatomical distribution and genetic relatedness of antimicrobial-resistant Escherichia coli from healthy companion animals. Journal of applied microbiology, 110(2), #Pages#

1119. M Maaland, L Guardabassi (2011). In vitro antimicrobial activity of nitrofurantoin against Escherichia coli and Staphylococcus pseudintermedius isolated from dogs and cats. Veterinary microbiology, 151(3-4), #Pages#

1120. Leonard, Erin K, Pearl, David L, Finley, Rita L, Janecko, Nicol, Reid-Smith, Richard J, Peregrine, Andrew S, Weese, J Scott (2012). Comparison of antimicrobial resistance patterns of Salmonella spp. and Escherichia coli recovered from pet dogs from volunteer households in Ontario (2005-06). The Journal of antimicrobial chemotherapy, 67(1), 174

1121. Milani, C, Corro, M, Drigo, M, Rota, A (2012). Antimicrobial resistance in bacteria from breeding dogs housed in kennels with differing neonatal mortality and use of antibiotics. Theriogenology, 78(6), 1321

1122. Boothe, Dawn, Smaha, Tim, Carpenter, D Mark, Shaheen, Bashar, Hatchcock, Terri (2012). Antimicrobial resistance and pharmacodynamics of canine and feline pathogenic E. coli in the United States. Journal of the American Animal Hospital Association, 48(6), 379

1123. TD Procter, DL Pearl, RL Finley, EK Leonard, N Janecko, RJ Reid-Smith, JS Weese, AS Peregrine, JM Sargeant (2014). A cross-sectional study examining the prevalence and risk factors for anti-microbial-resistant generic Escherichia coli in domestic dogs that frequent dog parks in three cities in south-western Ontario, Canada. Zoonoses and public health, 61(4), #Pages#

1124. Hall, J L, Holmes, M A, Baines, S J (2013). Prevalence and antimicrobial resistance of canine urinary tract pathogens. The Veterinary record, 173(22), 549

1125. Leite-Martins, Liliana R, Mahu, Maria I M, Costa, Ana L, Mendes, Angelo, Lopes, Elisabete, Mendonca, Denisa M V, Niza-Ribeiro, Joao J R, de Matos, Augusto J F, da Costa, Paulo Martins (2014). Prevalence of antimicrobial resistance in enteric Escherichia coli from domestic pets and assessment of associated risk markers using a generalized linear mixed model. Preventive veterinary medicine, 117(1), 28

1126. Dorsch, Roswitha, von Vopelius-Feldt, Clara, Wolf, Georg, Straubinger, Reinhard K, Hartmann, Katrin (2015). Feline urinary tract pathogens: prevalence of bacterial species and antimicrobial resistance over a 10-year period. The Veterinary record, 176(8), 201

1127. Rzewuska, Magdalena, Czopowicz, Michal, Kizerwetter-Swida, Magdalena, Chrobak, Dorota, Blaszczak, Borys, Binek, Marian (2015). Multidrug resistance in Escherichia coli strains isolated from infections in dogs and cats in Poland (2007-2013). TheScientificWorldJournal, 2015(#issue#), 408205

1128. Cummings, Kevin J, Aprea, Victor A, Altier, Craig (2015). Antimicrobial resistance trends among canine Escherichia coli isolates obtained from clinical samples in the northeastern USA, 2004-2011. The Canadian veterinary journal = La revue veterinaire canadienne, 56(4), 393

1129. Windahl, Ulrika, Bengtsson, Bjorn, Nyman, Ann-Kristin, Holst, Bodil Strom (2015). The distribution of pathogens and their antimicrobial susceptibility patterns among canine surgical wound infections in Sweden in relation to different risk factors. Acta veterinaria Scandinavica, 57(#issue#), 11

1130. Wong, C, Epstein, S E, Westropp, J L (2015). Antimicrobial Susceptibility Patterns in Urinary Tract Infections in Dogs (2010-2013). Journal of veterinary internal medicine, 29(4), 1045

1131. MC Nolff, S Reese, M Fehr, R Dening, A Meyer-Lindenberg (2016). Assessment of wound bio-burden and prevalence of multi-drug resistant bacteria during open wound management. The Journal of small animal practice, 57(5), #Pages#

1132. Morrissey, Ian, Moyaert, Hilde, de Jong, Anno, El Garch, Farid, Klein, Ulrich, Ludwig, Carolin, Thiry, Julien, Youala, Myriam (2016). Antimicrobial susceptibility monitoring of bacterial pathogens isolated from respiratory tract infections in dogs and cats across Europe: ComPath results. Veterinary microbiology, 191(#issue#), 44

1133. Ludwig, C, de Jong, A, Moyaert, H, El Garch, F, Janes, R, Klein, U, Morrissey, I, Thiry, J, Youala, M (2016). Antimicrobial susceptibility monitoring of dermatological bacterial pathogens isolated from diseased dogs and cats across Europe (ComPath results). Journal of applied microbiology, 121(5), 1254

1134. McMeekin, C H, Hill, K E, Gibson, I R, Bridges, J P, Benschop, J (2017). Antimicrobial resistance patterns of bacteria isolated from canine urinary samples submitted to a New Zealand veterinary diagnostic laboratory between 2005-2012. New Zealand veterinary journal, 65(2), 99

1135. Daodu, Oluwafemi Babatunde, Amosun, Elizabeth Adesola, Oluwayelu, Daniel Oladimeji (2017). ANTIBIOTIC RESISTANCE PROFILING AND MICROBIOTA OF THE UPPER RESPIRATORY TRACT OF APPARENTLY HEALTHY DOGS IN IBADAN, SOUTH WEST NIGERIA. African journal of infectious diseases, 11(1), 1

1136. Moyaert, Hilde, Morrissey, Ian, de Jong, Anno, El Garch, Farid, Klein, Ulrich, Ludwig, Carolin, Thiry, Julien, Youala, Myriam (2017). Antimicrobial Susceptibility Monitoring of Bacterial Pathogens Isolated from Urinary Tract Infections in Dogs and Cats Across Europe: ComPath Results. Microbial drug resistance (Larchmont, N.Y.), 23(3), 391

1137. Saputra, Sugiyono, Jordan, David, Mitchell, Tahlia, Wong, Hui San, Abraham, Rebecca J, Kidsley, Amanda, Turnidge, John, Trott, Darren J, Abraham, Sam (2017). Antimicrobial resistance in clinical Escherichia coli isolated from companion animals in Australia. Veterinary microbiology, 211(#issue#), 43

1138. Yousefi, Amirhossein, Torkan, Saam (2017). Uropathogenic Escherichia coli in the Urine Samples of Iranian Dogs: Antimicrobial Resistance Pattern and Distribution of Antibiotic Resistance Genes. BioMed research international, 2017(#issue#), 4180490

1139. Qekwana, Daniel Nenene, Phophi, Lufuno, Naidoo, Vinny, Oguttu, James Wabwire, Odoi, Agricola (2018). Antimicrobial resistance among Escherichia coli isolates from dogs presented with urinary tract infections at a veterinary teaching hospital in South Africa. BMC veterinary research, 14(1), 228

1140. Awosile, Babafela B, McClure, J Trenton, Saab, Matthew E, Heider, Luke C (2018). Antimicrobial resistance in bacteria isolated from cats and dogs from the Atlantic Provinces, Canada from 1994-2013. The Canadian veterinary journal = La revue veterinaire canadienne, 59(8), 885

1141. Bourne, Judith A, Chong, Wye Li, Gordon, David M (2019). Genetic structure, antimicrobial resistance and frequency of human associated Escherichia coli sequence types among faecal isolates from healthy dogs and cats living in Canberra, Australia. PloS one, 14(3), e0212867

1142. Moyaert, H, de Jong, A, Simjee, S, Rose, M, Youala, M, El Garch, F, Vila, T, Klein, U, Rzewuska, M, Morrissey, I (2019). Survey of antimicrobial susceptibility of bacterial pathogens isolated from dogs and cats with respiratory tract infections in Europe: ComPath results. Journal of applied microbiology, 127(1), 29

1143. Campbell, O, Gagnon, J, Rubin, J E (2019). Antibacterial activity of chemotherapeutic drugs against Escherichia coli and Staphylococcus pseudintermedius. Letters in applied microbiology, 69(5), 353

1144. McGovern, Diana A, Gaschen, Frederic, Habil, Dr, Roy, Alma (2019). Antimicrobial Susceptibility Patterns and Clinical Parameters in 208 Dogs with Positive Urine Cultures (2012-2014). Journal of the American Animal Hospital Association, 55(6), 306

1145. KuKanich, Kate, Lubbers, Brian, Salgado, Brianna (2020). Amoxicillin and amoxicillin-clavulanate resistance in urinary Escherichia coli antibiograms of cats and dogs from the Midwestern United States. Journal of veterinary internal medicine, 34(1), 227

1146. Espinosa-Gongora, Carmen, Jessen, Lisbeth Rem, Kieler, Ida Nordang, Damborg, Peter, Bjornvad, Charlotte Reinhard, Gudeta, Dereje Dadi, Pires Dos Santos, Teresa, Sablier-Gallis, Frederique, Sayah-Jeanne, Sakina, Corbel, Tanguy, Neviere, Agathe, Hugon, Perrine, Saint-Lu, Nathalie, de Gunzburg, Jean, Guardabassi, Luca (2020). Impact of oral amoxicillin and amoxicillin/clavulanic acid treatment on bacterial diversity and beta-lactam resistance in the canine faecal microbiota. The Journal of antimicrobial chemotherapy, 75(2), 351

1147. AR Yamanaka, AT Hayakawa, ÍSM Rocha, V Dutra, VRF Souza, JN Cruz, LM Camargo, L Nakazato (2019). The Occurrence of Multidrug Resistant Bacteria in the Urine of Healthy Dogs and Dogs with Cystitis. Animals : an open access journal from MDPI, 9(12), #Pages#

1148. Bourely, Clemence, Coeffic, Thomas, Caillon, Jocelyne, Thibaut, Sonia, Cazeau, Geraldine, Jouy, Eric, Jarrige, Nathalie, Chauvin, Claire, Madec, Jean-Yves, Haenni, Marisa, Leblond, Agnes, Gay, Emilie (2020). Trends in antimicrobial resistance among Escherichia coli from defined infections in humans and animals. The Journal of antimicrobial chemotherapy, 75(6), 1525

1149. Joosten, Philip, Ceccarelli, Daniela, Odent, Evelien, Sarrazin, Steven, Graveland, Haitske, Van Gompel, Liese, Battisti, Antonio, Caprioli, Andrea, Franco, Alessia, Wagenaar, Jaap A, Mevius, Dik, Dewulf, Jeroen (2020). Antimicrobial Usage and Resistance in Companion Animals: A Cross-Sectional Study in Three European Countries. Antibiotics (Basel, Switzerland), 9(2), #Pages#

1150. Amadi, Victor A, Hariharan, Harry, Amadi, Ozioma A, Matthew-Belmar, Vanessa, Nicholas-Thomas, Roxanne, Perea, Marta Lanza, Carter, Kenrith, Rennie, Eugene, Kalasi, Keith, Alhassan, Andy, Kabuusu, Richard M, Alozie, Grant Ugochukwu, Fields, Paul J, Pinckney, Rhonda, Sharma, Ravindra (2019). Antimicrobial resistance patterns of commensal Escherichia coli isolated from feces of non-diarrheic dogs in Grenada, West Indies. Veterinary world, 12(12), 2070

1151. Werner, Melanie, Suchodolski, Jan S, Straubinger, Reinhard K, Wolf, Georg, Steiner, Jorg M, Lidbury, Jonathan A, Neuerer, Felix, Hartmann, Katrin, Unterer, Stefan (2020). Effect of amoxicillin-clavulanic acid on clinical scores, intestinal microbiome, and amoxicillin-resistant Escherichia coli in dogs with uncomplicated acute diarrhea. Journal of veterinary internal medicine, 34(3), 1166

1152. de Jong, Anno, Youala, Myriam, El Garch, Farid, Simjee, Shabbir, Rose, Markus, Morrissey, Ian, Moyaert, Hilde (2020). Antimicrobial susceptibility monitoring of canine and feline skin and ear pathogens isolated from European veterinary clinics: results of the ComPath Surveillance programme. Veterinary dermatology, 31(6), 431

1153. Gomez-Beltran, David A, Villar, David, Lopez-Osorio, Sara, Ferguson, Duncan, Monsalve, Laura K, Chaparro-Gutierrez, Jenny J (2020). Prevalence of Antimicrobial Resistance in Bacterial Isolates from Dogs and Cats in a Veterinary Diagnostic Laboratory in Colombia from 2016-2019. Veterinary sciences, 7(4), #Pages#

1154. Scarborough, Ri, Bailey, Kirsten, Galgut, Bradley, Williamson, Adam, Hardefeldt, Laura, Gilkerson, James, Browning, Glenn (2020). Use of Local Antibiogram Data and Antimicrobial Importance Ratings to Select Optimal Empirical Therapies for Urinary Tract Infections in Dogs and Cats. Antibiotics (Basel, Switzerland), 9(12), #Pages#

1155. L Marchetti, D Buldain, L Gortari Castillo, A Buchamer, M Chirino-Trejo, N Mestorino (2021). Pet and Stray Dogs as Reservoirs of Antimicrobial-Resistant <i>Escherichia coli</i>. International journal of microbiology, 2021(), #Pages#

1156. Li, Yanli, Fernandez, Ruben, Duran, Inma, Molina-Lopez, Rafael A, Darwich, Laila (2020). Antimicrobial Resistance in Bacteria Isolated From Cats and Dogs From the Iberian Peninsula. Frontiers in microbiology, 11(#issue#), 621597

1157. Amphaiphan, Chiradet, Yano, Terdsak, Som-In, Manita, Kungwong, Phunnapha, Wongsawan, Kanruethai, Pusoonthornthum, Rosama, Salman, Mo D, Tangtrongsup, Sahatchai (2021). Antimicrobial drug resistance profile of isolated bacteria in dogs and cats with urologic problems at Chiang Mai University Veterinary Teaching Hospital, Thailand (2012-2016). Zoonoses and public health, 68(5), 452

1158. Darwich, Laila, Seminati, Chiara, Burballa, Ares, Nieto, Alba, Duran, Inma, Tarradas, Nuria, Molina-Lopez, Rafael A (2021). Antimicrobial susceptibility of bacterial isolates from urinary tract infections in companion animals in Spain. The Veterinary record, 188(9), e60

1159. Mavrides, Daphne E, Morgan, Alice L, Na, Jea G, Graham, Peter A, McHugh, Timothy D (2022). Antimicrobial resistance profiles of bacteria associated with lower respiratory tract infections in cats and dogs in England. The Veterinary record, 190(4), e779

1160. Fonseca, J D, Mavrides, D E, Graham, P A, McHugh, T D (2021). Results of urinary bacterial cultures and antibiotic susceptibility testing of dogs and cats in the UK. The Journal of small animal practice, 62(12), 1085

1161. Cocca, Giorgia, Piva, Silvia, Magno, Sara Del, Scarpellini, Raffaele, Giacometti, Federica, Serraino, Andrea, Giunti, Massimo (2021). Prevalence and Patterns of Antimicrobial Resistance among Escherichia coli and Staphylococcus spp. in a Veterinary University Hospital. Veterinary sciences, 8(12), #Pages#

1162. Yudhanto, Setyo, Hung, Chien-Che, Maddox, Carol W, Varga, Csaba (2022). Antimicrobial Resistance in Bacteria Isolated From Canine Urine Samples Submitted to a Veterinary Diagnostic Laboratory, Illinois, United States. Frontiers in veterinary science, 9(#issue#), 867784

1163. Y Furuya, M Matsuda, S Harada, M Kumakawa, T Shirakawa, M Uchiyama, R Akama, M Ozawa, M Kawanishi, Y Shimazaki, H Sekiguchi (2022). Nationwide Monitoring of Antimicrobial-Resistant <i>Escherichia coli</i> and <i>Enterococcus</i> spp. Isolated From Diseased and Healthy Dogs and Cats in Japan. Frontiers in veterinary science, 9(), #Pages#

1164. Ekakoro, John E, Hendrix, G Kenitra, Guptill, Lynn F, Ruple, Audrey (2022). Antimicrobial susceptibility and risk factors for resistance among Escherichia coli isolated from canine specimens submitted to a diagnostic laboratory in Indiana, 2010-2019. PloS one, 17(8), e0263949

1165. OSK Chan, M Baranger-Ete, WWT Lam, P Wu, M Yeung, E Lee, H Bond, O Swan, HM Tun (2022). A Retrospective Study of Antimicrobial Resistant Bacteria Associated with Feline and Canine Urinary Tract Infection in Hong Kong SAR, China-A Case Study on Implication of First-Line Antibiotics Use. Antibiotics (Basel, Switzerland), 11(9), #Pages#

1166. Osman, Marwan, Albarracin, Belen, Altier, Craig, Grohn, Yrjo T, Cazer, Casey (2022). Antimicrobial resistance trends among canine Escherichia coli isolated at a New York veterinary diagnostic laboratory between 2007 and 2020. Preventive veterinary medicine, 208(#issue#), 105767

1167. Smoglica, Camilla, Evangelisti, Giulia, Fani, Caterina, Marsilio, Fulvio, Trotta, Michele, Messina, Francesca, Di Francesco, Cristina Esmeralda (2022). Antimicrobial Resistance Profile of Bacterial Isolates from Urinary Tract Infections in Companion Animals in Central Italy. Antibiotics (Basel, Switzerland), 11(10), #Pages#

1168. Garces, Andreia, Lopes, Ricardo, Silva, Augusto, Sampaio, Filipe, Duque, Daniela, Brilhante-Simoes, Paula (2022). Bacterial Isolates from Urinary Tract Infection in Dogs and Cats in Portugal, and Their Antibiotic Susceptibility Pattern: A Retrospective Study of 5 Years (2017-2021). Antibiotics (Basel, Switzerland), 11(11), #Pages#

1169. Aurich, Sophie, Prenger-Berninghoff, Ellen, Ewers, Christa (2022). Prevalence and Antimicrobial Resistance of Bacterial Uropathogens Isolated from Dogs and Cats. Antibiotics (Basel, Switzerland), 11(12), #Pages#

1170. Leet-Otley, Kendall, Fellman, Claire L, Wayne, Annie S, Beaulac, Kirthana, DeStefano, Ian M, Chambers, Kelly, Marino, Kady B, Doron, Shira (2023). Demonstrating the importance of local culture and susceptibility data: antibiograms from dogs at a veterinary tertiary care center. Journal of the American Veterinary Medical Association, 261(7), 1

1171. DJ Woerde, KL Reagan, BA Byrne, BC Weimer, SE Epstein, C Schlesener, BC Huang, JE Sykes (2023). Characteristics of Extended-Spectrum β-Lactamase Producing Enterobacterales Isolated from Dogs and Cats, 2011-2021. Veterinary sciences, 10(3), #Pages#

1172. Cocco, Antonio, Alessiani, Alessandra, Salini, Romolo, Iapaolo, Federica, Averaimo, Daniela, Pompilii, Cinzia, Foschi, Giovanni, Bellucci, Fabio, Iannino, Filomena, Dalla Villa, Paolo, Janowicz, Anna, Caporale, Marco (2023). Detection of Potential Zoonotic Agents Isolated in Italian Shelters and the Assessment of Animal Welfare Correlation with

Antimicrobial Resistance in Escherichia coli Strains. Antibiotics (Basel, Switzerland), 12(5), #Pages#

1173. I Habib, K Mohteshamuddin, MI Mohamed, GB Lakshmi, A Abdalla, A Bakhit Ali Alkaabi (2023). Domestic Pets in the United Arab Emirates as Reservoirs for Antibiotic-Resistant Bacteria: A Comprehensive Analysis of Extended-Spectrum Beta-Lactamase Producing <i>Escherichia coli</i> Prevalence and Risk Factors. Animals : an open access journal from MDPI, 13(10), #Pages#

1174. Hansen-Jones, Christian L, Hill, Kate E, Cogger, Naomi (2023). Feline urinary tract pathogens in western Canada: Prevalence of bacterial species and antimicrobial resistance from 2012 to 2018. The Canadian veterinary journal = La revue veterinaire canadienne, 64(6), 558

1175. Tsuyuki, Y., Fukushima, Y., Takahashi, T. (2022). Characteristics of bacteria isolated in 2017-2020 from Japanese companion animals using blood culture, compared to those isolated in 2014-2016. Journal of the Japanese Association for Infectious Diseases, 96(3), 112

1176. Rey, L. M. R., Guaitolini, C. R. de F., Fazoli, K. G. Z., Silva, L. L. da, Fendrigo, T. T., Santos, I. C. dos, Zaniolo, M. M., Martins, L. de A., Gonçalves, D. D. (2020). Microbiome and antimicrobial resistance in members of the Enterobacteriaceae family from vaginal and preputial mucous isolates of stray dogs. Acta Scientiae Veterinariae, 48(1774), #Pages#

1177. Andrade, A. C. S., Santos, I. C. dos, Barbosa, L. N., Caetano, I. C. da S., Zaniolo, M. M., Fonseca, B. D., Martins, L. de A., Gonçalves, D. D. (2019). Antimicrobial resistance and extended-spectrum beta-lactamase production in Enterobacteriaceae isolates from household cats (Felis silvestris catus). Acta Scientiae Veterinariae, 47(#issue#), #Pages#

1178. Hamilton, E., Kruger, J. M., Schall, W., Beal, M., Manning, S. D., Kaneene, J. B. (2013). Acquisition and persistence of antimicrobial-resistant bacteria isolated from dogs and cats admitted to a veterinary teaching hospital. Journal of the American Veterinary Medical Association, 243(7), 990

1179. Mustapha, M., Audu, Y., Ezema, K. U., Abdulkadir, J. U., Lawal, J. R., Balami, A. G., Adamu, L., Bukar-Kolo, Y. M. (2021). Antimicrobial susceptibility profiles of Escherichia coli isolates from diarrheic dogs in Maiduguri, Borno State, Nigeria. Macedonian Veterinary Review, 44(1), 47

1180. Šiugždaitė, J., Gabinaitienė, A., Kerzienė, S., Laurusevičius, S. A., Laurusevičius, T. (2018). Antimicrobial resistance of Escherichia coli isolated from healthy dogs in Lithuania. Veterinarski Arhiv, 88(3), 355

1181. Chawnan, N., Lampang, K. N., Mektrirat, R., Awaiwanont, N., Thongkorn, K. (2021). Cultivation of bacterial pathogens and antimicrobial resistance in canine periapical tooth abscesses. Veterinary Integrative Sciences, 19(3), 513

1182. Sobkowich, Kurtis E, Weese, J Scott, Poljak, Zvonimir, Plum, Andy, Szlosek, Donald, Bernardo, Theresa M (2023). Epidemiology of companion animal AMR in the United States of America: filling a gap in the one health approach. Frontiers in public health, 11(#issue#), 1161950

1183. Marco Werhahn Beining, Hartmann, Maria, Luebke-Becker, Antina, Guenther, Sebastian, Schaufler, Katharina, Hille, Katja, Kreienbrock, Lothar (2023). Carriage of Extended Spectrum Beta Lactamase-Producing Escherichia coli: Prevalence and Factors Associated with Fecal Colonization of Dogs from a Pet Clinic in Lower Saxony, Germany Animals, 13(4), 584

1184. Verma, Ashutosh, Carney, Kimberly, Taylor, Marina, Amsler, Kaitlyn, Morgan, Joey, Gruszynski, Karen, Erol, Erdal, Carter, Craig, Locke, Stephan, Ashton Callipare, Shah, Devendra H (2021). Occurrence of potentially zoonotic and cephalosporin resistant enteric bacteria among shelter dogs in the Central and South-Central Appalachia BMC Veterinary Research, 17(#issue#), 1

1185. Marchetti, Laura, Buldain, Daniel, Castillo, Lihuel Gortari, Buchamer, Andrea, Manuel Chirino-Trejo, Mestorino, Nora (2021). Pet and Stray Dogs as Reservoirs of Antimicrobial-Resistant Escherichia coli International Journal of Microbiology, 2021(#issue#), #Pages#

1186. ANYANWU, Madubuike U, UGWU, Ifeoma C, EZEKWELU, Martins O, OKORAFOR, Obianuju N (2017). Prevalence and Antibiogram of Generic Extended-Spectrum β-Lactam-Resistant Enterobacteria in Healthy Dogs Notulae Scientia Biologicae, 9(1), #Pages#

1187. Naziri, Zahra, Firouzi, Roya, Derakhshandeh, Abdollah, Tabrizi, Aidin Shojaee (2015). Comparative analysis of phylogenetic group and antimicrobial resistance pattern of fecal Escherichia coli isolates between healthy dogs and their owners Comparative Clinical Pathology, 24(5), 1211

1188. Jay-Russell, Michele T, Hake, Alexis F, Bengson, Yingjia, Thiptara, Anyarat, Tran, Nguyen (2014). Prevalence and Characterization of Escherichia coli and Salmonella Strains Isolated from Stray Dog and Coyote Feces in a Major Leafy Greens Production Region at the United States-Mexico Border PLoS One, 9(11), #Pages#

1189. Eze, C A, Ugwu, J, Eze, J I, Nnaji, T O, Nweze, N E, Ngene, A A (2014). Characterisation of the bacterial isolates from transmissible venereal tumour lesions of dogs

and their antibiogram resistance in Nsukka area, Southeastern Nigeria Comparative Clinical Pathology, 23(3), 641

1190. Harada, Kazuki, Morimoto, Erika, Kataoka, Yasushi, Takahashi, Toshio (2011). Clonal spread of antimicrobial-resistant Escherichia coli isolates among pups in two kennels Acta Veterinaria Scandinavica, 53(#issue#), 11

1191. Mustapha, Muhammad, Audu, Yusuf, Ezema, Kingsley Uwakwe, Abdulkadir, Jafar Umar, Lawal, Jallailudeen Rabana, Balami, Arhyel Gana, Adamu, Lawan, Bukar-Kolo, Yachilla Maryam (2021). Antimicrobial Susceptibility Profiles of Escherichia Coli Isolates from Diarrheic Dogs in Maiduguri, Borno State, Nigeria Macedonian veterinary review, 44(1), 47

1192. Zenatto, K, Vieira, TR, Sambrano, GE, Cardoso, M (2022). Antimicrobial Resistance Profile of Bacteria Isolated from Canine and Feline Samples at the Preventive Veterinary Laboratory of the Federal University of Rio Grande do Sul (UFRGS) - Brazil Acta Scientiae Veterinariae, 50(#issue#), #Pages#

1193. Leonard, EK, Pearl, DL, Janecko, N, Finley, RL, Reid-Smith, RJ, Weese, JS, Peregrine, AS (2015). Risk factors for carriage of antimicrobial-resistant Salmonella spp and Escherichia coli in pet dogs from volunteer households in Ontario, Canada, in 2005 and 2006 American Journal Of Veterinary Research, 76(11), 959

1194. Dazio, V, Nigg, A, Schmidt, JS, Brilhante, M, Mauri, N, Kuster, SP, Brawand, SG, Schupbach-Regula, G, Willi, B, Endimiani, A, Perreten, V, Schuller, S (2021). Acquisition and carriage of multidrug-resistant organisms in dogs and cats presented to small animal practices and clinics in Switzerland Journal Of Veterinary Internal Medicine, 35(2), 970

1195. Yamanaka, AR, Hayakawa, AT, Rocha, ISM, Dutra, V, Souza, VRF, Cruz, JN, Camargo, LM, Nakazato, L (2019). The Occurrence of Multidrug Resistant Bacteria in the Urine of Healthy Dogs and Dogs with Cystitis ANIMALS, 9(12), #Pages#

1196. Lawrence, M, KuKanich, K, KuKanich, B, Heinrich, E, Coetzee, JF, Grauer, G, Narayanan, S (2013). Effect of cefovecin on the fecal flora of healthy dogs Veterinary Journal, 198(1), 259

1197. Oh, YI, Kim, HJ, Kim, YM, Kim, SS, Kim, JK, Kim, HW, Kang, BJ, Youn, HY (2017). Antimicrobial Resistance of Bacterial Isolates from Positive Urine Culture in Four Hundred Five Dogs Between 2013-2014 International Journal Of Applied Research In Veterinary Medicine, 15(2), 99

1198. Ojo, OE, Bello, AO, Amosun, EA, Ajadi, RA (2014). Multidrug resistant verocytotoxin-producing Escherichia coli O157:H7 in the faeces of diarrhoeic and non-diarrhoeic dogs in Abeokuta, Nigeria Veterinarski Arhiv, 84(1), 63

1199. Bennett, AB, Martin, PA, Gottlieb, SA, Govendir, M (2013). In vitro susceptibilities of feline and canine Escherichia coli and Pseudomonas spp. isolates to ticarcillin and ticarcillin-clavulanic acid Australian Veterinary Journal, 91(5), 171

1200. Adesiyun, AA, Campbell, M, Kaminjolo, JS (1997). Prevalence of bacterial enteropathogens in pet dogs in Trinidad Journal Of Veterinary Medicine Series B-Infectious Diseases And Veterinary Public Health, 44(1), 19

Data charted from studies that were included or deemed eligible

# Article	Name of data extractors	Date of data extraction	start_year	end_year	study_country	study_level	first_affil	first_country	study_design_reported	study_design	source_pop	clinical_history	categories	recent_antimicrobial	settings	specimen
1093	Rasaq Ojasanya, Uththami Kukathasan	5 Aug 2023 - 14 Sep 2023	1989	1997	United Kingdom	Regional	University of Glasgow	United Kingdom	Not reported	Observational	Yes	Yes	Sick	No	Veterinary clinic	Not reported
1094	Rasaq Ojasanya, Uththami Kukathasan	6 Aug 2023 - 14 Sep 2023	1997	1999	Spain	Regional	Universidad de La Rioja	Spain	Not reported	Observational	Yes	No	Not reported	No	Veterinary clinic	Not reported
1095	Rasaq Ojasanya, Uththami Kukathasan	7 Aug 2023 - 14 Sep 2023	1996	1998	United States	Regional	University of California Davis	United States	Observational	Observational	Yes	Yes	Sick	No	Veterinary clinic	urinary tract
1096	Rasaq Ojasanya, Uththami Kukathasan	8 Aug 2023 - 14 Sep 2023	1992	2001	United States	Individual	University of Missouri – Columbia	United States	Not reported	Observational	Yes	No	Not reported	No	Veterinary clinic	urinary tract
1097	Rasaq Ojasanya, Uththami Kukathasan	9 Aug 2023 - 14 Sep 2023	Not reported	Not reported	Belgium	Individual	Ghent University	Belgium	Not reported	Observational	Yes	Yes	Healthy	Yes	Household, shelter	other
1098	Rasaq Ojasanya, Uththa	10 Aug 2023 -	1996	1997	United States	Individual	University of Minnesota	United States	Observational	Observational	Yes	No	Not reported	No	Stray	other

	mi Kukathasan	14 Sep 2023																
1099	Rasaq Ojasanya, Uththami Kukathasan	11 Aug 2023 - 14 Sep 2023	1997	1998	Finland	Regional	University of Helsinki	Finland	Not reported	Observational	Yes	Yes	Sick & Healthy	Yes	Veterinary clinic	other
1100	Rasaq Ojasanya, Uththami Kukathasan	12 Aug 2023 - 14 Sep 2023	2002	2003	United States	Regional	Michigan State University	United States	Observational	Observational	Yes	No	Not reported	No	Household	other
1101	Rasaq Ojasanya, Uththami Kukathasan	13 Aug 2023 - 14 Sep 2023	Not reported	Not reported	Belgium	Regional	Ghent University	Belgium	Not reported	Observational	Yes	Yes	Sick	Yes	Household, shelter, veterinary clinic	other
1102	Rasaq Ojasanya, Uththami Kukathasan	14 Aug 2023 - 14 Sep 2023	1990	2003	Canada	Regional	Université de Montréal	Canada	Not reported	Observational	Yes	No	Not reported	No	Veterinary clinic	Not reported
1103	Rasaq Ojasanya, Uththami Kukathasan	15 Aug 2023 - 14 Sep 2023	2003	2003	Canada	Individual	University of Guelph	Canada	Observational	Observational	Yes	Yes	Sick	No	Veterinary clinic	other
1104	Rasaq Ojasanya, Uththami Kukathasan	16 Aug 2023 - 14 Sep 2023	2000	2005	Denmark	Individual	University of Copenhagen	Denmark	Not reported	Observational	Yes	Yes	Sick	No	Not reported	both
1105	Rasaq Ojasanya, Uththami	17 Aug 2023 - 14 Sep 2023	1999	2000	United States	Individual	Louisiana State University	United States	Observational	Observational	Yes	Yes	Sick	Yes	Veterinary clinic	both

	Kukathasan																
1106	Rasaq Ojasanya, Uththami Kukathasan	18 Aug 2023 - 14 Sep 2023	2008	2010	Czech Republic, France, Germany, Hungary, Italy, The Netherlands, Poland, Spain and the United Kingdom	International	IHMA Europe Sàrl	Switzerland	Not reported	Ongoing AMR monitoring or surveillance programs	Yes	Yes	Sick	No	Household	Not reported
1107	Rasaq Ojasanya, Uththami Kukathasan	19 Aug 2023 - 14 Sep 2023	2004	2007	Australia	Individual	University of Sydney	Australia	Observational	Observational	Yes	No	Not reported	No	Veterinary clinic	both
1108	Rasaq Ojasanya, Uththami Kukathasan	20 Aug 2023 - 14 Sep 2023	2006	2006	Denmark	Regional	University of Copenhagen	Denmark	Not reported	Observational	Yes	Yes	Healthy	No	Veterinary clinic	other
1109	Rasaq Ojasanya, Uththami Kukathasan	21 Aug 2023 - 14 Sep 2023	2002	2007	Canada	Individual	University of Saskatchewan	Canada	Not reported	Observational	Yes	No	Not reported	No	Veterinary clinic	urinary tract
1110	Rasaq Ojasanya, Uththami Kukathasan	22 Aug 2023 - 14 Sep 2023	Not reported	Not reported	United States	Individual	University of Tennessee	United States	Observational	Observational	Yes	No	Not reported	No	Household	other
1111	Rasaq Ojasanya, Uththami Kukathasan	23 Aug 2023 - 14 Sep 2023	2002	2002	Canada	Regional	University of Guelph	Canada	Observational	Observational	Yes	Yes	Healthy	No	Veterinary clinic	other

1112	Rasaq Ojasanya, Uththami Kukathasan	24 Aug 2023 - 14 Sep 2023	2005	2005	United States	Regional	Auburn University	United States	Not reported	Observational	Yes	No	Not reported	No	Veterinary clinic	both
1113	Rasaq Ojasanya, Uththami Kukathasan	25 Aug 2023 - 14 Sep 2023	2006	2007	Republic of Korea	National	National Veterinary Research and Quarantine Service	Republic of Korea	Not reported	Observational	Yes	No	Not reported	No	Shelter, veterinary clinic	other
1114	Rasaq Ojasanya, Uththami Kukathasan	26 Aug 2023 - 14 Sep 2023	Not reported	Not reported	United States	Individual	Cornell University	United States	Observational	Observational	Yes	Yes	Sick & Healthy	Yes	Veterinary clinic	other
1115	Rasaq Ojasanya, Uththami Kukathasan	27 Aug 2023 - 14 Sep 2023	2007	2009	Grenada	Regional	St. George's University	Grenada	Not reported	Observational	Yes	No	Not reported	No	Stray	other
1116	Rasaq Ojasanya, Uththami Kukathasan	28 Aug 2023 - 14 Sep 2023	2008	2010	United States	National	Auburn University	United States	Not reported	Observational	Yes	Yes	Sick	No	Veterinary clinic	both
1117	Rasaq Ojasanya, Uththami Kukathasan	29 Aug 2023 - 14 Sep 2023	2007	2007	United States	Regional	USDA Agricultural Research Service	United States	Not reported	Observational	Yes	Yes	Healthy	No	Veterinary clinic	other
1118	Rasaq Ojasanya, Uththami Kukathasan	30 Aug 2023 - 14 Sep 2023	1998	2010	unknown	Individual	University of Copenhagen	Denmark	Not reported	Observational	Yes	Yes	Sick & Healthy	No	Not reported	both

1119	Rasaq Ojasanya, Uththami Kukathasan	31 Aug 2023 - 14 Sep 2023	2005	2006	Canada	Regional	University of Guelph	Canada	Observational	Observational	Yes	No	Not reported	No	House hold	other
1120	Rasaq Ojasanya, Uththami Kukathasan	32 Aug 2023 - 14 Sep 2023	2006	2009	Italy	Regional	University of Padova	Italy	Observational	Observational	Yes	Yes	Healthy	Yes	Shelter	other
1121	Rasaq Ojasanya, Uththami Kukathasan	33 Aug 2023 - 14 Sep 2023	2005	2005	United States	National	Auburn University	United States	Not reported	Observational	Yes	Yes	Sick	No	Veterinary clinic	both
1122	Rasaq Ojasanya, Uththami Kukathasan	34 Aug 2023 - 14 Sep 2023	1999	2009	United Kingdom	Individual	University of Cambridge	United Kingdom	Not reported	Observational	Yes	No	Not reported	No	Veterinary clinic	urinary tract
1123	Rasaq Ojasanya, Uththami Kukathasan	35 Aug 2023 - 14 Sep 2023	2009	2012	Portugal	Individual	Porto University	Portugal	Observational	Observational	Yes	No	Not reported	No	House hold, veterinary clinic	other
1124	Rasaq Ojasanya, Uththami Kukathasan	36 Aug 2023 - 14 Sep 2023	2000	2009	Germany	Individual	Ludwig Maximilian University	Germany	Observational	Observational	Yes	No	Not reported	No	Veterinary clinic	urinary tract
1125	Rasaq Ojasanya, Uththami Kukathasan	37 Aug 2023 - 14 Sep 2023	2007	2013	Poland	Individual	Warsaw University of Life Sciences	Poland	Not reported	Observational	Yes	Yes	Sick	No	Veterinary clinic	both

1126	Rasaq Ojasanya, Uththami Kukathasan	38 Aug 2023 - 14 Sep 2023	2004	2011	United States	Regional	Texas A&M University	United States	Observational	Observational	Yes	Yes	Sick	No	Veterinary clinic	both
1127	Rasaq Ojasanya, Uththami Kukathasan	39 Aug 2023 - 14 Sep 2023	2008	2010	Sweden	Regional	National Veterinary Institute, SVA	Sweden	Not reported	Observational	Yes	Yes	Sick	Yes	Veterinary clinic	other
1128	Rasaq Ojasanya, Uththami Kukathasan	40 Aug 2023 - 14 Sep 2023	2010	2013	United States	Individual	University of California	United States	Observational	Observational	Yes	Yes	Sick	Yes	Veterinary clinic	urinary tract
1129	Rasaq Ojasanya, Uththami Kukathasan	41 Aug 2023 - 14 Sep 2023	2011	2013	Germany	Regional	Ludwig Maximilian University	Germany	Observational	Observational	Yes	Yes	Sick	Yes	Veterinary clinic	other
1130	Rasaq Ojasanya, Uththami Kukathasan	42 Aug 2023 - 14 Sep 2023	2008	2010	Czech Republic, France, Germany, Hungary, Italy, The Netherlands, Poland, Spain and the United Kingdom	International	IHMA Europe Sàrl	Switzerland	Not reported	Ongoing AMR monitoring or surveillance programs	Yes	Yes	Sick	No	House hold, shelter, veterinary clinic	other
1131	Rasaq Ojasanya, Uththami Kukathasan	43 Aug 2023 - 14 Sep 2023	2008	2010	Czech Republic, France, Germany, Hungary, Italy, the Netherlands, Poland,	International	CEESA ComPath Study Group	Belgium	Not reported	Ongoing AMR monitoring or surveillance programs	Yes	Yes	Sick	No	House hold, shelter, veterinary clinic	other

					Spain, Sweden and the United Kingdom											
1132	Rasaq Ojasanya, Uththami Kukathasan	44 Aug 2023 - 14 Sep 2023	2005	2012	New Zealand	Individual	Massey University	New Zealand	Observational	Observational	Yes	No	Not reported	No	Veterinary clinic	urinary tract
1133	Rasaq Ojasanya, Uththami Kukathasan	45 Aug 2023 - 14 Sep 2023	Not reported	Not reported	Nigeria	Individual	University of Ilorin	Nigeria	Not reported	Observational	Yes	Yes	Healthy	No	Household, shelter	other
1134	Rasaq Ojasanya, Uththami Kukathasan	46 Aug 2023 - 14 Sep 2023	2008	2010	Czech Republic, France, Germany, Hungary, Italy, the Netherlands, Poland, Spain, Sweden and the United Kingdom	International	CEESA ComPath Study Group	Belgium	Not reported	Ongoing AMR monitoring or surveillance programs	Yes	Yes	Sick	No	Household, shelter, veterinary clinic	other
1135	Rasaq Ojasanya, Uththami Kukathasan	47 Aug 2023 - 14 Sep 2023	2013	2014	Australia	Regional	University of Adelaide	Australia	Not reported	Observational	Yes	Yes	Sick	No	Veterinary clinic	urinary tract
1136	Rasaq Ojasanya, Uththami Kukathasan	48 Aug 2023 - 14 Sep 2023	2015	2016	Iran	Individual	Islamic Azad University	Iran	Not reported	Observational	Yes	Yes	Sick	No	Veterinary clinic	urinary tract
1137	Rasaq Ojasanya,	49 Aug 2023 -	2007	2012	South Africa	Individual	University of Pretoria	South Africa	Observational	Observational	Yes	Yes	Sick	No	Household, veterin	urinary tract

	Uththami Kukathasan	14 Sep 2023													ary clinic	
1138	Rasaq Ojasanya, Uththami Kukathasan	50 Aug 2023 - 14 Sep 2023	1994	2013	Canada	Individual	University of Prince Edward Island	Canada	Not reported	Observational	Yes	Yes	Sick	No	Veterinary clinic	both
1139	Rasaq Ojasanya, Uththami Kukathasan	51 Aug 2023 - 14 Sep 2023	2015	2017	Australia	Individual	Australian National University	Australia	Not reported	Observational	Yes	No	Not reported	No	Household, shelter	other
1140	Rasaq Ojasanya, Uththami Kukathasan	52 Aug 2023 - 14 Sep 2023	2013	2014	Belgium, Czech Republic, France, Germany, Hungary, Italy, The Netherlands, Poland, Spain, Switzerland and the United Kingdom	International	CEESA ComPath Study Group	Belgium	Not reported	Ongoing AMR monitoring or surveillance programs	Yes	Yes	Sick	No	Veterinary clinic	other
1141	Rasaq Ojasanya, Uththami Kukathasan	53 Aug 2023 - 14 Sep 2023	Not reported	Not reported	Canada	Individual	University of Saskatchewan	Canada	Not reported	Observational	Yes	Yes	Sick	No	Veterinary clinic	Not reported
1142	Rasaq Ojasanya, Uththami Kukathasan	54 Aug 2023 - 14 Sep 2023	2012	2014	United States	Individual	Louisiana State University	United States	Not reported	Observational	No	No	Not reported	No	Veterinary clinic	urinary tract

1143	Rasaq Ojasanya, Uththami Kukathasan	55 Aug 2023 - 14 Sep 2023	2013	2017	United States	Regional	Kansas State University	United States	Observational	Observational	Yes	No	Not reported	No	Veterinary clinic	both
1144	Rasaq Ojasanya, Uththami Kukathasan	56 Aug 2023 - 14 Sep 2023	Not reported	Not reported	Denmark	Regional	University of Copenhagen	Denmark	Experimental	Randomized controlled trial	Yes	Yes	Healthy	Yes	Veterinary clinic	other
1145	Rasaq Ojasanya, Uththami Kukathasan	57 Aug 2023 - 14 Sep 2023	2016	2016	Brazil	Individual	University of Cuiaba	Brazil	Observational	Observational	Yes	Yes	Sick & Healthy	No	Veterinary clinic	urinary tract
1146	Rasaq Ojasanya, Uththami Kukathasan	58 Aug 2023 - 14 Sep 2023	2014	2017	France	Regional	E´cole Nationale des Service Ve´ te´ rinaires, Universite´ de Lyon	France	Not reported	Ongoing AMR monitoring or surveillance programs	No	No	Not reported	No	Veterinary clinic	Not reported
1147	Rasaq Ojasanya, Uththami Kukathasan	59 Aug 2023 - 14 Sep 2023	2015	2016	Belgium, Italy, The Netherlands	International	Ghent University	Belgium	Observational	Observational	Yes	Yes	Healthy	No	Veterinary clinic	other
1148	Rasaq Ojasanya, Uththami Kukathasan	60 Aug 2023 - 14 Sep 2023	2016	2016	Grenada	National	St. George's University	Grenada	Not reported	Observational	Yes	No	Not reported	No	Household	other
1149	Rasaq Ojasanya, Uththami	61 Aug 2023 - 14 Sep 2023	2016	2018	Germany	Regional	Ludwig Maximilian University	Germany	Experimental	Randomized controlled trial	Yes	Yes	Healthy	No	Veterinary clinic	other

	Kukathasan																
1150	Rasaq Ojasanya, Uththami Kukathasan	62 Aug 2023 - 14 Sep 2023	2013	2014	Belgium, Czech Republic, France, Germany, Hungary, Italy, the Netherlands, Poland, Spain, Sweden, Switzerland and the UK	International	CEESA ComPath Study Group	Belgium	Not reported	Ongoing AMR monitoring or surveillance programs	Yes	Yes	Sick	No	Veterinary clinic	other
1151	Rasaq Ojasanya, Uththami Kukathasan	63 Aug 2023 - 14 Sep 2023	2016	2019	Colombia	Individual	Universidad de Antioquia	Colombia	Observational	Observational	Yes	Yes	Sick	No	Veterinary clinic	both
1152	Rasaq Ojasanya, Uththami Kukathasan	64 Aug 2023 - 14 Sep 2023	2015	2019	Australia	Individual	University of Melbourne	Australia	Observational	Observational	Yes	Yes	Sick	No	Veterinary clinic	urinary tract
1153	Rasaq Ojasanya, Uththami Kukathasan	65 Aug 2023 - 14 Sep 2023	2016	2016	Argentina	Regional	Universidad Nacional de La Plata	Argentina	Not reported	Observational	Yes	Yes	Healthy	No	Household	other
1154	Rasaq Ojasanya, Uththami Kukathasan	66 Aug 2023 - 14 Sep 2023	2016	2018	Spain	Regional	Universitat Autònoma de Barcelona	Spain	Observational	Observational	Yes	Yes	Sick	No	Veterinary clinic	other
1155	Rasaq Ojasanya, Uththami	67 Aug 2023 - 14 Sep 2023	2012	2016	Thailand	Individual	Chiang Mai University	Thailand	Not reported	Observational	Yes	Yes	Sick	No	Veterinary clinic	urinary tract

	Kukathasan																
1156	Rasaq Ojasanya, Uththami Kukathasan	68 Aug 2023 - 14 Sep 2023	2016	2018	Spain	Individual	Universitat Autònoma de Barcelona	Spain	Observational	Observational	Yes	Yes	Sick	No	Veterinary clinic	urinary tract
1157	Rasaq Ojasanya, Uththami Kukathasan	69 Aug 2023 - 14 Sep 2023	2002	2012	United Kingdom	Individual	University of Cambridge	United Kingdom	Observational	Observational	Yes	Yes	Sick	No	Veterinary clinic	other
1158	Rasaq Ojasanya, Uththami Kukathasan	70 Aug 2023 - 14 Sep 2023	2011	2012	United Kingdom	Individual	University College London	United Kingdom	Observational	Observational	Yes	Yes	Sick	No	Veterinary clinic	urinary tract
1159	Rasaq Ojasanya, Uththami Kukathasan	71 Aug 2023 - 14 Sep 2023	2018	2019	Italy	Individual	University of Bologna	Italy	Observational	Observational	Yes	Yes	Sick & Healthy	No	Veterinary clinic	other
1160	Rasaq Ojasanya, Uththami Kukathasan	72 Aug 2023 - 14 Sep 2023	2019	2020	United States	Individual	University of Illinois	United States	Not reported	Observational	Yes	Yes	Sick	No	Veterinary clinic	urinary tract
1161	Rasaq Ojasanya, Uththami Kukathasan	73 Aug 2023 - 14 Sep 2023	2018	2020	Japan	Regional	National Veterinary Assay Laboratory, Ministry of Agriculture, Forestry and Fisheries	Japan	Not reported	Ongoing AMR monitoring or surveillance programs	Yes	Yes	Sick & Healthy	No	Veterinary clinic	other

1162	Rasaq Ojasanya, Uththami Kukathasan	74 Aug 2023 - 14 Sep 2023	2010	2019	United States	National	Cornell University	United States	Not reported	Observational	Yes	No	Not reported	No	Veterinary clinic	both
1163	Rasaq Ojasanya, Uththami Kukathasan	75 Aug 2023 - 14 Sep 2023	2018	2020	China	Regional	University of Hong Kong	China	Observational	Observational	Yes	No	Not reported	No	Veterinary clinic	urinary tract
1164	Rasaq Ojasanya, Uththami Kukathasan	76 Aug 2023 - 14 Sep 2023	2007	2020	United States	Individual	Cornell University	United States	Observational	Observational	Yes	Yes	Sick	No	Veterinary clinic	both
1165	Rasaq Ojasanya, Uththami Kukathasan	77 Aug 2023 - 14 Sep 2023	2020	2020	Italy	Regional	University of Teramo	Italy	Observational	Observational	Yes	Yes	Sick	No	Veterinary clinic	urinary tract
1166	Rasaq Ojasanya, Uththami Kukathasan	78 Aug 2023 - 14 Sep 2023	2017	2021	Portugal	Regional	University of Trás-os-Montes	Portugal	Observational	Observational	Yes	No	Not reported	No	Veterinary clinic	urinary tract
1167	Rasaq Ojasanya, Uththami Kukathasan	79 Aug 2023 - 14 Sep 2023	2019	2020	Germany	Regional	Justus Liebig University Giessen	Germany	Observational	Observational	Yes	No	Not reported	No	Veterinary clinic	urinary tract
1168	Rasaq Ojasanya, Uththami Kukathasan	80 Aug 2023 - 14 Sep 2023	2019	2020	United States	Individual	Tufts University	United States	Not reported	Observational	Yes	No	Not reported	No	Veterinary clinic	both

1169	Rasaq Ojasanya, Uththami Kukathasan	81 Aug 2023 - 14 Sep 2023	2011	2021	United States	Individual	University of California Davis	United States	Not reported	Observational	Yes	No	Not reported	No	Veterinary clinic	urinary tract
1170	Rasaq Ojasanya, Uththami Kukathasan	82 Aug 2023 - 14 Sep 2023	Not reported	Not reported	Italy	Regional	Istituto Zooprofilattico Sperimentale dell'Abruzzo e Molise	Italy	Observational	Observational	Yes	Yes	Healthy	No	Shelter	other
1171	Rasaq Ojasanya, Uththami Kukathasan	83 Aug 2023 - 14 Sep 2023	2012	2018	Canada	Regional	Massey University	New Zealand	Not reported	Observational	Yes	No	Not reported	No	Veterinary clinic	urinary tract
1172	Rasaq Ojasanya, Uththami Kukathasan	84 Aug 2023 - 14 Sep 2023	2019	2021	United States	National	University of Guelph	Canada	Not reported	Observational	Yes	No	Not reported	No	Veterinary clinic	urinary tract
1173	Rasaq Ojasanya, Uththami Kukathasan	85 Aug 2023 - 14 Sep 2023	2014	2020	Japan	Regional	Kitasato University	Japan	Not reported	Observational	No	No	Not reported	No	Veterinary clinic	other
1174	Rasaq Ojasanya, Uththami Kukathasan	86 Aug 2023 - 14 Sep 2023	2019	2020	Brazil	Regional	Universidade Paranaense	Brazil	Not reported	Observational	Yes	No	Not reported	No	Stray	other
1175	Rasaq Ojasanya, Uththami Kukathasan	87 Aug 2023 - 14 Sep 2023	Not reported	Not reported	Brazil	Individual	Universidade Paranaense	Brazil	Not reported	Observational	Yes	Yes	Healthy	No	Household, veterinary clinic	other

1176	Rasaq Ojasanya, Uththami Kukathasan	88 Aug 2023 - 14 Sep 2023	2007	2009	United States	Individual	Michigan State University	United States	Observational	Observational	Yes	No	Not reported	No	Veterinary clinic	other
1177	Rasaq Ojasanya, Uththami Kukathasan	89 Aug 2023 - 14 Sep 2023	2019	2019	Nigeria	Regional	University of Maiduguri	Nigeria	Not reported	Observational	Yes	No	Not reported	No	House hold, stray	other
1178	Rasaq Ojasanya, Uththami Kukathasan	90 Aug 2023 - 14 Sep 2023	Not reported	Not reported	Lithuania	Regional	Lithuanian University of Health Science	Lithuania	Not reported	Observational	Yes	Yes	Healthy	No	House hold, shelter	other
1179	Rasaq Ojasanya, Uththami Kukathasan	91 Aug 2023 - 14 Sep 2023	2019	2020	Thailand	Individual	Chiang Mai University	Thailand	Not reported	Observational	Yes	Yes	Healthy	No	Veterinary clinic	other
1180	Rasaq Ojasanya, Uththami Kukathasan	92 Aug 2023 - 14 Sep 2023	2007	2008	China	Regional	South China Agricultural University	China	Not reported	Observational	Yes	Yes	Healthy	No	Veterinary clinic	other
1181	Rasaq Ojasanya, Uththami Kukathasan	93 Aug 2023 - 14 Sep 2023	2009	2009	Canada	Regional	University of Guelph	Canada	Observational	Observational	Yes	Yes	Healthy	Yes	House hold	other
1182	Rasaq Ojasanya, Uththami Kukathasan	94 Aug 2023 - 14 Sep 2023	2022	2022	United Arab Emirates	Regional	United Arab of Emirates University, Alexandria	United Arab Emirates	Not reported	Observational	Yes	Yes	Healthy	No	Veterinary clinic	other

							University									
1183	Rasaq Ojasanya, Uththami Kukathasan	95 Aug 2023 - 14 Sep 2023	2016	2017	Germany	Individual	University of Veterinary Medicine	Germany	Observational	Observational	Yes	Yes	Healthy	Yes	Veterinary clinic	other
1184	Rasaq Ojasanya, Uththami Kukathasan	96 Aug 2023 - 14 Sep 2023	2019	2019	United States	Regional	Center for Infectious, Zoonotic and Vector-borne diseases, College of Veterinary Medicine	United States	Not reported	Observational	Yes	Yes	Healthy	No	Shelter	other
1185	Rasaq Ojasanya, Uththami Kukathasan	97 Aug 2023 - 14 Sep 2023	2016	2016	Argentina	Regional	Universidad Nacional de La Plata	Argentina	Not reported	Observational	Yes	Yes	Healthy	No	Household, stray	other
1186	Rasaq Ojasanya, Uththami Kukathasan	98 Aug 2023 - 14 Sep 2023	2016	2016	Nigeria	Regional	University of Nigeria	Nigeria	Observational	Observational	Yes	Yes	Healthy	No	Household	other
1187	Rasaq Ojasanya, Uththami Kukathasan	99 Aug 2023 - 14 Sep 2023	2013	2014	Iran	Regional	Shiraz University	Iran	Not reported	Observational	Yes	Yes	Healthy	No	Household	other
1188	Rasaq Ojasanya, Uththami	100 Aug 2023 - 14 Sep 2023	2010	2011	United States	Regional	University of California Davis	United States	Not reported	Observational	Yes	No	Not reported	No	Shelter	other

	Kukathasan															
1189	Rasaq Ojasanya, Uththami Kukathasan	101 Aug 2023 - 14 Sep 2023	Not reported	Not reported	Nigeria	Regional	University of Nigeria	Nigeria	Not reported	Observational	Yes	Yes	Sick & Healthy	No	Market, veterinary clinic	other
1190	Rasaq Ojasanya, Uththami Kukathasan	102 Aug 2023 - 14 Sep 2023	Not reported	Not reported	Japan	Regional	Nippon Veterinary and Life Science University	Japan	Not reported	Observational	Yes	Yes	Healthy	No	Shelter	other
1191	Rasaq Ojasanya, Uththami Kukathasan	103 Aug 2023 - 14 Sep 2023	2019	2019	Nigeria	Regional	University of Maiduguri	Nigeria	Not reported	Observational	Yes	No	Not reported	No	House hold	other
1192	Rasaq Ojasanya, Uththami Kukathasan	104 Aug 2023 - 14 Sep 2023	2016	2020	Brazil	Individual	Universidade Federal do Rio Grande do Sul	Brazil	Descriptive	Observational	Yes	No	Not reported	No	Veterinary clinic	both
1193	Rasaq Ojasanya, Uththami Kukathasan	105 Aug 2023 - 14 Sep 2023	2005	2006	Canada	Regional	University of Guelph	Canada	Observational	Observational	Yes	No	Not reported	No	House hold	other
1194	Rasaq Ojasanya, Uththami Kukathasan	106 Aug 2023 - 14 Sep 2023	2018	2018	Switzerland	Regional	University of Bern	Switzerland	Observational	Observational	Yes	No	Not reported	No	Veterinary clinic	other
1195	Rasaq Ojasanya, Uththami	107 Aug 2023 - 14 Sep 2023	2016	2016	Brazil	Regional	University of Cuiaba	Brazil	Not reported	Observational	Yes	Yes	Sick & Healthy	No	Veterinary clinic	urinary tract

	Kukathasan															
1196	Rasaq Ojasanya, Uththami Kukathasan	108 Aug 2023 - 14 Sep 2023	Not reported	Not reported	United States	Individual	Kansas State University	United States	Experimental	Randomized controlled trial	Yes	Yes	Healthy	No	Household	other
1197	Rasaq Ojasanya, Uththami Kukathasan	109 Aug 2023 - 14 Sep 2023	2013	2014	Republic of Korea	Individual	Seoul National University	Republic of Korea	Not reported	Observational	Yes	No	Not reported	No	Veterinary clinic	urinary tract
1198	Rasaq Ojasanya, Uththami Kukathasan	110 Aug 2023 - 14 Sep 2023	Not reported	Not reported	Nigeria	Regional	University of Agriculture Abeokuta	Nigeria	Not reported	Observational	Yes	No	Not reported	No	Veterinary clinic	other
1199	Rasaq Ojasanya, Uththami Kukathasan	111 Aug 2023 - 14 Sep 2023	2004	2011	Australia	Regional	University of Sydney	Australia	Not reported	Observational	Yes	No	Not reported	No	Veterinary clinic	both
1200	Rasaq Ojasanya, Uththami Kukathasan	112 Aug 2023 - 14 Sep 2023	1993	1995	Trinidad	Individual	University of the West Indies	Trinidad	Not reported	Observational	Yes	Yes	Sick & Healthy	No	Veterinary clinic	other

bacteria_type	prev_estimate	animals_tested	dogs_tested	cats_tested	isolates_tested	isolates_dogs	isolates_cats	anim_iso_tested	anim_iso_dogs	anim_iso_cats	total_isolates_tested	dog_isolates_tested	cat_isolates_tested	AST_method	MIC_reported	antimicrobials_tested
E. coli	isolate				4198									disk diffusion	No	9
E. coli	isolate				4197									disk diffusion	No	16
E. coli	isolate				56	56								broth microdilution	No	6
E. coli	isolate				547	547								broth microdilution	Yes	2
E. coli	isolate				154	154								disk diffusion	Yes	18
E. coli	isolate				45	45								disk diffusion	No	12
E. coli	isolate				98	98								disk diffusion	Yes	15
E. coli	isolate				21									disk diffusion	No	12
E. coli	isolate				88		88							disk diffusion, broth microdilution	Yes	16
E. coli	isolate				450	337	113							disk diffusion	No	12
E. coli	isolate				413	413								disk diffusion	No	12
E. coli	isolate				570	570								broth microdilution	Yes	17
E. coli	prevalence at the animal level and the isolate level							108	108		89	89		Not reported	No	7

E. coli	isolate				24	24								broth microdilution	Yes	10
E. coli	unclear													disk diffusion, broth dilution, e-test	Yes	4
E. coli	isolate				117	117								disk diffusion	No	12
E. coli	isolate													disk diffusion	No	10
E. coli	isolate				183	183								disk diffusion	No	17
Both E. coli and ESBL	prevalence at the animal level and the isolate level							227	188	39	1135	940	195	disk diffusion, broth dilution	Yes	11
E. coli	isolate				376	301	75							e-test	Yes	7
E. coli	isolate				628	628								disk diffusion	No	15
E. coli	animal	31	31											broth microdilution	Yes	18
E. coli	isolate				54		54							disk diffusion	No	6
E. coli	isolate				275	275								broth microdilution, e-test	Yes	9
E. coli	isolate				317	199	118							broth microdilution	Yes	16
Both E. coli and ESBL	isolate				106									broth microdilution	Yes	1

E. coli	prevalence at the animal level and the isolate level							133	133		395	395		broth microdilution	Yes	15
E. coli	isolate				50	50								disk diffusion	No	5
E. coli	isolate				376	301	75							e-test	Yes	7
E. coli	isolate													disk diffusion	No	17
E. coli	isolate				396	307	89							disk diffusion	No	19
E. coli	isolate				157		157							disk diffusion	No	9
E. coli	isolate				730	401	329							disk diffusion	No	13
E. coli	isolate				3519	3519								broth microdilution	Yes	11
E. coli	isolate				20	20								broth microdilution	Yes	11
E. coli	isolate				858	858								Not reported	No	5
E. coli	isolate													Not reported	No	12
E. coli	isolate				34	24	10							broth microdilution	Yes	10
E. coli	animal	122	108	14										broth microdilution	Yes	13
E. coli	isolate				1082	1082								disk diffusion	No	6
E. coli	isolate				40	40								disk diffusion	No	10

Both E. coli and ESBL	isolate				122	108	14							broth microdilution	Yes	14
E. coli	isolate				855	514	341							broth microdilution	Yes	15
E. coli	isolate				270	270								disk diffusion	No	16
E. coli	isolate				168	168								disk diffusion	No	15
E. coli	isolate				4193	3364	829							disk diffusion	No	14
E. coli	isolate				537	203	334							disk diffusion	No	8
E. coli	isolate				55	33	22							broth microdilution	Yes	16
E. coli	isolate				32	32								broth microdilution	Yes	6
E. coli	animal	208	208											disk diffusion	No	8
E. coli	isolate				783	640	143							broth microdilution	Yes	10
E. coli	animal	42	42											Not reported	No	2
E. coli	isolate				54	54								disk diffusion	No	15
E. coli	isolate													Not reported	No	5
E. coli	isolate				285	148	137							broth microdilution	Yes	14
E. coli	isolate				402	402								disk diffusion	No	12
E. coli	animal	8	8											Not reported	No	1

E. coli	animal	138	138											broth microd ilution	Yes	18
E. coli	isolate				176	163	13							disk diffusi on	No	12
E. coli	isolate				3294	2058	1236							disk diffusi on	No	6
Both E. coli and ESBL	animal	95	95											disk diffusi on	No	20
E. coli	isolate				449	405	44							disk diffusi on	Yes	35
E. coli	isolate				33	33								disk diffusi on	No	9
E. coli	isolate				152	116	36							disk diffusi on	No	24
E. coli	isolate				194	164	30							disk diffusi on	No	16
E. coli	isolate				731	637	94							disk diffusi on	No	10
E. coli	isolate				58	58								disk diffusi on	No	11
E. coli	isolate				366	366								broth microd ilution	Yes	19
E. coli	animal	1873	999	874										broth microd ilution	Yes	14
E. coli	isolate				2738	2738								broth microd ilution	Yes	35
E. coli	isolate				28	16	12							disk dilutio n	No	32
E. coli	isolate				6765	6765								broth microd ilution	Yes	22
E. coli	isolate				343	263	80							broth microd ilution	No	26

E. coli	isolate				5306	2730	2576							broth microdilution	Yes	21
E. coli	isolate				489	332	157							broth microdilution	Yes	15
E. coli	isolate				275	275								broth microdilution	Yes	16
Both E. coli and ESBL	isolate				30	29	1							broth microdilution	Yes	28
E. coli	isolate				54	54								broth microdilution	No	14
E. coli	isolate				4847		4847							disk dilution, broth microdilution	Yes	11
E. coli	isolate				510482	388417	122065							disk dilution, broth microdilution	No	8
Both E. coli and ESBL	isolate				138	127	11							disk dilution, broth microdilution	No	19
E. coli	isolate				13	13								disk diffusion	No	19
Extended spectrum beta-lactamases (ESBL)	isolate				68		68							disk diffusion	No	20
E. coli	isolate				766									broth microdilution	Yes	14

E. coli	isolate				147	147								disk diffusion	No	10
E. coli	isolate				48	48								e-test	Yes	11
E. coli	isolate				12	12								disk diffusion	No	15
E. coli	isolate				244	187	57							broth microdilution	No	14
E. coli	prevalence at the animal level and the isolate level							237	237		709	709		broth microdilution	Yes	15
Extended spectrum beta-lactamases (ESBL)	isolate				77	77								disk diffusion	No	12
Extended spectrum beta-lactamases (ESBL)	unclear													broth microdilution	No	
E. coli	isolate				18	18								broth microdilution	No	19
Both E. coli and ESBL	isolate				95	95								disk diffusion	No	20
E. coli	isolate				27	27								disk diffusion	No	10
E. coli	isolate				160	160								disk diffusion	No	13
E. coli	isolate				18	18								broth microdilution	Yes	12

E. coli	isolate				7	7								disk diffusion	No	11
Both E. coli and ESBL	prevalence at the animal level and the isolate level							43	43		86	86		broth microdilution	Yes	11
E. coli	animal	147	147											disk diffusion	No	10
E. coli	isolate				157	157								disk diffusion, broth microdilution	No	11
E. coli	prevalence at the animal level and the isolate level							133	133		395	395		broth microdilution	Yes	15
E. coli	prevalence at the animal level and the isolate level							271			50			broth microdilution	Yes	14
E. coli	prevalence at the animal level and the isolate level							114	114		54	54		disk diffusion	No	15
E. coli	isolate				15	15								disk diffusion	Yes	12
E. coli	isolate				154	154								disk diffusion	No	15

E. coli	isolate				22	22								disk diffusion	No	11
E. coli	isolate				148	83	65							disk diffusion, e-test	Yes	2
E. coli	isolate				99	99								disk diffusion	No	8

carbapenems_test_reported	MDR_reported	consistent_MDR_reporting	guidelines_reported	intermediate_category_handing	same_panel_testing
No	Yes	Yes	Not reported	Not reported	No
No	Yes	Yes	CLSI	Not reported	Yes
No	No		CLSI	Reported separately	Yes
No	No		CLSI	Reported separately	Yes
No	Yes	Yes	CLSI	Combined with resistance data (reported as non-susceptible)	No
No	Yes	Yes	CLSI	Combined with susceptible data (reported as non-resistant)	Yes
No	Yes	Yes	CLSI	Combined with resistance data (reported as non-susceptible)	Yes
No	Yes	Yes	CLSI	Not reported	Yes
No	Yes	No	CLSI	Not reported	Yes
No	No		CLSI	Not reported	No
No	No		CLSI	Combined with resistance data (reported as non-susceptible)	Yes
No	No		CLSI	Combined with susceptible data (reported as non-resistant)	Yes
No	No		Not reported	Not reported	No
No	No		CLSI, EUCAST	Reported separately	No
No	No		CLSI	Reported separately	Yes
No	Yes	No	CLSI	Reported separately	Yes
No	No		CLSI	Combined with resistance data (reported as non-susceptible)	Yes
Yes	Yes	Yes	CLSI	Reported separately	Yes
No	Yes	No	CLSI	Combined with resistance data (reported as non-susceptible)	Yes
No	Yes	No	CLSI	Combined with resistance data (reported as non-susceptible)	Yes
No	Yes	Yes	CLSI	Not reported	Yes
Yes	No		CLSI	Reported separately	Yes
No	No		CLSI	Not reported	Yes
No	Yes	No	CLSI	Reported separately	Yes
No	Yes	No	CLSI	Reported separately	Yes
No	No		CLSI	Not reported	Yes
No	Yes	No	CLSI	Combined with susceptible data (reported as non-resistant)	Yes
No	Yes	No	CLSI	Reported separately	Yes
No	No		CLSI	Combined with resistance data (reported as non-susceptible)	Yes
Yes	No		CLSI	Not reported	Yes
Yes	Yes	Yes	CLSI	Combined with susceptible data (reported as non-resistant)	Yes

Yes	No		CLSI	Combined with susceptible data (reported as non-resistant)	Yes
No	Yes	Yes	CLSI	Combined with resistance data (reported as non-susceptible)	Yes
No	Yes	No	CLSI	Combined with susceptible data (reported as non-resistant)	Yes
No	Yes	Yes	CLSI, EUCAST	Reported separately	Yes
No	Yes	Yes	CLSI	Combined with resistance data (reported as non-susceptible)	Yes
Yes	Yes	Yes	CLSI	Not reported	Yes
No	No		CLSI, EUCAST	Reported separately	Yes
No	No		CLSI, EUCAST	Reported separately	Yes
No	No		CLSI	Combined with resistance data (reported as non-susceptible)	No
No	Yes	Yes	Not reported	Not reported	Yes
No	No		CLSI, EUCAST	Reported separately	Yes
Yes	Yes	Yes	CLSI	Combined with resistance data (reported as non-susceptible)	Yes
No	No		CLSI	Not reported	Yes
Yes	Yes	Yes	CLSI	Combined with resistance data (reported as non-susceptible)	Yes
No	Yes	Yes	CLSI	Not reported	Yes
Yes	No		Other	Reported separately	Yes
No	No		CLSI	Reported separately	Yes
No	Yes	No	CLSI	Not reported	Yes
No	Yes	Yes	CLSI	Reported separately	Yes
No	No		CLSI	Reported separately	Yes
No	No		Not reported	Not reported	Yes
Yes	Yes	Yes	CLSI	Not reported	Yes
No	No		Other, EUCAST	Combined with resistance data (reported as non-susceptible)	Yes
Yes	Yes	Yes	Other, EUCAST	Not reported	Yes
Yes	Yes	Yes	CLSI	Reported separately	Yes
No	No		CLSI	Not reported	Yes
No	No		CLSI, EUCAST	Reported separately	Yes
No	Yes	Yes	CLSI	Not reported	Yes
No	Yes	Yes	Other	Not reported	Yes
Yes	Yes	Yes	CLSI	Not reported	Yes
Yes	No		CLSI	Combined with resistance data (reported as non-susceptible)	Yes
No	Yes	Yes	CLSI	Not reported	Yes
Yes	Yes	Yes	CLSI	Combined with resistance data (reported as non-susceptible)	Yes
No	Yes	Yes	CLSI	Not reported	No
No	No		CLSI	Reported separately	Yes
Yes	Yes	Yes	CLSI	Combined with resistance data (reported as non-susceptible)	Yes
Yes	Yes	Yes	CLSI	Combined with resistance data (reported as non-susceptible)	Yes
Yes	No		CLSI, EUCAST	Not reported	Yes
Yes	Yes	Yes	CLSI	Reported separately	Yes
Yes	Yes	Yes	CLSI	Not reported	Yes
Yes	Yes	Yes	CLSI	Combined with resistance data (reported as non-susceptible)	No
Yes	Yes	Yes	CLSI, EUCAST	Not reported	Yes
Yes	Yes	Yes	CLSI	Reported separately	Yes

No	Yes	Yes	CLSI, EUCAST	Reported separately	Yes
Yes	No		CLSI	Reported separately	Yes
Yes	Yes	Yes	CLSI	Reported separately	Yes
Yes	Yes	Yes	CLSI	Not reported	Yes
No	Yes	Yes	CLSI	Reported separately	Yes
No	No		CLSI	Combined with resistance data (reported as non-susceptible)	Yes
Yes	No		CLSI	Reported separately	Yes
No	Yes	Yes	Other	Reported separately	Yes
Yes	Yes	No	CLSI	Not reported	Yes
No	Yes	No	CLSI	Reported separately	Yes
No	Yes	Yes	CLSI	Reported separately	Yes
No	Yes	Yes	CLSI, EUCAST	Not reported	Yes
Yes	No		CLSI	Reported separately	Yes
No	Yes	No	CLSI	Combined with susceptible data (reported as non-resistant)	Yes
No	Yes	No	CLSI	Combined with susceptible data (reported as non-resistant)	Yes
Yes	Yes	Yes	CLSI	Reported separately	Yes
No	No		CLSI	Not reported	Yes
Yes	Yes	Yes	CLSI	Not reported	Yes
Yes	Yes	Yes	CLSI	Not reported	Yes
No	Yes	Yes	CLSI, EUCAST	Reported separately	Yes
Yes	Yes	Yes	CLSI	Combined with resistance data (reported as non-susceptible)	Yes
No	No		CLSI	Not reported	Yes
No	No		Other	Not reported	Yes
No	Yes	No	CLSI	Not reported	Yes
No	Yes	Yes	CLSI	Reported separately	Yes
No	Yes	Yes	CLSI	Not reported	Yes
No	No		CLSI	Combined with susceptible data (reported as non-resistant)	Yes
Yes	Yes	Yes	CLSI, EUCAST, Other	Not reported	Yes
Yes	Yes	Yes	CLSI	Not reported	Yes
Yes	No		CLSI	Not reported	Yes
Yes	Yes	No	CLSI	Combined with resistance data (reported as non-susceptible)	Yes
No	Yes	No	CLSI	Not reported	Yes
No	No		CLSI	Reported separately	Yes
No	No		Not reported	Not reported	Yes

Variable names and the full questions that were asked

Variable names	Questions asked
start_year	What was the start year of the data used in the study?
end_year	What was the end year of the data used in the study?
study_country	In which country(ies) was the study conducted?
study_level	What was the level at which the study was conducted?
first_affil	What was the affiliation of the first author? (University name)
first_country	In which country is the institution of the first author located?
study_design_reported	What was the study design as reported by the authors for this research?
study_design	What was the study design as assessed by the reviewers?
source_pop	Was the source population of animals reported or defined by the authors [either explicitly reported or easily interpretable by the reader]?
clinical_history	Was the clinical history of the animal(s) disclosed?
categories	Classify the health status of the animals enrolled in the study
recent_antimicrobial	Was the recent history of prescribed antimicrobials disclosed?
settings	Where did the animal from which the sample was collected come from?
specimen	From which specimen was E. coli isolated?
bacteria_type	What type of bacteria was the study based on?
prev_estimate	What was the prevalence estimate of E. coli or ESBL that had antimicrobial susceptibility testing based on?
animals_tested	If the antimicrobial resistance rate was based on animal level, how many animals were tested?
dogs_tested	If the antimicrobial resistance rate was based on animal level, how many dogs were tested?
cats_tested	If the antimicrobial resistance rate was based on animal level, how many cats were tested?
isolates_tested	If the antimicrobial resistance rate was based on isolate level, how many isolates were tested?
isolates_dogs	If the antimicrobial resistance rate was based on isolate level, how many isolates were tested from dogs?
isolates_cats	If the antimicrobial resistance rate was based on isolate level, how many isolates were tested from cats?
anim_iso_tested	If the antimicrobial resistance rate was based on both animal and isolate levels, how many animals were tested?

anim_iso_dogs	If the antimicrobial resistance rate was based on both animal and isolate levels, how many isolates were tested from dogs?
anim_iso_cats	If the antimicrobial resistance rate was based on both animal and isolate levels, how many isolates were tested from cats?
total_isolates_tested	If the antimicrobial resistance rate was based on both animal and isolate levels, how many isolates were tested?
dog_isolates_tested	If the antimicrobial resistance rate was based on both animal and isolate levels, how many isolates were tested from dogs?
cat_isolates_tested	If the antimicrobial resistance rate was based on both animal and isolate levels, how many isolates were tested from cats?
AMR_method	What was the AMR testing method for bacterial isolates?
MIC_reported	Was the minimum inhibition concentration (MIC) reported?
antimicrobials_tested	What was the number of antimicrobials that were tested against E. coli isolates? (count how many antimicrobials that were tested)
carbapenems_test_reported	Was there a Carbapenems (e.g., Imipenem) test reported for E. coli or ESBL?
MDR_reported	Was multi-drug resistance (MDR) reported for E. coli?
consistent_MDR_reporting	Was it consistent with MDR reporting (i.e., resistance to one or more drugs in three or more classes?)
guidelines_reported	What guidelines did the researchers' report follow?
Edition	Which edition?
intermediate_category_handling	How was the intermediate category handled?
same_panel_tested	Were all bacterial isolates recovered in the study (inclusive of E. coli) tested against the same panel of antimicrobials?
