# Supplementary material for: Antimicrobial-resistance of Escherichia coli in dogs and cats: A scoping review
Source: PLoS One. 2025 May 30;20(5):e0323246. doi: 10.1371/journal.pone.0323246 (PMC12124559; doi:10.1371/journal.pone.0323246)
Supplement: S2 Table — (PDF) [file pone.0323246.s006.pdf]

S2 Table. Frequency of conflicts and the corresponding Kappa coefficient at each of the screening and data charting stages of the study selection process.

Study selection process	Number of studies reviewed	Number of studies with conflict	Kappa coefficient before conflict resolution	Kappa coefficient after conflict resolution
Primary screening	1,205	36	0.60	0.75
Secondary screening	153	27	0.89	0.97
Data charting	108	24	0.75	0.98
