# Supplementary material for: Antimicrobial-resistance of Escherichia coli in dogs and cats: A scoping review
Source: PLoS One. 2025 May 30;20(5):e0323246. doi: 10.1371/journal.pone.0323246 (PMC12124559; doi:10.1371/journal.pone.0323246)
Supplement: S3 Table — (PDF) [file pone.0323246.s007.pdf]

S3 Table. Affiliations of the first authors involved in research on the antimicrobial resistance of *E. coli* in dogs and/or cats.

	Affiliation of the first author	Country of affiliation	Number of studies
	University of Guelph	Canada	6 (5.6%)
	European Animal Health Study Centre (CEESA) ComPath Study	Belgium	4 (3.7%)
	University of Copenhagen	Denmark	4 (3.7%)
	Ludwig Maximilian University	Germany	3 (2.8%)
	Auburn University	United States	3 (2.8%)
	Cornell University	United States	3 (2.8%)
	University of California Davis	United States	3 (2.8%)
	Chiang Mai University	Thailand	2 (1.9%)
	IHMA Europe Sàrl	Switzerland	2 (1.9%)
	Kansas State University	United States	2 (1.9%)
	Louisiana State University	United States	2 (1.9%)
	Massey University	New Zealand	2 (1.9%)
	Michigan State University	United States	2 (1.9%)
	St. George's University	Grenada	2 (1.9%)
	Universidad Nacional de La Plata	Argentina	2 (1.9%)
	Universidade Paranaense	Brazil	2 (1.9%)
	Universitat Autònoma de Barcelona	Spain	2 (1.9%)
	University of Maiduguri	Nigeria	2 (1.9%)
	University of Cambridge	United Kingdom	2 (1.9%)
	University of Cuiaba	Brazil	2 (1.9%)
	University of Nigeria	Nigeria	2 (1.9%)
	University of Saskatchewan	Canada	2 (1.9%)
	University of Sydney	Australia	2 (1.9%)
	Australian National University	Australia	1 (0.9%)
	Center for Infectious, Zoonotic and Vector-borne Diseases, College of Veterinary Medicine	United States	1 (0.9%)

	E´cole Nationale des Service Ve´ te´ rinaires, Universite´ de Lyon	France	1 (0.9%)
	Islamic Azad University	Iran	1 (0.9%)
	Istituto Zooprofilattico Sperimentale..	Italy	1 (0.9%)
	Justus Liebig University Giessen	Germany	1 (0.9%)
	Kitasato University	Japan	1 (0.9%)
	Lithuanian University of Health Science	Lithuania	1 (0.9%)
	National Veterinary Assay Laboratory, Ministry of Agriculture, Forestry and Fisheries	Japan	1 (0.9%)
	National Veterinary Institute, SVA	Sweden	1 (0.9%)
	National Veterinary Research and Quarantine Service	Republic of Korea	1 (0.9%)
	Nippon Veterinary and Life Science University	Japan	1 (0.9%)
	Porto University	Portugal	1 (0.9%)
	Seoul National University	South Korea	1 (0.9%)
	Shiraz University	Iran	1 (0.9%)
	South China Agricultural University	China	1 (0.9%)
	Texas A&M University	United States	1 (0.9%)
	Tufts University	United States	1 (0.9%)
	USDA Agricultural Research Service	United States	1 (0.9%)
	United Arab of Emirates University	United Arab Emirates	1 (0.9%)
	Universidad de Antioquia	Colombia	1 (0.9%)
	Universidad de La Rioja	Spain	1 (0.9%)
	Universidade Federal do Rio Grande d..	Brazil	1 (0.9%)
	University College London	United Kingdom	1 (0.9%)
	University of Adelaide	Australia	1 (0.9%)
	University of Agriculture Abeokuta	Nigeria	1 (0.9%)
	University of Bern	Switzerland	1 (0.9%)
	University of Bologna	Italy	1 (0.9%)
	University of California	United States	1 (0.9%)
	University of Glasgow	United Kingdom	1 (0.9%)
	University of Helsinki	Finland	1 (0.9%)
	University of Hong Kong	Hong Kong	1 (0.9%)

	University of Illinois	United States	1 (0.9%)
	University of Ilorin	Nigeria	1 (0.9%)
	University of Melbourne	Australia	1 (0.9%)
	University of Minnesota	United States	1 (0.9%)
	University of Missouri– Columbia	United States	1 (0.9%)
	University of Padova	Italy	1 (0.9%)
	University of Pretoria	South Africa	1 (0.9%)
	University of Prince Edward Island	Canada	1 (0.9%)
	University of Tennessee	United States	1 (0.9%)
	University of Teramo	Italy	1 (0.9%)
	University of Trás-os-Montes	Portugal	1 (0.9%)
	University of Veterinary Medicine	Germany	1 (0.9%)
	University of the West Indies	Trinidad	1 (0.9%)
	Université de Montréal	Canada	1 (0.9%)
	Warsaw University of Life Sciences	Poland	1 (0.9%)
