# Supplementary material for: Antimicrobial-resistance of Escherichia coli in dogs and cats: A scoping review
Source: PLoS One. 2025 May 30;20(5):e0323246. doi: 10.1371/journal.pone.0323246 (PMC12124559; doi:10.1371/journal.pone.0323246)
Supplement: S4 Table — (PDF) [file pone.0323246.s008.pdf]

S4 Table. Cross-tabulation of the study designs as evaluated by the reviewers with study designs reported by the authors of the research on the antimicrobial resistance of *E. coli* in dogs and/or cats.

	Study designs, as reported by the authors. n (%)				
**Study Design as evaluated by the reviewers**	**Descriptive**	**Hypothesis testing (Experiment)**	**Hypothesis testing (Observational)**	**No study design was reported**	**Total no. of study**
Observational	1 (100.0)	0 (0.0)	40 (100.0)	56 (87.5)	97 (89.8)
Antimicrobial monitoring and surveillance program	0 (0.0)	0 (0.0)	0 (0.0)	8 (12.5)	8 (7.4)
Randomized controlled trial	0 (0.0)	3 (100.0)	0 (0.0)	0 (0.0)	3 (2.8)
Total	1 (0.9)	3 (2.8)	40 (37.0)	64 (59.3)	108
