# Supplementary material for: Antimicrobial-resistance of Escherichia coli in dogs and cats: A scoping review
Source: PLoS One. 2025 May 30;20(5):e0323246. doi: 10.1371/journal.pone.0323246 (PMC12124559; doi:10.1371/journal.pone.0323246)
Supplement: Attachment — Submitted filename: Response to Reviewers.docx [file pone.0323246.s010.docx]

Dear Editor,

We appreciate the opportunity to submit a revised version of our manuscript titled, “Antimicrobial-resistance of *Escherichia coli* in dogs and cats: a scoping review”.

We found the comments helpful and have revised our manuscript accordingly. Please see attached for a detailed point-by-point response to each of the issues raised by the reviewers and editor.

Sincerely,

Rasaq Ojasanya

| **Comments** | **Responses and Rationale** | **Action taken/location of changes in manuscript**  (i.e., page and line number in manuscript with track changes) |
| --- | --- | --- |
| **Editor** |  |  |
| 1. Please ensure that your manuscript meets PLOS ONE's style requirements, including those for file naming. | The abstract and the main body of the manuscript have been adjusted to meet PLOS ONE’s style. | In the abstract, the title of this manuscript has been revised to use lowercase for "A scoping review." The word "and" has been removed from the list of authors' names, the email address of the corresponding author has been provided, and the authors' initials have been included.  The author contributions section has been provided on lines 552 to 562 of page 35.  In the main body of the manuscript, the capitalization of each sectional title has been adjusted so that only the first letter is capitalized, with all others revised to lowercase.  The supplementary information has been separated, with each file having its own individual copy. |
| 2. Thank you for stating the following financial disclosure: [The authors would like to thank IDEXX Laboratories for their support of the IDEXX Chair in Emerging Technologies and Preventive Healthcare, which funded this research.].  Please state what role the funders took in the study.  If the funders had no role, please state: "The funders had no role in study design, data collection and analysis, decision to publish, or preparation of the manuscript." If this statement is not correct you must amend it as needed. Please include this amended Role of Funder statement in your cover letter; we will change the online submission form on your behalf | The role of the funder has been corrected based on your recommendation | The statement, “The funders had no role in study design, data collection and analysis, decision to publish, or preparation of the manuscript,” has been added to line 550, page 35. |
| 3. As required by our policy on Data Availability, please ensure your manuscript or supplementary information includes the following:   A numbered table of all studies identified in the literature search, including those that were excluded from the analyses.  For every excluded study, the table should list the reason(s) for exclusion.  If any of the included studies are unpublished, include a link (URL) to the primary source or detailed information about how the content can be accessed.  A table of all data extracted from the primary research sources for the systematic review and/or meta-analysis. The table must include the following information for each study:  Name of data extractors and date of data extraction  Confirmation that the study was eligible to be included in the review.  All data extracted from each study for the reported systematic review and/or meta-analysis that would be needed to replicate your analyses.  If data or supporting information were obtained from another source (e.g. correspondence with the author of the original research article), please provide the source of data and dates on which the data/information were obtained by your research group.  If applicable for your analysis, a table showing the completed risk of bias and quality/certainty assessments for each study or outcome.  Please ensure this is provided for each domain or parameter assessed. For example, if you used the Cochrane risk-of-bias tool for randomized trials, provide answers to each of the signalling questions for each study. If you used GRADE to assess certainty of evidence, provide judgements about each of the quality of evidence factor. This should be provided for each outcome.  An explanation of how missing data were handled. | The supplementary information includes:   1. A numbered table listing all studies from the literature search, including excluded ones with reasons for exclusion 2. A detailed table of extracted data from eligible studies used in the scoping review, including:  - data extractors' names and extraction dates - study eligibility confirmation - all extracted data required to replicate analyses   As this is a scoping review, no bias analysis was conducted. Missing data were categorized as “information not reported” and quantified in the results section of the manuscript. | This revision has been made to the supplementary information and has been provided in S1 Table in line 779, page 45 |
| 4. We note that Figure 2 in your submission contain [map/satellite] images which may be copyrighted. | I confirm that I am the original creator of Figure 2. | I grant PLOS ONE permission to publish this figure under the Creative Commons Attribution License (CC BY 4.0). |
| **Reviewer #1** |  |  |
| Figures 1,2,3 and 4 are not visible. Those must be incorporated accordingly.  Data in Tables is shown in some tables as n(%) while in Table-4 n(%) is mentioned in the title. All tables must be symmetric | Figures 1, 2, 3, and 4 have now been incorporated into the manuscript to ensure they are visible.  The presentation of data in all tables has been reviewed and updated to maintain consistency. | Figure 1 is located at line 193, Figure 2 at line 211, Figure 3 at line 234, and Figure 4 at line 244.  The notation "n(%)" has been deleted from the title of Table 4. Additionally, for simplicity, Table 5 has been divided into three separate tables, spanning lines 320 to 360. |
| **Reviewer #2** |  |  |
| 1. Because the study is more towards systematic search, I would like to recommend to put “Systematic and a scoping review” at the end of article title. | Thank you for this suggestion. While we acknowledge that a systematic search was conducted, we believe it is important to clarify that the scope of this study does not extend to a systematic review. Our concern is that putting systematic in the title would result in expectation that a risk of bias has been conducted in these papers in a comprehensive, detailed and systematic way; which is not the expectation if scoping review has been done. | As such, we prefer to retain the current title to accurately reflect the study's objectives. |
| 2. In the search strategy, the authors should use “*Enterobacteriaceae*; *Enterobacterales*” in order to make sure that there is no article left for *E. coli*. | Thank you for this suggestion | Revision has been made on page 6. |
| 3. At the end of introduction section, the authors should provide the study rationale and justification. What is the need to conduct study on *E. coli* only? There are plenty of other organism related to the animals. | Thank you for this suggestion | The justification has been added in line 59, page 3. |
| 4. In the articles included, did the authors found any information regarding the type of cats and dogs, which means whether they were pets or street animals? | We found articles that enrolled stray or wandering pets in their studies | Information regarding the primary study settings from which the pets originated is provided in the Results section of the manuscript (Line 272, Page 15) |
| 5. The use of abbreviations is appropriate. The authors should carefully check again the use of abbreviations throughout the manuscript. for example, at line 50 and 51 the word “antimicrobial resistance” is used twice but the abbreviation was mentioned on the second time while any abbreviation should be mentioned at it’s first appearance. | Thank you for this observation | Revisions have been made to lines 50 and 51 to properly abbreviate AMR. Subsequent corrections have also been applied to lines 99 and 535 to ensure consistency. |
| 6. The authors should carefully check the organism’s name and follow the same throughout the manuscript. For example, at some place they mentioned full form of *Escherichia coli*, and at certain levels only *E. coli*. | Thank you for this observation | Revision has been made to lines 55, 99, and 535 |
| 7. Line 52-53: The authors should mention the background only related to the companion animals. Remove the irrelevant background. | Thank you for your suggestion | This has been removed on lines 49 to 50 |
| 8. Line 73: Mention about how the protocol was prepared. | Thank you for your suggestion | This has been revised in lines 70 to 71 |
| 9. Line 88-89: Remove the sentence “this review has one…..” | Thank you for this suggestion | The statement, “This review had one main research question:” has been deleted in line 86 |
| 10. In eligibility criteria, what type of studies were considered for this article? I mean whether any case series etc.? | Observational studies like cross-sectional, case-control, cohort, etc., and experimental studies like randomized controlled trial were considered for inclusion in the scoping review | This has been revised in lines 95 to 96 |
| 11. Table 1: This table is not necessary and can be removed. Also, the number of studies not matching with the final studies included in the article. | The major results were presented here while the remaining was provided in the supplementary. | This table has been removed and added to the supplementary information S2 Table in line 786 |
| 12. Table 3, 4: replace the legend with “n: number of studies”. | Thank you for this observation | This has been revised in line 297 and 311 |
| 13. Table 5 is very confusing. The authors are recommended to redesign the table and split in 3 or 4. Some of the information can be described in the text. | Thank you for your recommendation | Table 5 has been divided into three separate tables, spanning lines 320 to 360. |
