# Supplementary material for: Antimicrobial-resistance of Escherichia coli in dogs and cats: A scoping review
Source: PLoS One. 2025 May 30;20(5):e0323246. doi: 10.1371/journal.pone.0323246 (PMC12124559; doi:10.1371/journal.pone.0323246)
Supplement: Attachment — Submitted filename: Response_to_Reviewers_auresp_2.docx [file pone.0323246.s011.docx]

Dear Editor,

We appreciate the opportunity to submit a revised version of our manuscript titled, “Antimicrobial-resistance of *Escherichia coli* in dogs and cats: a scoping review”.

A detailed response has been provided below this email to the query raised by the Editor regarding Figure 2.

Sincerely,

Rasaq Ojasanya

| **Comments** | **Responses and Rationale** | **Action taken/location of changes in manuscript**  (i.e., page and line number in manuscript with track changes) |
| --- | --- | --- |
| **Journal Requirement** |  |  |
| 1. Thank you for returning your submission, but we note you did not respond to our previous request for information on the creation of your Figure 2. We note you state the following: "I confirm that I am the original creator of Figure 2. I grant PLOS ONE permission to publish this figure under the Creative Commons Attribution License (CC BY 4.0)."  Please kindly clarify where the authors obtained the basemaps or what software was used to layer these images. | The shapefile of the world was retrieved from the Database of Global Administrative Areas’ website, and subsequently imported into R (Core Team, 2023), and this was used to represent the distribution of retrieved publications on the map. | These details were provided in lines 162 to 165. The reference of the website where the shape files were retrieved has been provided |
